# Fe-oxyhydroxide deposits at Semenov hydrothermal field (13°30′N), Mid-Atlantic ridge: insights into formation, modification and resource potential

**DOI:** 10.1007/s00126-025-01376-6

**Published:** 2025-08-01

**Authors:** Christian S. Bishop, Anna Lichtschlag, Stephen Roberts, Maxime Lesage, Bramley J. Murton

**Affiliations:** 1 School of Ocean and Earth Science, University of Southampton, Southampton, UK; 2 National Oceanography Centre, Southampton, UK; 3 Nedre Slottsgate 8–0157, Oslo, Norway

**Keywords:** Fe-oxyhydroxide, Weathering, Metal mobility, Seafloor massive sulphide deposits, Ultramafic, Mid-Atlantic ridge

## Abstract

Secondary Fe-oxyhydroxide (FeOOH) forms at seafloor massive sulphide (SMS) deposits through the oxidation of sulphide minerals by oxygenated seawater. Secondary FeOOH deposits are enriched in economically important metals, such as Cu and Zn, potentially representing an additional resource. However, how the metal content of secondary FeOOH evolves through post-formational modification at the seafloor, is poorly understood. Moreover, FeOOH can form as metal-poor primary precipitates, but our knowledge is insufficient to effectively discriminate between primary and secondary FeOOH at SMS deposits. At the Mid-Atlantic Ridge hosted Semenov hydrothermal field, primary FeOOH deposits are typically metal-depleted (< 0.4 wt% Cu + Ni + Zn, *n* = 6) and form layered chimney structures with alternating Mn-oxide and FeOOH bands, with the fluid conduit lined with green smectite. In contrast, secondary FeOOH deposits are enriched in Cu, averaging 2.40 wt% (*n* = 31), and exhibit diverse textural morphologies including chimney, brecciated, layered, ochre, ocherous and massive, inherited from the sulphide protolith. Secondary FeOOH behaves similarly to terrestrial gossans, acting as a metal trap through sorption or precipitation of metals released during underlying sulphide oxidation. A correlation between the Cu content of secondary FeOOH and that of sulphide suggests its potential use as a geochemical vector in exploration. Finally, the Cu content in the secondary FeOOH remains stable even under prolonged exposure to seawater at the seafloor, indicating that older, off-axis, buried and oxidised SMS deposits may still contain secondary FeOOH with appreciable amounts of Cu. Overall this study demonstrates the potential of secondary FeOOH as a potential metal resource and a tool to guide exploration at SMS deposits.

**Supplementary Information:**

The online version contains supplementary material available at 10.1007/s00126-025-01376-6.

## Introduction

Seafloor hydrothermal activity occurs in diverse tectonic settings, including Mid-Ocean Ridges (MOR), intra-oceanic volcanic arcs, back-arc spreading centres, ocean-continent transition and hotspots (Rona 1988; Hannington et al. 1994; Patten et al. 2022). Hydrothermal fluids often precipitate metal-rich minerals at and below the seafloor that can develop into Seafloor Massive Sulphide (SMS) deposits, which are of international interest due to their high metal content (e.g. Cu; Hannington et al. 2010; Monecke et al. 2016; Juliani and Ellefmo 2018). At SMS deposits, oxidation of sulphide minerals by oxygenated seawater produces secondary minerals such as Fe-oxyhydroxide (FeOOH), sulphates and atacamite (Herzig et al. 1991; Hekinian et al. 1993; Hannington 1993). This oxidative weathering mobilises potentially economically valuable metals from the primary sulphides and this process could diminish the metal content of SMS deposits over time. However, FeOOH also could retain these metals, potentially preserving the metal content of SMS deposits (Herzig et al. 1991; Melekestseva et al. 2020; Hu et al. 2022; Hou et al. 2024).

At SMS deposits, FeOOH deposits can be present in the form of chimneys, mounds, or crust covering sulphide structures (Hekinian et al. 1993; Melekestseva et al. 2020). FeOOH formation occurs through two processes: (1) as metal-depleted primary precipitates from low-temperature (< ~ 100^o^C), iron-rich hydrothermal fluids (Alt 1988; Hekinian et al. 1993; Gini et al. 2024), and (2) as metal-rich, secondary FeOOH, formed through the oxidation of sulphide by oxygenated seawater (Herzig et al. 1991; Hekinian et al. 1993). Despite extensive studies of SMS deposits, few studies investigate the variability of FeOOH across a SMS deposit, and consequently, our understanding on their formation mechanisms and metal content is incomplete. It remains uncertain whether primary or secondary mechanisms of FeOOH formation dominate at SMS deposits (Hekinian et al. 1993). Clarifying this distinction is crucial because, similarly to terrestrial gossans (e.g., Constantinou 1972), secondary FeOOH may indicate underlying sulphide and provide clues to the metal content of the sulphide, serving as a valuable tool for targeting exploration and as an additional resource. However, post-formational modification may alter the metal content of secondary FeOOH. For example, when secondary FeOOH is exposed to seawater, competing cations may displace adsorbed economic metals (Balistrieri and Murray 1982; Calmano et al. 1988). Consequently, extensive seawater influence may reduce economic metal concentrations in secondary FeOOH, impacting the resource potential of these products. To address these questions, this study focuses on the Semenov hydrothermal field, an ultramafic-hosted SMS cluster located at 13^o^ 30’N on the Mid-Atlantic Ridge (Beltenev et al. 2007). The Semenov field comprises of five hydrothermal areas each containing exposed sulphide and FeOOH material (Beltenev et al. 2007; Escartín et al. 2017; Murton 2022). Our objectives are to: (1) discriminate primary and secondary FeOOH deposits across the Semenov hydrothermal field; (2) assess how seawater interaction can influence the economic metal content (i.e., Cu and Zn) of secondary FeOOH, and; (3) evaluate the potential of the secondary FeOOH as an exploration tool for identifying high-grade sulphide deposits.

### Geological setting

**Fig. 1:**
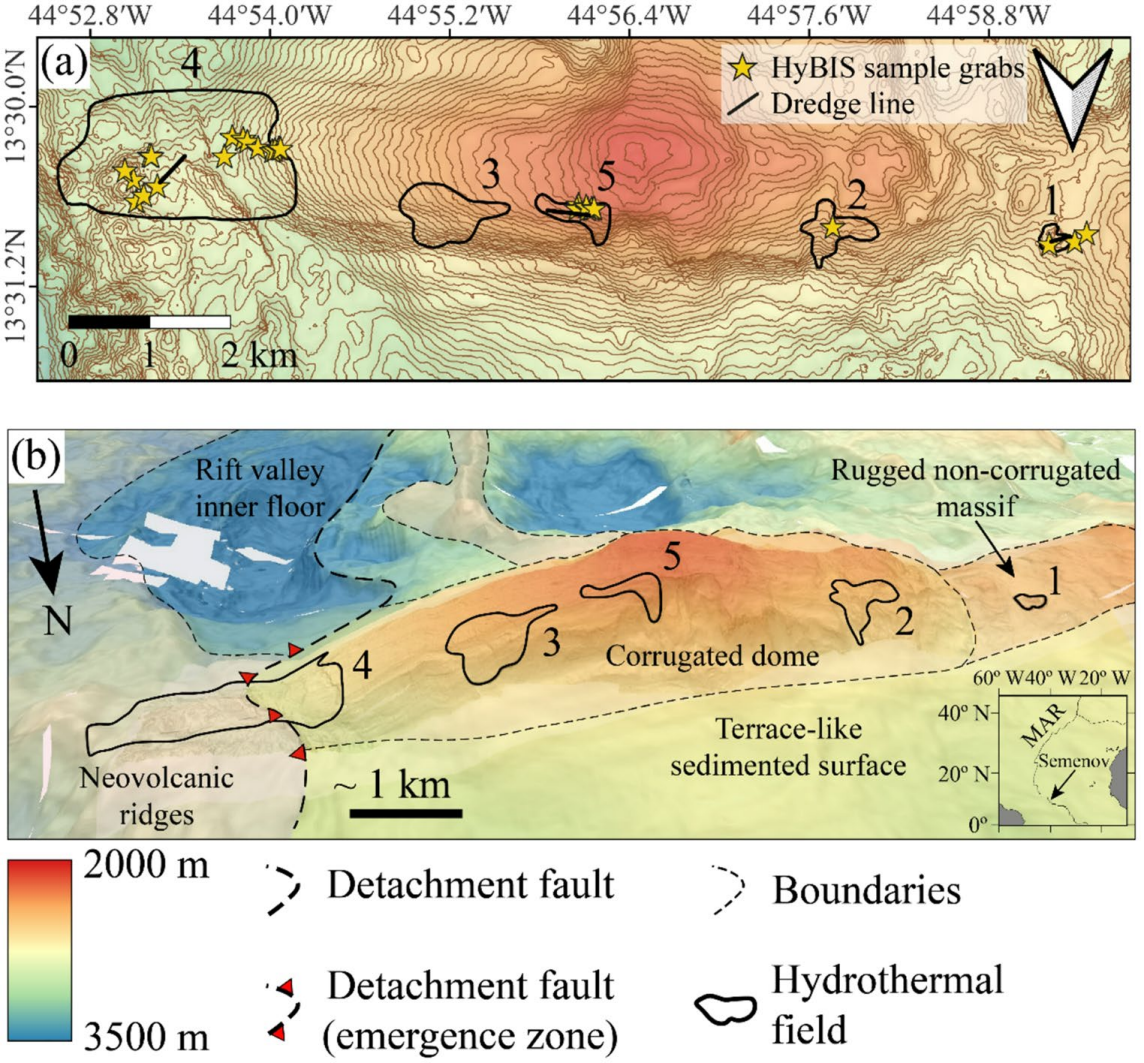
Location map of the Semenov on the Mid-Atlantic Ridge. **(a)** Bathymetric map of Semenov showing the location of hydrothermal fields. **(b)** 1.5x vertical exaggeration 3D bathymetry map of Semenov illustrating major tectonic features and the location of hydrothermal mounds. The inset image shows the regional location of Semenov on the MAR. Bathymetry after Escartín (2014), with tectonic settings, boundaries and location of hydrothermal mounds after Firstova et al. (2022) and Pertsev et al. (2012). The OCC is made up of the corrugated dome, rugged non-corrugated massive and terrace-like sedimented surface. Numbers represent a hydrothermal cluster (i.e., 1 is Semenov-1 and so on)

The Semenov hydrothermal field is located at a water depth of between 2480 and 2950 m below sea level, on an E-W elongate, dome structure that is 10 km long and 4.5 km wide (Fig. 1; Smith et al. 2006; Beltenev et al. 2007; Smith et al. 2008; Beltenev et al. 2009). Rocks previously sampled from the field include harzburgites, olivine gabbros, gabbronorites, gabbros, ferrograbbros, plagiogranites, tonalites, diorites and basalts (Pertsev et al. 2012). The occurrence of ultramafic lithologies at the seafloor and dome structure with a characteristic striated surface, is indicative of an oceanic core complex (OCC; MacLeod et al. 2009). Semenov consists of five distinct hydrothermal areas; four inactive (Semenov-1, Semenov-3, Semenov-4 and Semenov-5) and one active (Semenov-2; Pertsev et al. 2012; Cherkashov et al. 2013). ^230^U/Th dating of massive sulphide samples indicate hydrothermal activity at Semenov initiated at ~ 124 ka before present (Kuznetsov et al. 2011; Cherkashov et al. 2016).

Sulphide recovered from Semenov consist mainly of iron sulphides (pyrite, pyrrhotite and marcasite) and copper sulphides (chalcopyrite, isocubanite and secondary copper sulphides), with pyrite-marcasite breccias, cemented by microcrystalline silica, aragonite, quartz or barite (Beltenev et al. 2009; Firstova et al. 2019; Melekestseva et al. 2014, 2018). Alteration products include covellite (CuS), atacamite, FeOOH and jarosite (Melekestseva et al. 2014, 2017a).

## Methods

### Seafloor sampling

Forty-two samples were collected during the project “ULTRA” with the *RRS James Cook* (JC224; 2022; Murton 2022). Samples from Semenov-1, Semenov-2, Semenov-4 and Semenov-5 were recovered at the seafloor using the Hydraulic Benthic Interactive Sampler (HyBIS) robotic underwater vehicle (Murton et al. 2012). Additional samples were acquired by dredging across Semenov-1 and Semenov-4. A full list of sample locations and descriptions can be found in electronic supplementary materials (ESM) 1.

### Reflective light microscopy

The mineralogy, textures and paragenesis of FeOOH and massive sulphide samples were observed with reflective light microphotography with a LEICA DM2700 P microscope. Images were captured by the LEICA DMC 4500 and processed with the Leica Application Suite X software.

### X-ray Diffraction (XRD)

The mineralogical composition of a total of 30 samples were analysed by XRD. Each sample was prepared by crushing of the material with an agate mortar and pestle to approximately < 250 μm and weighing out 1.5 g of material, with an addition of 0.5 g of corundum to act as an internal standard. Samples were ground further in a McCrone mill with agate grinding elements for 8 min with isopropanol to form a slurry. This slurry was dried and side-loaded into XRD sample holders to mitigate any effects from preferred orientation. XRD analysis was undertaken at the University of Southampton using a PANalytical X’Pert Pro diffractometer with a Cu X-ray source and Fe filter set at an accelerating voltage of 35 kV and beam current of 40 mA utilising automatic slits. ​The X-ray diffraction (XRD) analysis was conducted using a step size of 0.02° 2θ over a range of 2.01° to 75.99° 2θ. The time per step was set to either one or two seconds, depending on the number of X-ray counts recorded. Detection limits for identifying mineral phases of crystalline materials were generally 2.0 ± 0.5% volume.

### Major and trace element analyses

Bulk geochemical analysis was performed on 42 samples consisting of FeOOH (*n* = 37) and massive sulphide (*n* = 5) at the University of Southampton by Inductively Couple Plasma-Mass Spectrometry (ICP-MS; Agilent 8900) and the National Oceanography Centre with Inductively Coupled Plasma-Optical Emission Spectrometry (ICP-OES; iCAP 6000). Bulk concentration of Na, Mg, Al, Ca, Mn, Fe, Cu and Sr were obtained by ICP-OES with the exception of Mn where the concentration was below 1000 ppm. Bulk concentrations of Li, Mn (below 1000 ppm), Ti, Co, Ni, Zn, As, Nb, Cd, Sb, Ba, REE, W, Pb, Bi, Th and U were obtained by ICP-MS. Bulk samples of approximately 50 mg were crushed using an agate mortar and pestle and then completely dissolved in a mixture of hydrofluoric acid, nitric acid and hydrochloric acid. This solution was dried down and re-dissolved in 20 mL of 6 M hydrochloric acid. Approximately 1 mL of this solution was dried down and made up to 20 mL with spiked 3% nitric acid. Full acid digestion method can be found in ESM2.1.

Accuracy and precision were determined by triplicate measurements of certified reference material (CRM) of JMn-1, RTS-1, GSPN-2, except for Fe, Ni, Mg and Ti, which were measured in duplicate for RTS-1 and GSPN-2. Further single measurements of GSPN-3, CH-4, Nod-A-1 and Nod-P-1 CRM were taken to ascertain accuracy. Signal drift in ICP–MS measurements is corrected by continuously monitoring two internal standards (Re–In at 5 ppb and Be at 20 ppb) spiked into every sample in 3% nitric acid. For the ICP–OES, we ran a calibration standard after every ten measurements to correct for any instrumental drift. Of the elements discussed in the study, major and trace element measurements exhibited good accuracy, the majority of elements were within 10% error of the expected values, except for Mg (± 13.0%), Ca (± 11.7%), Co (± 17.6%), Zn (± 10.0%) and La (± 11%). Precision exhibited good reproducibility with elements showing a standard deviation of ≤ 10.0%, except for As (± 14.5%). See ESM2.2 for more details.

### Sr-Nd-Pb isotope systematics

Neodymium and Sr were extracted from each sample using two-column passes: AG50-X8 200–400 mesh for the initial separation, followed by Sr-spec resin for Sr and LN Spec columns for Nd. Pb was separated using AG1 × 8 anion exchange resin, replicating the method of Lang et al. (2014). Pb isotopes were corrected for instrumental mass fractionation with the SBL74 spike ^207^Pb/^204^Pb (Taylor et al. 2015). This analysis was conducted at the University of Southampton using a Thermo Scientific Neptune Multi-Collector Inductively Coupled Plasma Mass Spectrometer (MC-ICP-MS).

Three CRMs were used for Nd isotope systematics (Nod-A-1, Nod-P-1 & BHVO-1) with internal standard JNdi-1. Two CRMs (Nod-A-1, Nod-P-1) with standard reference material NBS981 were used for Pb isotope systematics, and one CRM (BHVO-1) with internal standard NBS987 for Sr isotope fractionation. Measurements for BHVO-1, Nod-A-1 and Nod-P-1 all fall within the range of published values for each isotope systematic. Lead isotope standard reference material NBS981 showed ^206^Pb/^204^Pb = 16.9400 and a two-standard error (2SE) of 0.0023, ^207^Pb/^204^Pb = 15.4965 with a 2SE of 0.0026, and ^208^Pb/^204^Pb = 36.7124 with a 2SE of 0.0076 (from > 100 analyses over the past 4 years). The Nd internal standard JNdi-1 averaged 0.512117 with a 2SE of 0.000006 (*n* = 5), while NBS987, used for Sr measurements, averaged 0.710272 with a 2SE of 0.000010 (*n* = 6).

### End-member mixing

To account for the impact of sediment on the isotopic ratio of Pb within the FeOOH samples, the proportion of pelagic sediment in the FeOOH sample was assessed using binary mixing plots based on the Al/(Al + Fe + Mn) ratio as described by Boström (1973) to identify detrital input and Ca for carbonate within the pelagic sediment. This is done in order to create an accurate fluid-to-rock (F/R) ratio that accounts for the contamination of pelagic sediment within a given sample. By utilising massive sulphide samples from this study and North Atlantic carbonate ooze sediment composition after Menendez et al. (2017) as two endmembers, the resulting mixing curve allows to determine the percentage of the sedimentary component in a sample. The general form of a binary mixing equation adapted from Faure (1998) is used to quantify the contribution of pelagic sediment to each FeOOH sample using Eq. 1 and Eq. 2. Equation 1 estimates the proportion of detrital material using the Al/(Al + Fe + Mn) ratio, where PS_DC_ and MS_DS_ represent the Al/(Al + Fe + Mn) ratios of pelagic sediment and massive sulphide, respectively. PS_f_ and MS_f_ are the mixing fractions of pelagic sediment and massive sulphide, while Al, Fe, and Mn are the concentrations (in ppm) of each element in the respective endmembers.

1 $$\begin{array}{l}{\left(\frac{Al}{Al+Fe+Mn}\right)}_{mix}=\\\frac{\left({PS}_{DC}\times\left({{Al}_{PS}+{Fe}_{PS}+Mn}_{PS}\right)\times{PS}_f\right)+\left({MS}_{DC}\times\left({{Al}_{MS}+{Fe}_{MS}+Mn}_{MS}\right)\times{MS}_f\right)}{\left({{Al}_{PS}+{Fe}_{PS}+Mn}_{PS})\times{PS}_f\right)+\left({{Al}_{MS}+{Fe}_{MS}+Mn}_{MS})\times{MS}_f\right)}\end{array}$$

To account for carbonate input, Ca concentrations are also evaluated in Eq. 2, based on Faure (1998). In this equation Ca_PS_ and Ca_MS_ are the Ca concentrations (ppm) in the pelagic sediment and massive sulphide endmembers, respectively, and PS_f_ represents the sediment fraction.

2 $${Ca}_{mix}={PS}_f\times{Ca}_{ps}+(1-{PS}_f)\times{Ca}_{MS}$$

### Seawater mixing

Lead isotopic compositions in FeOOH can reflect the interaction between seawater and FeOOH and can be used to determine how this interaction may affect metal concentration. This interaction between FeOOH and seawater is expressed as the F/R ratio and represents the amount of seawater required to isotopically modify FeOOH from a massive sulphide signature toward a seawater-like composition. To estimate the F/R of secondary FeOOH, a binary mixing model is used, with massive sulphide and seawater as two endmembers. The position of the FeOOH sample along this mixing curve reflects the degree of Pb isotope exchange, and thus the F/R ratio required to shift the isotopic composition from that of the massive sulphide toward seawater. The concentration of Pb in seawater is acquired after Bridgestock et al. (2018) from a bottom water sample to the south-east of the Semenov field (station 16, 8.20°N, 28.20°W). The Pb isotopic ratios of seawater is derived from Abouchami et al. (1999).

Compilation of mixing models were developed using two component mixing equations from Faure (1998; Eq. 3 and Eq. 4). Equation 3 calculates the isotopic composition of a mixture between sulphide and pelagic sediment. Using ^206^Pb/^204^Pb as an example, ^206^Pb/^204^Pb_MS_ and ^206^Pb/^204^Pb_PS_ are the isotopic signatures of the sulphide and pelagic sediment endmembers, respectively; Pb_MS_ and Pb_PS_ are their Pb concentrations (ppm); and f_MS_ and f_PS_ are their respective mixing fractions. The fraction of sediment within each sample is obtained by the sulphide-pelagic sediment mixing line derived from Eq. 1 and Eq. 2.

3 $$\begin{array}{l}{\frac{206Pb}{204Pb}}_{MIX}=\\\frac{\left({\frac{206Pb}{204Pb}}_{MS}\times{Pb}_{MS}\times f_{MS}\right)+\left({\frac{206Pb}{204Pb}}_{PS}\times{Pb}_{PS}\times f_{PS}\right)}{\left({Pb}_{PS}\times f_{MS}\right)+\left({Pb}_{MS}\times f_{PS}\right)}\end{array}$$

The isotopic composition derived from Eq. 3 represents the Pb isotope ratio of a mixture between massive sulphide and pelagic sediment. This mixture is then treated as a single endmember, with seawater serving as the second endmember in subsequent mixing calculations. Equation 4 is used to generate a final Pb isotopic ratio between the sulphide–sediment mixture and seawater to generate a binary mixing curve. In this equation, ^206^Pb/^204^Pb_MIX_ and ^206^Pb/^204^Pb_SW_ represents the isotopic ratios of sulphide-sediment and seawater, respectively; Pb_MIX_ and Pb_SW_ are the corresponding Pb concentrations; and f_MIX_ and f_SW_ are their respective mixing fractions. The position of the Pb isotope ratio of the FeOOH sample along the mixing curve from Eq. 4 will derive a quantitative F/R that accounts for the contamination of sediment.

4 $$\begin{array}{l}{\frac{206Pb}{204Pb}}_{Final}=\\\frac{\left({\frac{206Pb}{204Pb}}_{MIX}\times{Pb}_{MIX}\times f_{MIX}\right)+\left({\frac{206Pb}{204Pb}}_{SW}\times{Pb}_{SW}\times f_{SW}\right)}{\left({Pb}_{SW}\times f_{SW}\right)+\left({Pb}_{MIX}\times f_{MIX}\right)}\end{array}$$

## Results

### Morphology, lithology and mineralogy of FeOOH deposits at Semenov

**Fig. 2:**
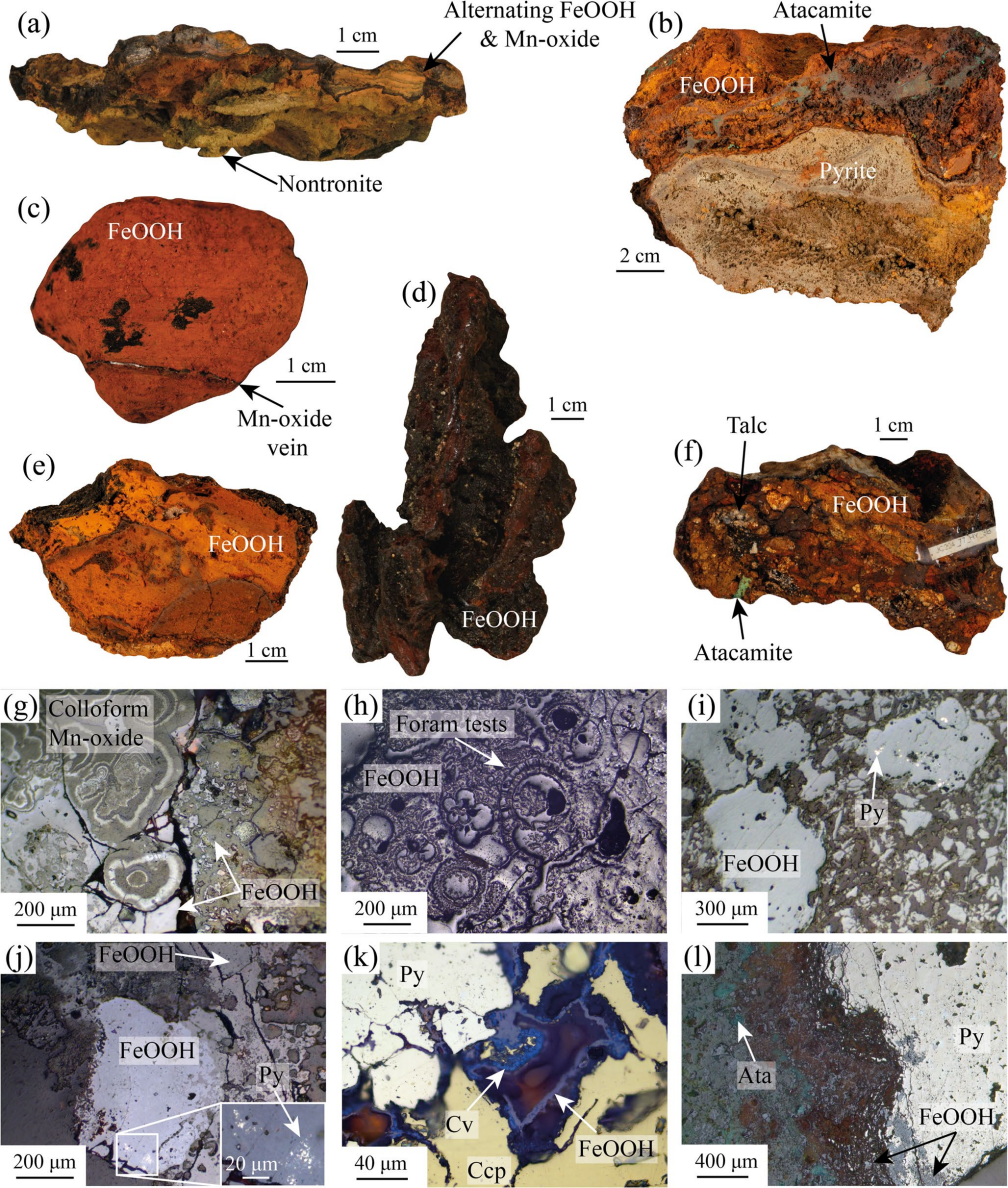
Representative hand specimen photos and reflected light photomicrographs of samples from Semenov hydrothermal field. **(a)** Type-I chimney FeOOH composed of nontronite with alternating layers of FeOOH and Mn-oxides (71_DR_01). **(b)** Massive FeOOH with actively weathering massive sulphide and an FeOOH crust comprising of FeOOH and atacamite (82_HY_06). **(c)** Type-II ochre FeOOH comprise of orange, soft, fine grains with thin layers (< 1 mm) of alternating darker and lighter FeOOH (28_DR_18). **(d)** Type-II chimney with dark red to orange FeOOH and thin veins (~ 1 mm) of Mn-oxides (102_HY_04). **(e)** Ocherous FeOOH comprise of fine grained FeOOH (82_HY_05). **(f)** Brecciated FeOOH of predominately FeOOH with minor quantities of atacamite and talc, quartz, and barite (77_HY_03). **(g)** Colloform Mn-oxides growth adjacent to FeOOH shown by the granular, subhedral–anhedral texture (71_DR_07). **(h)** Multiple foraminifera tests replaced by FeOOH (71_DR_03). **(i)** Grains of FeOOH with one grain containing relict pyrite indicating FeOOH grain is secondary. FeOOH form as pseudomorphs of pyrite crystals (82_HY_05). **(j)** Relict pyrite within large grain of FeOOH surrounded by FeOOH (71_DR_01). **(k)** Subhedral to anhedral pyrite set beside chalcopyrite. Covellite occurs adjacent to chalcopyrite and is typically encased by FeOOH (82_HY_06). **(l)** Redox front separating massive pyrite and FeOOH. Close to redox front, pyrite is replaced to FeOOH, at the redox front, pyrite forms as brecciated pyrite set in FeOOH. Atacamite vein is observed within FeOOH (82_HY_06_b_). Mineral abbreviations: Py – Pyrite, Cv – covellite, Ccp – chalcopyrite

The FeOOH deposits samples at Semenov have various textural morphologies, including chimney, layered, ochre, brecciated, massive and ocherous material (Fig. 2; ESM1). Results of combined XRD, hand sample observations and reflective microscopy indicate that all FeOOH samples contain x-ray amorphous FeOOH and goethite (α-FeOOH) with the majority of samples containing a minor amount (< 5% by volume) of quartz. Broad peaks of goethite in some samples (e.g., 82_HY_01) suggest over milling, but does not result in the formation of amorphous FeOOH. Atacamite is present in eight FeOOH samples, typically as veins (Fig. 2b; see ESM3 for full XRD data).

FeOOH deposits with chimney textural morphologies are subdivided into two types; Type-I is only found at Semenov-1 and comprise of pale brown to yellow alternating layers of FeOOH and Mn-oxide. Type-I chimney deposits also have a coating of fine grained, green smectite on the inside of the fluid conduit (Fig. 2a), which, based on visual observations, is likely nontronite (Murnane and Clague 1983; Dekov et al. 2007). Type-I chimneys also contain minor amounts of quartz, barite and calcite. Reflected light microscopy reveals colloform Mn-oxide textures, rare relict pyrite surrounded by FeOOH and foraminifera tests replaced by FeOOH (Fig. 2 g, h and j). Pyrite remnants are typically found within specific FeOOH grains (Fig. 2j), as such, the pyrite remnants are likely pyrite that have not yet been altered to FeOOH. Type-II chimneys at Semenov-4 comprise dark brown to dark red FeOOH that forms fluid conduit structures with minor amounts of atacamite, akageneite, calcite, barite and quartz (Fig. 2 d).

FeOOH deposits with layered textural morphologies are subdivided into two types with Type-I at Semenov-1 comprising of < 1 cm alternating horizontal layers of pale brown to yellow FeOOH and Mn-oxide with minor amount of talc. Type-II layered morphologies lack the Mn-oxide layers, but instead exhibit < 1 cm layers of light orange to dark red FeOOH (ESM1). Atacamite within type-II layered morphologies form as veins conformable to layering or as precipitates on the sample.

FeOOH deposits with ochre textural morphology are relatively soft compared with other textural morphologies and contain orange, granular FeOOH that is moderately sorted to well sorted with minor to trace amounts of quartz, barite, calcite, mackinawite and hematite. Ochre textural morphologies exhibit either a homogenous texture (Type-I) or form alternating lamination of pale orange to orange FeOOH (Type-II; Fig. 2c). While similar to ochre, ocherous textural morphologies are more consolidated and mechanically competent, often displaying a denser fabric and darker colouration ranging from dark brown to brownish orange. Ocherous textural morphologies are composed of granular FeOOH with minor quartz and pyrite, and exhibit internal veining. Ochreous samples may also exhibit subtle layering of orange to orange-brown FeOOH (Fig. 2e).

Brecciated FeOOH deposits exhibit clasts set in a matrix of FeOOH and are subdivided into two types. Type-I with clasts of angular, dark red to orange FeOOH and veins of atacamite within the matrix with minor to trace amounts of Mn-oxide and akageneite acquired at Semenov-1 and Semenov-5. Type-II brecciated deposits at Semenov-2 occur as clasts of rounded to sub-angular clasts of barite, talc, quartz and FeOOH (Fig. 2f). Brecciated deposits contain frequent pyrite with a single chalcopyrite crystal observed. Massive FeOOH deposits have no obvious textural morphology and are dominated by FeOOH with minor amounts of quartz, calcite, atacamite, Mn-oxide, paratacamite, and relict pyrite.

Massive sulphide samples contain pyrite (> 50% by volume), followed by minor amounts of chalcopyrite, sphalerite, FeOOH ± barite ± atacamite, and trace quantities of covellite replacing chalcopyrite (Fig. 2k and l). Atacamite is observed within FeOOH and not in close association with sulphide minerals (Fig. 2 l).

One sample comprises of both massive sulphide and FeOOH that forms as a crust on top of massive sulphide (Fig. 2b). The massive sulphide is dominated by pyrite (> 80% by volume), with minor amounts of chalcopyrite and FeOOH with trace amounts of sphalerite, atacamite and covellite. FeOOH typically occurs as a possible redox front with pyrite (Fig. 2 l). The FeOOH crust is dominated by FeOOH with atacamite making up approximately 10% by volume of the crust. Relict pyrite within grains of FeOOH are rare and have a similar appearance as relict pyrite observed in Fig. 2i and j.

### Bulk major and trace elemental composition

The geochemical composition of FeOOH deposits and sulphides from Semenov are summarised in Table 1, with details of each sample shown in ESM2.3. The chemical composition of FeOOH deposits, regardless of morphology, is highly variable in Cu, Fe, and Mn with variations in Mg, Ca, Al, Zn, Ti, Li, Co and As (Table 1). The highest concentration of Cu is found in a massive FeOOH sample (82_HY_06_a_) with a concentration of 16.79 wt% with the lowest concentration at 0.04 wt% (86_HY_08). Zn is less variable, with the highest measured concentration of 0.55 wt% in an ochre sample (86_HY_09) and the lowest measured concentration of 0.01 wt%.

**Table 1:** Average contents of major and trace elements in FeOOH and massive sulphide. < lod = below limit of detection. ± is 1 standard deviation. Where *n* = 2, the range of composition is given

	Mg (wt.%)	Al (wt.%)	Ca (wt.%)	Mn (wt.%)	Fe (wt.%)	Cu (wt.%)	Li (ppm)	Ti (ppm)
Type-I chimney (n=5)	0.71 ± 0.04	0.17 ± 0.06	0.78 ± 0.15	7.76 ± 2.72	21.87 ± 1.84	0.14 ± 0.08	107 ± 29	100 ± 50
Type-II chimney (n=3)	0.33 ± 0.07	0.14 ± 0.12	0.58 ± 0.48	0.29 ± 0.39	35.04 ± 2.74	0.73 ± 1.11	1.2 ± 0.2	70 ± 70
Type-I layered (n=1)	1.06 ± 0.07	0.32 ± 0.03	0.67 ± 0.00	7.51 ± 0.01	20.48 ± 0.03	0.14 ± 0.00	102 ± 1.7	240 ± 10
Type-II layered (n=6)	0.50 ± 0.20	0.18 ± 0.11	0.72 ± 0.40	2.26 ± 2.72	33.40 ± 4.11	3.12 ± 4.40	11 ± 15	170 ± 130
Type-I brecciated (n=2)	0.75–0.61	0.15–0.24	0.72–1.00	0.19–0.62	30.00–34.68	1.84–2.48	3.6–3.9	330–120
Type-II brecciated (n=2)	2.05–0.89	0.46–1.00	0.56–1.53	1.38–2.48	18.65–24.64	0.87–6.90	3.0–7.1	180–510
Ocherous (n=3)	0.72 ± 0.15	0.22 ± 0.04	0.92 ± 0.44	0.21 ± 0.15	31.74 ± 2.62	1.56 ± 1.12	3.9 ± 2.9	80 ± 30
Type-I & II Ochre (n=6)	0.64 ± 0.34	0.57 ± 0.64	0.68 ± 0.45	0.63 ± 0.68	28.87 ± 4.70	2.42 ± 1.90	3.6 ± 3.6	420 ± 510
Massive FeOOH (n=9)	0.68 ± 0.29	0.38 ± 0.29	1.99 ± 1.65	2.12 ± 2.86	31.10 ± 3.54	2.46 ± 5.41	13 ± 21	360 ± 390
Massive sulphide (n=5)	0.03 ± 0.03	0.04 ± 0.05	0.08 ± 0.16	<0.01	32.64 ± 2.19	4.75 ± 6.73	0.2 ± 0.2	10 ± 10

	Co (ppm)	Zn (ppm)	As (ppm)	Sr (ppm)	Pb (ppm)	Bi (ppm)	U (ppm)	Al/(Al+Fe+Mn)
Type-I chimney (n=5)	35 ± 26	840 ± 330	87 ± 44	310 ± 100	19 ± 12	0.15 ± 0.21	2.0 ± 1.6	0.0055 ± 0.0016
Type-II chimney (n=3)	45 ± 36	2420 ± 990	207 ± 45	230 ± 150	293 ± 295	0.06 ± 0.02	25.6 ± 24.3	0.0039 ± 0.0020
Type-I layered (n=1)	111 ± 1	840 ± 10	199 ± 1	480 ± <10	51 ± 0.5	1.2 ± 2.0	19.3 ± 15.2	0.0113
Type-II layered (n=6)	240 ± 212	1620 ± 870	286 ± 262	240 ± 120	190 ± 175	1.12± 2.0	19.3 ± 15.2	0.0061 ± 0.0038
Type-I brecciated (n=2)	38–40	310–1060	241–329	210–270	16–190	0.2–15.1	5.3–16.7	0.0050–0.0068
Type-II brecciated (n=2)	59–139	130–290	159–171	490–1180	57–284	1.0–2.2	75–7.7	0.0168–0.0476
Ocherous (n=3)	29 ± 22	1940 ± 1750	469 ± 113	290 ± 50	351 ± 84	2.7 ± 3.7	13.6 ± 7.7	0.0067 ± 0.0008
Type-I & II Ochre (n=6)	82 ± 83	1900 ± 1920	423 ± 283	620 ± 240	265 ± 193	1.3 ± 1.0	10.4 ± 4.9	0.0212 ± 0.0273
Massive FeOOH (n=9)	95 ± 80	1300 ± 590	300 ± 128	440 ± 270	167 ± 143	2.1 ± 3.4	18.1 ± 12.9	0.0115 ± 0.0102
Massive sulphide (n=5)	111 ± 241	1900 ± 1010	214 ± 39	190 ± 260	156 ± 58	1.6 ± 2.4	4.1 ± 1.8	0.0013 ± 0.0013

Manganese and Li are enriched in Type-I chimney and Type-I layered deposits relative to other textural morphologies, whereas Fe and Cu are depleted in Type 1 chimney and Type 1 layered deposits. Type-II chimney, Type-II layered brecciated, massive, ochre and ocherous deposits exhibit similar contents of Al, Ca, Cu, Ti and Zn (Table 1).

Massive sulphide samples exhibit greater variation in Cu concentrations compared to Zn and As concentrations (Table 1). Relative to barite-free massive sulphide samples, barite-rich massive sulphides are strongly enriched in Cu (6.03–15.23 wt% vs. 0.11–2.01 wt%), and depleted in Fe (28.96–29.25 vs. 33.7–34.6 wt%).

### REE concentrations and correlation patterns

**Fig. 3:**
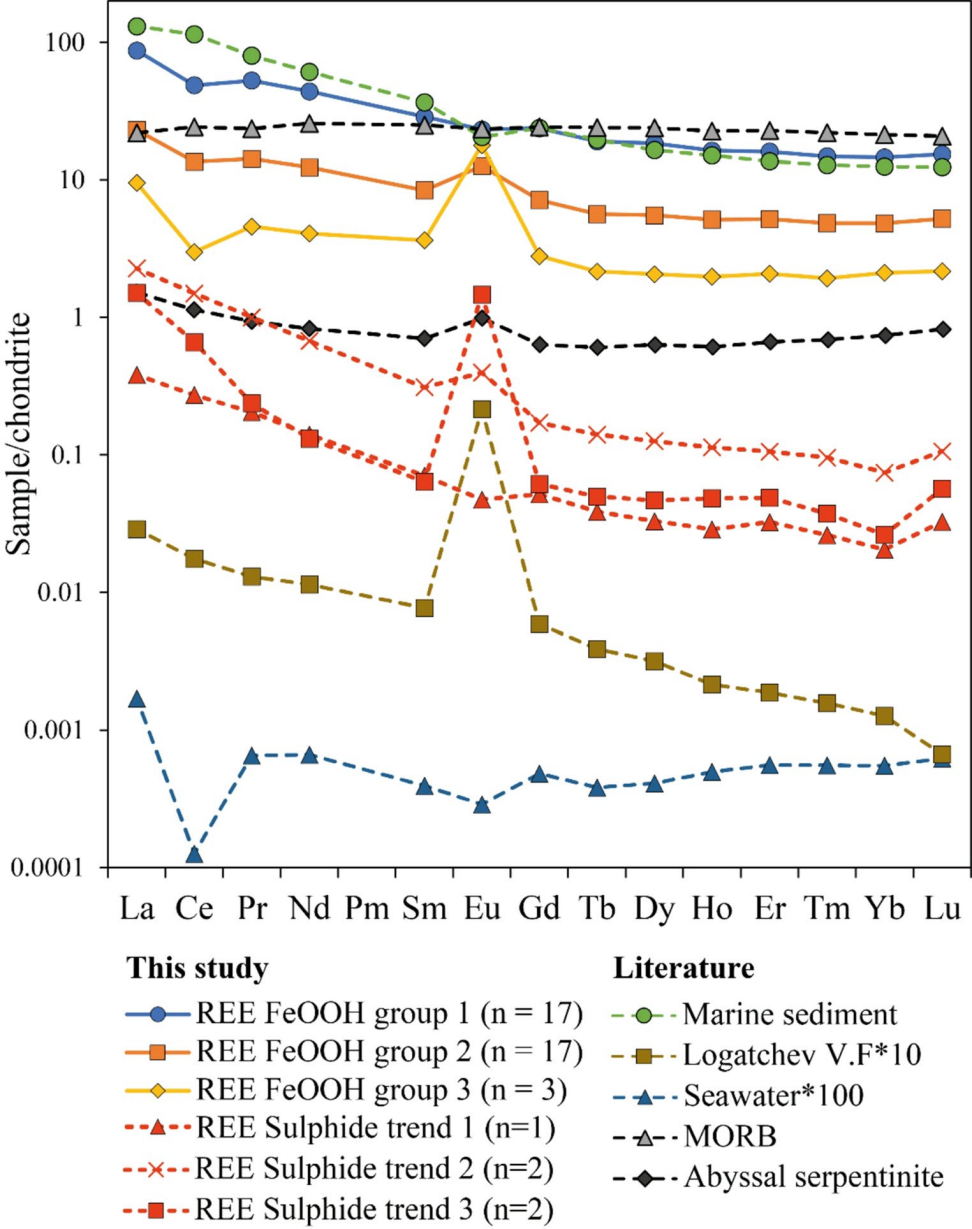
Chondrite-normalised (Barrat et al. 2012) REE distribution patterns of FeOOH and massive sulphide samples collected at Semenov. Seawater obtained from Douville et al. (1999), Logatchev vent fluid obtained from Schmidt et al. (2007), marine sediment obtained from Menendez et al. (2017), Mid Ocean Ridge Basalt (MORB) data from Gale et al. (2013) and abyssal serpentinite data after Debret et al. (2024). Note, the data from nearby ultramafic hosted Logatchev vent fluid are used as there is no published data on the composition of hydrothermal vent fluid at Semenov

REE patterns of FeOOH from Semenov are categorised into three groups based on similar REE pattern regardless of textural morphologies or whether primary or secondary FeOOH deposits, and are shown in Table 2; Fig. 3 (see also ESM2.4 and ESM4). REE FeOOH group 1 is defined by no or slightly negative Eu anomaly, weakly negative Ce anomaly and a considerable variation in ΣREE content (Table 2). REE FeOOH group 2 is characterised by a positive Eu anomaly, a weak negative Ce anomaly and lower average ΣREE concentration relative to REE FeOOH group (1) REE FeOOH group 3 has a strong positive Eu anomaly, a negative Ce anomaly and typically lower average ΣREE concentrations relative to REE FeOOH groups 1 and (2) The ΣREE concentrations of REE FeOOH group 1 exhibit a strong correlation with detrital elements such as Al, Al/(Al + Fe + Mn; Boström 1973) and Ca (R^2^ = 0.83, 0.67 and 0.81 respectively) and a moderate correlation with Ti (R^2^ = 0.45; ESM4). REE FeOOH group 2 show moderate positive correlations of ΣREE with Ti and Al/(Al + Fe + Mn; R^2^ = 0.66 and 0.60 respectively) and a weak negative correlation with Fe/Ti ratio (R^2^ = 0.42; ESM4). Due to only three samples in REE FeOOH group 3, no correlation analyses were performed. However, relative to REE FeOOH group 2 and 1, REE FeOOH group 3 is strongly depleted in Ti (average of 30 ppm vs. 160 ppm and 400 ppm) and the Al/(Al + Fe + Mn) ratio (0.0036 vs. 0.0098 and 0.0122).

**Table 2:** Contents of REE in FeOOH and massive sulphides samples at semenov. Both FeOOH and massive sulphide are categorised into three REE groups based on their REE trend. Sulphide group 1 and 3 only have two samples and the values depict the range. Ce and Eu anomalies (Ce* and Eu*) are calculated after McLennan (1989) using the following formula Ce* = Ce_n_/(La_n_*Pr_n_)*0.5. Eu* = Eu_n_/(Sm_n_*Gd_n_)*0.5. Elements with Xn indicate chondrite normalised

	La (ppm)	Ce (ppm)	Pr (ppm)	Nd (ppm)	Sm (ppm)	Eu (ppm)	Gd (ppm)	Tb (ppm)	Dy (ppm)	Ho (ppm)
REE FeOOH group 1 (n=17)	20.7 ± 19.7	29.8 ± 25.8	5.0 ± 4.9	20.6 ± 19.8	4.4 ± 4.2	1.3 ± 1.1	4.9 ± 4.4	0.7 ± 0.6	4.7 ± 4.0	0.9 ± 0.8
REE FeOOH group 2 (n=17)	5.5 ± 3.2	8.3 ± 6.0	1.4 ± 0.8	5.8 ± 3.5	1.3 ± 0.8	0.7 ± 0.4	1.5 ± 0.9	0.2 ± 0.1	1.4 ± 0.9	0.3 ± 0.2
REE FeOOH group 3 (n=3)	2.3 ± 0.7	1.8 ± 0.7	0.4 ± 0.2	1.9 ± 1.0	0.6 ± 0.3	1.0 ± 0.3	0.6 ±−0.3	0.08 ± 0.05	0.5 ± 0.3	0.11 ± 0.06
REE Sulphide trend 1	0.1 ± 0.0	0.2 ± 0.0	0.02 ± 0.0	0.1 ± 0.0	0.01 ± 0.00	0.003 ± 0.000	0.01 ± 0.00	0.001 ± 0.000	0.01 ± 0.00	0.002 ± 0.000
REE Sulphide trend 2	0.2–0.9	0.5–1.4	0.05–0.14	0.2–0.5	0.03–0.07	0.02–0.03	0.02–0.05	0.003–0.008	0.01–0.01	0.003–0.009
REE Sulphide trend 3	0.2–0.5	0.2–0.6	0.01–0.03	<0.1–0.1	0.01–0.02	0.02–0.04	0.01–0.02	0.002–0.003	0.01–0.01	0.003–0.003

	Tm (ppm)	Yb (ppm)	Lu (ppm)	ΣLREE (ppm)	ΣHREE (ppm)	ΣREE (ppm)	Ce anomaly	Eu anomaly	La_n_/Yb_n_
REE FeOOH group 1 (n=17)	0.4 ± 0.3	2.5 ± 1.9	0.4 ± 0.3	80.5 ± 74.0	18.4 ± 15.3	98.9 ± 89.2	0.71 ± 0.21	0.90 ± 0.17	5.8 ± 1.3
REE FeOOH group 2 (n=17)	0.12 ± 0.08	0.8 ± 0.6	0.13 ± 0.10	25.2 ± 13.6	7.5 ± 3.7	32.8 ± 17.0	0.71 ± 0.27	1.68 ± 0.24	5.9 ± 2.9
REE FeOOH group 3 (n=3)	0.05 ± 0.03	0.4 ± 0.2	0.05 ± 0.03	7.0 ± 3.0	3.1 ± 1.4	10.1 ± 4.4	0.47 ± 0.05	8.31 ± 4.70	5.2 ± 1.4
REE Sulphide trend 1	0.001 ± 0.000	0.003 ± 0.000	0.001 ± 0.000	0.35 ± 0.000	0.04 ± 0.000	0.39 ± 0.000	0.97 ± 0.00	0.78 ± 0.00	18.7 ± 0.0
REE Sulphide trend 2	0.001–0.000	0.007–0.018	0.001–0.004	0.94–2.89	0.08–0.19	1.02–3.08	0.97–1.08	1.69–1.81	21.95–34.12
REE Sulphide trend 3	<0.001–0.001	0.004–0.005	0.001–0.002	0.46–1.25	0.06–0.20	0.52–1.45	0.01–1.14	5.60–34.85	29.19–86.23

Massive sulphide samples (*n* = 5) are also categorised into three REE groups with REE sulphide trend 1 exhibits neither an Eu anomaly or a Ce anomaly (Table 2; Fig. 3). REE sulphide trend 2 characterised by a weakly positive Eu anomaly and no Ce anomaly. REE sulphide trend 3 show a strong positive Eu anomaly and no to weakly negative Ce anomaly.

### Sr-Nd-Pb isotope systematics.

**Fig. 4:**
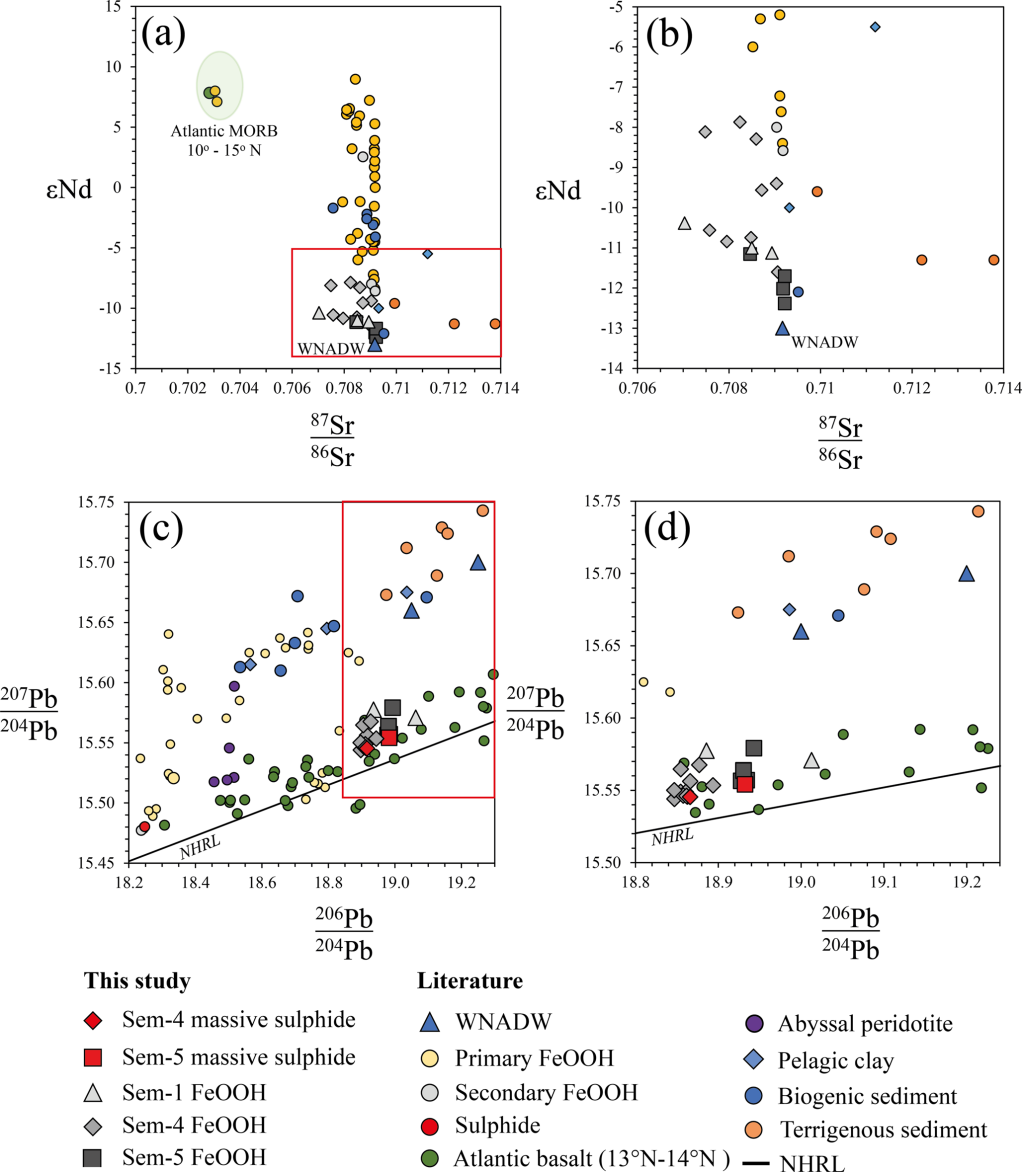
Summary of Pb-Nd-Sr isotopic data of the study. The data is compared with marine sediment (terrigenous, pelagic clay and biogenic) collected from Ben Othman et al. (1989), Western North Atlantic Deep Water (WNADW) collected from Abouchami et al. (1999), basalt derived from 13°N-14°N at the MAR after Wilson et al. 2013 and ultramafic rock after Burton et al. (2012). Primary FeOOH obtained after Dekov et al. (2010), Yang et al. (2015) and Ta et al. (2021) with secondary FeOOH and sulphide after Li et al. (2024). The NHRL is the Northern Hemisphere Regression Line of Hart (1984). Additional data for Nd-Sr ratios includes Atlantic MORB obtained between 10^o^N and 15^o^N after Agranier et al. (2005). Panels **(b)**, and **(d)** are magnified views of the red‑boxed regions in **(a)** and **(c)** respectively

Strontium isotopic ratios of FeOOH deposits range from 0.70703 to 0.70922 (Fig. 4a). While samples from Semenov-5 typically exhibit ^87^Sr/^86^Sr ratios indistinguishable from seawater, only a minority of samples (33%) at Semenov-4 exhibit present day seawater/detrital sediment. Nd isotopic ratios vary from εNd = −12.39 to −5.02, resembling Western North Atlantic Deep Water (WNADW; Fig. 4a; Abouchami et al. 1999). Combined Sr- εNd plots on Fig. 4b show FeOOH samples of Semenov plotting towards seawater.

Lead isotopic ratios of FeOOH and massive sulphide samples are comparable to those found along the Northern Hemisphere Regression Line (NHRL; Hart 1984) and basalt samples measured at the 13°30′N OCC by Wilson et al. (2013). In each Semenov area, the Pb isotopic compositions of FeOOH samples plot along mixing lines that extend from the local massive sulphide signature toward WNADW; this trend is most pronounced at Semenov-5 (Fig. 4b and c). The exceptions to this trend are two samples from Semenov-1, of which one lies close to the FeOOH and massive sulphide samples at Semenov-4, while the other exhibits increased radiogenic Pb, closer to that of basalt derived from the Semenov OCC. Full details of isotopic data are found in ESM2.5.

## Discussion

### Mechanisms of FeOOH formation at seafloor massive sulphide deposits

FeOOH formation at SMS systems occurs through either primary precipitation from hydrothermal fluids or via secondary oxidation of massive sulphide minerals (Alt 1988; Hekinian et al. 1993). At Semenov, both primary and secondary FeOOH are present, with varying textural morphologies, mineralogy, chemical composition and isotopic ratios.

Type-I chimney FeOOH deposits from Semenov-1 are likely coated with nontronite in the fluid conduit structure; nontronite typically precipitates from hydrothermal fluids at low temperatures (~ 30^o^C), overlapping with the temperature at which hydrothermal fluid can precipitate primary FeOOH and may represent a cooling trend from primary FeOOH to nontronite (≤ 100^o^C; Hekinian et al. 1993; Dekov et al. 2007; Gini et al. 2024). Type-I chimney deposits also exhibit alternating layers of FeOOH and Mn-oxide; these minerals can precipitate from low temperature hydrothermal fluid and the layering could represent changes in fluid composition (i.e., Mn-rich to Fe-rich), pH, Eh or temperature fluctuations (Roy 1992; Binns et al. 1993; Hekinian et al. 1993; Hein et al. 2008). Type-I chimney deposits are also depleted in base/trace metals (< 0.4 wt% Cu + Ni + Zn). Combined with the characteristics of nontronite precipitation, alternating FeOOH and Mn-oxide layers, chimney-like morphologies and low base/trace metal contents, the Semenov-1 FeOOH deposits share characteristics similar to primary FeOOH deposits reported by Hekinian et al. (1993), which form chimney morphologies and mounds. This suggests that Type-I chimney deposits (*n* = 5) are pre-dominantly primary FeOOH precipitates formed from low temperature Fe and Mn-rich hydrothermal fluids venting under fluctuating reducing conditions during the waning stage and low (< 100 ^o^C) temperature (Hekinian et al. 1993; Alt 1988; Gini et al. 2024). The trace amounts of disseminated pyrite within Type-I chimney deposits indicate episodes of high temperature (> 240 ^o^C), H_2_S-rich, hydrothermal fluid (Koski et al. 1994). The FeOOH associated with relict pyrite is likely to be secondary FeOOH, thus while Type-I chimney morphologies are pre-dominantly primary precipitates, secondary FeOOH does occur together with primary FeOOH. Type-I layered deposits at Semenov-1 are similar to Type-I chimney deposits (i.e., low metal content, alternating FeOOH and Mn-oxide) and as such, are also interpreted as primary FeOOH.

In contrast, Type-II FeOOH chimney deposits (Semenov-4) and other morphologies including brecciated, Type-II layered, ochre, ocherous and massive samples acquired at Semenov typically have elevated Cu and Zn contents (> 0.4 wt%), with FeOOH forming pseudomorphs after primary sulphide textures such as subhedral or colloform pyrite. Relict pyrite, akageneite (a pyrrhotite weathering product) and/or atacamite (indicative of Cu-sulphide weathering; Nambu 1968; Hannington 1993) are observed in at least one or more of these textural morphologies and indicate high temperature (> 240 ^o^C) hydrothermal fluid capable of producing pyrite, Cu-sulphides and pyrrhotite (Koski et al. 1994).

Moreover, Pb isotope ratios of these FeOOH samples closely match those of co-located massive sulphides, so that FeOOH from Semenov-5 inherits the Pb signature of Semenov-5 sulphide, and similarly for those from Semenov-4. The exception of this includes 66_HY_05, which aligns with the Semenov-4 sulphide, suggesting a similar sulphide source to Semenov-4 and 71_DR_01 (Type-I chimney) whose radiogenic Pb indicates a distinct source. Taken together, the textural evidence (pseudomorphic textures and sulphide relics), mineralogy (akaganeite, atacamite, sulphide) and isotopic inheritance demonstrate that Type-II chimney, Type-II layered, brecciated, massive, ochre and ocherous deposits (84% of samples) are secondary FeOOH formed by sulphide oxidation, whereas the remaining Type-I chimney and Type-I layered deposits are predominately primary FeOOH precipitates.

**Fig. 5:**
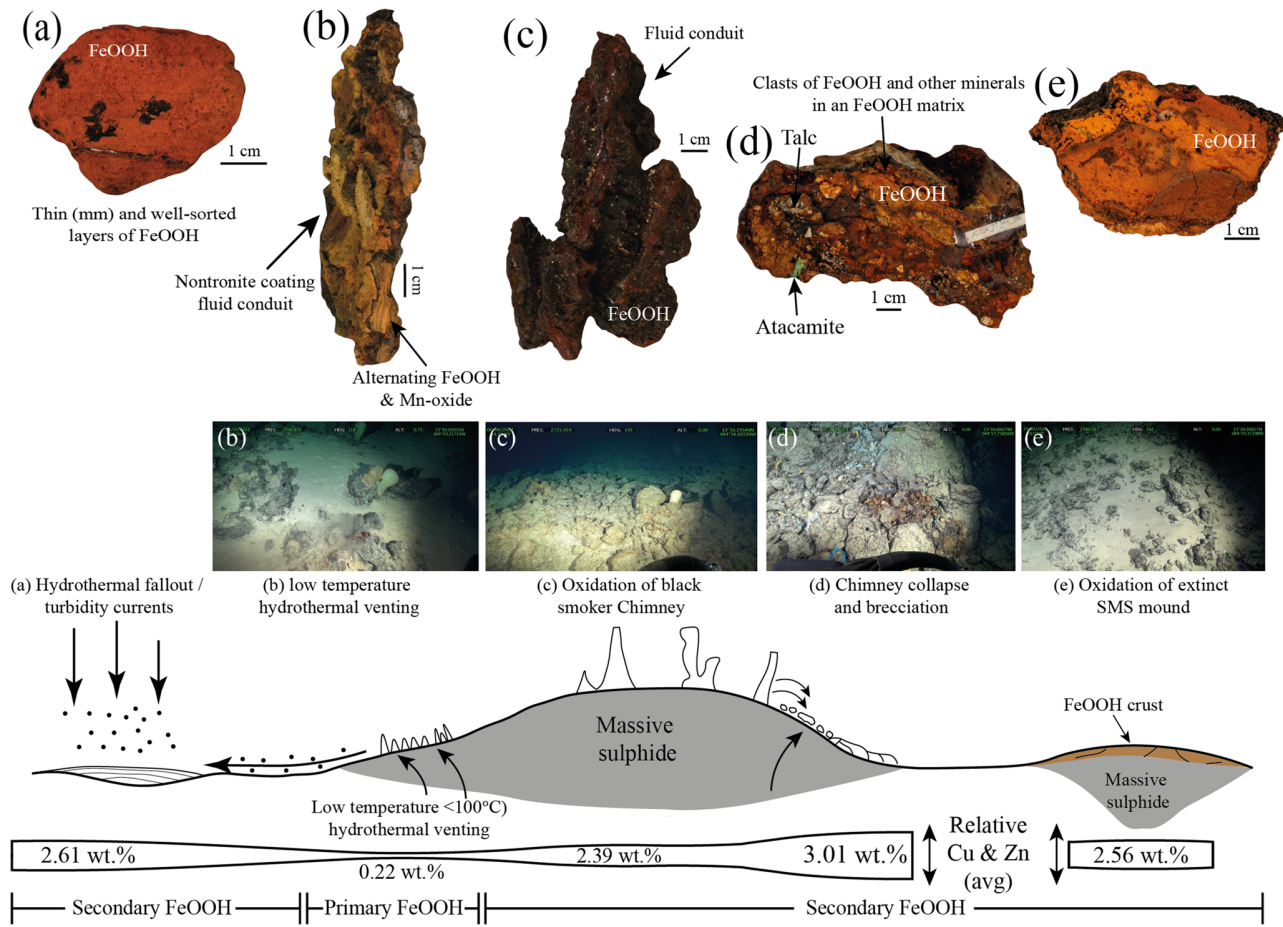
Formational mechanisms of FeOOH and their morphologies at Semenov with average of Cu and Zn contents. **(a)** Hydrothermal fallout precipitates as sulphides that are subsequently oxidised to secondary FeOOH. Alternatively, mass transport of metalliferous sediment and lithification may also result in the formation of ochre (e.g., 28_DR_18). Cu and Zn content is the average of type-I and type-II ochre morphologies (*n* = 6). **(b)** Low-temperature hydrothermal venting precipitates primary FeOOH and relict pyrite which are oxidised to secondary FeOOH (e.g., 71_DR_01). Cu and Zn content is the average of type-I chimney and type-I layered morphologies (*n* = 6). **(c)** Oxidation of black smoker hydrothermal chimneys to secondary FeOOH (e.g., 102_HY_04). Cu and Zn content is the average of type-II chimney and type-II layered morphologies (*n* = 10). **(d)** Brecciation of chimney material that may be followed by diffuse hydrothermal venting that precipitates various minerals such as barite and talc result in brecciated secondary FeOOH like those found in Semenov-2 (e.g., 77_HY_03). Brecciated samples may also form via mass wasting of sulphide material. Cu and Zn content is the average of type-I and type-II brecciated morphologies (*n* = 4). **(e)** Ambient seawater ingress into hydrothermal mound result in the oxidation of sulphide, forming secondary FeOOH. This mode of formation can produce massive, ocherous and ochre products (e.g., 66_HY_05). Cu and Zn content is the average of massive and ocherous morphologies (*n* = 11)

Having shown that most of the FeOOH morphological types are pre-dominantly secondary, it is possible that they also preserve some of the characteristics of their sulphide protoliths (Fig. 5). For example, based on their textural morphology and petrography, the protolith of a brecciated FeOOH samples are likely similar to brecciated massive sulphide, often found within the upper part of the SMS mound (Fig. 5 d; Humphris et al. 1996). Type-II chimney morphologies indicate focussed areas of fluid upwelling and are similar to black smoker chimneys located at the summit or close to the summit of SMS mounds (Fig. 5c; Haymon 1983; Hekinian et al. 1993; Knott et al. 1998). Type-II layered morphologies could be the result of the oxidation of black smoker fragments where there are zonations of mineral phases (Haymon 1983). The rhythmic layering of Type-II ochre indicates low energy levels and can be interpreted to form as plume fallout (Lilley et al. 1995; Gartman et al. 2024), or as products of gravity flow (Fig. 5a; Dutrieux et al. 2023). While Type-I ochre does not exhibit lamination, we suggest that as they are also granular with similar mineralogy, that Type-I ochre forms under similar processes to Type-II ochre. Ocherous and massive textural morphologies lack discernible features that can be directly linked to a specific sulphide protolith. While their exact origins are uncertain, ocherous and massive textural morphologies are nonetheless pre-dominantly secondary FeOOH.

In summary, secondary FeOOH formation mechanisms are dominant at the Semenov hydrothermal field. Both, primary and secondary FeOOH formation mechanisms produce FeOOH with varying textural morphologies with primary FeOOH identified by layering of FeOOH and Mn-oxide, low base metal contents (< 0.4 wt% Cu + Zn + Co) and the likely presence of nontronite. Secondary FeOOH are identified by high base metal content (> 0.4 wt% Cu + Zn + Co), Pb isotopic ratios inherited by massive sulphide, the presence of relict sulphide minerals (i.e., pyrite and chalcopyrite), sulphide weathering products (i.e., atacamite and akageneite) and FeOOH forming as pseudomorphs of sulphide minerals. The textural morphologies of secondary FeOOH can be used to infer their sulphide protolith and formation mechanisms (i.e., chimney-like secondary FeOOH are indicative of black smoker protolith). It is known that massive sulphides typically have varying metal contents depending on their texture (e.g., colloform vs. massive), which in turn relates to where they formed in the SMS mound (Fouquet et al. 1996; Martin et al. 2022). For example, black smoker chimneys that form at the summit of hydrothermal mounds are typically high in Zn and/or Cu (> 10 wt%) relative to massive sulphide found in other parts of the mound such as brecciated sulphide that has been modified within a mound (Fouquet et al. 1996; Hannington et al. 1998a). Thus, it is possible that secondary FeOOH inherit this variation of metal content based on textural morphology, similar to that of massive sulphide.

### Preservation of metal content in secondary FeOOH deposits

**Fig. 6:**
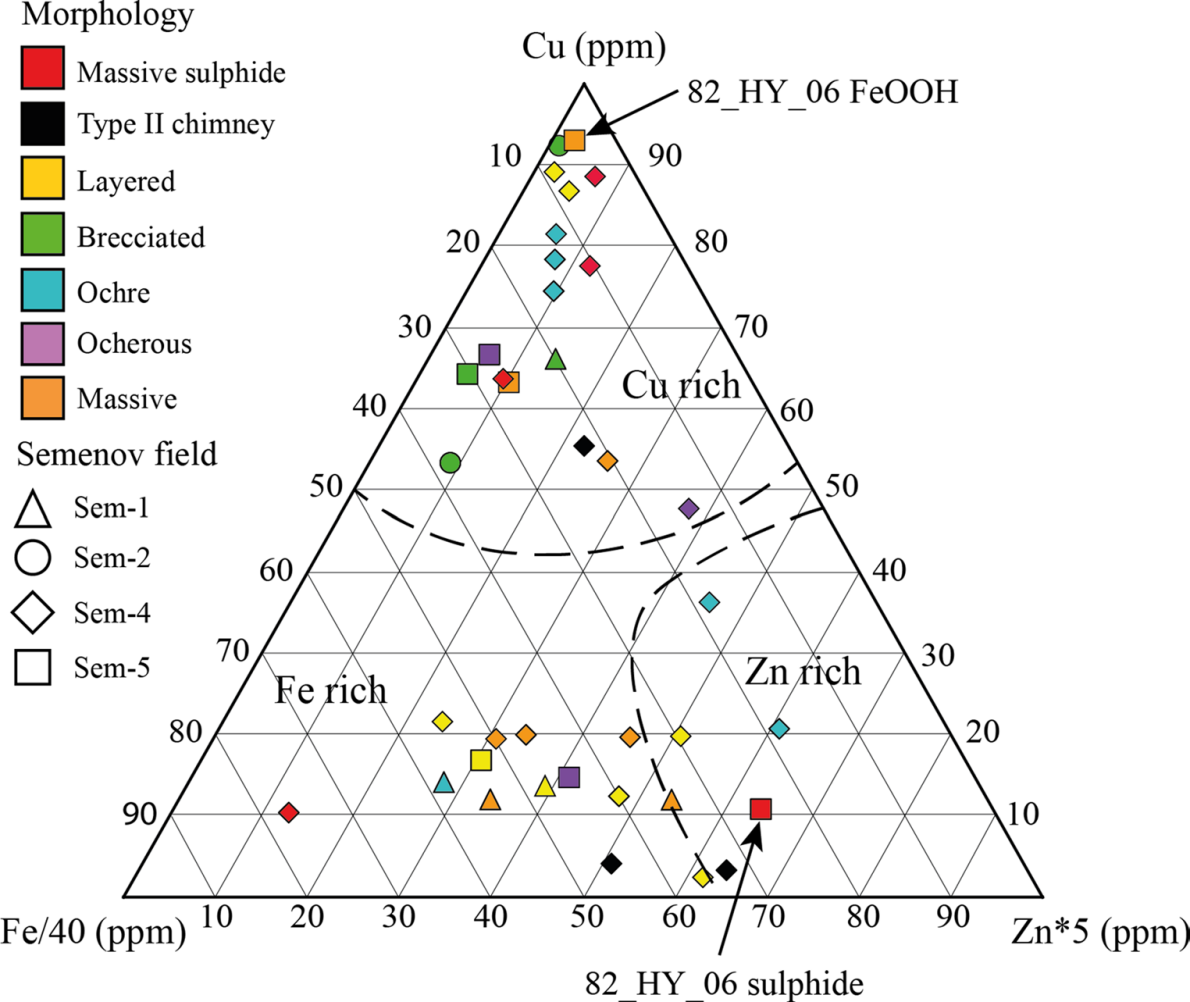
Ternary diagram of Cu, Zn*5 and Fe/40 illustrating secondary FeOOH of the study. The samples group into Fe rich (bottom left), Zn rich (bottom right) and Cu rich (top) regardless of morphology and Semenov area. Massive sulphide occupies each of these areas. 82_HY_06_a_ (FeOOH) and 82_HY_06_b_ (sulphide) are labelled

**Fig. 7:**
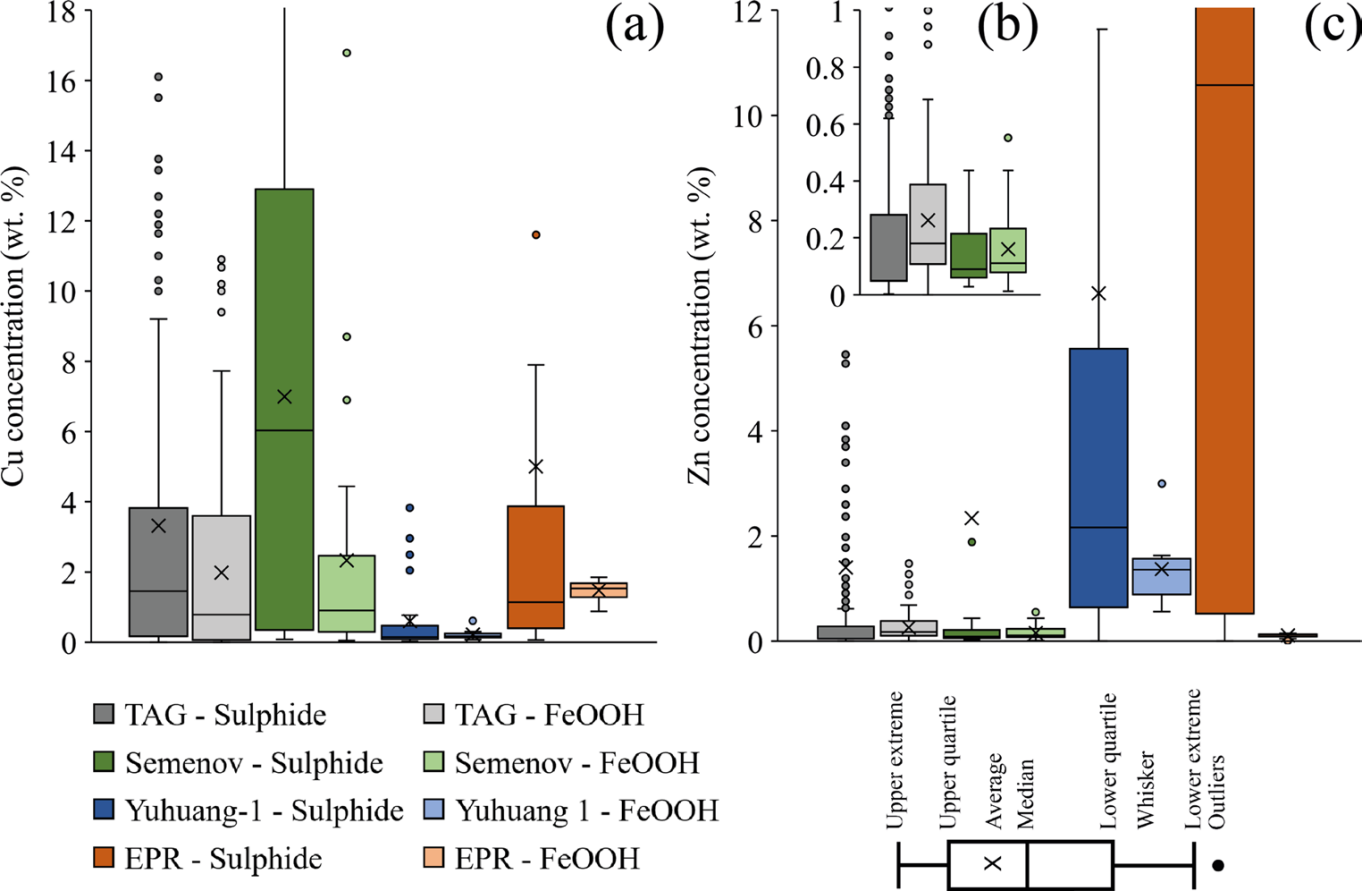
Average massive sulphide concentration and average FeOOH composition of Cu **(a)** and Zn **(b)** in box-whisker plots to visualise deviation and median of data. **(c)** Box-whisker plot displaying solely TAG and Semenov sulphide and FeOOH to better visualise Zn variation. TAG sulphide data (*n* = 270) obtained from Fouquet et al. (1998; *n* = 72), Hannington et al. (1998a; *n* = 67), Miller (1998; *n* = 23), Pelleter et al. (2024; *n* = 66) and Murton et al. (2019; *n* = 42) with FeOOH data (*n* = 210) obtained from Herzig et al. (1991; *n* = 4), Hannington et al. (1998b; *n* = 2), Petersen (2000; *n* = 163), Murton et al. (2019; *n* = 7) and Pelleter et al. (2024; *n* = 34). Semenov massive sulphide data (*n* = 37) obtained from this study (*n* = 5), Firstova et al. (2022; *n* = 18), Melekestseva et al. (2014; *n* = 2), Melekestseva et al. (2017b; *n* = 7) and Melekestseva et al. (2018; *n* = 5), with secondary FeOOH (*n* = 31) obtained from this study. Yuhuang-1 massive sulphide (*n* = 32) obtained from Liao et al. (2018; *n* = 18), Yu et al. (2021; *n* = 2) and Hu et al. (2022; n=12) with FeOOH (*n* = 9) obtained from Hu et al. (2022). EPR massive sulphide (*n* = 60) data obtained from Fouquet et al. (1996; *n* = 44) and Zeng et al. (2010; *n* = 16) with FeOOH data (*n* = 17) from Zeng et al. (2008)

Textural morphologies of sulphide often correspond to distinct metal enrichments, for example, black smoker chimneys are typically enriched in Cu and Zn relative to massive sulphide within mound (Fouquet et al. 1998; Knott et al. 1998). In contrast, secondary FeOOH deposits at Semenov form three chemical distinct groups: (i) Fe-rich, (ii) Cu-rich and (iii) Zn-rich, without any association to their textural morphology (Fig. 6). This decoupling suggests that the secondary FeOOH metal content does not reflect the metal content of the sulphide protolith, but is instead overprinted by post-formational processes that redistribute Cu and Zn within the crust. In order to prove so, a direct comparison must be made between the sulphides and weathering products. For this, 82_HY_06 is the only sample for which a direct comparison can be made between secondary FeOOH and the massive sulphide protolith. The massive sulphide (82_HY_06_b_) contains 0.37 wt% Cu and 0.44 wt% Zn, with the texturally massive FeOOH (82_HY_06_a_) crust exhibiting considerable enrichment in Cu (16.79 wt%) and depletion in Zn (0.09 wt%; Fig. 6), compared to the sulphide protolith. In this singular example, the metal content of secondary FeOOH does not reflect the metal content of the associated massive sulphide. The enrichment in Cu within the FeOOH crust can be partially attributed to the presence of atacamite that makes up approximately 10 wt% by volume. Atacamite veins are present in eight samples of secondary FeOOH samples from Semenov and where they are found, they increase the bulk Cu content in the sample (average and standard deviation of 6.27 ± 5.30 wt%, *n* = 8) relative to secondary FeOOH samples with no atacamite (1.05 ± 1.20 wt% Cu, *n* = 23). Atacamite forms through the oxidation of Cu-sulphides and can be mobilised some distance from the sulphide protolith to form as atacamite veins (Hannington 1993; Dekov et al. 2011) as observed in the samples of this study (Fig. 2b). This observation indicates that secondary FeOOH do not necessarily reflect the metal content of the sulphide protolith as previous observations suggest (Herzig et al. 1991; Hannington et al. 1991; Hu et al. 2022); instead, we propose that post-formational modification alters the composition of secondary FeOOH deposits over time. Due to the adsorptive qualities of FeOOH (Cornell and Schwertmann 2003), FeOOH may act as a gossan by trapping metals released during the oxidation of underlying sulphide (Hu et al. 2022). For instance, atacamite veins are found to cross-cut the FeOOH crust in 82_HY_06_a_ (Fig. 2b), which indicates that metal mobilisation and reprecipitation can occur throughout the FeOOH crust, rather than confined to a single boundary. If secondary FeOOH acts as a gossan, the metal content of secondary FeOOH cannot be used to determine the metal content of the sulphide protolith, but may be used to infer the metal content of the underlying ore body. To test this, secondary FeOOH and massive sulphide reported from elsewhere have been compared with the results from this study (Fig. 7).

This study identifies that Semenov has a higher average Cu content (average of 2.40 ± 3.51 wt%, *n* = 31) in secondary FeOOH relative to other reported studies (Fig. 7). Although the Cu and Zn distributions include a few high-concentration outliers, we have chosen to report average values to capture the full range of metal enrichment within secondary FeOOH. These outliers are geologically meaningful because they may represent secondary FeOOH formed via weathering of high-grade sulphide or as effective gossan, capturing metals mobilised from the oxidation of underlying sulphide. We also find that the Cu content of secondary FeOOH correlates with the Cu content of massive sulphide; indicating that the average Cu grade of secondary FeOOH can reflect the Cu grade of massive sulphide. However, this correlation is not observed in Zn. Furthermore, relative to the average massive sulphide composition, secondary FeOOH are lower by up to 71% Cu and 99% Zn (Fig. 7).

The observed decreased capacity of FeOOH to adsorb Zn relative to Cu may be a function of the pH of acidic pore fluids produced during sulphide oxidation, which are estimated to range from 3.6 to 5.5 (Hannington 1993). Zinc adsorption onto FeOOH is more efficient at pH levels above 5.5, whereas Cu adsorption is effective above pH 4.5 (Benjamin and Leckie 1981; Kooner 1993). Given that goethite can precipitate at pH as low as 4.0 (Glasby and Schulz 1999), Zn may not adsorb effectively onto FeOOH in environments where the pH remains below 5.5. This pH dependent adsorption behaviour may explain the increased loss of Zn within secondary FeOOH relative to massive sulphide as compared with Cu (Fig. 7). Furthermore, Zn is more soluble than Cu and will have a higher probability of being mobilised into the overlying seawater (Rose 1976; Hem 1985; Haynes 2016).

The findings of this study support the premise that secondary FeOOH acts as a gossan to trap metals released as a result of sulphide oxidation. As the Cu content of secondary FeOOH correlates with Cu content of massive sulphide, secondary FeOOH deposits may be used as an exploration tool for identifying underlying Cu-rich massive sulphide. For example, during initial investigation, ROVs can collect grab samples of FeOOH deposits across the SMS deposit. These samples can be rapidly screened ship-board using portable X-ray fluorescence (pXRF; Murton 2022) to identify areas with anomalously high Cu (or Zn). By mapping Cu “on the fly” during site investigation, teams can focus subsequent geochemical or geophysical surveys (e.g., seismic surveys; Murton et al. 2019) on the most prospective sectors. By combining geochemical and geophysical models, this can create drilling targets or coring sites where secondary FeOOH deposits are most prospective with geophysical surveys guiding towards the larger sulphide bodies. Additionally, this study has shown that secondary FeOOH can contain appreciable quantities of Cu at on average 2.40 wt% (*n* = 31) and could be an additional resource at SMS deposits. This is more important when considering that metalliferous sediment (that has a component of secondary FeOOH; Dutrieux et al. 2023) at Semenov-4 is extensive and can reach > 1 m in thickness (Murton 2022), with one instance at TAG reporting metalliferous sediment > 10 m thick (Murton et al. 2019). Despite this, secondary FeOOH deposits are still lower in both Cu and Zn compared with massive sulphide by up to 71% Cu and 99% Zn indicating that the weathering of massive sulphide results in a loss of both Cu and Zn (Fig. 7).

### FeOOH-seawater interactions: long term effects on Cu and Zn content

**Fig. 8:**
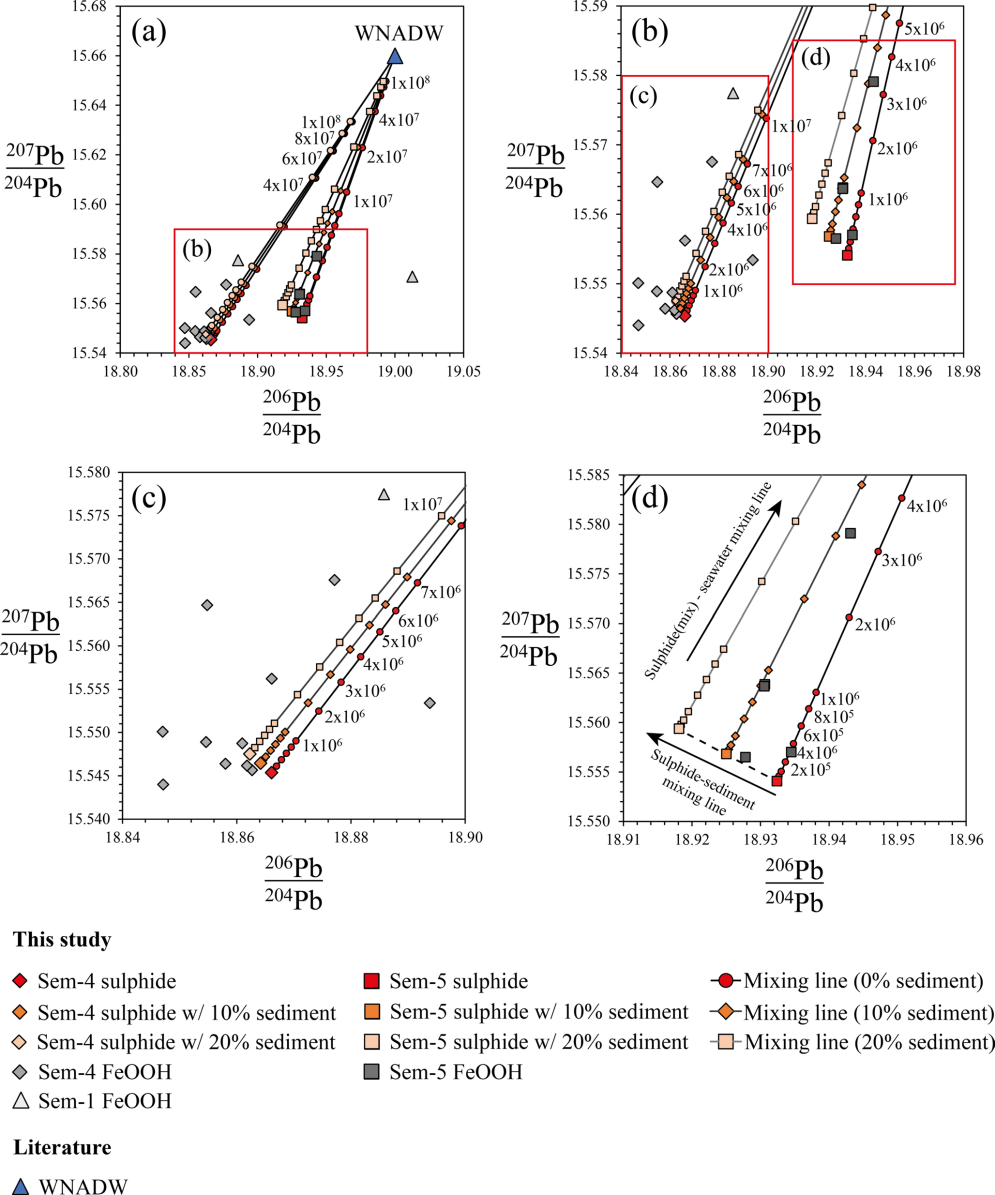
Lead isotope compositions of massive sulphide and FeOOH samples from this study, shown alongside a Western North Atlantic Deep Water (WNADW) reference at 3.57 ppt (Abouchami et al. 1999). Sulphide samples were modelled with 0%, 10%, and 20% sediment contribution to illustrate how sediment contribution impact mixing lines with seawater, and mixing curves illustrate the effect of increasing seawater input (higher F/R ratio) on Pb isotope signatures in FeOOH. **(a)** full isotope data showing Semenov-4 and Semenov-5 sulphide-seawater mixing lines. **(b)** magnified view of red box in panel (a) to highlight lower F/R values. **(c)** detailed view of Semenov-4 from region shown in panel (b). **(d)** detailed view of Semenov-5 from region shown in panel (b) with labelled theoretical mixing lines of (1) sulphide-sediment mixing lines (Eqs. 3) and (2) sulphide-seawater mixing lines (Eq. 4)

As secondary FeOOH accumulate on SMS deposits, they remain continually subjected to seawater flow. The interaction of seawater with FeOOH deposits is evidence by negative Ce anomalies (Debaar et al. 1985; Mills and Elderfield 1995), enrichment of seawater-derived elements (e.g., Ni and Sc; Koschinsky and Hein 2003; Hein et al. 2017) in FeOOH relative to massive sulphide and ^87^Sr/^86^Sr and εNd isotopic ratios trending towards seawater ratios (Fig. 4). Seawater interaction with FeOOH may also be observed in Pb isotope ratios, where FeOOH ratios lie close to or on mixing lines between massive sulphide and seawater (Fig. 8).

It is currently unclear how seawater interaction may affect the metal content of Cu and Zn, whether ions dissolved in seawater (e.g., Na^+^, Mg^2+^, Ca^2+^) compete with sorbed metals on FeOOH, resulting in their depletion or if the content of Cu and Zn remain stable (Balistrieri and Murray 1982; Calmano et al. 1988). Here, we quantify the degree of seawater exposure using a fluid-to-rock (F/R), defined as the cumulative mass of seawater that has percolated through FeOOH. Practically, as seawater is continuously replacing the interstitial fluid in the FeOOH pore network, thereby incrementally increasing the F/R ratio and providing new competing dissolved ions that compete with sorbed metals on the FeOOH surface. We derive F/R values by applying Pb isotope mixing models between massive sulphide and seawater endmembers (Fig. 8; Abouchami et al. 1999; Bridgestock et al. 2018). However, pelagic sediment contamination within the FeOOH deposits is geochemically evident by similar REE trends as marine sediment (Fig. 3) and a positive correlation exists between ΣREE and both Al/(Al + Fe + Mn) and Ca, suggesting that FeOOH samples could contain some pelagic sediment and hence impact the Pb isotopic ratios of the FeOOH. Pelagic sediment typically have lower ^206^Pb^/204^Pb and higher ^207^Pb/^204^Pb and ^208^Pb/^204^Pb ratios (Fig. 4) and should be considered when looking at Pb isotope systematics of FeOOH samples. Pelagic sediments are primarily composed of biogenic material (i.e. carbonates) and clay-sized particles, and will, over longer term, settle on the seafloor and could get incorporated into the porous FeOOH minerals. Sediment studies at Semenov identify foraminiferal ooze common within the sediment, supporting the interpretation of pelagic sediment at Semenov (Rusakov et al. 2013). Using Eq. 1 and Eq. 2 (ESM2.6) an average of 4% (*n* = 17) sediment might be contained in our bulk FeOOH samples, and this was considered in when calculating the sulphide-seawater mixing (Eq. 3 and Eq. 4; Fig. 8; ESM2.7).

**Fig. 9:**
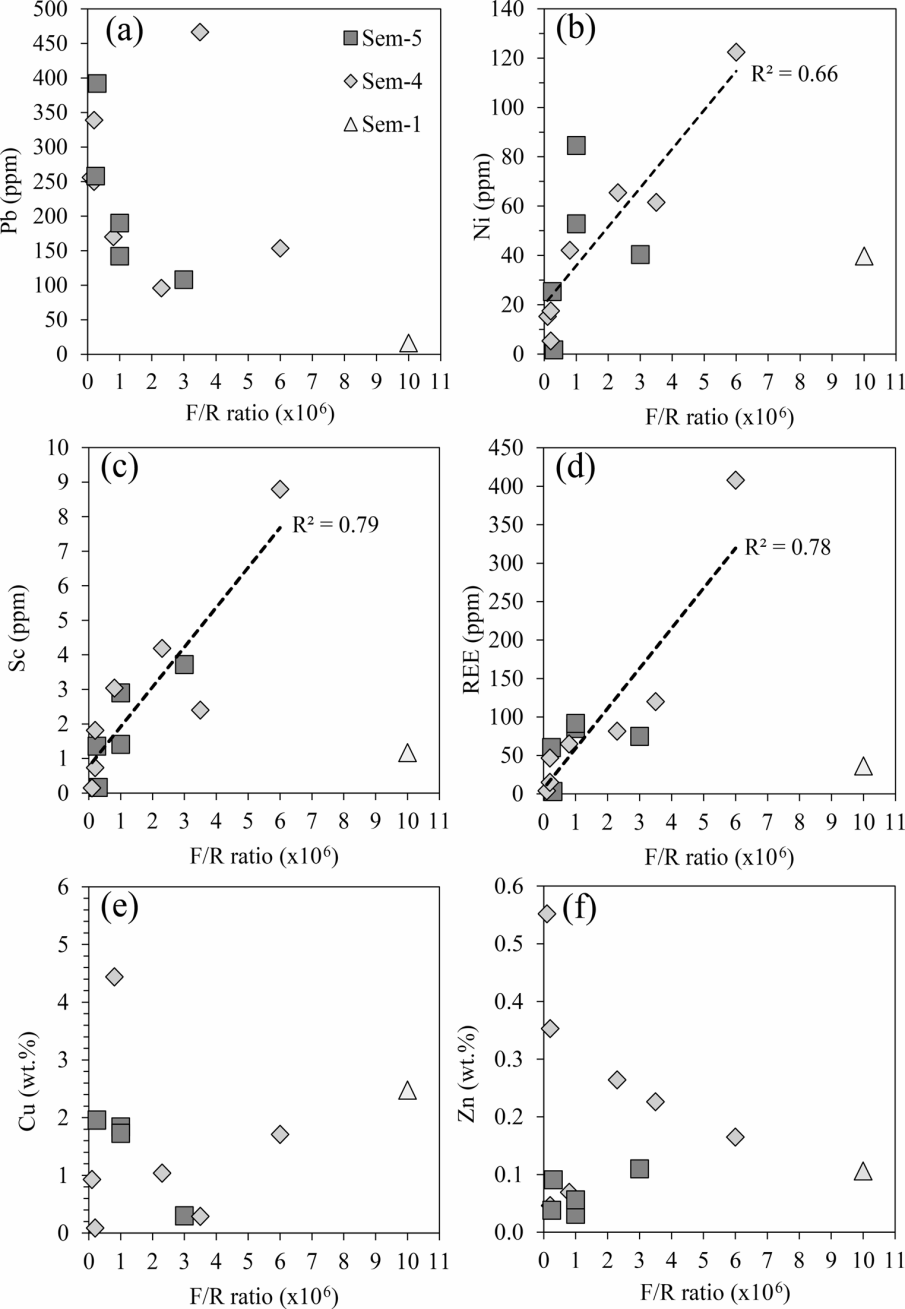
Bivariate plots comparing F/R ratios derived from Pb isotopic ratios against **(a)** Pb, **(b)** Ni, **(c)** Sc, **(d)** REE, **(e)** Cu and **(f)** Zn. Note, an outlier is not shown for (e) Cu at 16.79 wt% Cu and 3.0 × 10^5^ F/R ratio. Correlations visible on (b), (c) and (d) do not consider Sem-1 due to its low Pb content at 16.5 ppm making it highly susceptible to seawater influence resulting in an inflated F/R ratio

The calculated F/R ratios form positive correlations with seawater derived elements including Sc (R^2^ = 0.79), REE (0.78) and Ni (0.66; Fig. 9; Koschinsky and Hein 2003; Hein et al. 2017). Consequently, the F/R ratio can serve as a proxy for assessing the interaction between seawater and FeOOH. Crucially, despite extensive F/R ratios observed in this study, Cu concentrations in secondary FeOOH remain stables at ~ 2 wt% (Fig. 9e). In contrast Zn behaviour is more variable and display no obvious trends except that not all Zn is depleted (Fig. 9f).

In summary, increasing seawater exposure does not result in the depletion of Cu, which remains constant at approximately 2 wt%, hence Cu is not released/desorbed from FeOOH and lost to seawater during FeOOH-seawater interactions. These findings suggest that secondary FeOOH are capable of retaining the adsorbed Cu and possibly Zn over time. This behaviour might be especially important at extinct, older, buried off-axis SMS deposits that have undergone increased oxidation and exhibit greater quantities of secondary FeOOH.

### Implications for terrestrial analogues at Volcanogenic Massive Sulphide (VMS) deposits

Ochre and gossanite deposits associated with VMS deposits, such as those from Skouriotissa in Cyprus (Constantinou 1972) and Molodezhnoye in the Urals (Maslennikov et al. 2012), are regarded as ancient analogues of submarine sulphide weathering products (secondary FeOOH). By comparing modern secondary FeOOH from SMS deposits with terrestrial deposits, we can evaluate the genetic links and secondary alteration processes that affect metal content during seafloor oxidation and subsequent obduction. Although the Troodos ochres are hosted by mafic rocks and the Urals gossanites by ultramafic rocks, compared to the mixture of ultramafic and mafic influences in Semenov, all undergo seawater-driven oxidation.

In terms of morphology and mineralogy, secondary FeOOH deposits at Semenov, exposed to seafloor conditions for up to ~ 130 ka (Kuznetsov et al. 2011), display distinctive chimney, massive, ocherous, layered and brecciated textures. In contrast, the ochre deposits at Cyprus, exposed to continuous seafloor conditions for 5 million years (Ravizza et al. 2001), are predominantly goethite and exhibit well-bedded, granular, graded textures (Constantinou 1972), suggesting that in Cyprus the prolonged oxidation and gravity-driven reworking (e.g., mass-wasting) progressively erased the original textures that currently is still at Semenov. Gossanite deposits from the Urals are composed of oxidised sulphide clasts with varying components of hematised carbonate and/or hyaloclastic material replaced by silica, chlorite and hematite. Gossanite is formed by the oxidation of clastic sulphide in ore turbidites intermixed with sediment that has been later subject to metamorphism (150^o^−300^o^C; Maslennikov et al. 2012; Vikentyev et al. 2017). Although Urals gossanite experience sedimentary dilution and metamorphism, it retains primary oxidative weathering processes and can be used evaluate how alteration can modify the metal content of oxidative products.

**Fig. 10:**
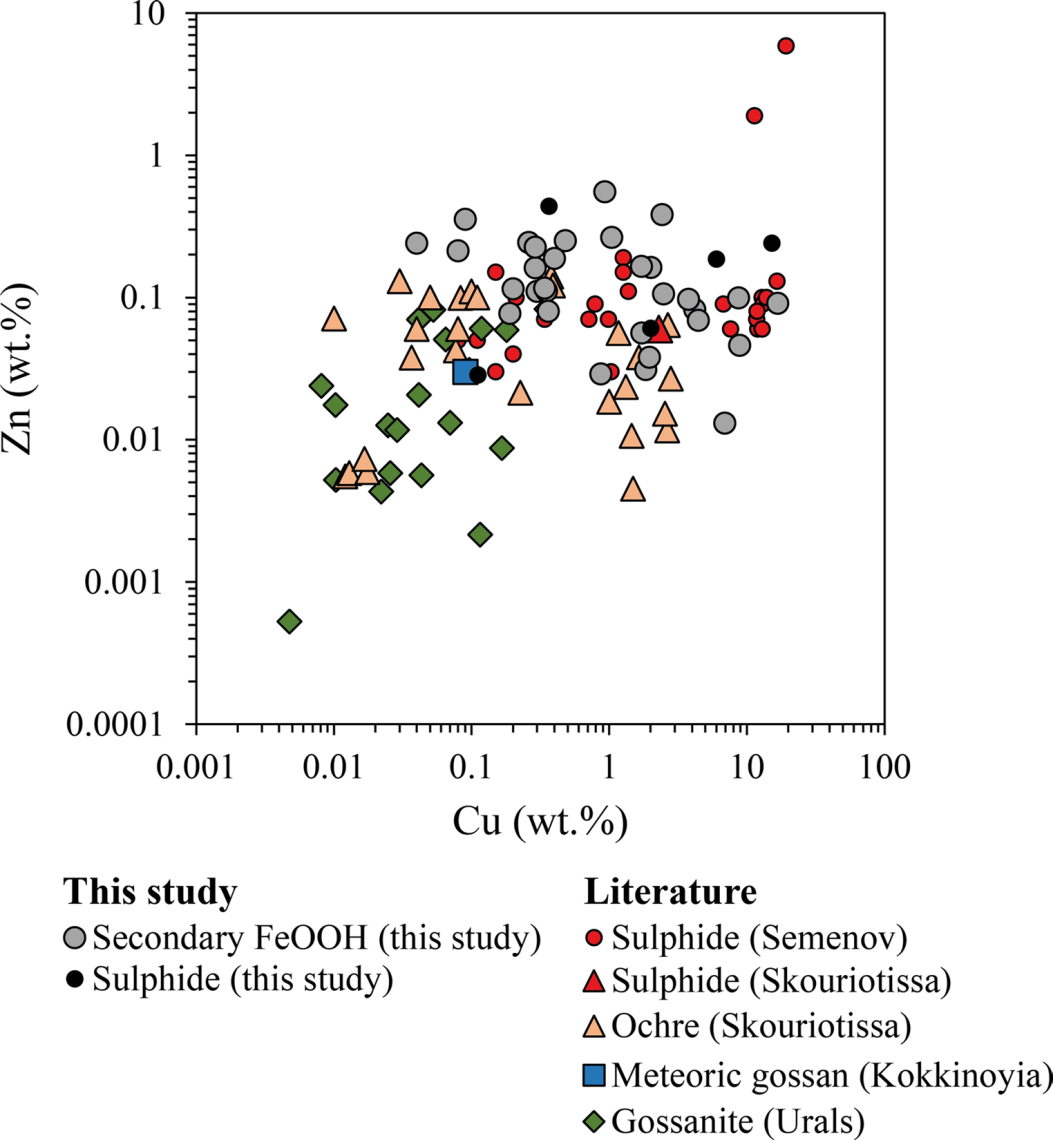
Bivariate plot comparing the content of Cu and Zn from secondary FeOOH at Semenov to ochre obtained at Skouriotissa, meteoric gossan material obtained at Kokkinoyia and gossanite material obtained from various VMS deposits in the Urals. Data for ochre obtained after Constantinou (1972; *n* = 17); Robertson and Fleet (1976; *n* = 3); Herzig et al. (1991; *n* = 6) and Wells (1998; *n* = 4), with meteoric gossan after Herzig et al. (1991; *n* = 1), gossanite after Maslennikov et al. (2012; *n* = 20), sulphide at Skouriotissa after Hannington et al. 1998b and sulphide at Semenov (*n* = 37) obtained from this study (*n* = 5), Firstova et al. (2022; *n* = 18), Melekestseva et al. (2014; *n* = 2), Melekestseva et al. (2017b; *n* = 7) and Melekestseva et al. (2018; *n* = 5)

Our geochemical analysis indicate that secondary FeOOH at Semenov exhibits higher average Cu and Zn concentrations at 2.55 wt% (*n* = 31), compared to Skouriotissa ochre at 0.81 wt% (*n* = 27) and Urals gossanite at 0.10 wt% (*n* = 20; Fig. 10). The notably lower metal content in the gossanite may result from sedimentary dilution or metamorphic processes that can induce dehydration reactions in minerals like goethite to hematite and can lead to potential leaching of Cu and Zn during fluid interactions (Jolly 1974; Goss 1987). The lower Cu and Zn concentration in Skouriotissa ochre relative to secondary FeOOH at Semenov may result from: (i) a metal poor sulphide protolith (Hannington et al. 1998b; Fig. 10); (ii) enhanced metal leaching by meteoric groundwater circulation (Lydon 1984); or (iii) dissolution of atacamite during obduction or meteoric alteration (Hannington 1993). The data distribution (Fig. 10) suggest minimal meteoric alteration in most Skouriotissa samples, likely preserving their original composition. However, the absence of atacamite and reduced Cu and Zn levels, compared to meteoric gossan from Kokkinoyia, suggest some influence of meteoric groundwater. Consequently, while secondary FeOOH retains Cu and Zn under submarine conditions, subaerial exposure may lead to their remobilisation. This is supported by F/R ratios indicating that older, off-axis SMS deposits likely preserve these metals.

Comparing secondary FeOOH from SMS deposits with ochre/gossanite deposits from VMS systems demonstrates that seafloor FeOOH undergoes morphological alteration, losing its original textural morphology, likely due to continuous oxidation and gravity-driven flows. Despite these processes, the Cu and Zn content in secondary FeOOH may remain unaffected at the seafloor, indicating that older, off-axis SMS deposits likely also preserve their metal content. Furthermore, since ochre deposits, such as Skouriotissa, are constrained to overlying the sulphide orebody (Constantinou 1972), the detection of secondary FeOOH deposits at the seafloor such as those at Semenov can serve as effective exploration marker to identify concealed massive sulphide mineralisation.

## Summary and conclusions

At the Semenov hydrothermal field, primary FeOOH, characterised by low metal content, can be initially identified by its chimney-like textures, formed through alternating precipitation of FeOOH and Mn-oxide, accompanied by green smectite (nontronite) deposition within the fluid conduits. In contrast, secondary FeOOH constitutes the majority of FeOOH deposits at the Semenov hydrothermal field and displays a broader range of textural morphologies than previously reported at SMS deposits. Secondary FeOOH deposits inherit the textural morphology from the sulphide protolith (e.g., Type-II chimney and brecciated forms). We propose that secondary FeOOH can be chemically differentiated into distinct compositional groups of Fe-rich, Cu-rich and Zn-rich, resulting from the initially inherited metal content of the sulphide protolith and post-formational modification processes. For instance, atacamite veins may precipitate within FeOOH, as a result of the oxidation of underlying Cu-sulphides, enriching the secondary FeOOH crust in Cu. In this way, secondary FeOOH functions similarly to what has been shown in terrestrial gossan, i.e. acting as a trap for metals. Further to this, the average Cu-content of secondary FeOOH may provide an indication of the Cu-content of underlying sulphide and this can help to guide future exploration strategies to target areas of Cu-rich FeOOH crust that are likely identified by atacamite mineralisation. This may be especially useful in extinct SMS deposits where much of the sulphide has been oxidised to secondary FeOOH, resulting in limited sulphide exposure.

Secondary FeOOH at Semenov can retain appreciable quantities of Cu (average of 2.40 wt%) that could be considered as an additional resource at SMS deposits. Derived F/R ratios and comparison with ochre/gossanite deposits from VMS deposits indicate that secondary FeOOH are likely to retain appreciable quantities of Cu at the seafloor. This suggests that secondary FeOOH in off-axis SMS deposits could contain increased quantities of Cu. Furthermore, as these off-axis SMS deposits are older, they are likely to have been subject to greater oxidation, and thus, secondary FeOOH might be present in larger quantities and economic importance relative to younger SMS deposits close to the MOR, such as in Semenov. Overall, we suggest that secondary FeOOH at SMS deposits could represent both an additional resource and as valuable exploration guides in seafloor mineral exploration.

## Electronic supplementary material

Below is the link to the electronic supplementary material.

Supplementary Material 1 (PDF 9.65 MB)

Supplementary Material 2 (PDF 1.06 MB)

Supplementary Material 3 (XLSX 81.4 KB)

Supplementary Material 4 (DOCX 268 KB)

## Data Availability

The data used in this study are available in the electronic supplementary material and are also stored in the British Oceanographic Data Centre (BODC) database via 10.5285/2e0aa2c7-6331-62b9-e063-7086abc06891 after (Bishop et al. 2025a) and 10.5285/2e0a9d33-56f6-622d-e063-7086abc06e59 after (Bishop et al. 2025b).
