# Supplementary material for: Fe-oxyhydroxide deposits at Semenov hydrothermal field (13°30′N), Mid-Atlantic ridge: insights into formation, modification and resource potential
Source: Miner Depos. 2025 Aug 1;61(2):257–79. doi: 10.1007/s00126-025-01376-6 (PMC12858624; doi:10.1007/s00126-025-01376-6)

Fe-oxyhydroxide deposits at Semenov Hydrothermal Field (13°30′N), Mid-Atlantic Ridge: insights into formation, modification and resource potential

Authors - Christian Bishop[1], Anna Lichtschlag[2], Stephen Roberts1, Maxime Lesage[3] & Bramley J. Murton[2]

[1] School of Ocean and Earth Science, University of Southampton, Southampton, UK,

[2] National Oceanography Centre, Southampton, UK,

[3] Nedre Slottsgate 8 - 0157 Oslo, Norway

E-mail – csb1u21@soton.ac.uk & chris.star.bish@gmail.com

ORCiD - 0000-0003-0652-3008

Electronic supplementary materials 1 – Sample descriptions and sample summary data

Table S1.1 Summary data of samples used in the study. Depth refers to meters below sea level and the field refers to the hydrothermal field at Semenov. The coordinates (latitude and longitude) are presented in degrees and decimal minutes.

Sample	Morphology	Latitude	Longitude	Depth	Field
28_DR_03	Type II layered	13°30.263 N	44°53.497W	2878	4
28_DR_04	Type I ochre	13°30.263 N	44°53.497W	2878	4
28_DR_05	Type II chimney	13°30.263 N	44°53.497W	2878	4
28_DR_18	Type II ochre	13°30.263 N	44°53.497W	2878	4
39_HY_02	Type I layered	13°30.925 N	44°59.204 W	2602	1
66_HY_05	Type I brecciated	13°30.907 N	44°59.374 W	2589	1
71_DR_01	Type I chimney	13°30.856 N	44°59.344 W	2584	1
71_DR_02	Type I chimney	13°30.856 N	44°59.344 W	2584	1
71_DR_03	Type I chimney	13°30.856 N	44°59.344 W	2584	1
71_DR_04	Massive	13°30.856 N	44°59.344 W	2584	1
71_DR_07	Type I chimney	13°30.856 N	44°59.344 W	2584	1
71_DR_09	Type I chimney	13°30.856 N	44°59.344 W	2584	1
71_DR_12	Type I chimney	13°30.856 N	44°59.344 W	2584	1
77_HY_02	Type II brecciated	13°30.807 N	44°57.757 W	2429	2
77_HY_03	Type II brecciated	13°30.807 N	44°57.757 W	2429	2
82_HY_01	Type I brecciated	13°30.660 N	44°56.060 W	2222	5
82_HY_05	Ocherous	13°30.687 N	44°56.147 W	2230	5
82_HY_06$_a$ (FeOOH)	Massive	13°30.684 N	44°56.049 W	2230	5
82_HY_06$_b$ (sulphide)	Pyrite dominated massive sulphide	13°30.684 N	44°56.049 W	2230	5
82_HY_09	Type II layered	13°30.690 N	44°56.122 W	2206	5
82_HY_10	Massive	13°30.671 N	44°56.112 W	2206	5
82_HY_11	Ocherous	13°30.684 N	44°56.164 W	2211	5
86_HY_02	Colloform massive sulphide	13°30.491 N	44°53.089 W	2839	4
86_HY_04	Ocherous	13°30.430 N	44°53.031 W	2798	4
86_HY_07	Pyrite dominated massive sulphide	13°30.331 N	44°53.196 W	2871	4
86_HY_08	Type II layered	13°30.331 N	44°53.196 W	2872	4
86_HY_09	Type I ochre	13°30.335 N	44°53.208 W	2864	4
87_MC_01	Massive	13°30.851 N	44°59.453 W	2568	1
90_HY_04$_a$ (FeOOH)	Massive	13°30.637 N	44°53.106 W	2824	4
90_HY_04$_b$ (sulphide)	Barite rich massive sulphide	13°30.637 N	44°53.106 W	2824	4
90_HY_04$_c$ (sulphide)	Barite rich massive sulphide	13°30.637 N	44°53.106 W	2824	4
90_HY_05	Massive	13°30.597 N	44°53.157 W	2768	4
90_HY_06	Massive	13°30.535 N	44°53.248 W	2817	4
94_HY_01	Type I ochre	13°30.206 N	44°53.748 W	2887	4
94_HY_04	Type I ochre	13°30.219 N	44°53.821 W	2841	4
94_HY_05	Type II layered	13°30.237 N	44°53.848 W	2822	4
94_HY_06	Type II layered	13°30.336 N	44°53.695 W	2865	4
102_HY_03	Type II layered	13°30.279 N	44°53.920 W	2779	4
102_HY_04	Type II chimney	13°30.301 N	44°59.015 W	2740	4
102_HY_05	Type II chimney	13°30.293 N	44°54.033 W	2725	4
102_HY_06	Massive	13°30.285 N	44°59.059 W	2717	4
102_HY_07	Massive	13°30.285 N	44°59.070 W	2713	4

JC224_028_DR_03

Date / time (GMT)	Location	Start (latitude/ longitude)	End (latitude/ longitude)	Water depth (m)
19/03/22; 05:03	Sem 4	13˚30.263 N 44˚53.497W	13˚30.486 N 44˚53.285 W	2878

Description: Layered, soft, friable FeOOH with layers comprising of FeOOH material ranging in colour from brownish orange do dark orangish brown. Layers from from thick(~0.5 cm) to thin (<1 mm). There are rare veins (~1 mm thick) consisting of grey, submetallic in lustre hard mineral. Could be magnetite. Hole like structures in the sample may be a previous hydrothermal fluid conduit.

Morphology – Type II layered

Geological association – Anywhere along dredge line from Sem 4. HyBIS observations, topography and samples collected in dredge have allowed a rough geological map where it is likely that the dredge crossed both igneous rock and hydrothermal crust.

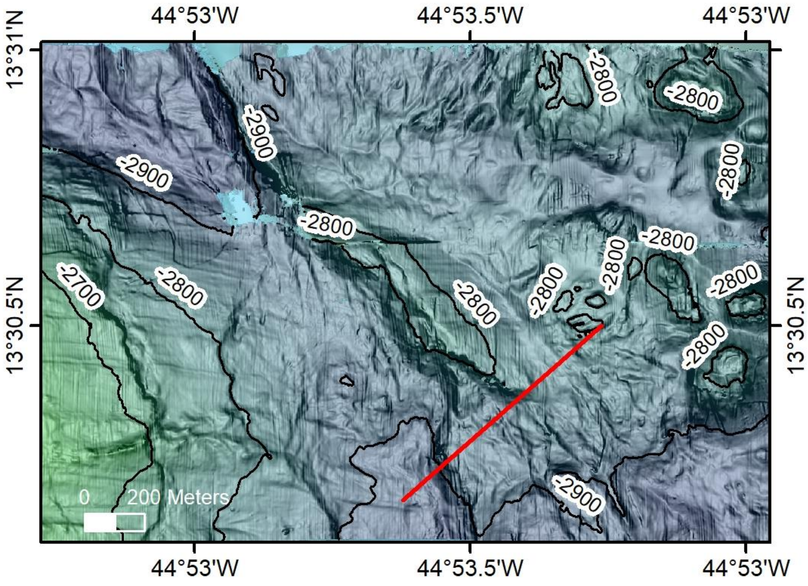

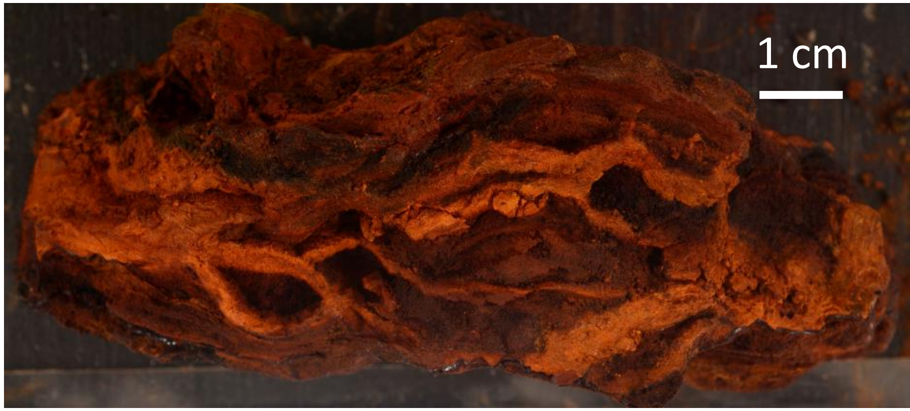

JC224_028_DR_04

Date / time (GMT)	Location	Start (latitude/ longitude)	End (latitude/ longitude)	Water depth (m)
19/03/22; 05:03	Sem 4	13˚30.263 N 44˚53.497W	13˚30.486 N 44˚53.285 W	2878

Description: Ochre sample comprising of clay-silt sized grains dominated by orange FeOOH material. Within samples contains <1mm vitreous platy minerals, likely barite with some larger (~1 mm) sized hard minerals that appear greenish clear in colour that could be quartz. Sample is soft and easily broken. Surface of sample has Fe-Mn oxide precipitate.

Morphology – Type I ochre

Geological association – Anywhere along dredge line from Sem 4.

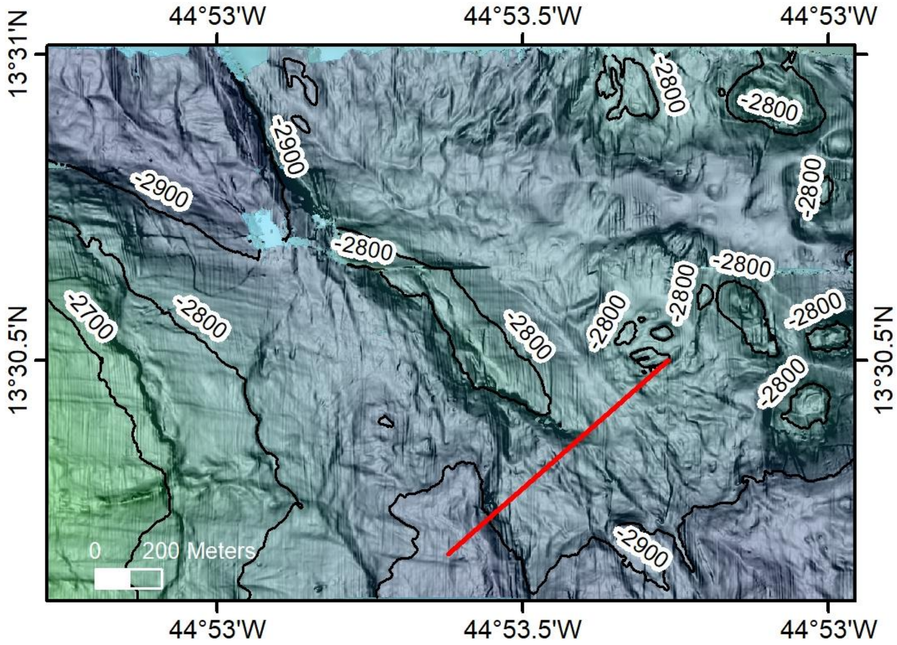

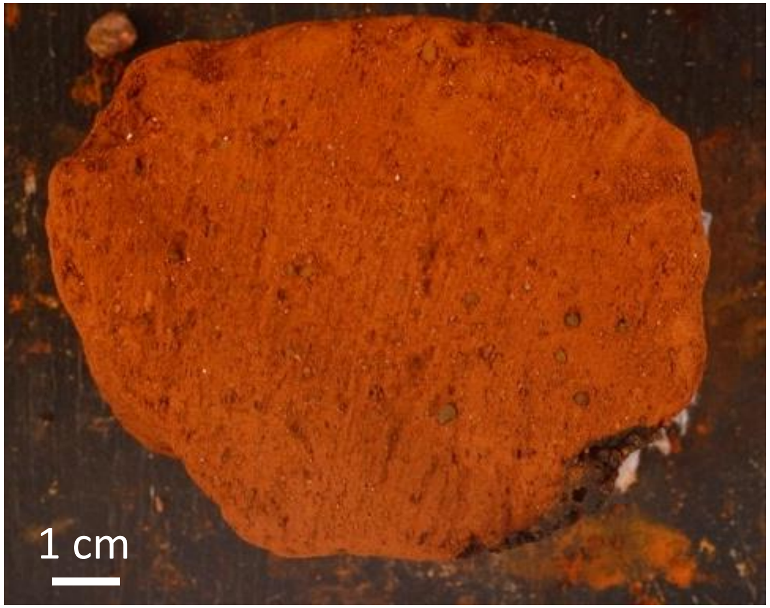

JC224_028_DR_05

Date / time (GMT)	Location	Start (latitude/ longitude)	End (latitude/ longitude)	Water depth (m)
19/03/22; 05:03	Sem 4	13˚30.263 N 44˚53.497W	13˚30.486 N 44˚53.285 W	2878

Description: Firm, relatively hard FeOOH material consisting of fine clay-silt sized grains ranging from orangish brown to reddish brown in colour. Texture is massive. Fluid like conduits are observed on sample with the edge comprising a thin (~1 mm) vein of dark grey, submetallic lustre Mn-oxide/magnetite.

Morphology – Type II chimney

Geological association – Anywhere along dredge line from Sem 4

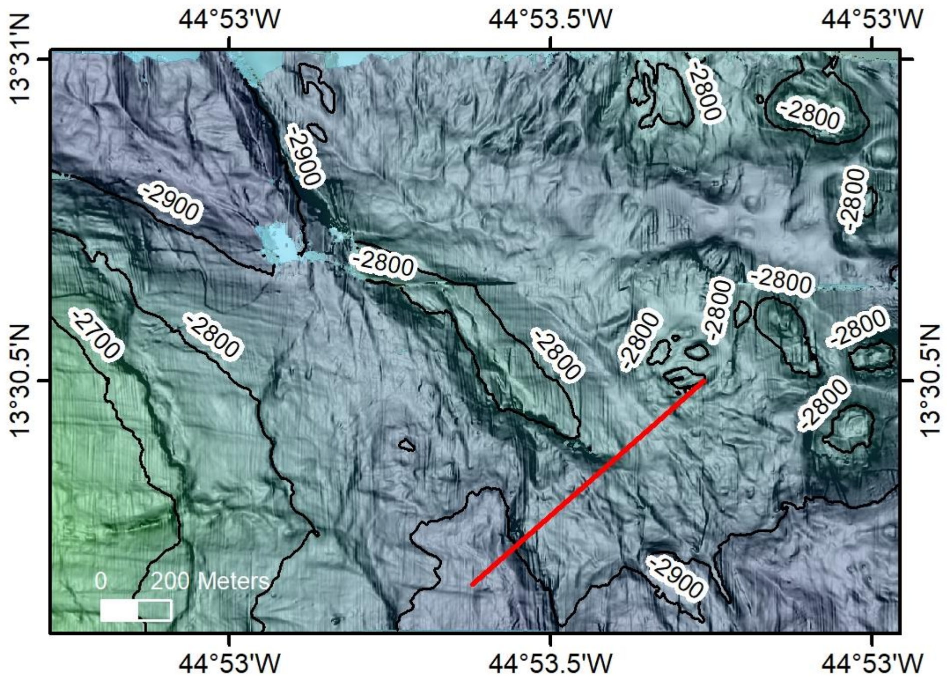

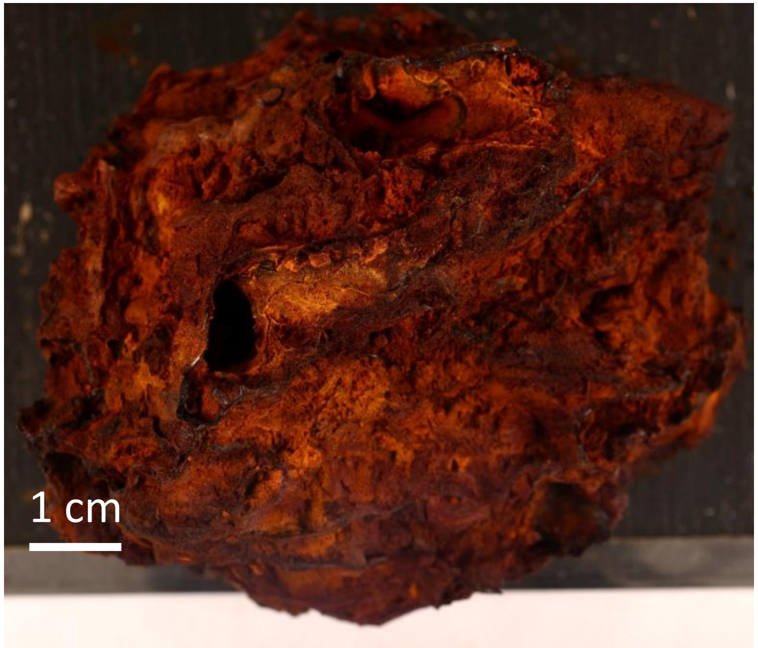

JC224_028_DR_018

Date / time (GMT)	Location	Start (latitude/ longitude)	End (latitude/ longitude)	Water depth (m)
19/03/22; 05:03	Sem 4	13˚30.263 N 44˚53.497W	13˚30.486 N 44˚53.285 W	2878

Description: full dredge. Assorted pieces of fine-grained, Fe-rich ochre. One piece is cut by a vein, fine grains of a platey lustrous material disseminated throughout.

Morphology – Type II ochre

Geological association – Dredge along Sem 4 that covers igneous rock and hydrothermal crust/sulphide mounds.

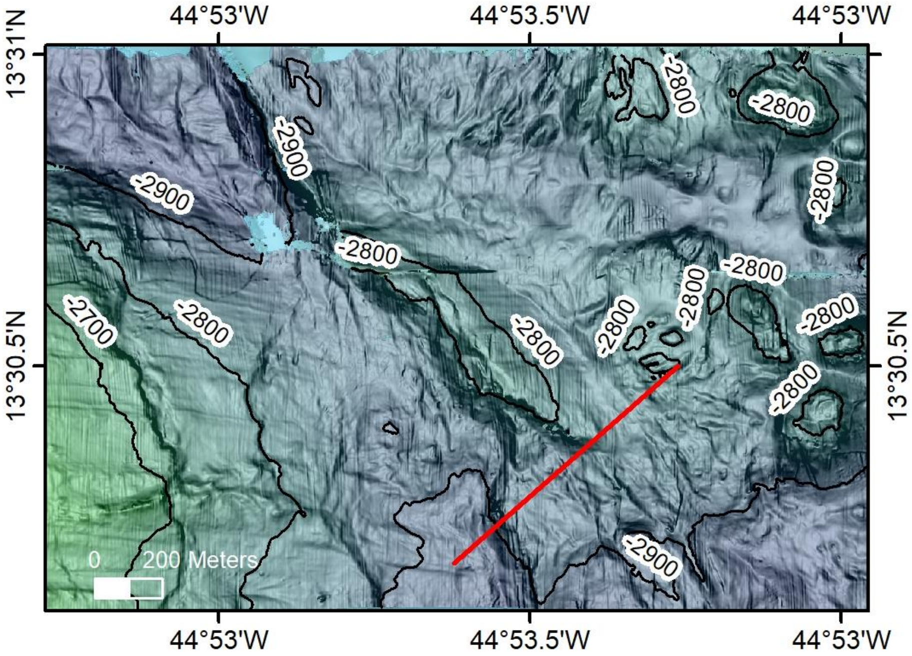

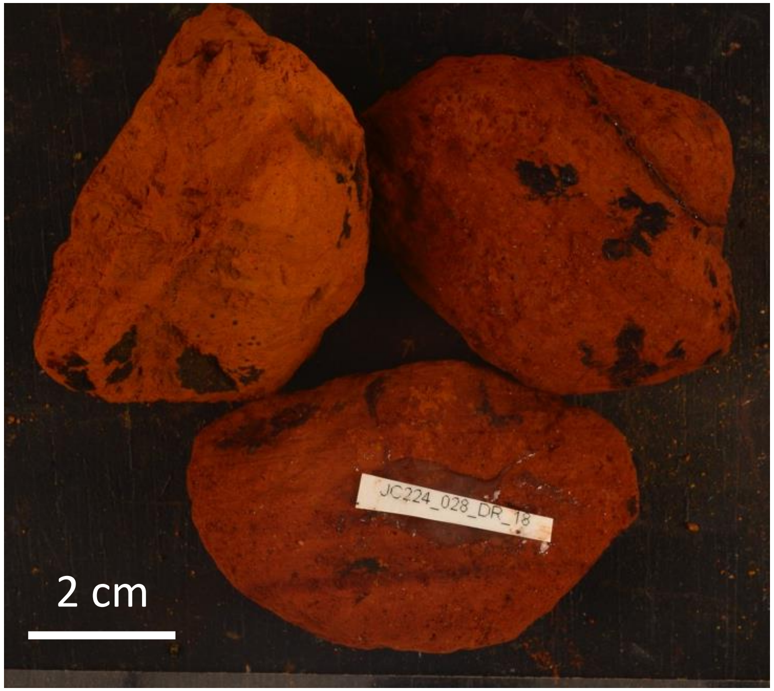

JC224_39_HY_02

Date / time (GMT)	Location	Latitude/ longitude	Water depth (m)
21/03/22; 21:25	Sem 1	13˚30.925 N<br>44˚59.204 W	2602

Description: crumbly, oxide covered pebble. Loosely cemented breccia, composed of fragments (<1 cm) of Mn and Fe-oxide rich material (black to orange).
Cut surface exhibits dominant Fe-Mn oxide surface with some FeOOH material. Exposed surface 90 degrees shows layering of FeOOH and Fe-Mn oxide. Veins of Fe-Mn oxide are ~2 mm thick, the FeOOH comprises of an orange red to a yellowish brown. Sample may represent an old chimney material. These layers indicate sample is a layered FeOOH and not a brecciated sample.
Morphology type – Type I layered
Geological association – Talus slope on Semyenov 1.

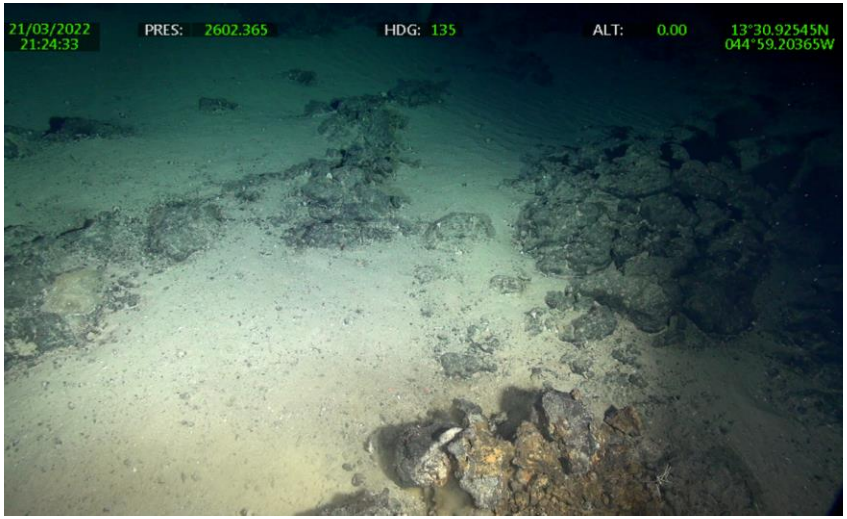

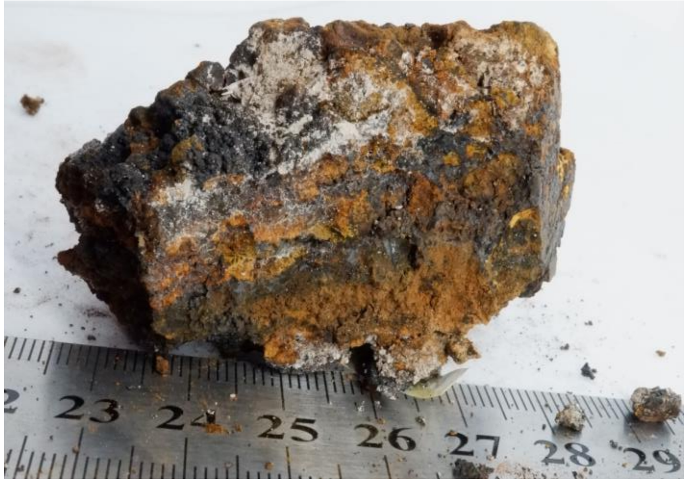

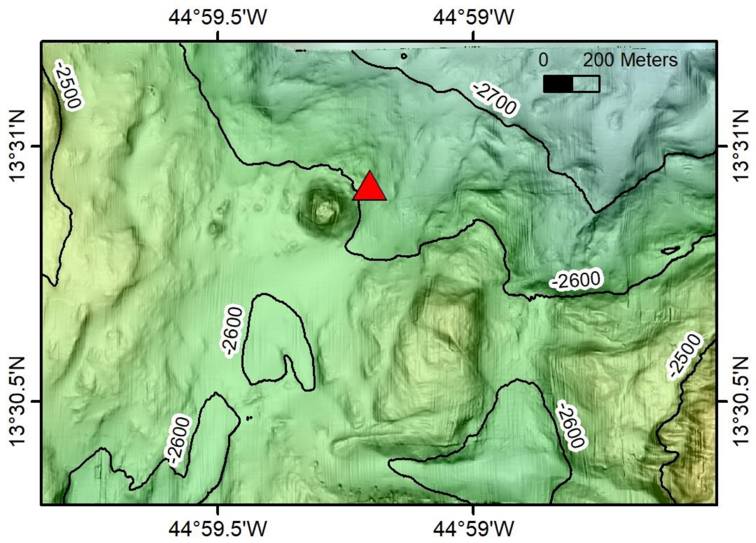

JC224_66_HY_05

Date / time (GMT)	Location	Latitude/ longitude	Water depth (m)
29/03/22; 20:13	Sem 1	13˚30.907 N<br>44˚59.374 W	2589

Description: Bright red external weathered surface and some specks of atacamite and traces of veins (suggestive of altered sulphides containing trace Cu).

Porous, altered sulphide breccia of sub-rounded to rounded clasts up to 1 cm (mostly 1-5 mm) of orange Fe-oxy-hydroxides, cemented by deep red to purple-black material. Sample exposed to seawater comprises of Fe-Mn oxide material which has also resulted in veins within the rock likely through cracks and fractures within the sample.

Morphology – Type I brecciated

Geological association – West of Sem-1, obtained on top of hydrothermal crust on a low lying mound.

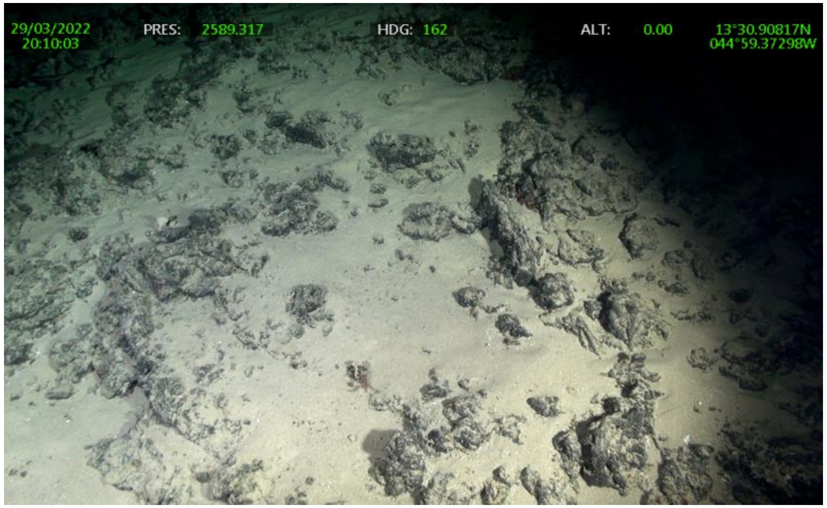

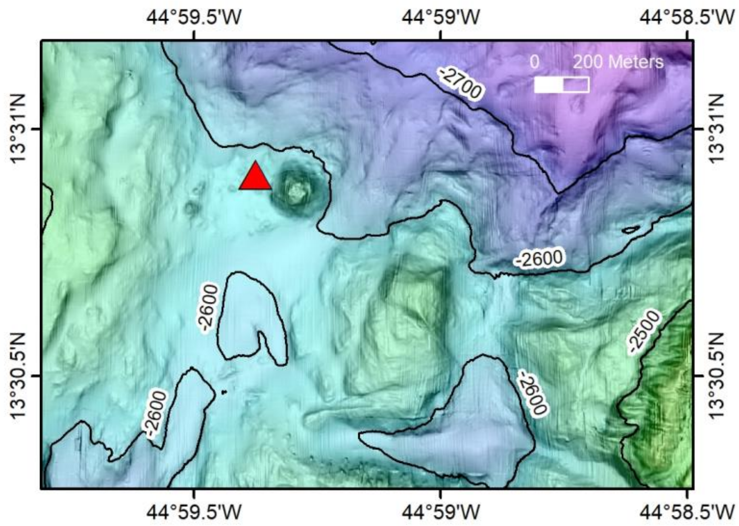

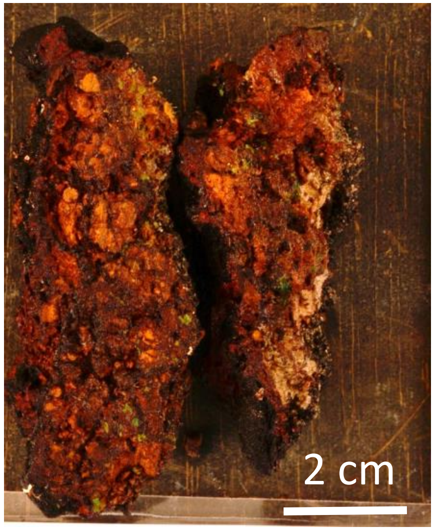

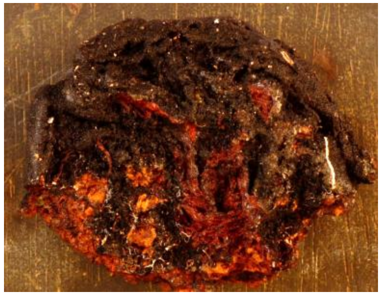

JC224_071_DR_01

Date / time (GMT)	Location	Start (latitude/ longitude)	End (latitude/ longitude)	Water depth (m)
30/03/22; 13:35	Sem 1	13˚30.856 N 44˚59.344 W	13˚30.879 N 44˚59.246 W	2584

Description: Sample shows an Fe-Mn crust where exposed to seawater. The other side exhibits FeOOH material with nontronite where there appears to be fluid conduits. Inside the fluid conduits are lined with FeOOH material with the edge having Fe-Mn oxide layer.
Cut surface exhibits layering textures of Fe-Mn oxide and FeOOH. Each layer is quite thin (<1 mm – 1 mm). Cut surface shows FeOOH material ranging from derker brownish red to a lighter yellowish brown.There are some thick veins of Fe-Mn oxide.hematite.
Morphology – Type I chimney
Geological association – Dredge on Sem-1

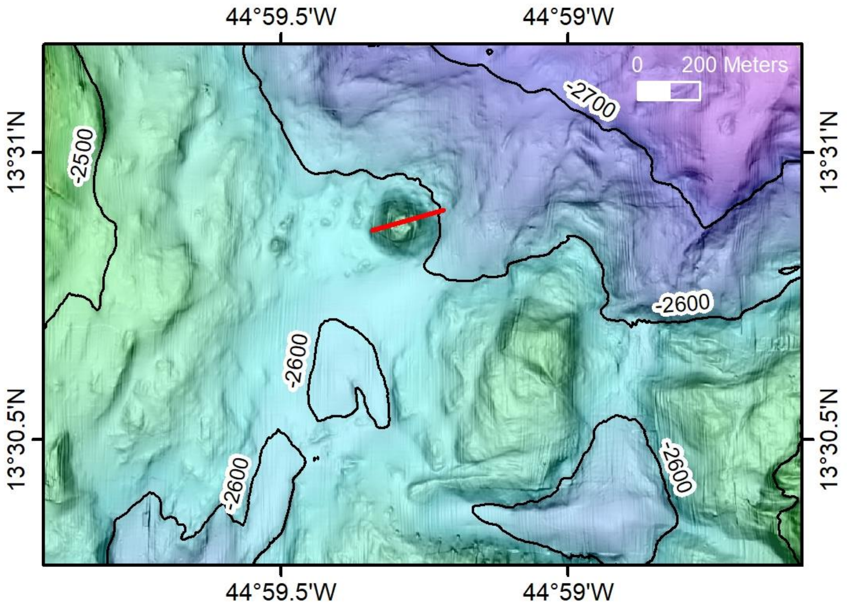

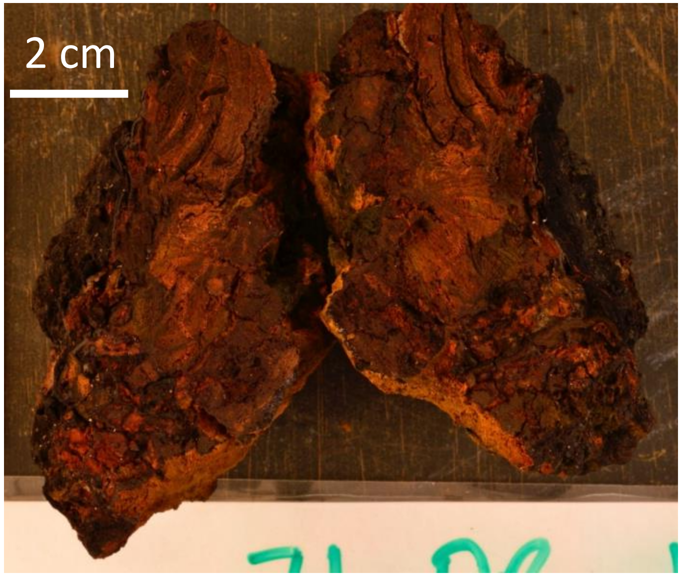

JC224_071_DR_02

Date / time (GMT)	Location	Start (latitude/ longitude)	End (latitude/ longitude)	Water depth (m)
30/03/22; 13:35	Sem 1	13˚30.856 N 44˚59.344 W	13˚30.879 N 44˚59.246 W	2584

Description: Whole sample is very similar to previous sample. This sample shows layering on cut surface. This sample exhibits more dark colouration of FeOOH material being mostly dark brownish red. Layering is more chaotic and can observe small layering of various colour FeOOH material and Fe-Mn oxide. Where chimney textures occur, nontronite forms within suggesting reducing environment.

Morphology – Type I chimney

Geological association – Dredge on Sem-1

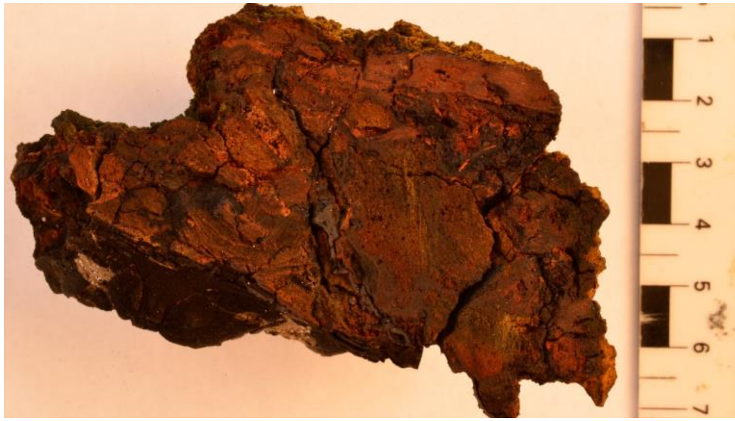

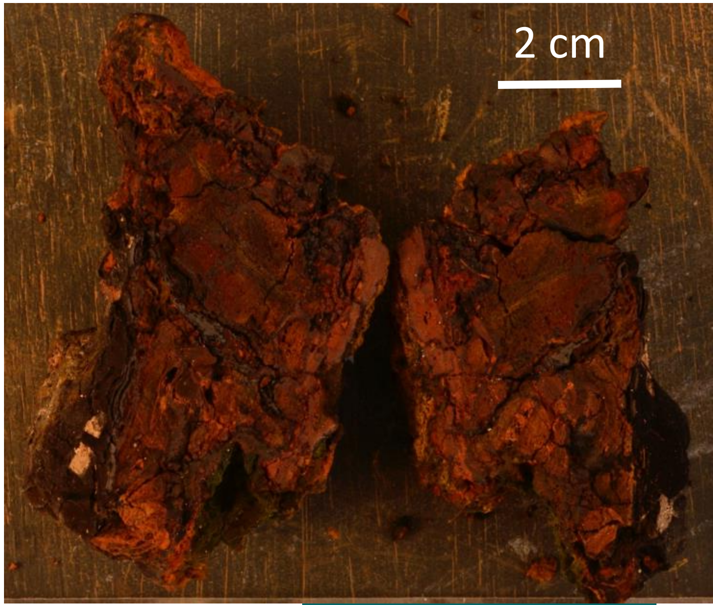

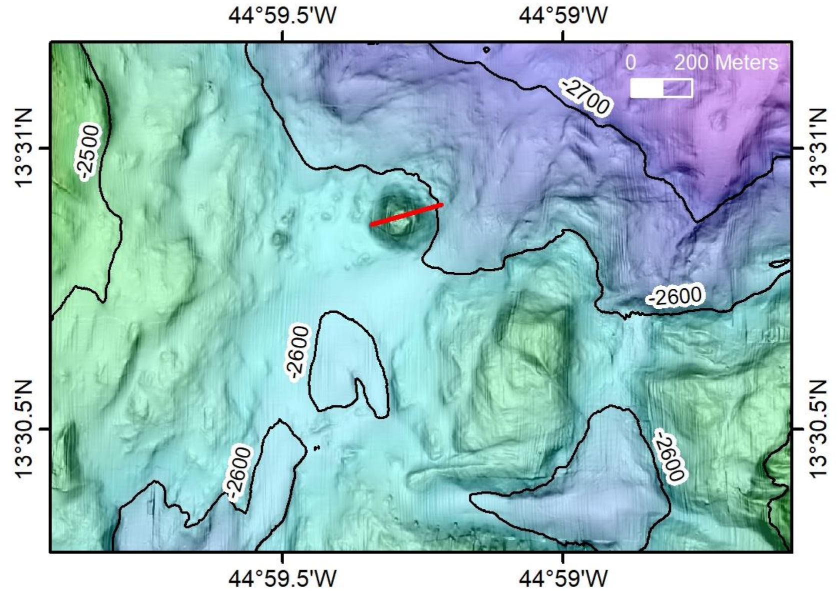

JC224_071_DR_03

Date / time (GMT)	Location	Start (latitude/ longitude)	End (latitude/ longitude)	Water depth (m)
30/03/22; 13:35	Sem 1	13˚30.856 N 44˚59.344 W	13˚30.879 N 44˚59.246 W	2584

Description: Fe-oxyhydroxide with Mn-oxide and FeOOH layer.
Sample has fluid conduit lined with nontronite.
Similar to previous Type I chimney samples.
Morphology – Type I chimney
Geological association – Dredge on Sem-1

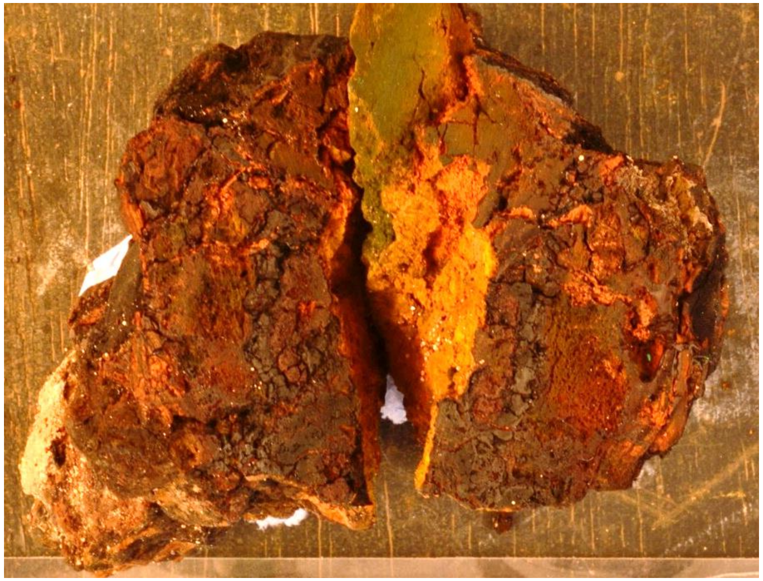

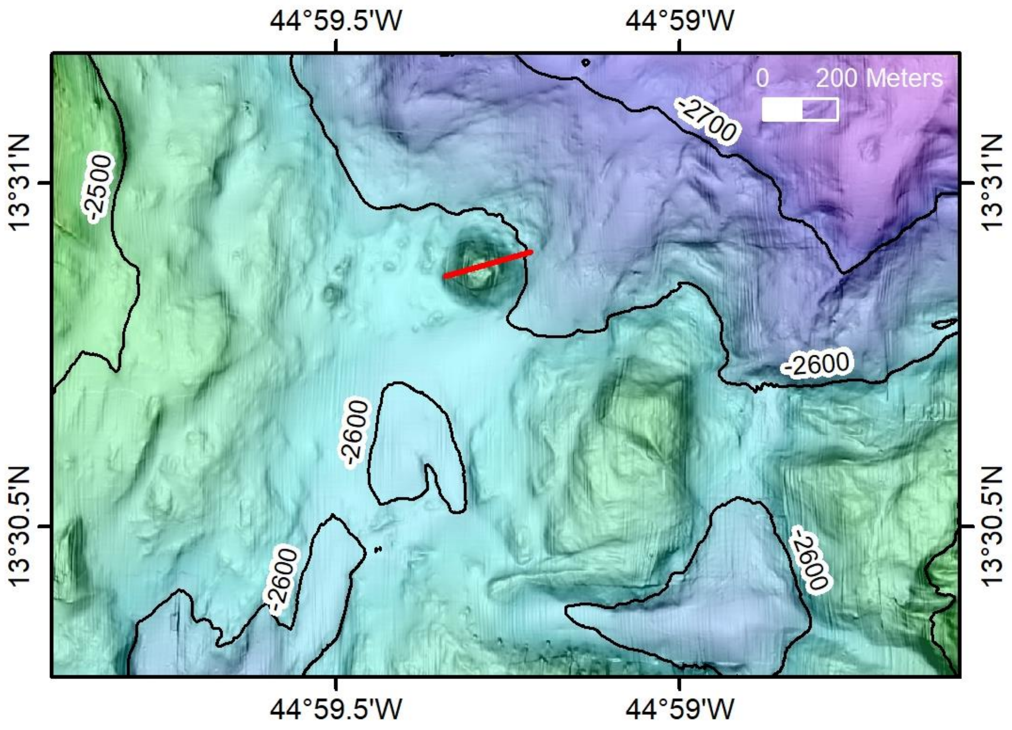

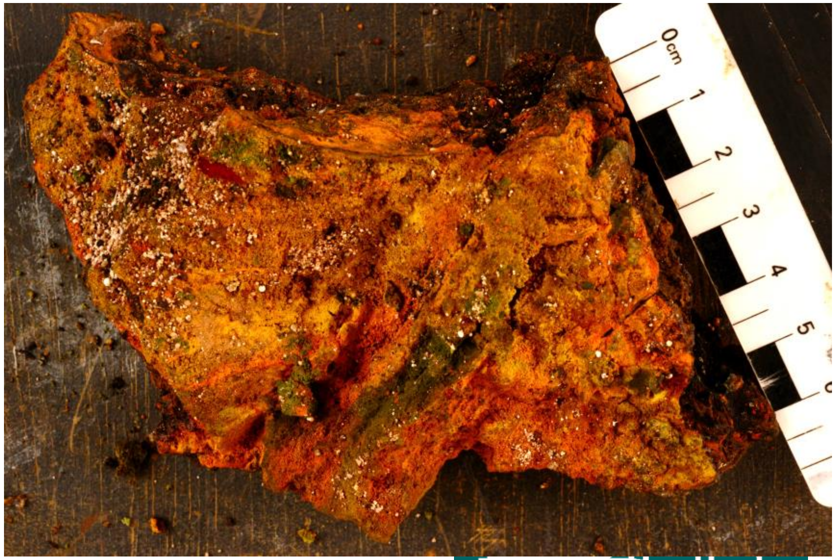

JC224_071_DR_04

Date / time (GMT)	Location	Start (latitude/ longitude)	End (latitude/ longitude)	Water depth (m)
30/03/22; 13:35	Sem 1	13˚30.856 N 44˚59.344 W	13˚30.879 N 44˚59.246 W	2584

Description: Fe-oxyhydroxide with Mn-oxide coating on exposed surface and no nontronite. Bowl like shaped hole that could represent a clast that fell out or this is a piece of a hydrothermal chimney that fell off. FeOOH material has a brownish red colour and consist of fine grained material. Sample has Se of 104 ppm & 451 ppm of Mo. However has low Cu and other metals.

Morphology – Massive

Geological association – Sem -1 mound.

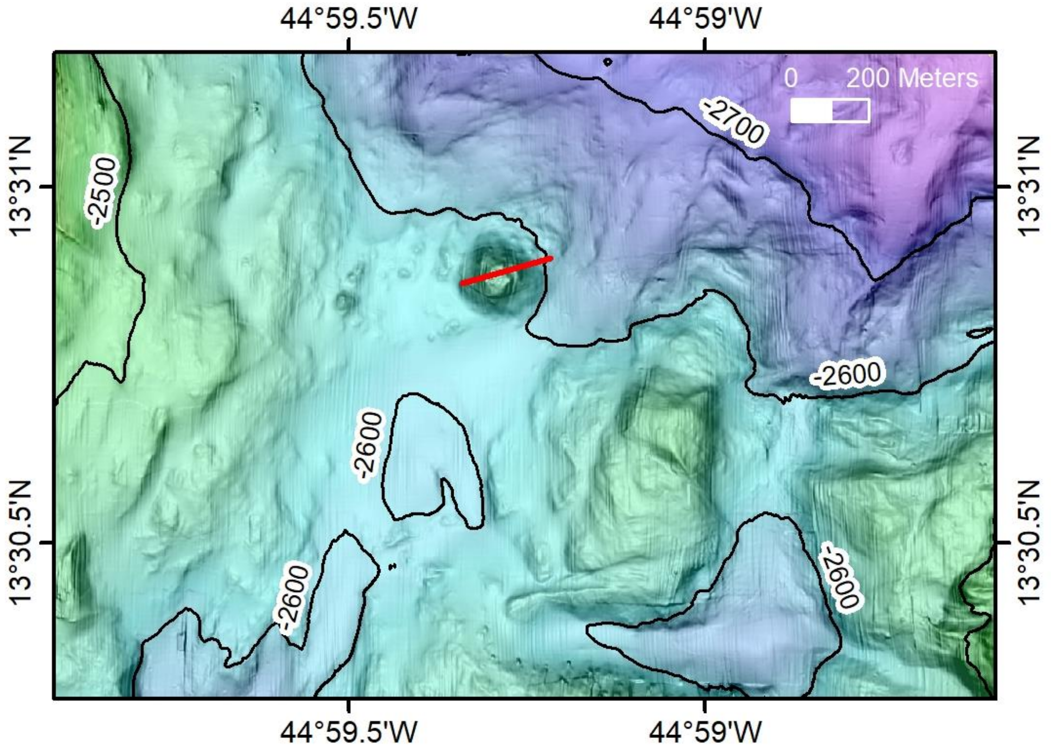

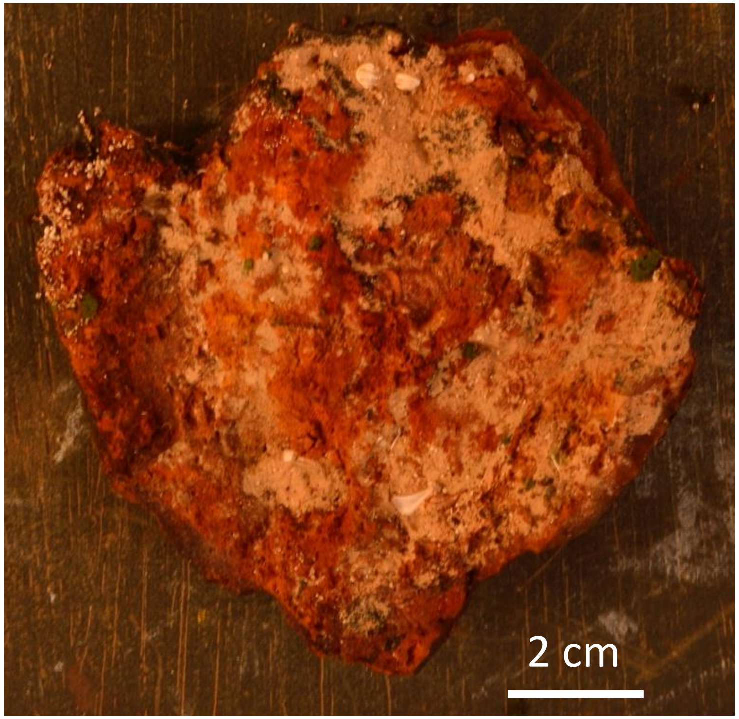

JC224_071_DR_07

Date / time (GMT)	Location	Start (latitude/ longitude)	End (latitude/ longitude)	Water depth (m)
30/03/22; 13:35	Sem 1	13˚30.856 N 44˚59.344 W	13˚30.879 N 44˚59.246 W	2584

Description: Firm FeOOH sample ranging from a dominant yellowish brown colour to sometimes orangish brown colour. Layering textures are excellent repeating FeOOH and Fe-Mn oxide layers (~1mm). Nontronite occurs as fine, powdery soft material within fluid conduits. Mn-oxide coats the surface of sample exposed to seawater.

Morphology – Type I chimney

Geological association – Sem -1 mound.

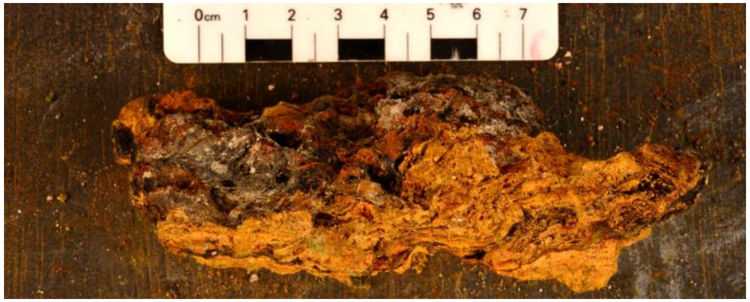

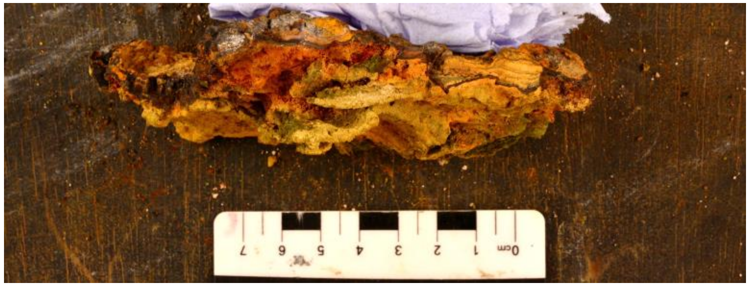

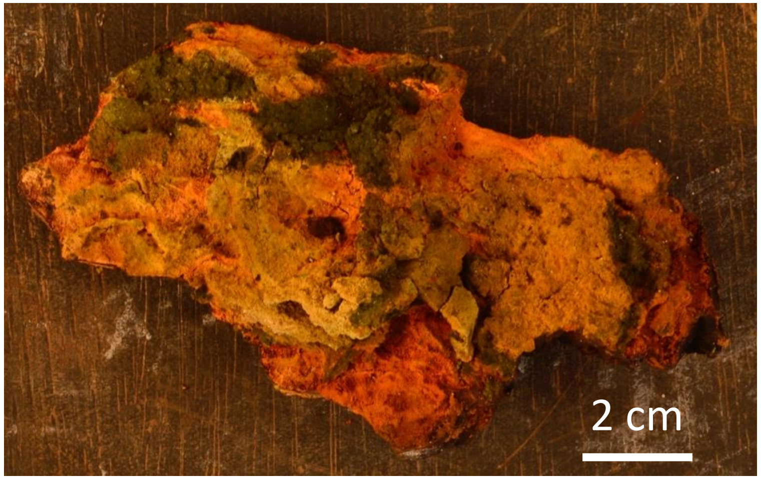

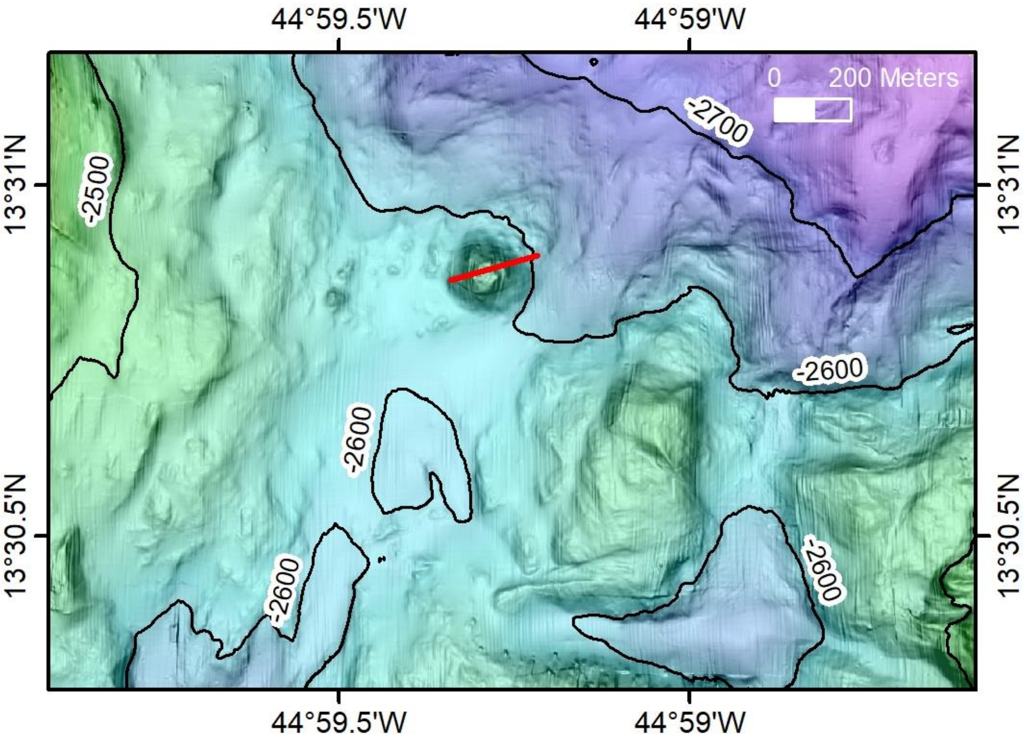

JC224_071_DR_09

Date / time (GMT)	Location	Start (latitude/ longitude)	End (latitude/ longitude)	Water depth (m)
30/03/22; 13:35	Sem 1	13˚30.856 N 44˚59.344 W	13˚30.879 N 44˚59.246 W	2584

Description: Sample comprises of clay like grains orange in colour. Mn-oxide occurs as a coating where sample was exposed to seawater. Sample has patches of nontronite, likely a result of the dredge and not actually there in-situ. Small <1 mm platy vitreous crystals could be barite or amorphous silica.

Morphology – Type I Ochre

Geological association – Sem -1 mound.

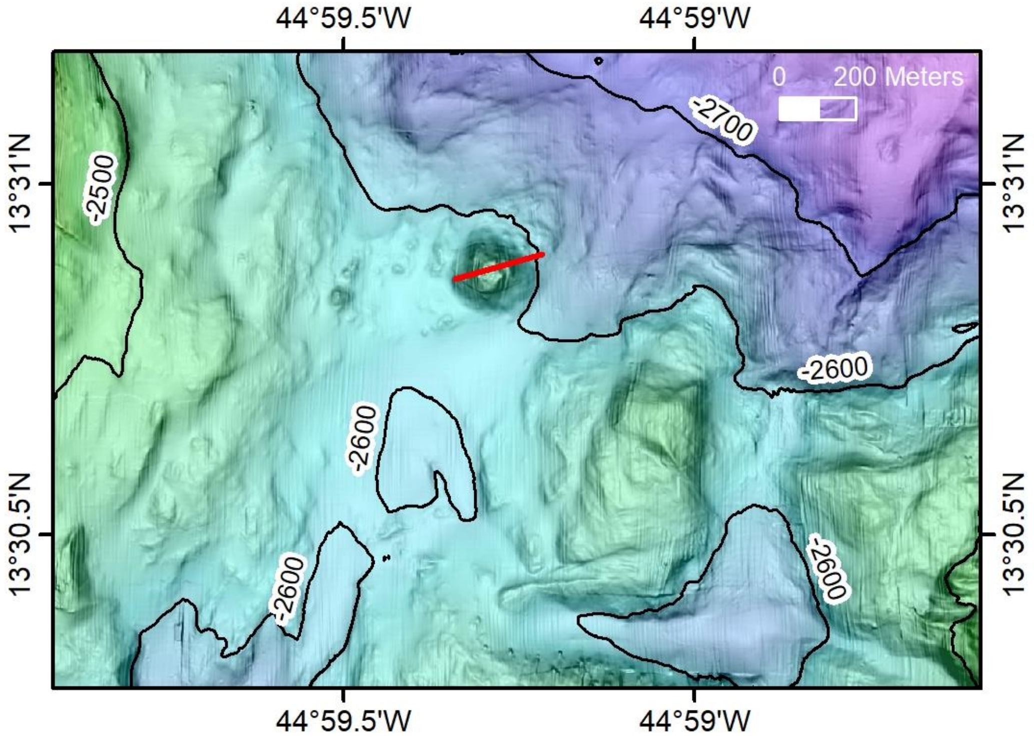

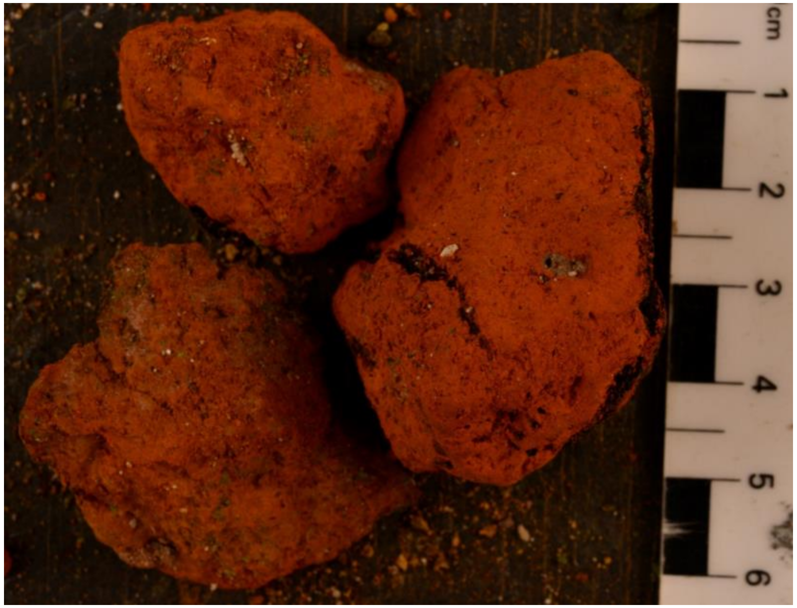

JC224_071_DR_012

Date / time (GMT)	Location	Start (latitude/ longitude)	End (latitude/ longitude)	Water depth (m)
30/03/22; 13:35	Sem 1	13˚30.856 N 44˚59.344 W	13˚30.879 N 44˚59.246 W	2584

Description: 16 fragments of potentially pervasively weathered hydrothermal chimney and or Fe-oxyhydroxides. Fragments exhibit layering of FeOOH and ~3mm - <1 mm Fe-Mn oxide, representing possible growth of chimneys.
Morphology – Type I chimney
Geological association – Sem -1 mound.

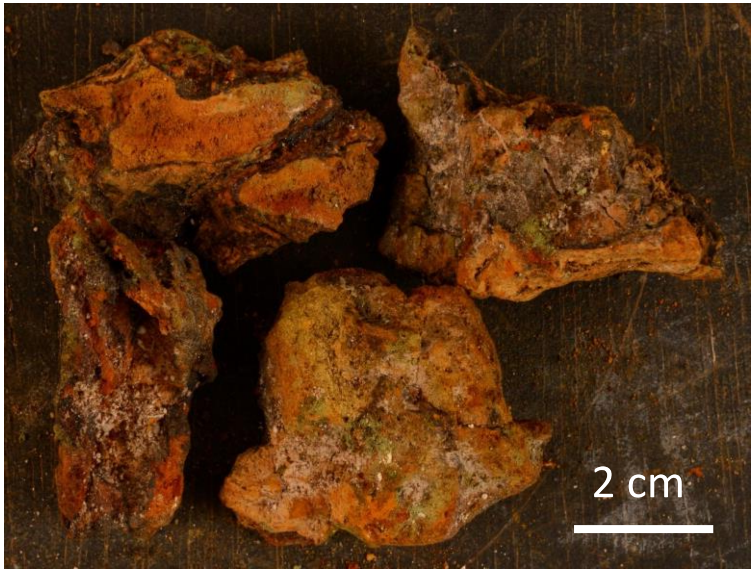

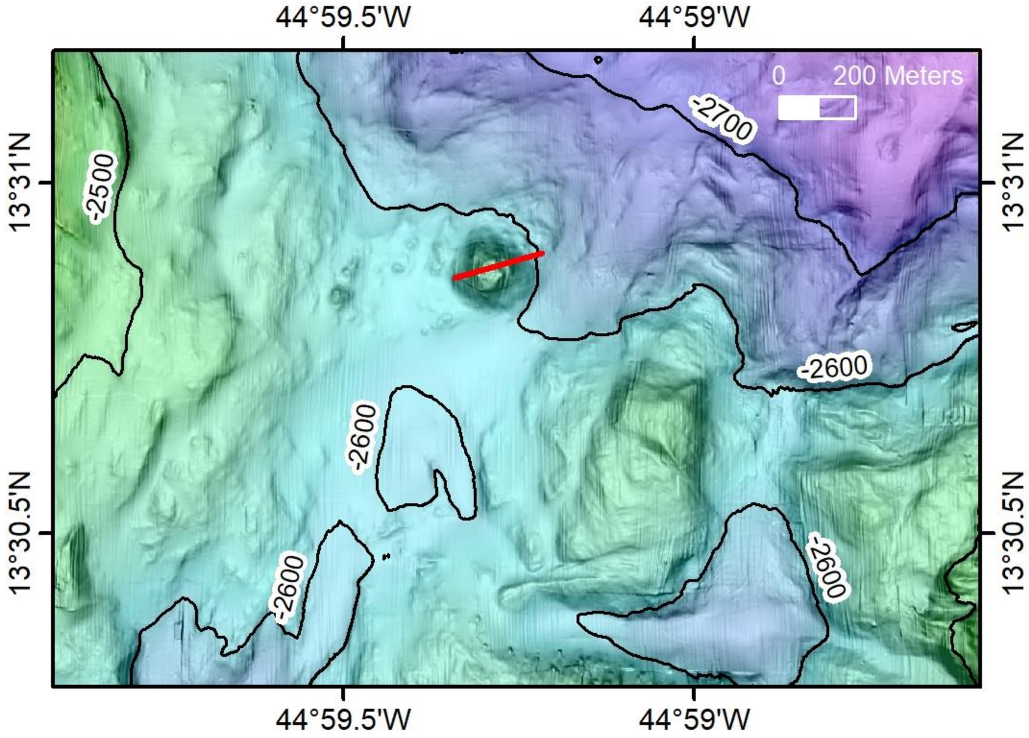

JC224_77_HY_02

Date / time (GMT)	Location	Latitude/ longitude	Water depth (m)
31/03/22; 19:21	Sem 2	13˚30.807 N<br>44˚57.757 W	2429

Description: angular, broken edge red fragment with Fe coating, small yellow underside. Mn oxide coated vuggy pale cream coloured, banded soft material, which is clay-like in texture with fine but chaotic banding of cream and brown material. White mineral is likely barite.
Sample shows brecciation in parts of the sample of small ~0.5 cm rounded clasts. Veins of Fe-Mn oxide ~5 mm cross cut sample. FeOOH material consist of slight reddish/yellowish brown to reddish brown.
Morphology – Type II brecciated
Geological association – Obtained in hydrothermal crust with diffuse hydrothermal venting

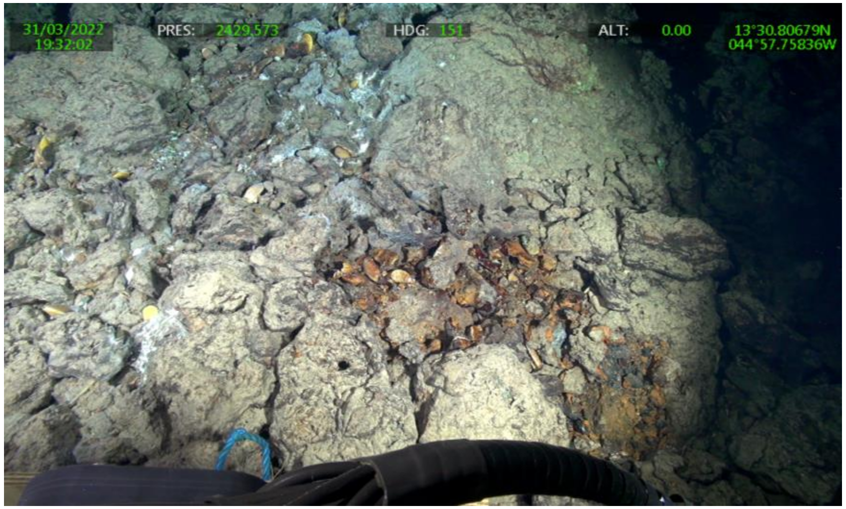

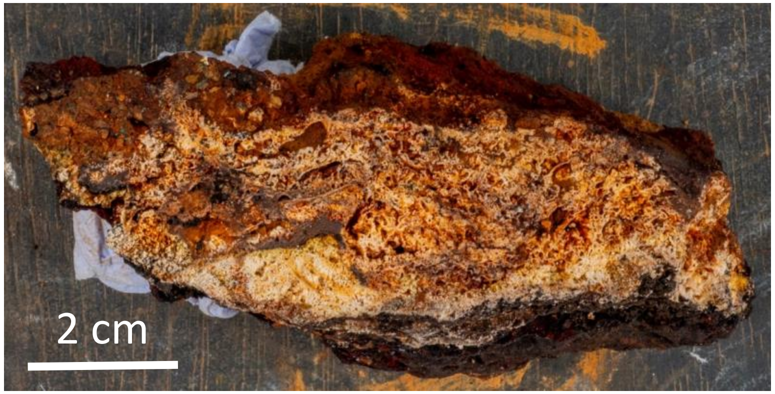

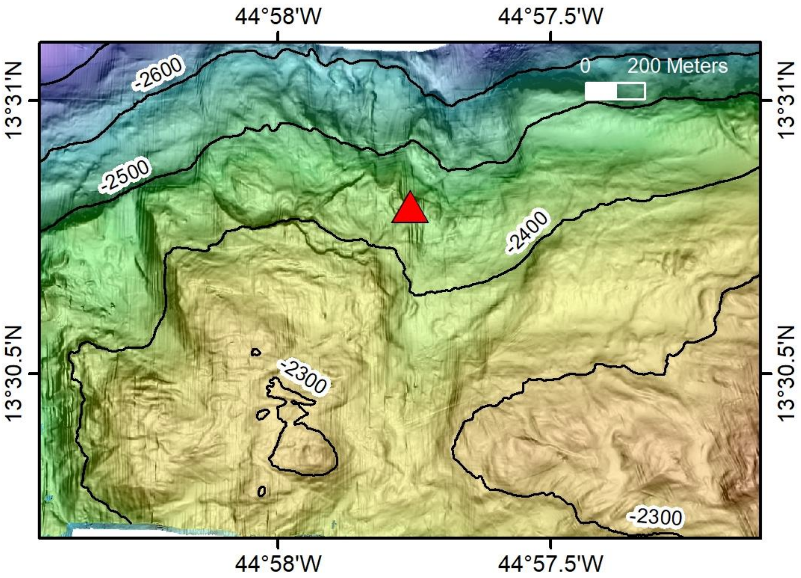

JC224_77_HY_03

Date / time (GMT)	Location	Latitude/ longitude	Water depth (m)
31/03/22; 19:30	Sem 2	13˚30.807 N<br>44˚57.757 W	2429

Description: angular fragment of brown-orange breccia. Polymict clasts of pale orange-brown to cream coloured fragments. These clasts comprise up to 1 cm and <1 mm in size and commonly subrounded with some subangular to angular clasts. Many are elongated and micro-fractured. Clasts could be Barite and FeOOH material.

Matrix is composed of dark brown fine-grained material, which is also soft. Small patch of a green material (atacamite?) and an exterior veneer of orange-brown Fe-oxide.

Morphology – Type II brecciated
Geological association – Obtained in hydrothermal crust with diffuse hydrothermal venting

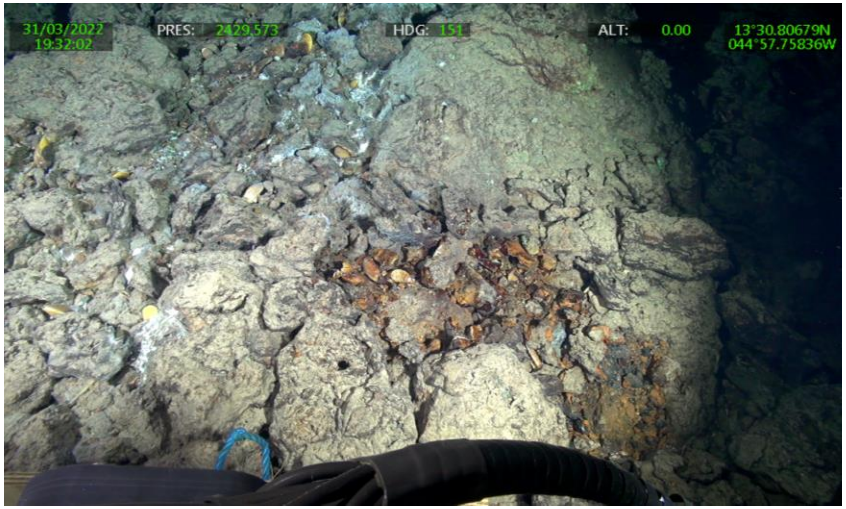

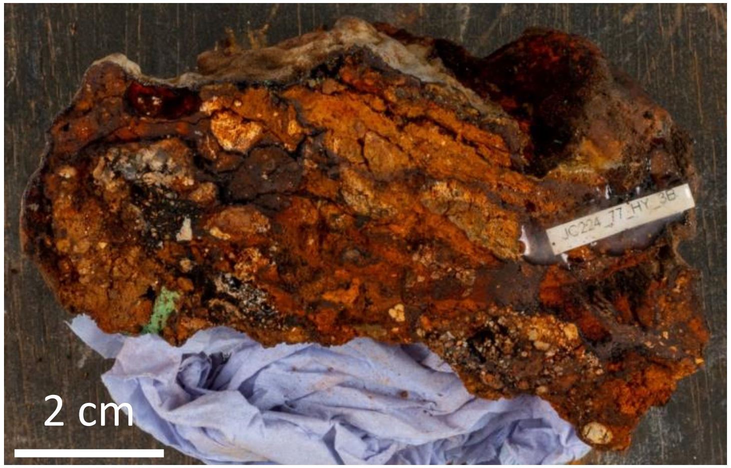

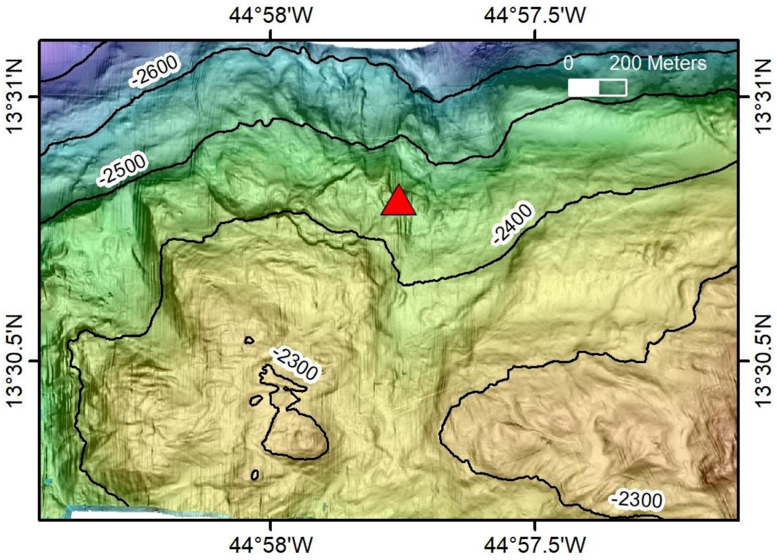

JC224_82_HY_01

Date / time (GMT)	Location	Latitude/ longitude	Water depth (m)
01/04/22; 12:47	Sem 5	13˚30.660 N<br>44˚56.060 W	2222

Description: Fe-Mn coating on surface exposed to seawater with rest of sample or reddish - orange brown colour. Cut sample exhibits brecciated textures with also massive textures more akin to ochre. Where the lighter orange red colour dominates is where sample most appears like ochre comprising of soft clay like grains. Where sample is darker to a dark orange red to reddish black, sample is harder and shows brecciated textures.

Morphology – Type I brecciated

Geological association – Located on hydrothermal crust on top of hydrothermal mound

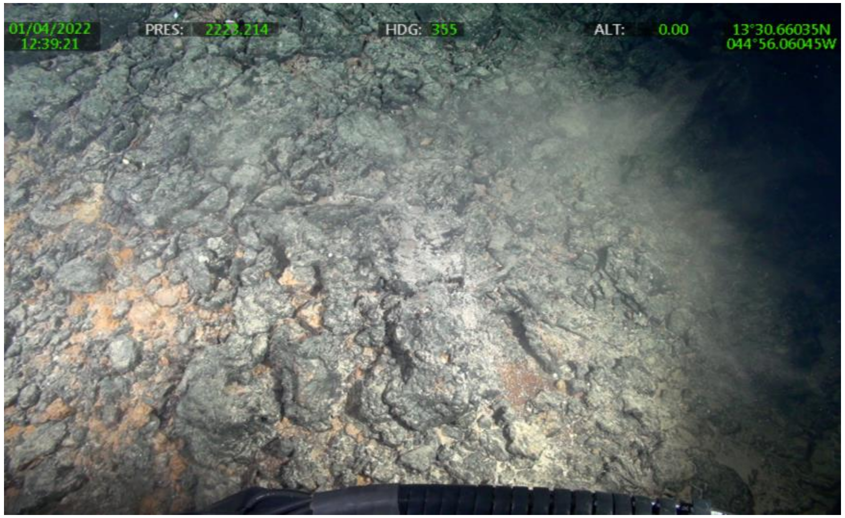

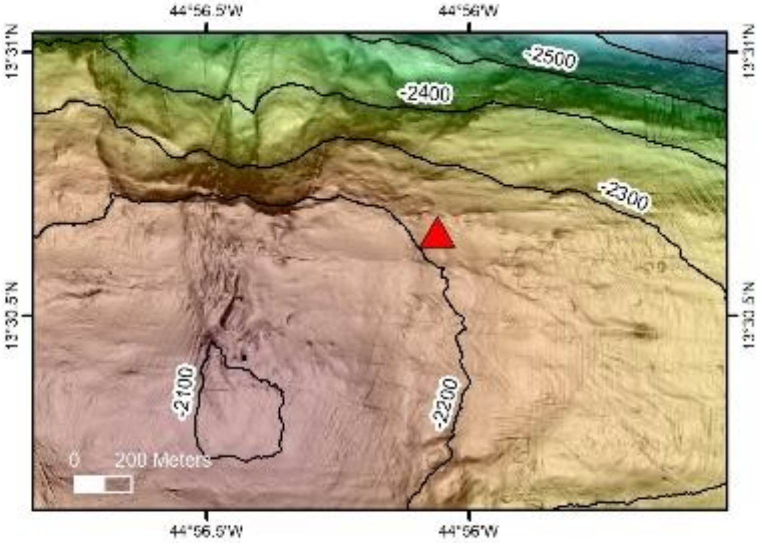

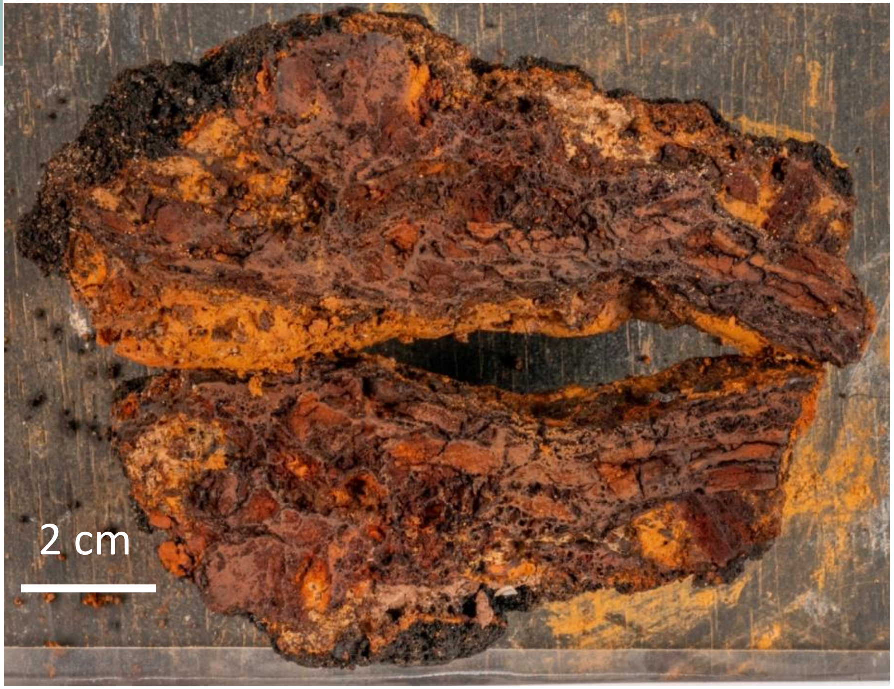

JC224_82_HY_05

Date / time (GMT)	Location	Latitude/ longitude	Water depth (m)
01/04/22; 13:56	Sem 5	13˚30.687 N<br>44˚56.147 W	2230

Description: Sample is reddish brown to orange brown in colour. Surface exposed to seawater has a Fe-Mn crust. Possible clast like structures and potential hematite or Fe-Mn oxide veins. Darker colour likely a result of increased Mn content or presence of hematite. Sample is ocherous and apart from the veining, appears massive.
Morphology – Ocherous
Geological association – Located on hydrothermal crust with exposed massive sulphide

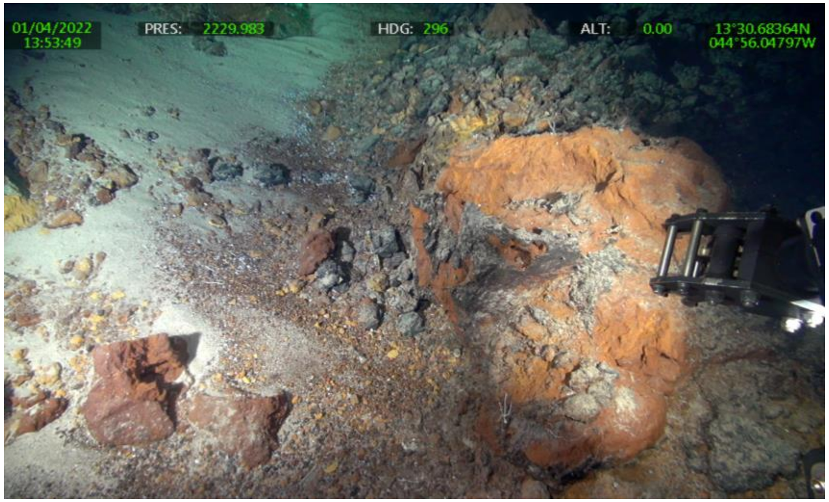

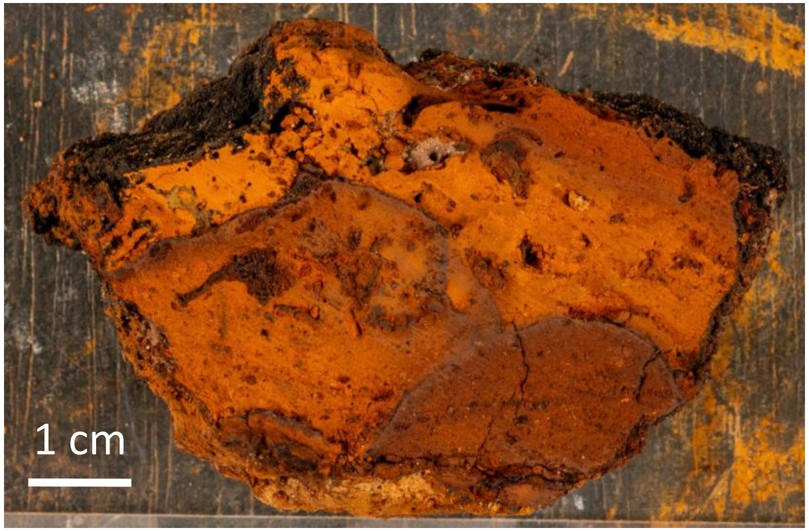

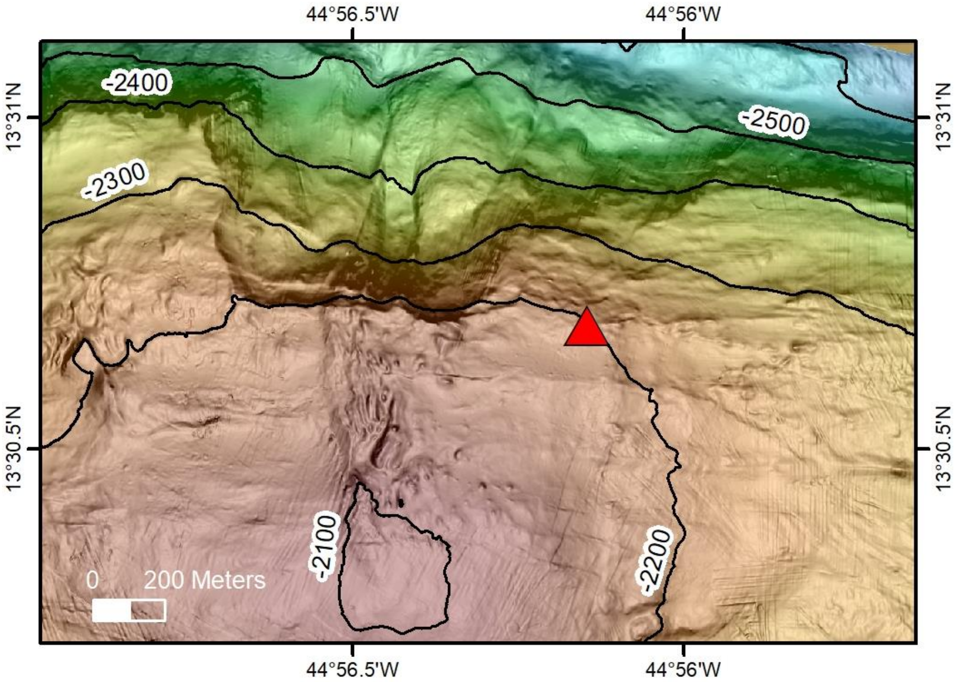

JC224_82_HY_06

Date / time (GMT)	Location	Latitude/ longitude	Water depth (m)
01/04/22; 14:14	Sem 5	13˚30.684 N<br>44˚56.049 W	2230

Description: Sample consists of two separate rocks, an underlying pyrite dominated massive sulfide and an overlying Fe-oxide crust. Atacamite observed in boundary between the two rocks and may act as a cap for rising Cu-rich fluids.

The pyrite dominated massive sulphides appear porous/vuggy. Where appears extremely vuggy, there is colouration to orange brown, likely where increased oxidation has taken place to form FeOOH. Veins of an amorphous, light grey mineral near top of sample possibly marcasite.

The FeOOH cap comprises of colouration rom orange brown to lighter yellowish orange to dark reddish grey and green. Sample overall appears massive but with also brecciated textures at the massive sulphide boundary. Atacamite veins (green) cross cut the FeOOH. The black hard parts of the crust are Fe-Mn oxide.

Morphology – Massive FeOOH (82_HY_06_a) Pyrite dominated massive sulphide (82_HY_06_b)

Geological association – Located where massive sulphide is exposed.

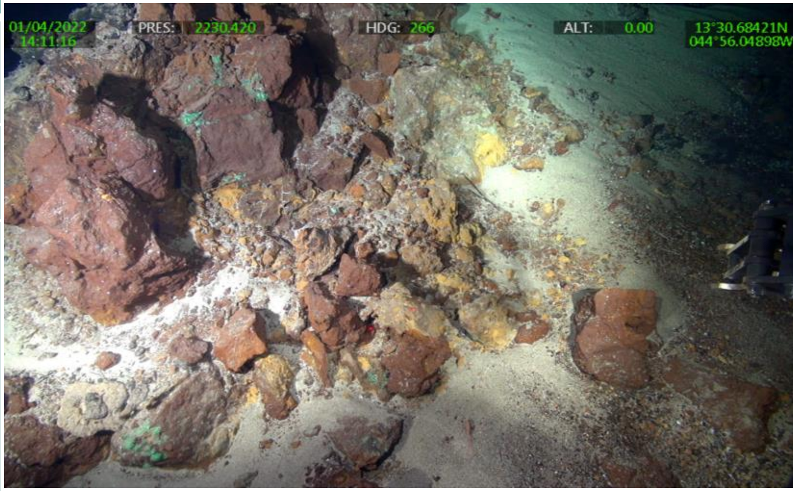

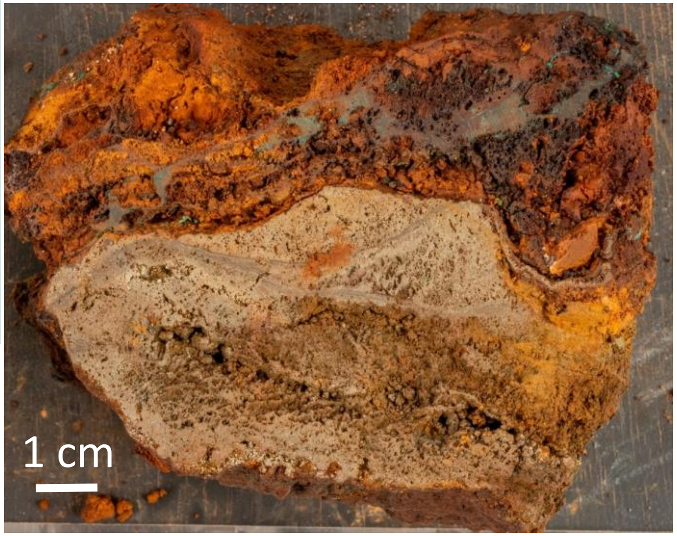

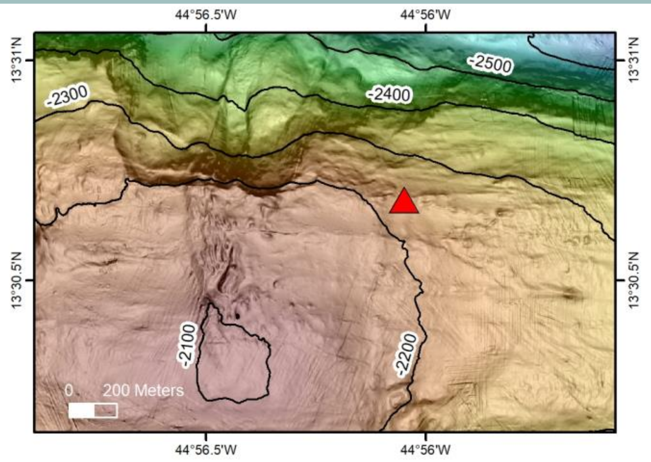

JC224_82_HY_09

Date / time (GMT)	Location	Latitude/ longitude	Water depth (m)
01/04/22; 16:40	Sem 5	13˚30.690 N<br>44˚56.122 W	2206

Description: FeOOH material comprising of soft, fine, clay – silt like grains. Colour is dominantly brownish red. Sample exhibits good layering textures. Layers change from a brownish red to a yellowish brown FeOOH with layers ranging from >1 mm to ~1 mm.

Morphology – Type II layered

Located on hydrothermal crust with exposed massive sulphide

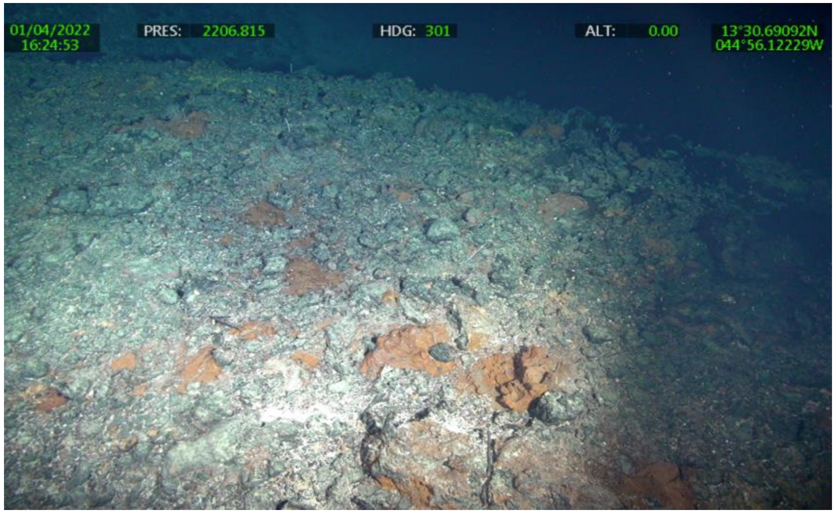

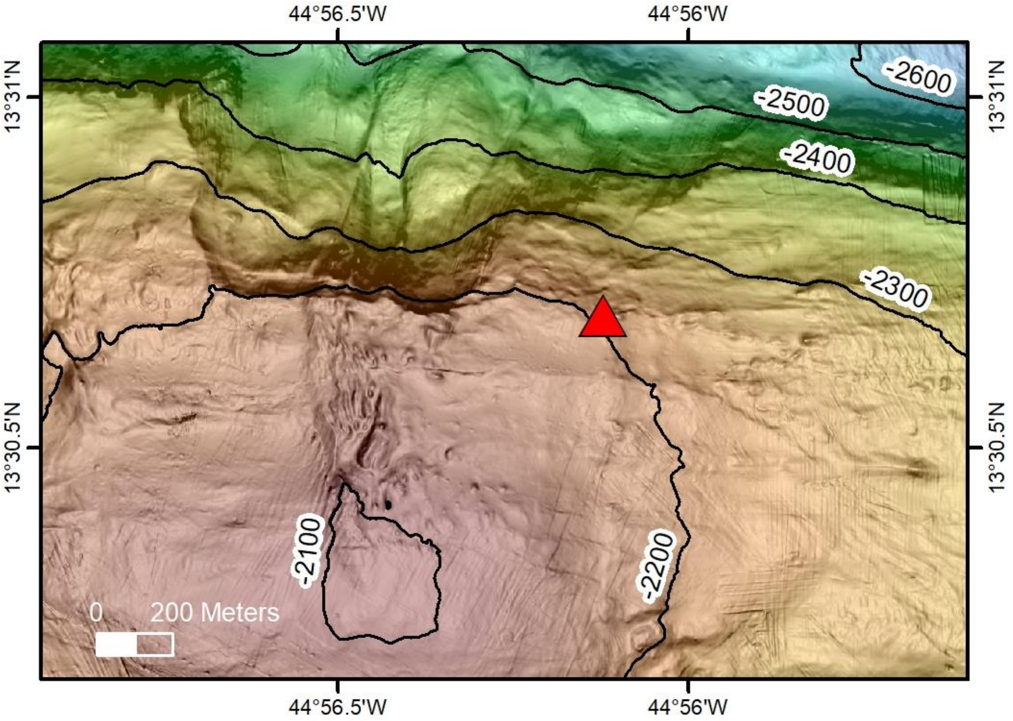

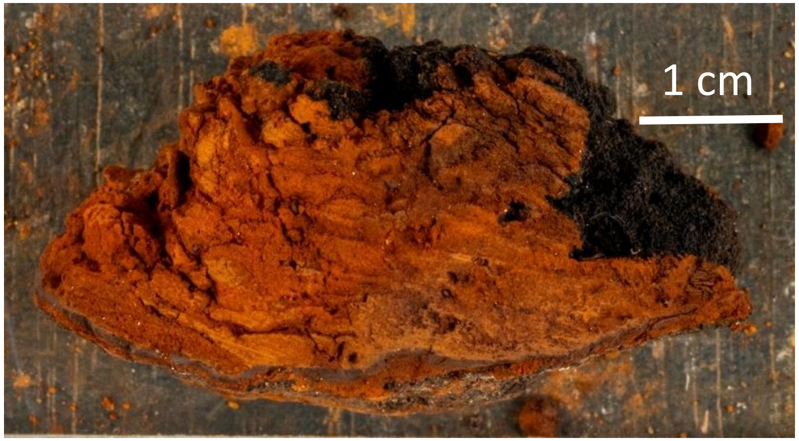

JC224_82_HY_10

Date / time (GMT)	Location	Latitude/ longitude	Water depth (m)
01/04/22; 16:53	Sem 5	13˚30.671 N<br>44˚56.112 W	2206

Description: Small, black and orange Fe-oxyhydroxide sample. Fe-oxyhydroxide with an Fe-Mn coating on surface exposed to seawater. Possible veins of hematite within the rock. Fe-Mn oxide veins appear as thin layers. Large hole in sample could potentially be a relict chimney. FeOOH material comprises of fine grains, relatively hard to other FeOOH and colour ranges from brownish red to orange brown. Groundmass comprises of clay like grains. Texture of sample is massive with some Fe-Mn oxide veins. Sample could by hydrothermal chimney but uncertain.
Morphology – Massive
Geological association – Located on hydrothermal crust.

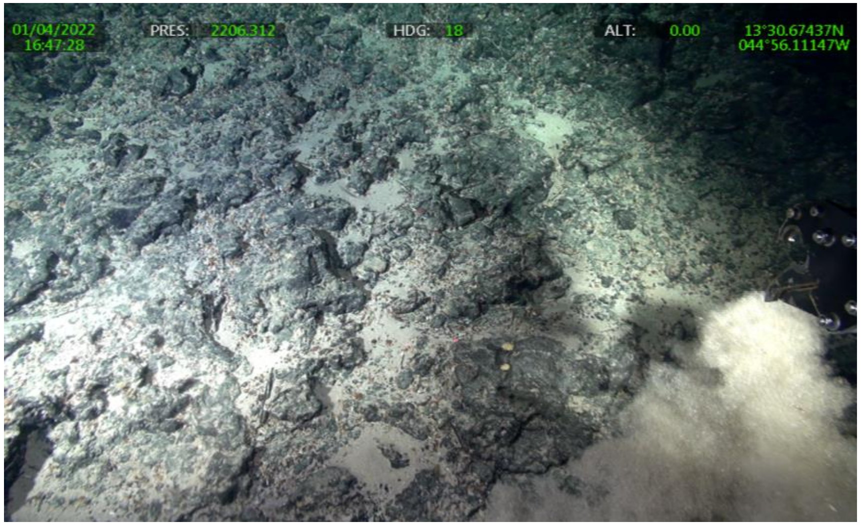

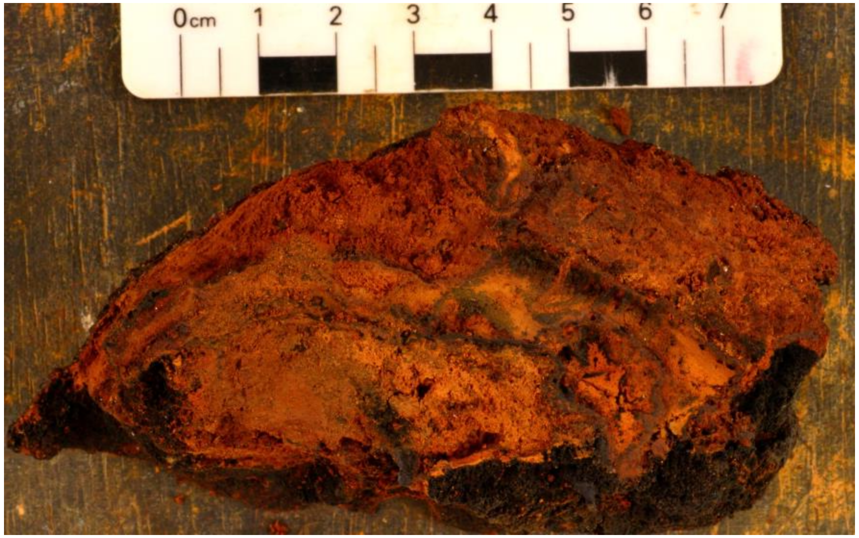

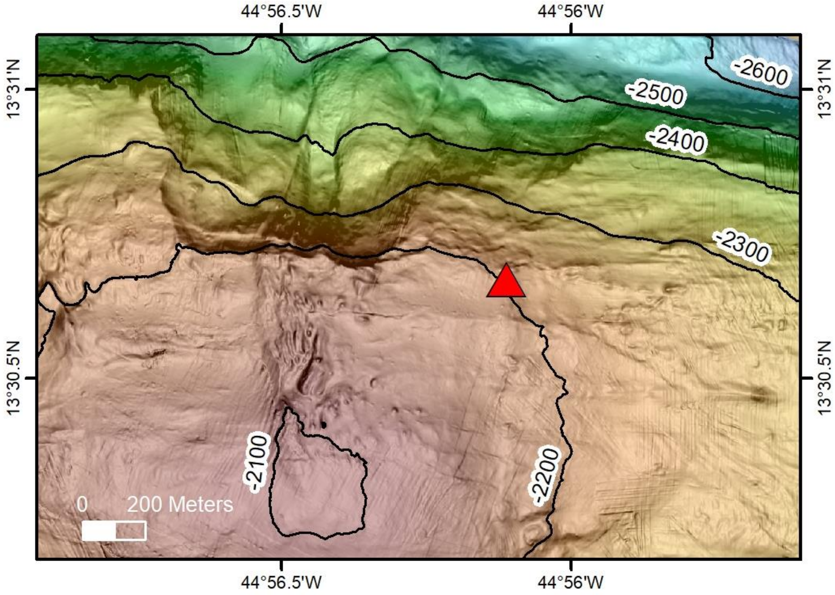

JC224_82_HY_11

Date / time (GMT)	Location	Latitude/ longitude	Water depth (m)
01/04/22; 17:49	Sem 5	13˚30.684 N<br>44˚56.164 W	2211

Description: Small, blade Fe-Mn crust piece. Broken edge and yellow to beige underside. Sample is an Fe-oxyhydroxide with a Fe-Mn crust. Possible 1 mm veins of hematite.

Cut surface shows a massive ocherous texture with veins of Fe-Mn oxide ~3 mm thickness. Groundmass comprises of orange red clay like grains. Ocherous sample is relatively hard.
Morphology – Ocherous
Geological association – Located on hydrothermal crust.

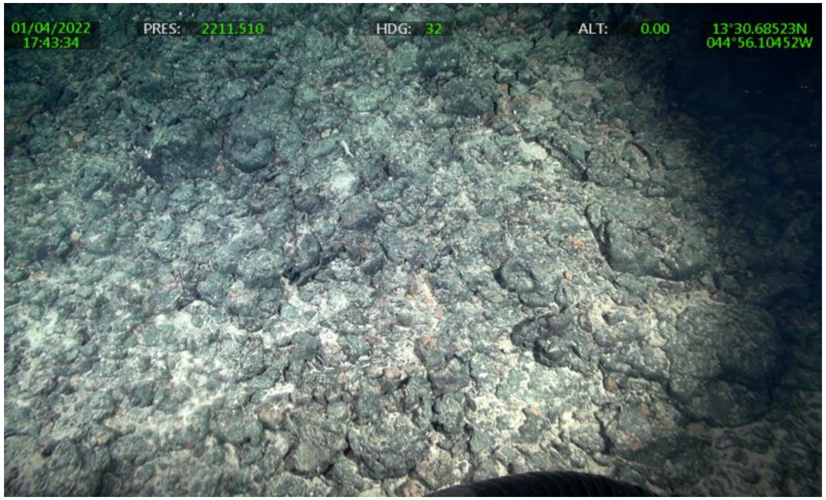

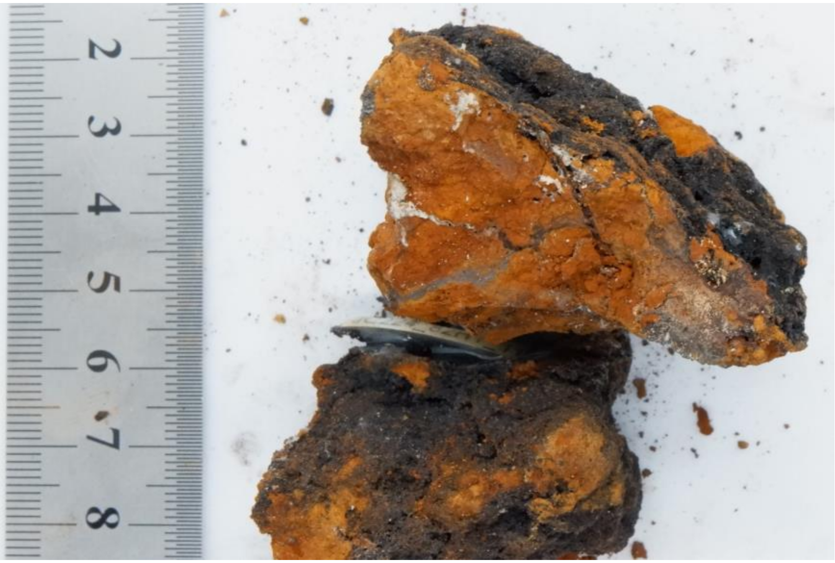

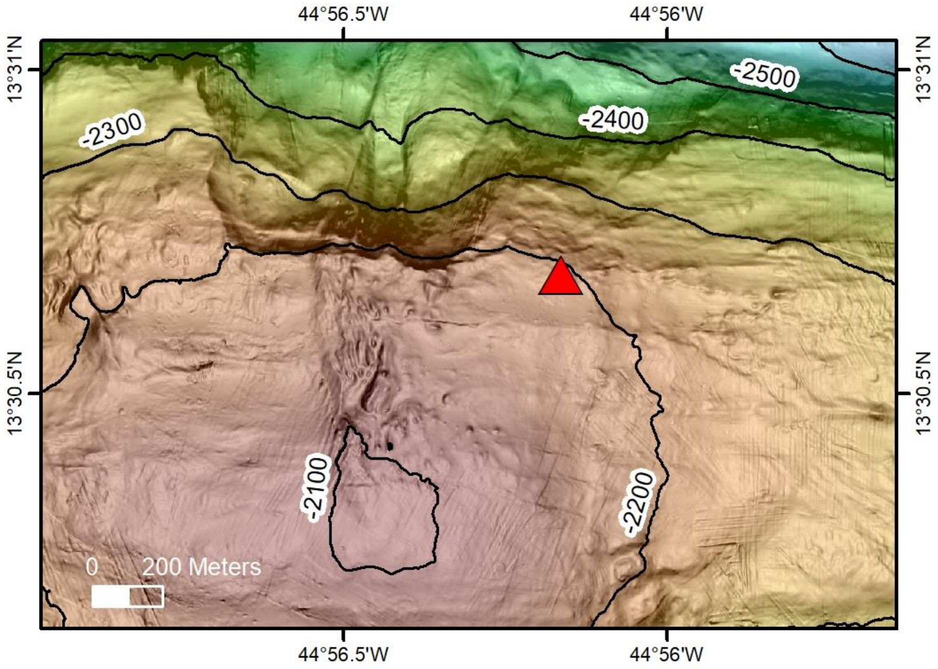

JC224_86_HY_02

Date / time (GMT)	Location	Latitude/ longitude	Water depth (m)
02/04/22; 13:40	Sem 4	13˚30.491 N<br>44˚53.089 W	2839

Description: Red oxide coated, angular fragment. Massive sulfide dominated by pyrite. Sample has a colloform texture of alternating pyrite and marcasite-rich bands. Sample has a low porosity of ~5%.

Morphology – Colloform massive sulphide

Geological association – Located on Fe-rich sediment with small areas of exposed massive sulphide talus.

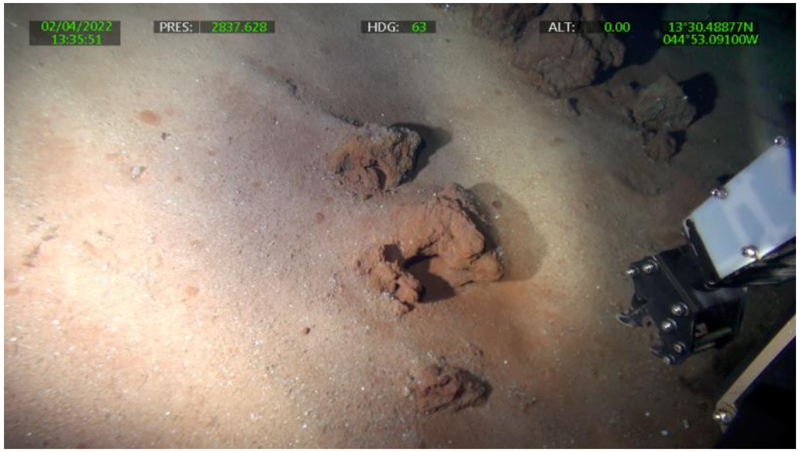

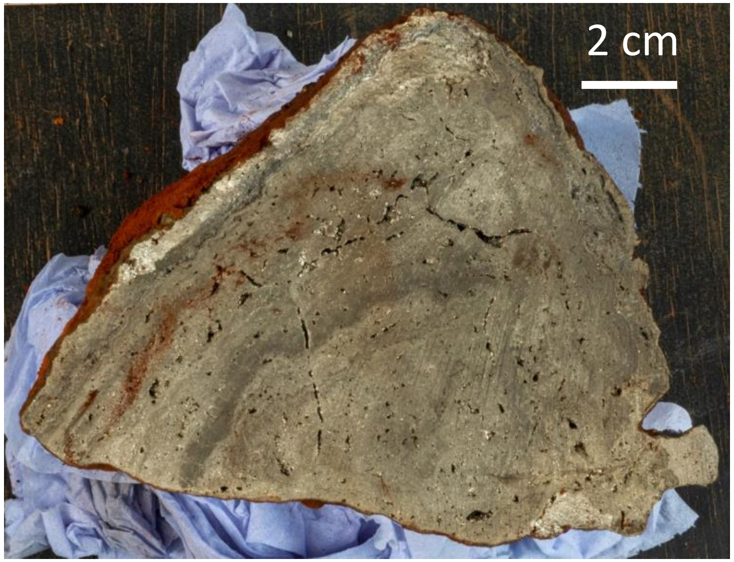

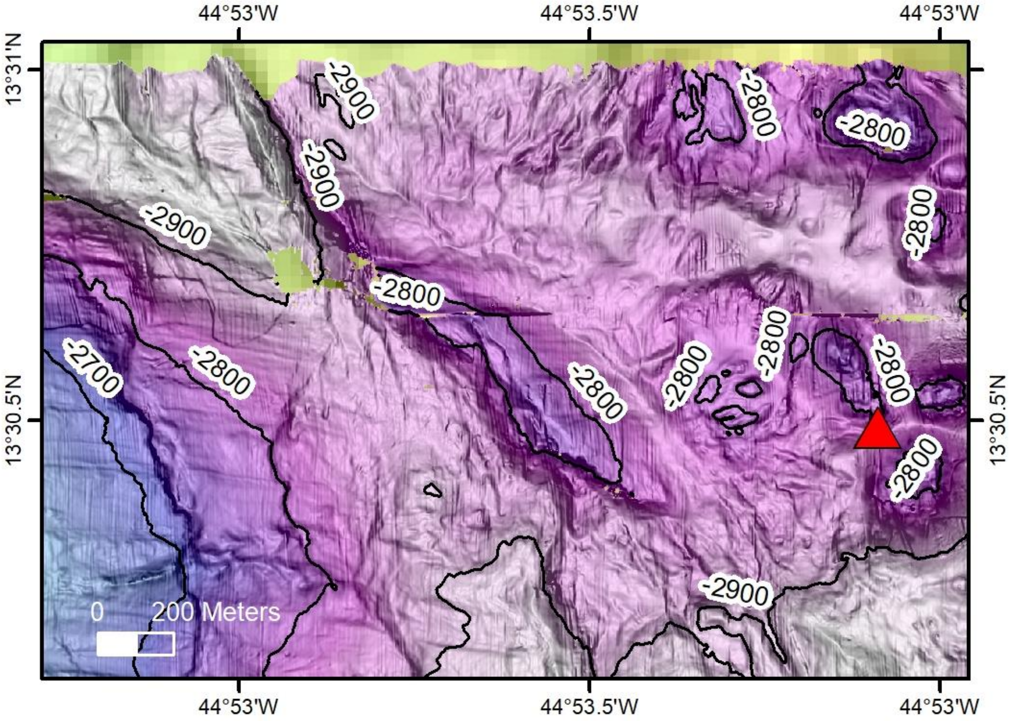

JC224_86_HY_04

Date / time (GMT)	Location	Latitude/ longitude	Water depth (m)
02/04/22; 15:07	Sem 4	13˚30.430 N<br>44˚53.031 W	2798

Description: Tabular orange piece, roughly square ~20cm. Fe-oxyhydroxide with an Fe-Mn coating.
Sample is soft and exhibits massive texture comprising of fine clay sized grains ranging from an orange red to orange yellow colour. Samples is ocherous. Sample has thin, sinuous veins composed of hematite or Fe-Mn oxide.
Morphology – Ocherous
Geological association – Located near the summit on largest hydrothermal mound in Semyenov with expose sulphide.

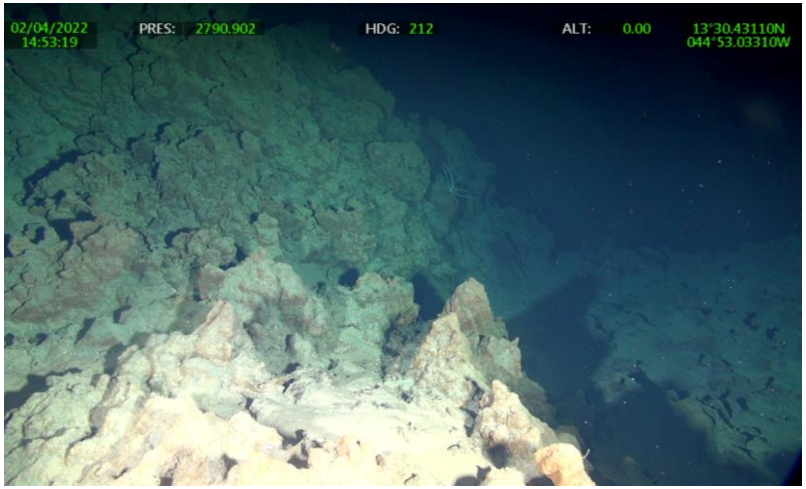

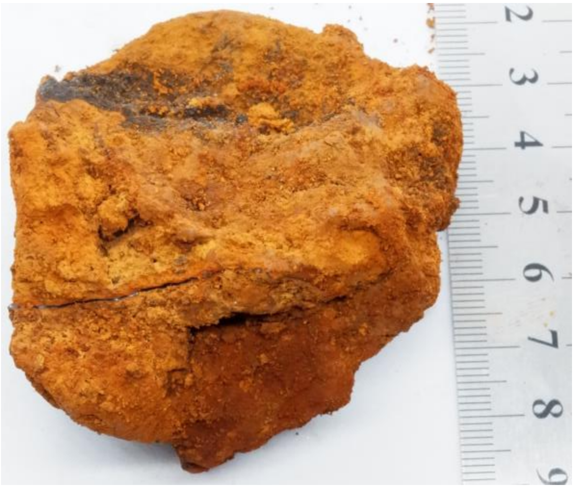

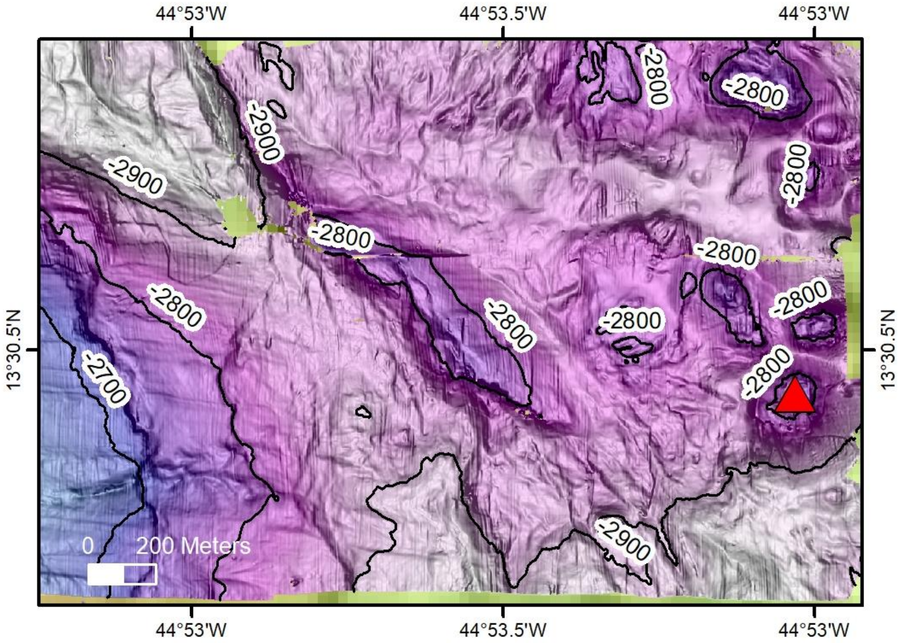

JC224_86_HY_07

Date / time (GMT)	Location	Latitude/ longitude	Water depth (m)
02/04/22; 17:29	Sem 4	13˚30.331 N<br>44˚53.196 W	2871

Description: Large, orange, triangular block. Massive sulfide dominated by pyrite with some marcasite. Tarnished areas around edge of samples show golden coloured cubic minerals inside vugs, likely tarnished pyrite (possibly chalcopyrite?). Exterior surface composed of thin Fe-oxide coating.
Morphology – Pyrite dominated massive sulphide
Geological association – Located on hydrothermal crust with exposed massive sulphide talus.

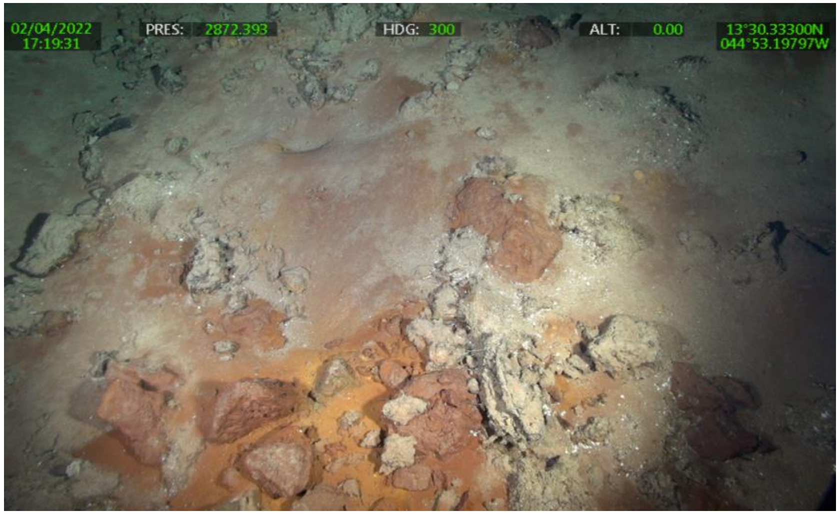

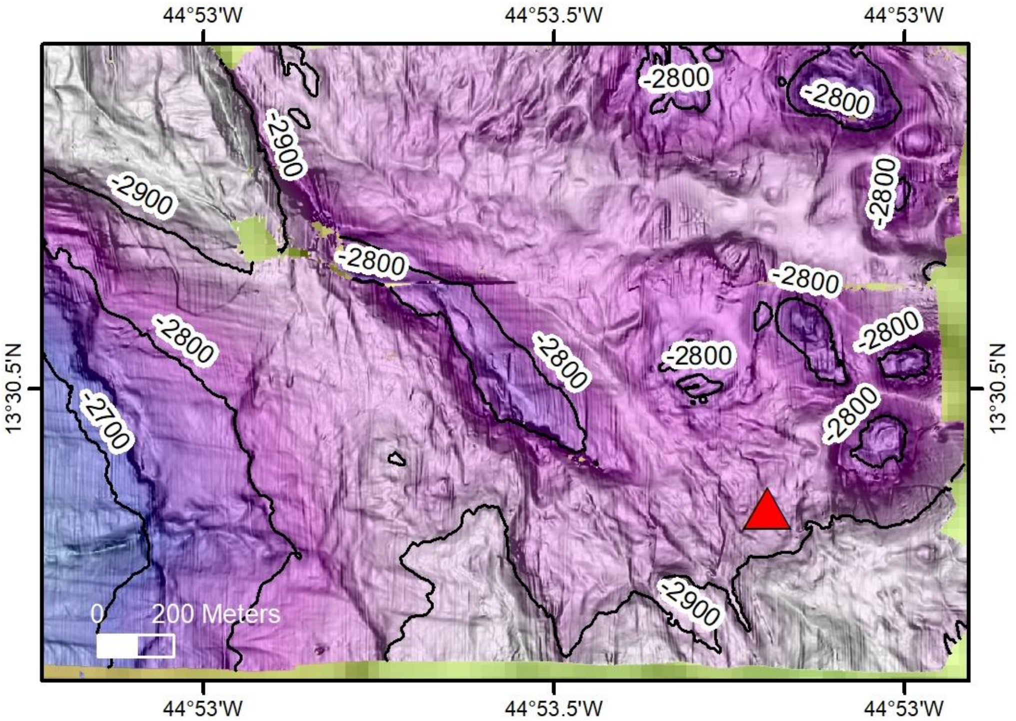

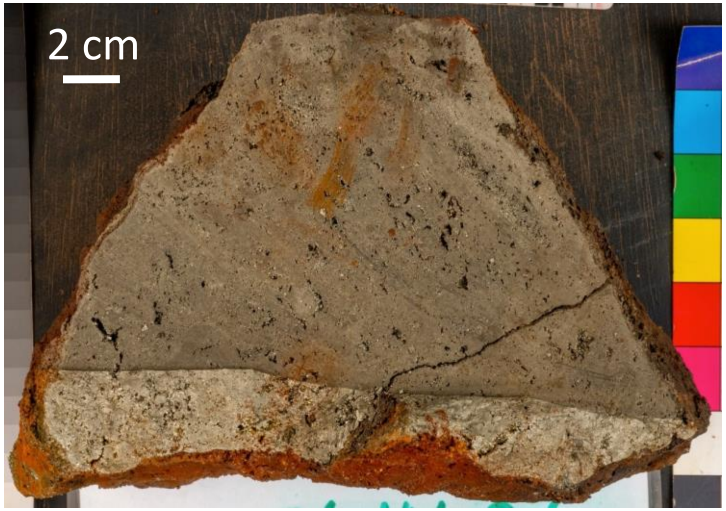

JC224_86_HY_08

Date / time (GMT)	Location	Latitude/ longitude	Water depth (m)
02/04/22; 17:34	Sem 4	13˚30.331 N<br>44˚53.196 W	2872

Description: Soft, fine grained FeOOH material ranging from dark orange red to orange red with 1 mm - <1 mm layers of Fe-Mn oxide/hematite. Sample exhibits layering of various FeOOH from darker yellowish brown to yellowish brown.

Morphology – Type II layered

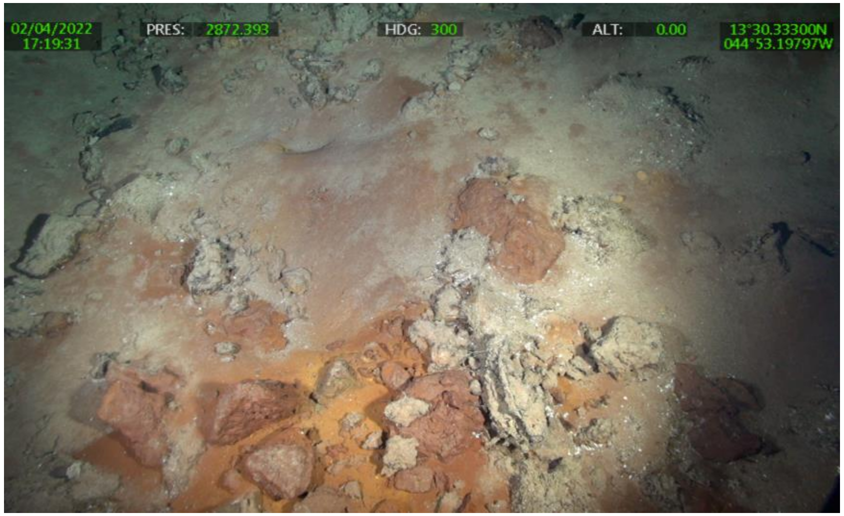

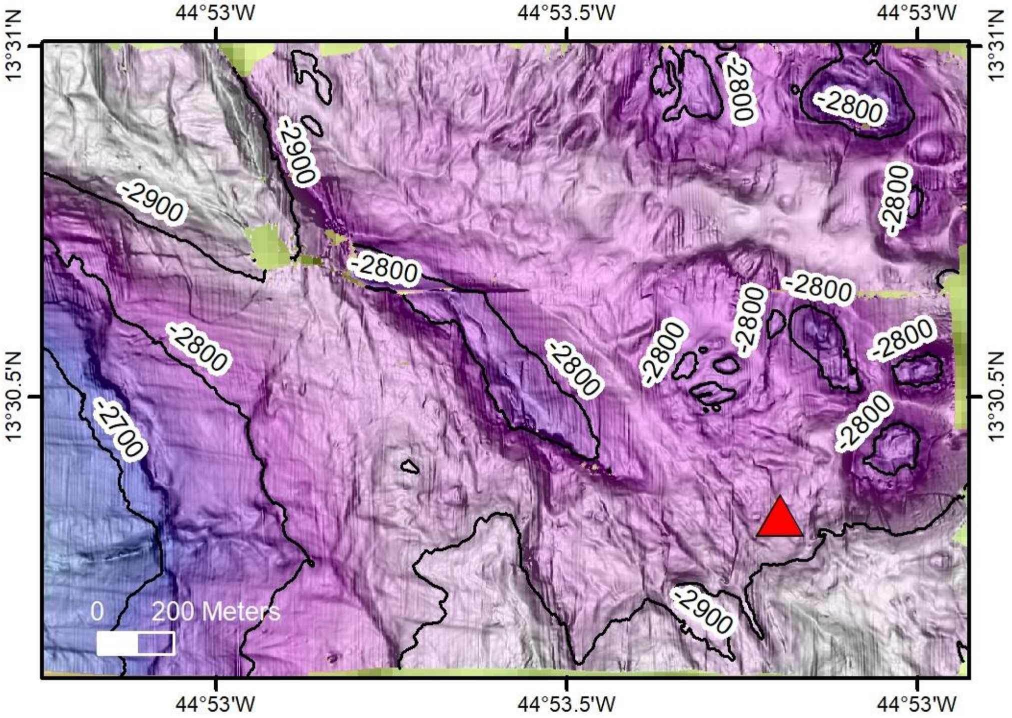

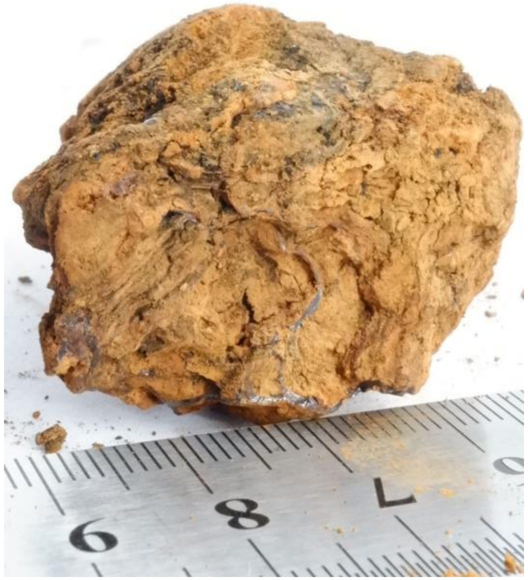

JC224_86_HY_09

Date / time (GMT)	Location	Latitude/ longitude	Water depth (m)
02/04/22; 17:44	Sem 4	13˚30.335 N<br>44˚53.208 W	2864

Description: Rounded boulder. Ochre with a thin Mn-oxide coating on exterior. Cut surface shows some disseminated barite/anhydrite?

Morphology – Type I ochre
Geological association – Located adjacent to exposed massive sulphide.

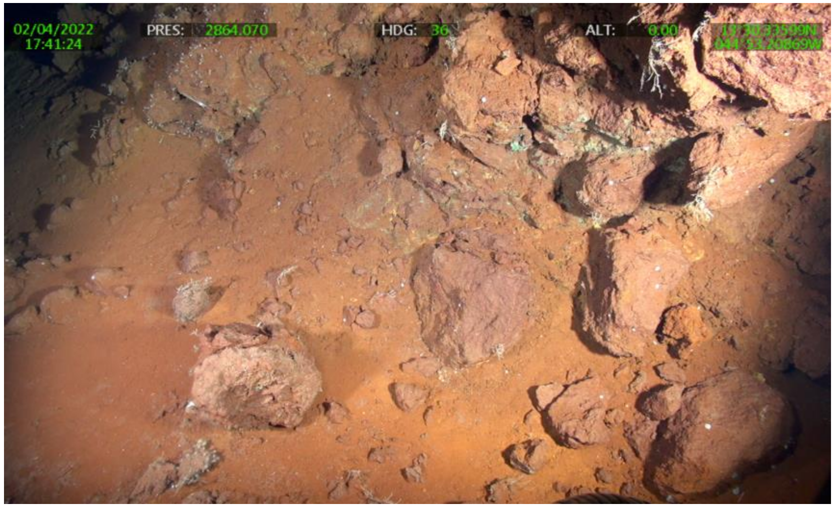

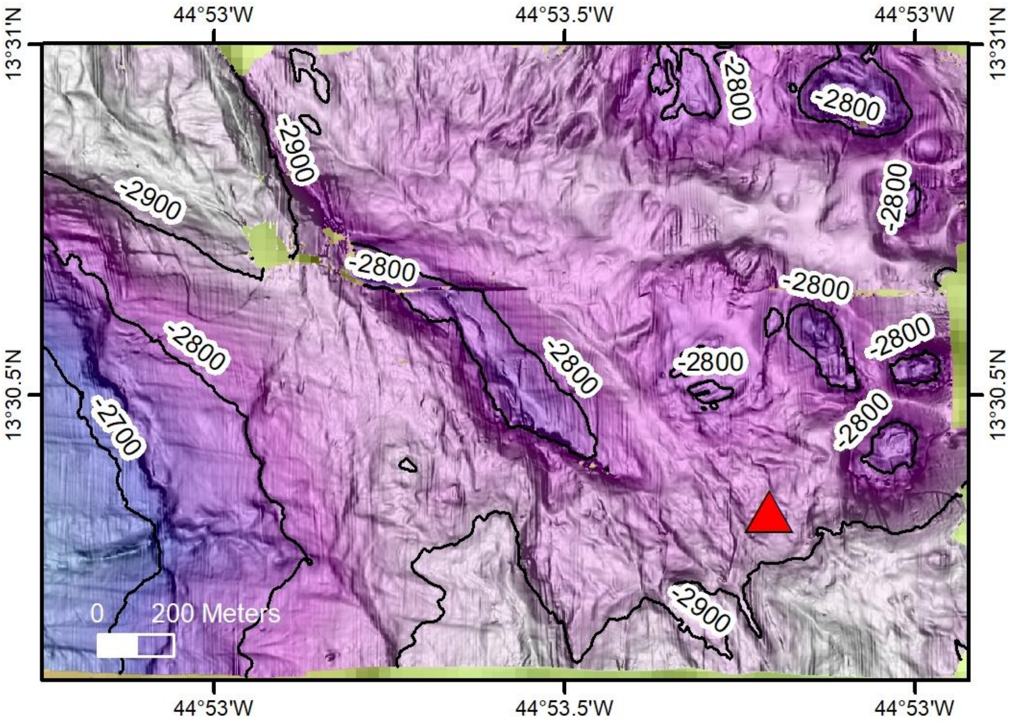

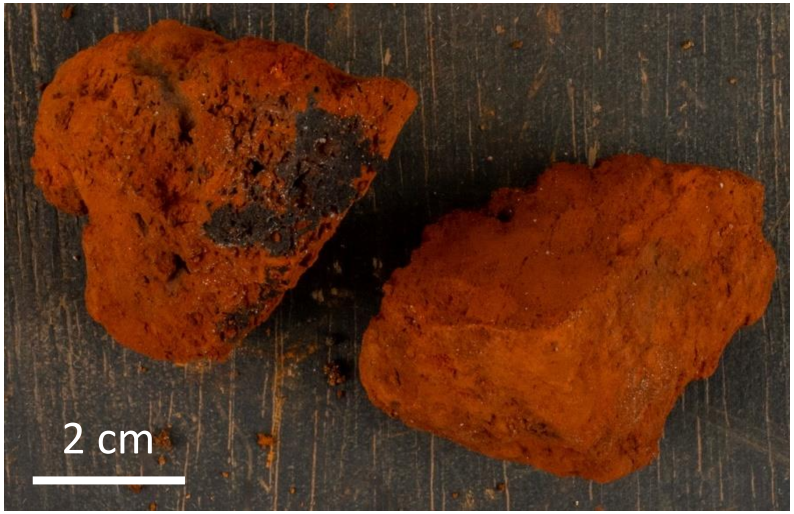

JC224_87_MC_01

Date / time (GMT)	Location	Latitude/ longitude	Water depth (m)
02/04/22, 23:31	Sem 1	13.5141833<br>-44.9908833	2568

Description: Fe-oxyhydroxide material with groundmass comprising of orange red to dark red silt-clay material. Where exposed to seawater has ~2 mm coating of Mn-O. Quite dense compared to other FeOOH material.
Morphology – Massive
Geological association – West of Sem-1 in between low lying mounds.

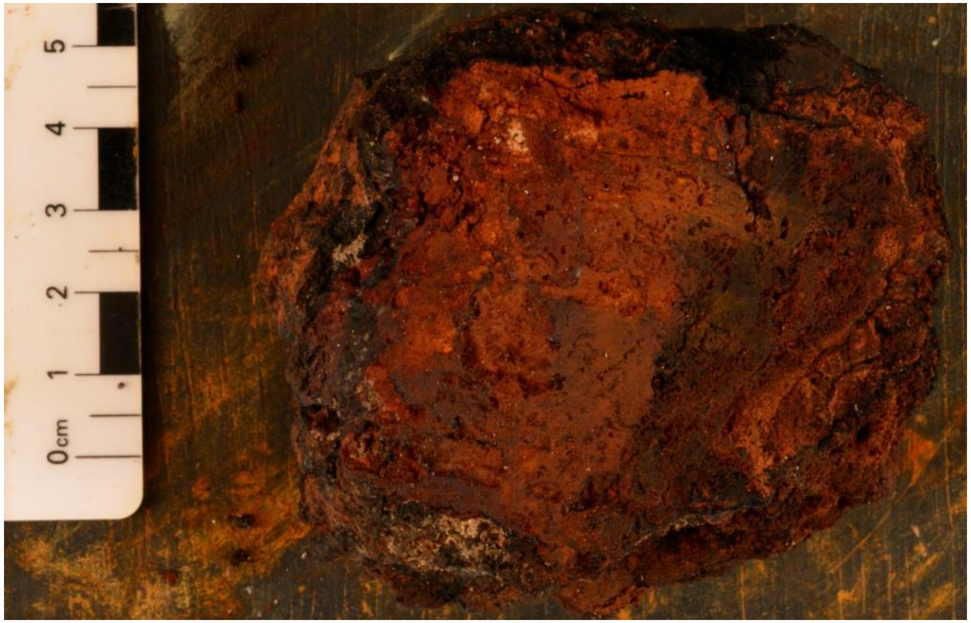

JC224_90_HY_04

Date / time (GMT)	Location	Latitude/ longitude	Water depth (m)
03/04/22; 16:50	Sem 4	13˚30.637 N<br>44˚53.106 W	2824

Description: Sample comprises of three pieces.
$90\_HY\_04_a$ (FeOOH) – Orange brown in colour with black ~! mm veins of hematite or Mn-oxide.
$90\_HY\_04_b$ (Massive sulphide) – Black in colour with flecks of barite/calcite? Mineralogy of sulphides are chalcopyrite and pyrite with sphalerite(?)
$90\_HY\_04_c$ (Weathered sulphide) – Greenish/Reddish in colour. Mineralogy of pyrite, chalcopyrite and sphalerite(?)
Morphology – Barite rich massive sulphide ($90\_HY\_04_b$ & $90\_HY\_04_c$) and massive FeOOH ($90\_HY\_04_a$)
Geological association – Found on hydrothermal mound

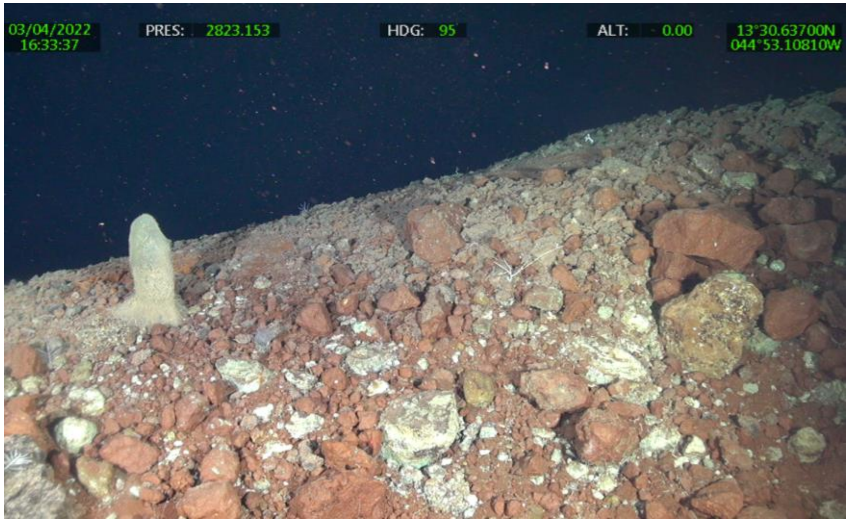

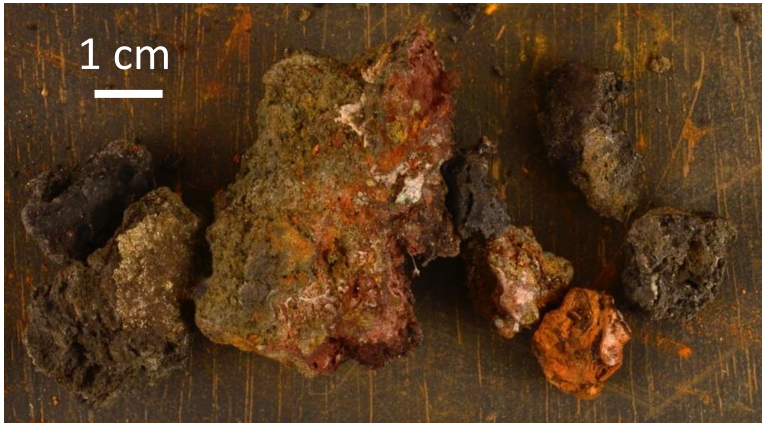

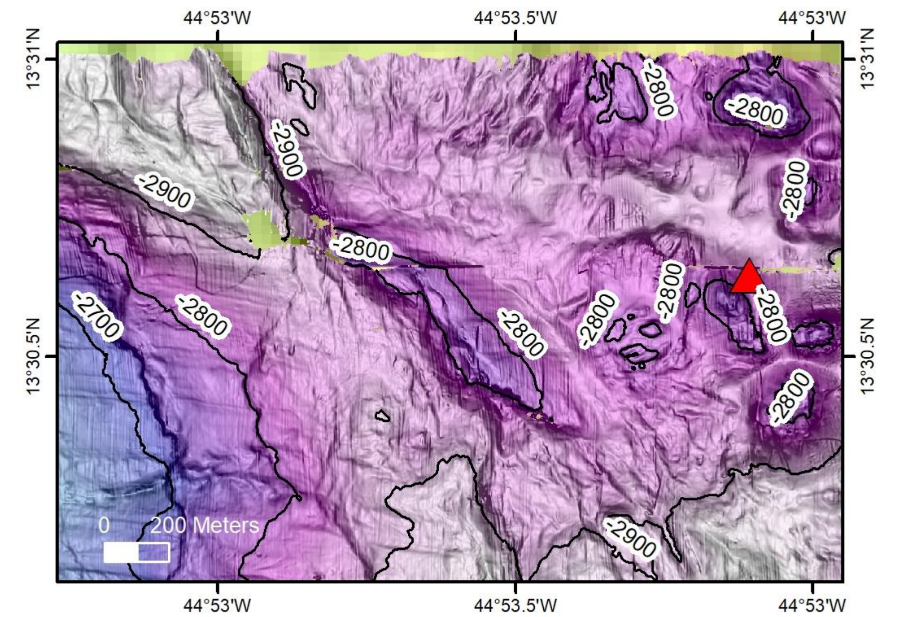

JC224_90_HY_05

Date / time (GMT)	Location	Latitude/ longitude	Water depth (m)
03/04/22; 17:33	Sem 4	13˚30.597 N<br>44˚53.157 W	2768

Description: tiny friable piece of crust. Fe-oxyhydroxide with Mn-oxide coating with veins of hematite. Veins of hematite develop a thin (<1 mm) Fe rich alteration halo.
Morphology – Massive
Geological association – Located adjacent to sulphide mound lying on hydrothermal crust.

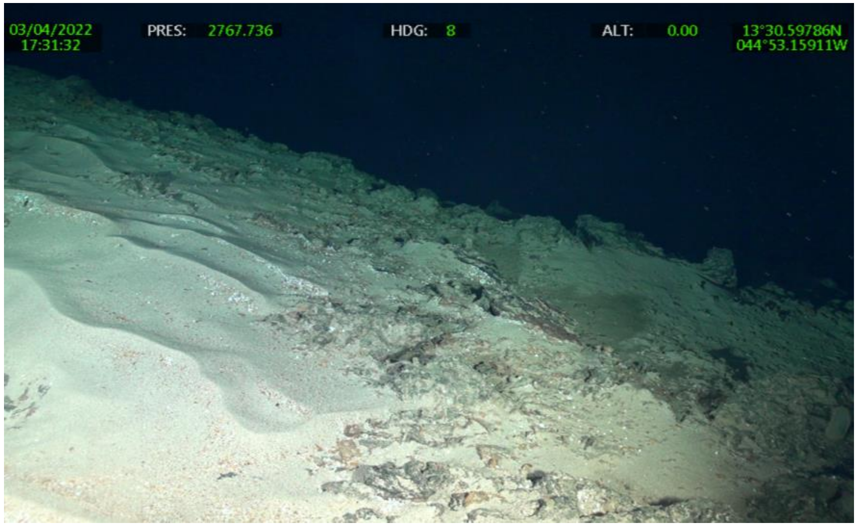

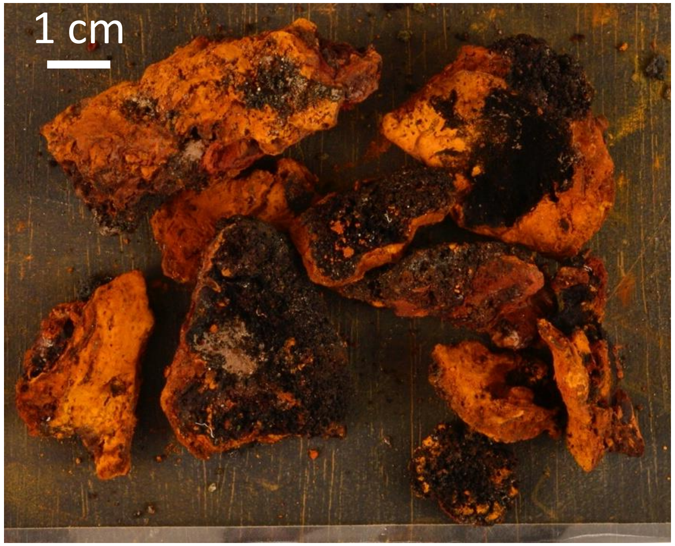

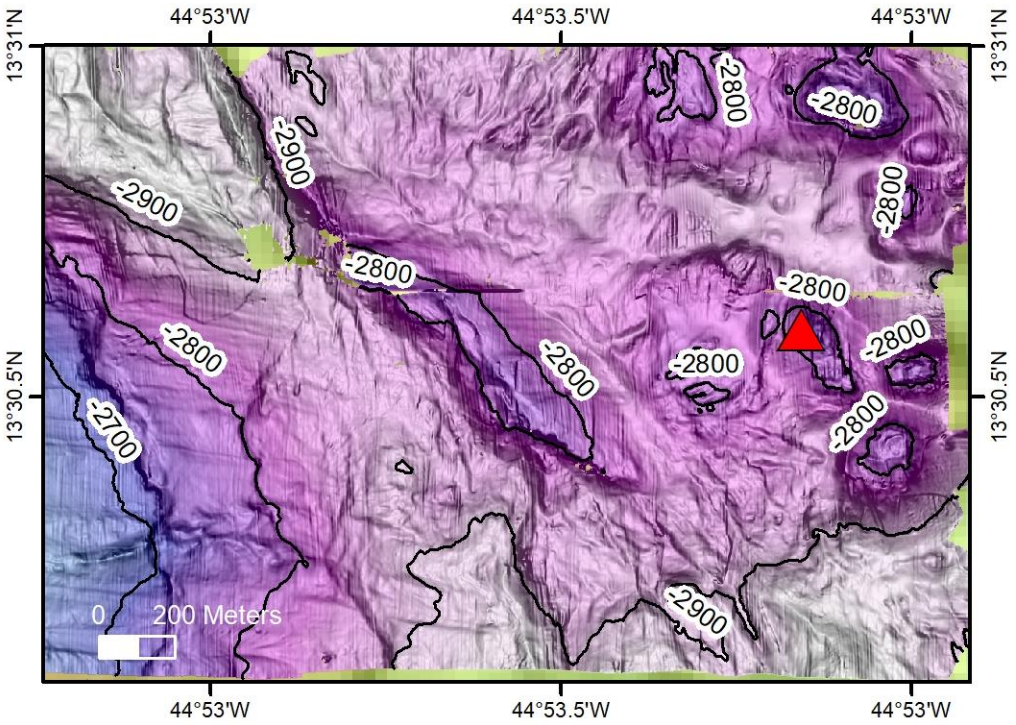

JC224_90_HY_06

Date / time (GMT)	Location	Latitude/ longitude	Water depth (m)
03/04/22; 18:38	Sem 4	13˚30.535 N<br>44˚53.248 W	2817

Description: long angular piece Fe-Mn crust, with some orange staining. Fe-oxyhydroxide with thin (<1 mm) hematite veins and a thin Mn-oxide coating. Hematite forms a ring on the cut surface where it is hollow and filled with pelagic sediment.

Morphology – Massive

Geological association – Located ontop of hydrothermal crust adjacent to hydrothermal crust and sulphide talus.

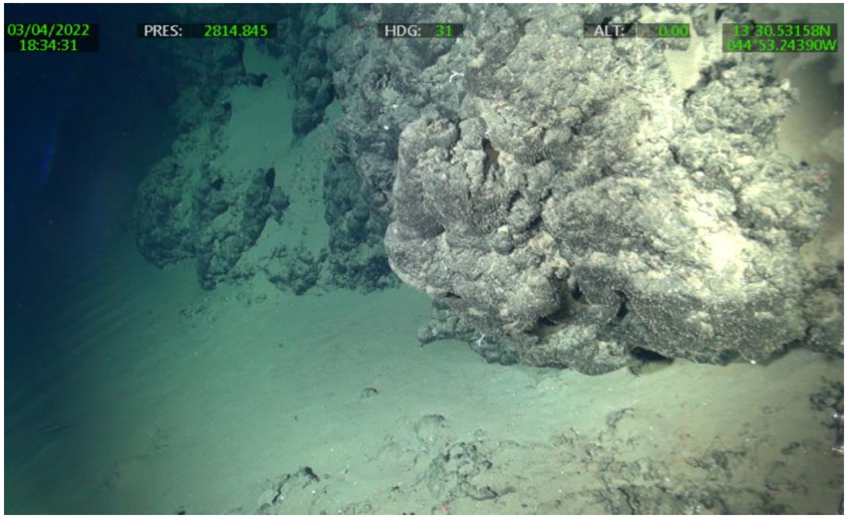

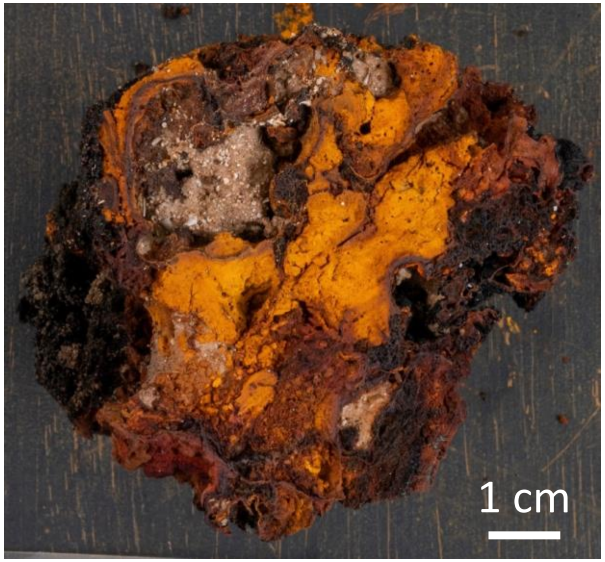

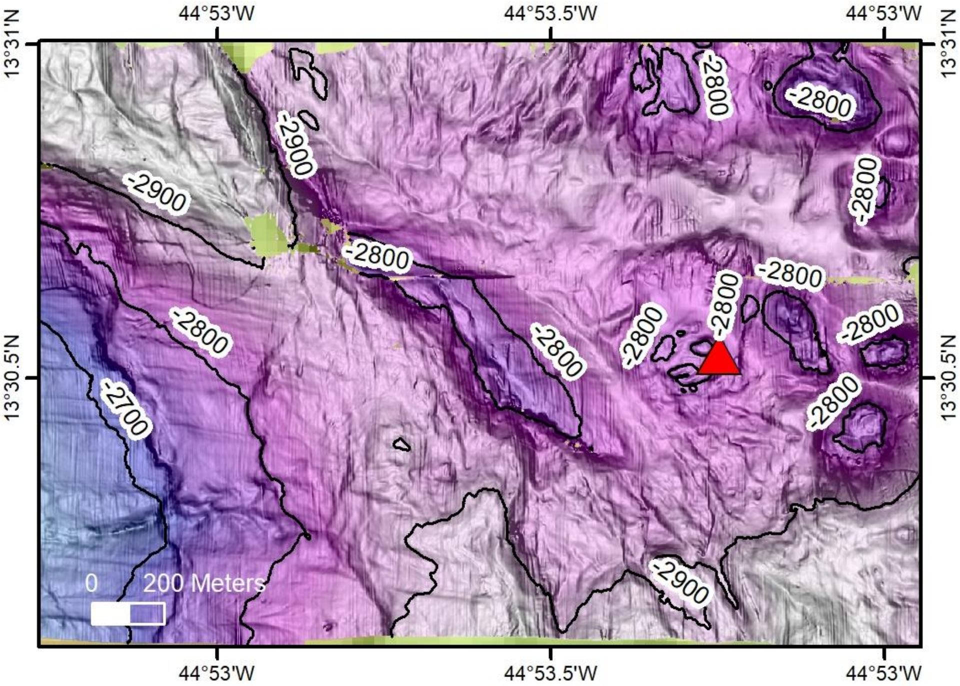

JC224_94_HY_01

Date / time (GMT)	Location	Latitude/ longitude	Water depth (m)
04/04/22; 13:11	Sem 4	13˚30.206 N<br>44˚53.748 W	2887

Description: crusty piece of small, friable, black outside orange interior FeOOH. Assorted pieces of Fe-oxyhydroxide transitioning to ochre. Thin Mn oxide coating on some surfaces. Interior is fine grained, homogenous and porous.
Morphology – Type I ochre
Geological association – Hydrothermal crust with basalt talus.

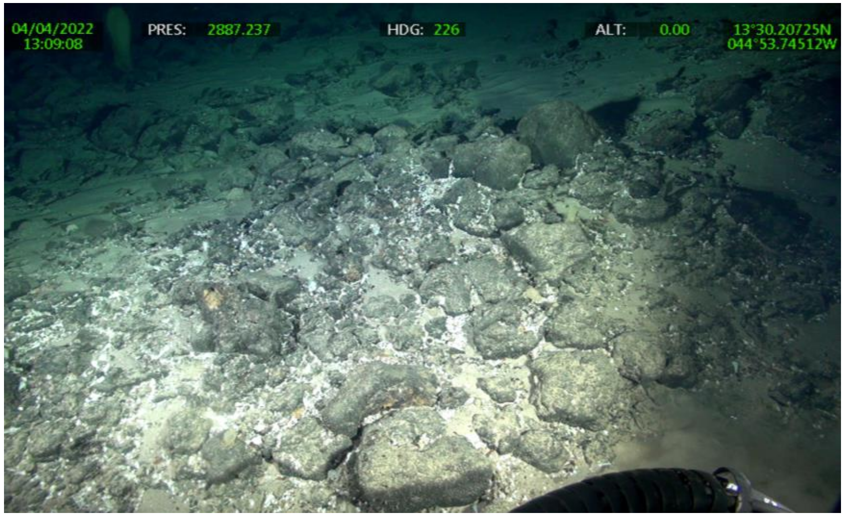

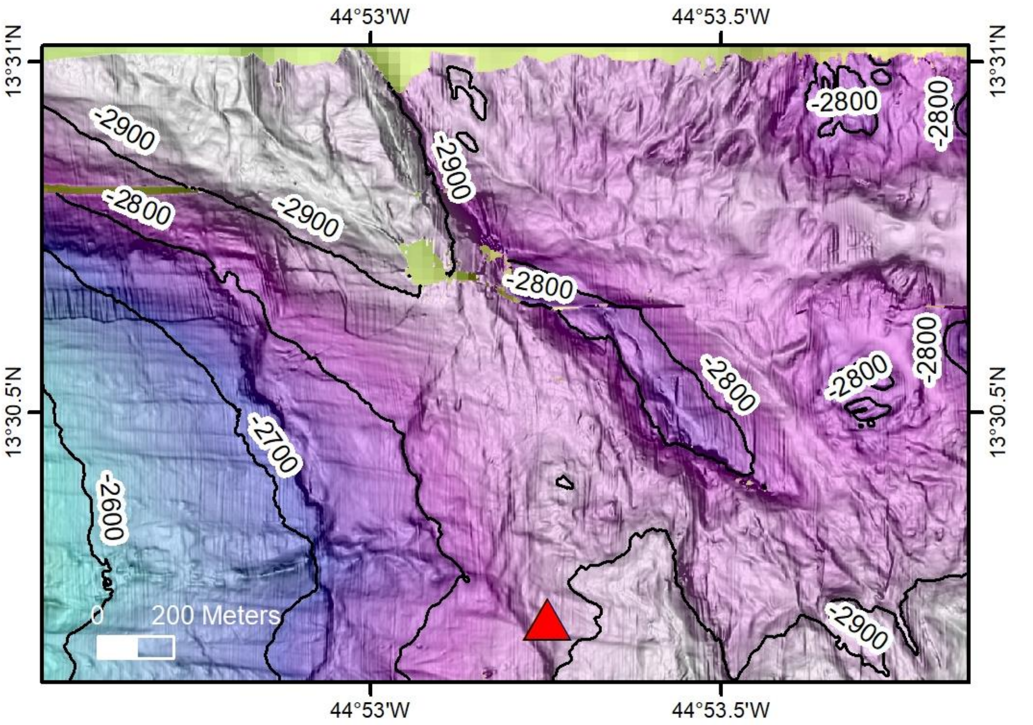

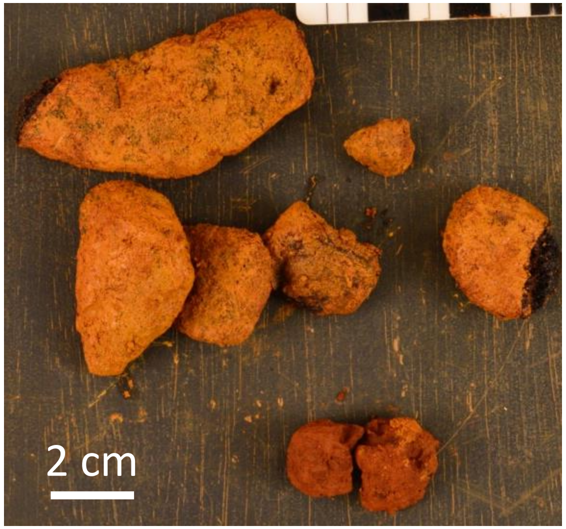

JC224_94_HY_04

Date / time (GMT)	Location	Latitude/ longitude	Water depth (m)
04/04/22; 14:01	Sem 4	13˚30.219 N<br>44˚53.821 W	2841

Description: Soft, fine grained ochre material orange red in colour. Black Fe-Mn oxide crust has developed on surface exposed to seawater. Rare 1 mm Fe-Mn oxide/hematite veins. Groundmass comprises of FeOOH material with some transparent, vitreous crystals that could be barite or quartz. Groundmass is massive in texture.

Geological association – Found in hydrothermal crust ~5-10m west of massive sulphide.

Morphology - Type I ochre

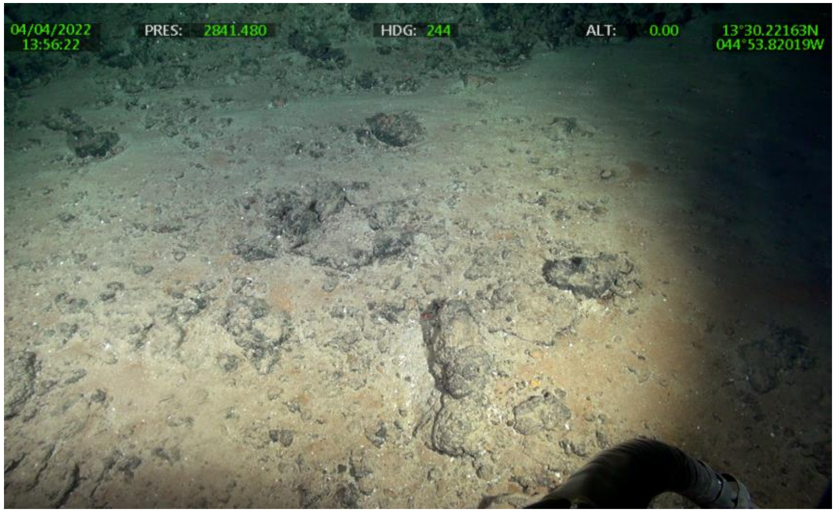

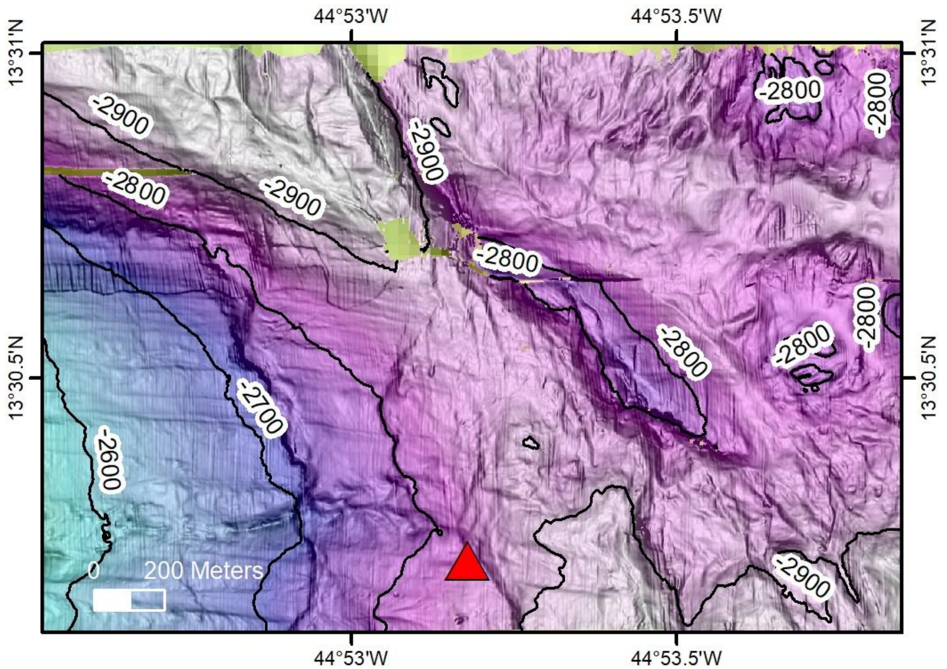

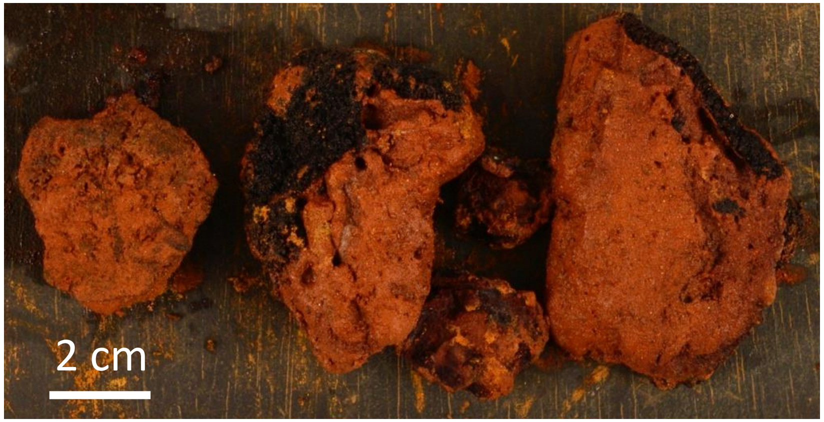

JC224_94_HY_05

Date / time (GMT)	Location	Latitude/ longitude	Water depth (m)
04/04/22; 14:20	Sem 4	13˚30.237 N<br>44˚53.848 W	2822

Description: Surface exposed to seawater comprises of black Fe-Mn crust. Sample is significantly more dense compared to previous FeOOH samples and is harder as a result. Sample exhibits chaotic layering of brownish red to yellowish orange. Trace atacamite observed as green specks on sample.
Morphology – Type II layered
Geological association – Found ontop of hydrothermal crust. No massive sulphide mound in close proximity. Closest massive sulphide material is ~65 m north west and east.

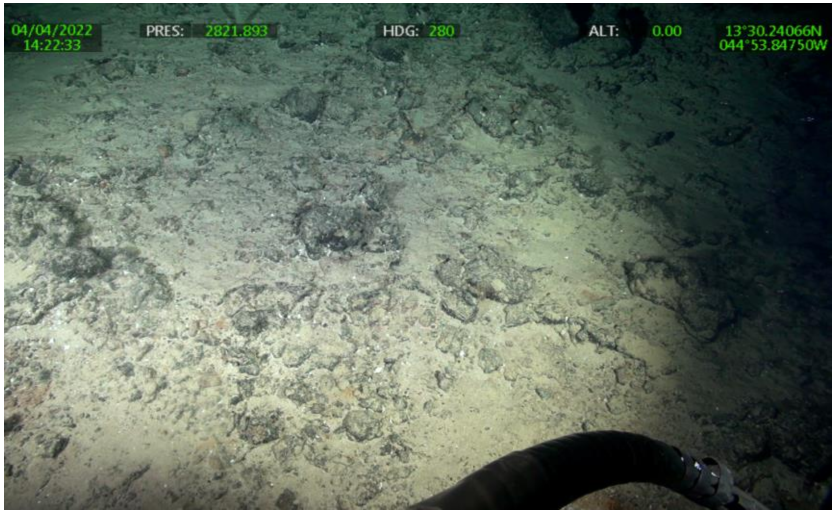

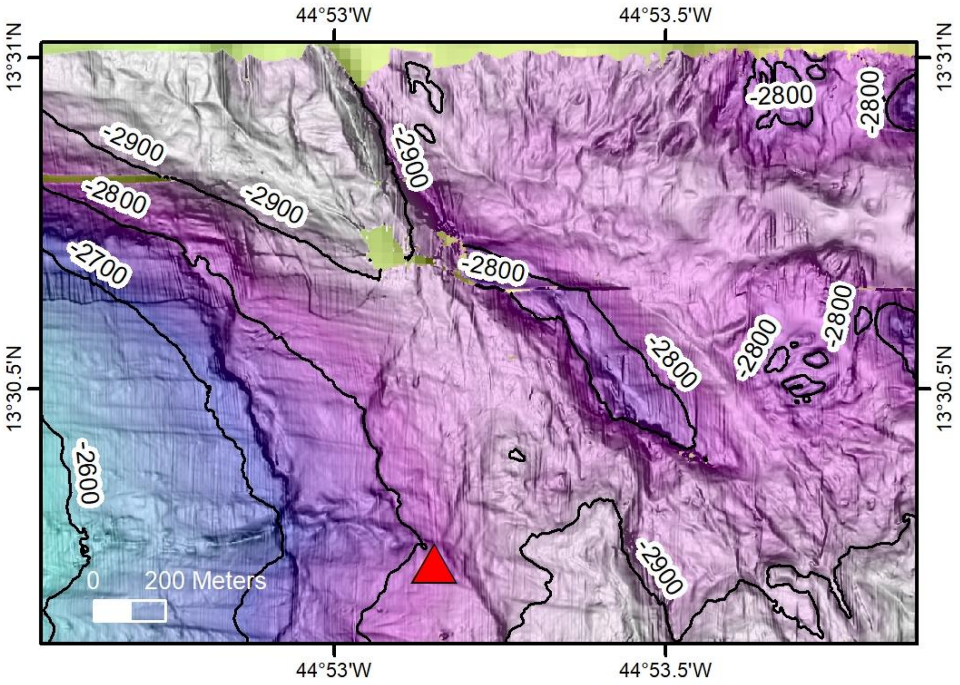

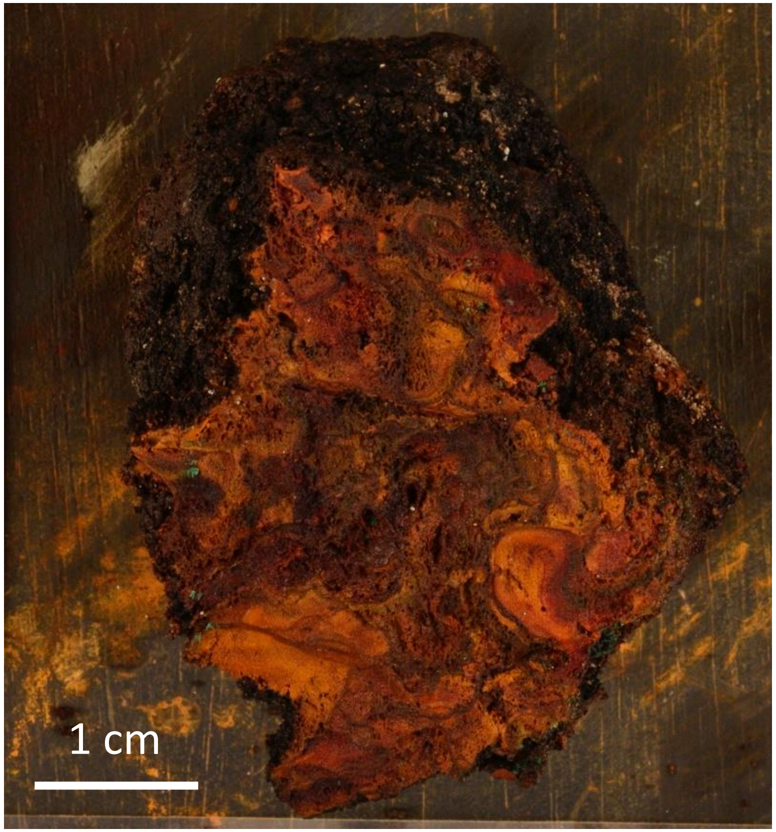

JC224_94_HY_06

Date / time (GMT)	Location	Latitude/ longitude	Water depth (m)
04/04/22; 15:49	Sem 4	13˚30.336 N 44˚53.695 W	2865

Description: Relatively firm, orange red to brownish yellow FeOOH. Sample comprises of ocherous texture with silt – clay sized grains of FeOOH.. Veins of Fe-Mn oxide/hematite are present and occur as ~1 mm thick veins. Cut sample shows chaotic layering of the darker and pale coloured FeOOH. Texture is overall unclear on morphology. The outside of the sample comprises of thin Fe-Mn oxide crust and abundant atacamite present on the sample.
Morphology – Type II layered
Geological association – Located on hydrothermal mound with hydrothermal crust and massive sulphide talus

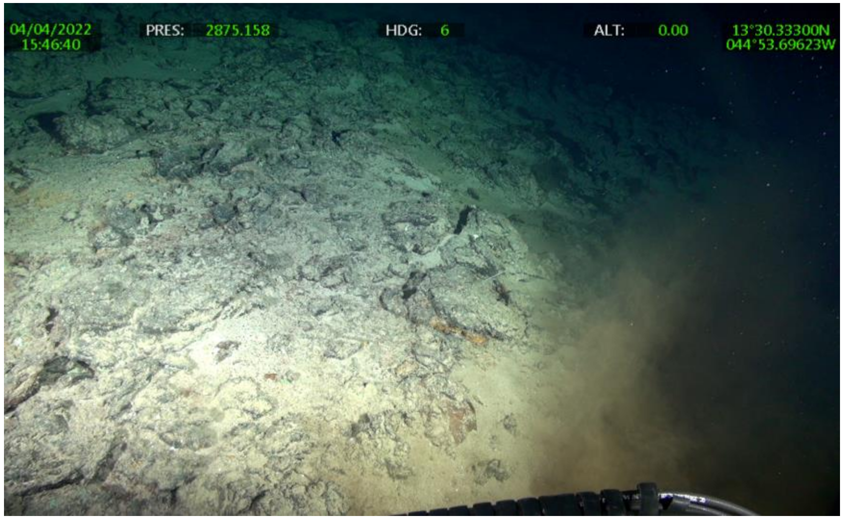

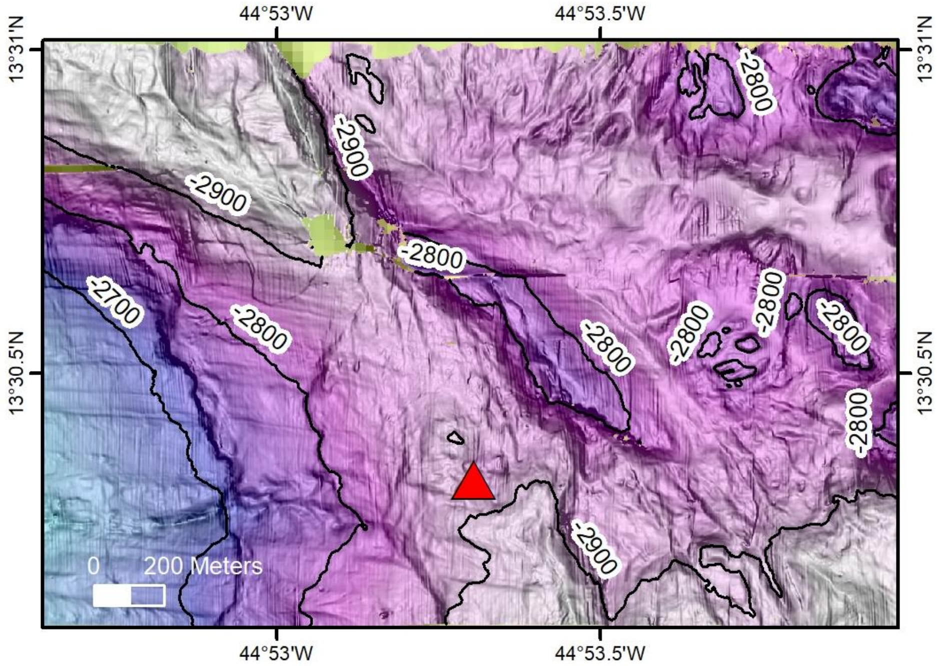

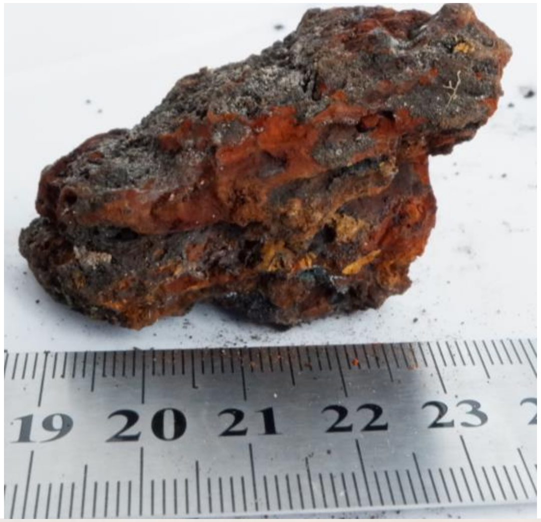

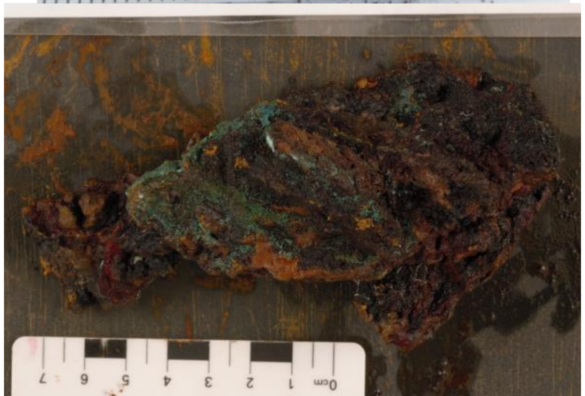

JC224_102_HY_03

Date / time (GMT)	Location	Latitude/ longitude	Water depth (m)
06/04/22; 13:28	Sem 4	13˚xx.xxx N<br>44˚xx.xxx W	2779

Description: Sample comprises of brownish red to reddish orange with sample soft in the lighter areas. Black, relatively harder Mn-oxide (?) forms as a crust ontop of the sample where exposed to seawater . FeOOH in the middle exhibits chaotic layering.

Morphology – Type II layered

Geological association – Sample obtained ontop of hydrothermal crust located adjacent to sulphide hydrothermal mound.

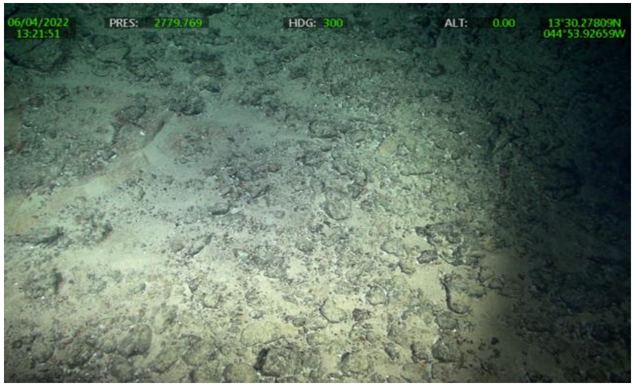

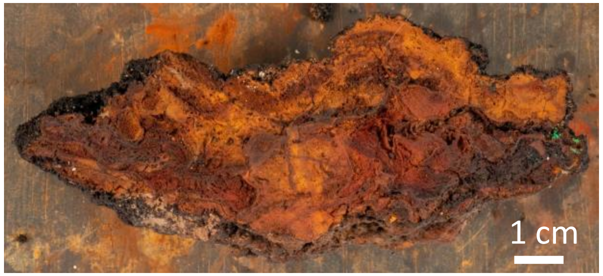

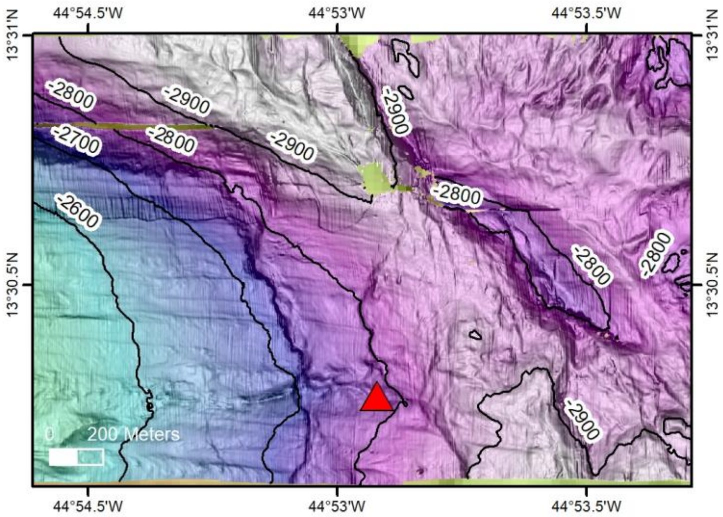

JC224_102_HY_04

Date / time (GMT)	Location	Latitude/ longitude	Water depth (m)
06/04/22; 14:20	Sem 4	13˚30.301 N<br>44˚59.015 W	2740

Description: Red to orange red and black relatively firm Fe-oxyhydroxide. Sample shows an obvious hydrothermal chimney structure with thin veins of hematite(?)/Mn-oxide(?) <1mm running parallel to the hydrothermal chimney. Odd circular structures found at bottom of sample and could represent attachment to substrate or encased hydrothermal chimneys.
Morphology – Type II chimney
Geological association – Located ontop of hydrothermal crust ~25m from closest sulphide mound. Bottom features clearly show hydrothermal crust and no obvious signs sulphide. Geographically is located on a hill which could possibly be a hydrothermal mound.

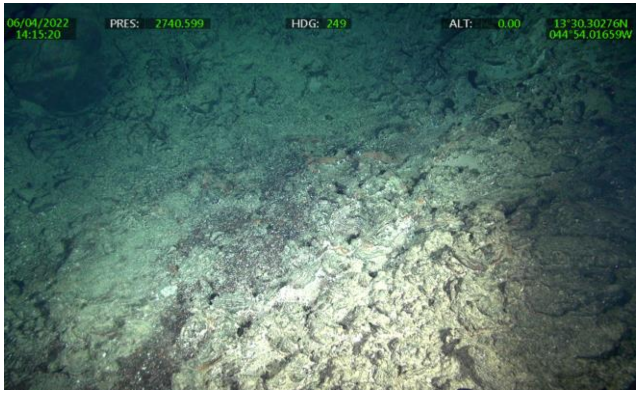

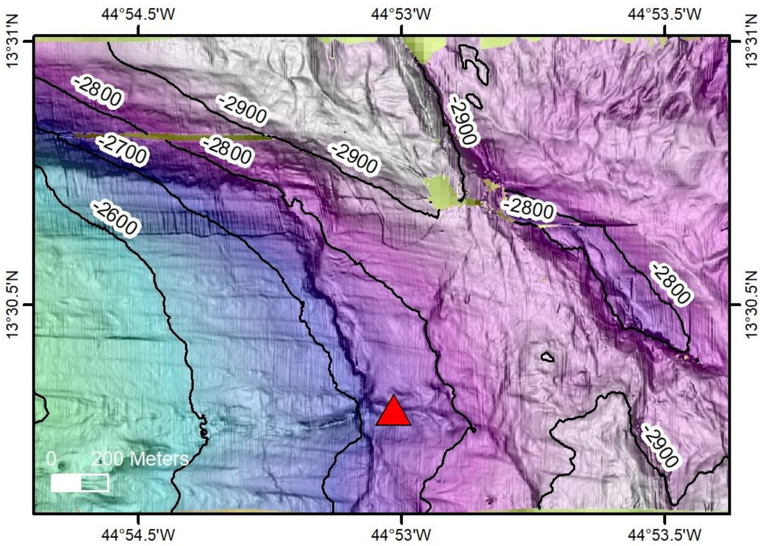

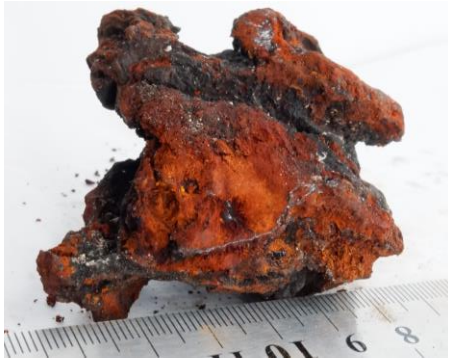

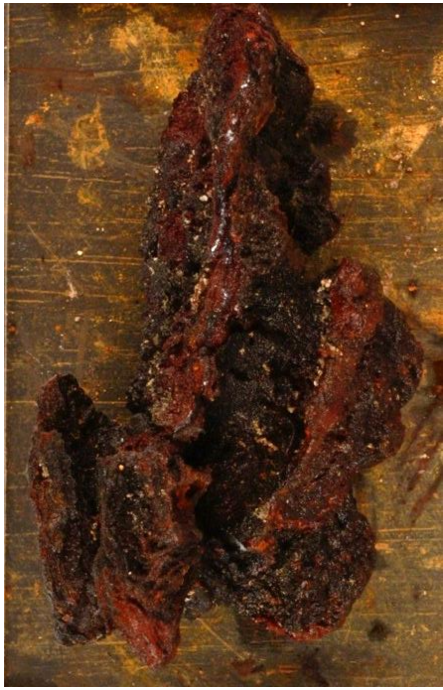

JC224_102_HY_05

Date / time (GMT)	Location	Latitude/ longitude	Water depth (m)
06/04/22; 14:32	Sem 4	13˚30.293 N<br>44˚54.033 W	2725

Description: Sample is relatively hard for FeOOH, exhibits a orangish red to red colour with veins of black ~1 mm Fe-Mn oxide/hematite. Cut surface shows chaotic layering. Whole sample shows an obvious chimney like structure with conformable layering of FeOOH material.
Odd circular structures at the base of sample similar to 102_HY_04. These structures run perpendicular to the chimney, so may have been offshoots of the chimney?
Morphology – Type II Chimney
Geological association Obtained on hydrothermal mound with sulphide talus

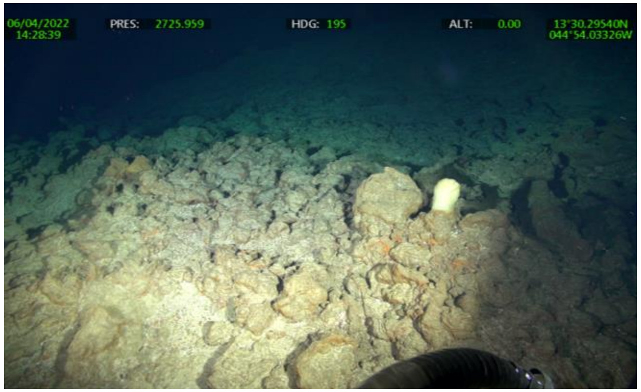

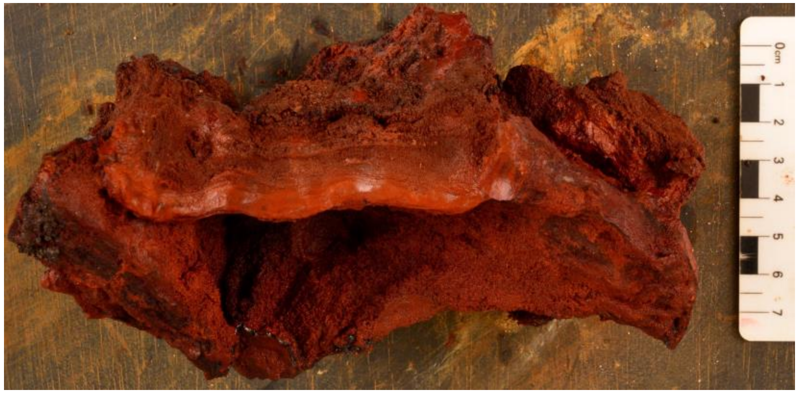

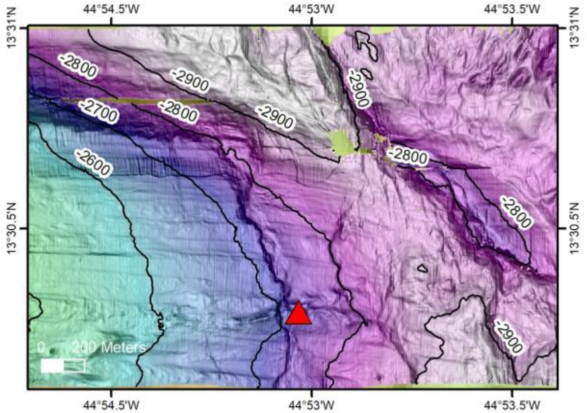

JC224_102_HY_06

Date / time (GMT)	Location	Latitude/ longitude	Water depth (m)
06/04/22; 14:59	Sem 4	13˚30.285 N 44˚59.059 W	2717

Description: thin, Fe-Mn crust, dark, elongated ~10 cm x 3 cm x 2 cm. Fe-Mn oxide crust and Fe-oxyhydroxide. Sample is highly porous, Fe-rich alteration of primary material (volcanogenic or sulfide?) covered by 2-3 mm Fe-Mn oxide rim with botryoids. Veins of crystalline dark grey hematite/Mn - oxide cross cutting the groundmass.
Morphology – Massive
Geological association – Found on hydrothermal crust close to hydrothermal mounds with massive sulphide

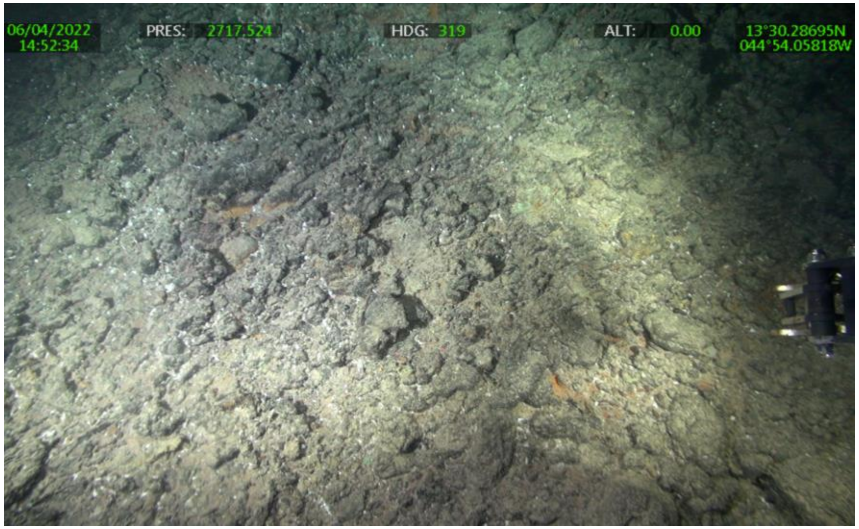

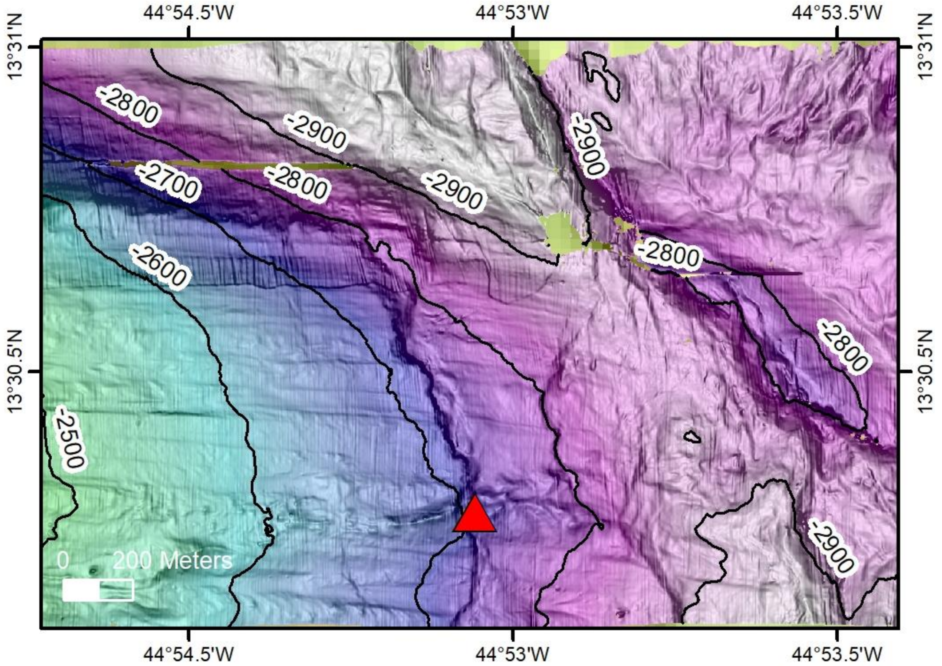

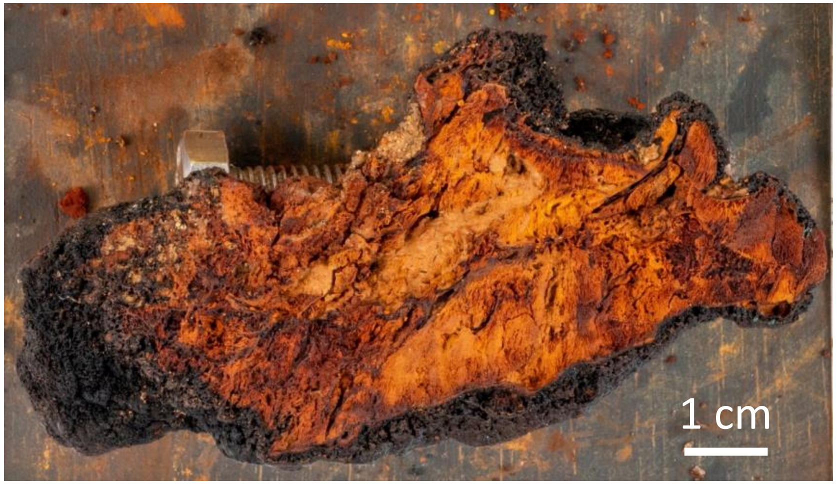

JC224_102_HY_07

Date / time (GMT)	Location	Latitude/ longitude	Water depth (m)
06/04/22; 15:01	Sem 4	13˚30.285 N<br>44˚59.070 W	2713

Description: Fe-oxyhydroxide, reddish, highly porous and altered clast(s) cemented by black oxides and veins of grey, crystalline hematite/Mn oxide. Samples is coated by 0.1-2 cm of Fe-Mn oxide. The FeOOH material is soft.

Morphology – Massive

Geological association – Obtained on hydrothermal crust close to hydrothermal mounds with massive sulphide

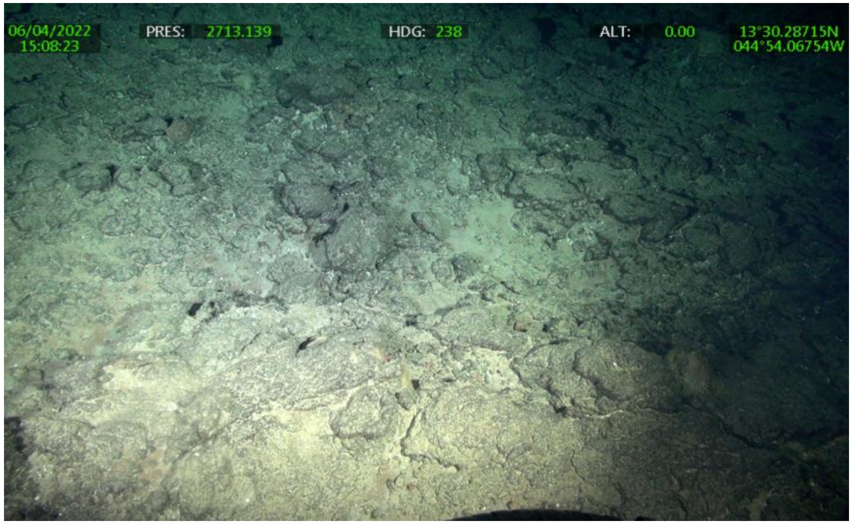

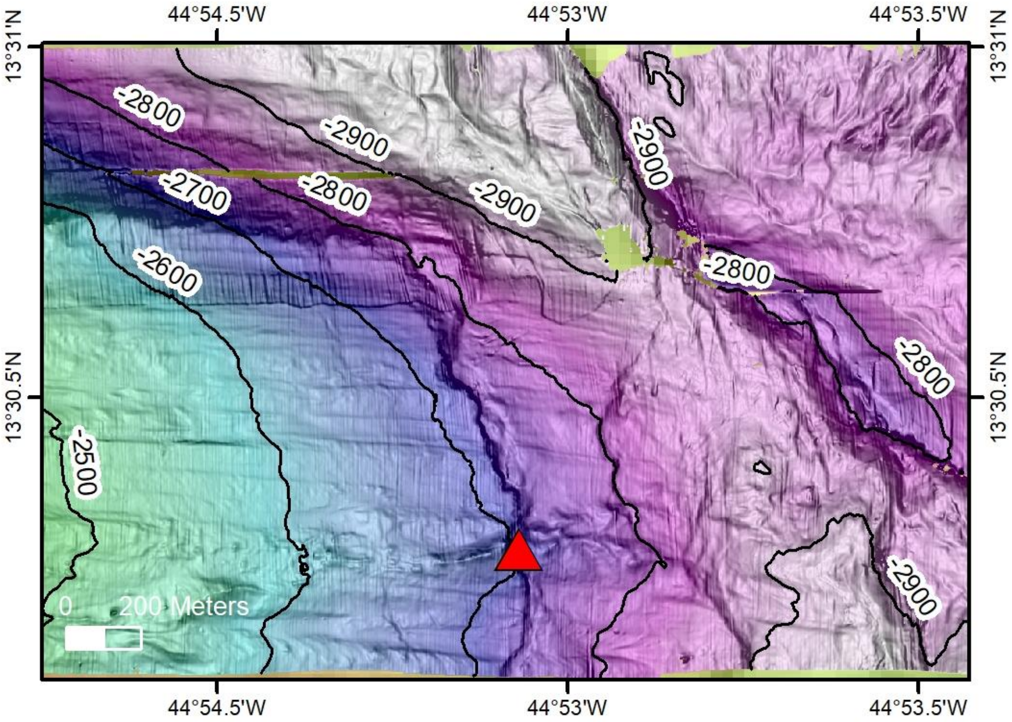

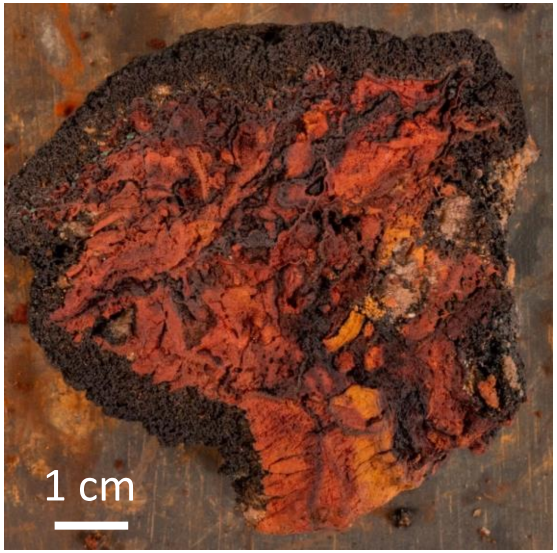

Supplement: Supplementary file 1 — Supplementary Material 1 (PDF 9.65 MB) [file 126_2025_1376_MOESM1_ESM.pdf]
