# Supplementary material for: Fe-oxyhydroxide deposits at Semenov hydrothermal field (13°30′N), Mid-Atlantic ridge: insights into formation, modification and resource potential
Source: Miner Depos. 2025 Aug 1;61(2):257–79. doi: 10.1007/s00126-025-01376-6 (PMC12858624; doi:10.1007/s00126-025-01376-6)
Supplement: Supplementary file 2 — Supplementary Material 2 (PDF 1.06 MB) [file 126_2025_1376_MOESM2_ESM.pdf]

Fe-oxyhydroxide deposits at Semenov Hydrothermal Field (13°30′N), Mid-Atlantic Ridge: insights into formation, modification and resource potential

Christian Bishop[1], Anna Lichtschlag[2], Stephen Roberts[1], Maxime Lesage[3] & Bramley J. Murton[2]

[1]School of Ocean and Earth Science, University of Southampton, Southampton, UK,

[2]National Oceanography Centre, Southampton, UK,

[3]Nedre Slottsgate 8 - 0157 Oslo, Norway

csb1u21@soton.ac.uk & chris.star.bish@gmail.com

Electronic supplementary materials 3 - XRD results

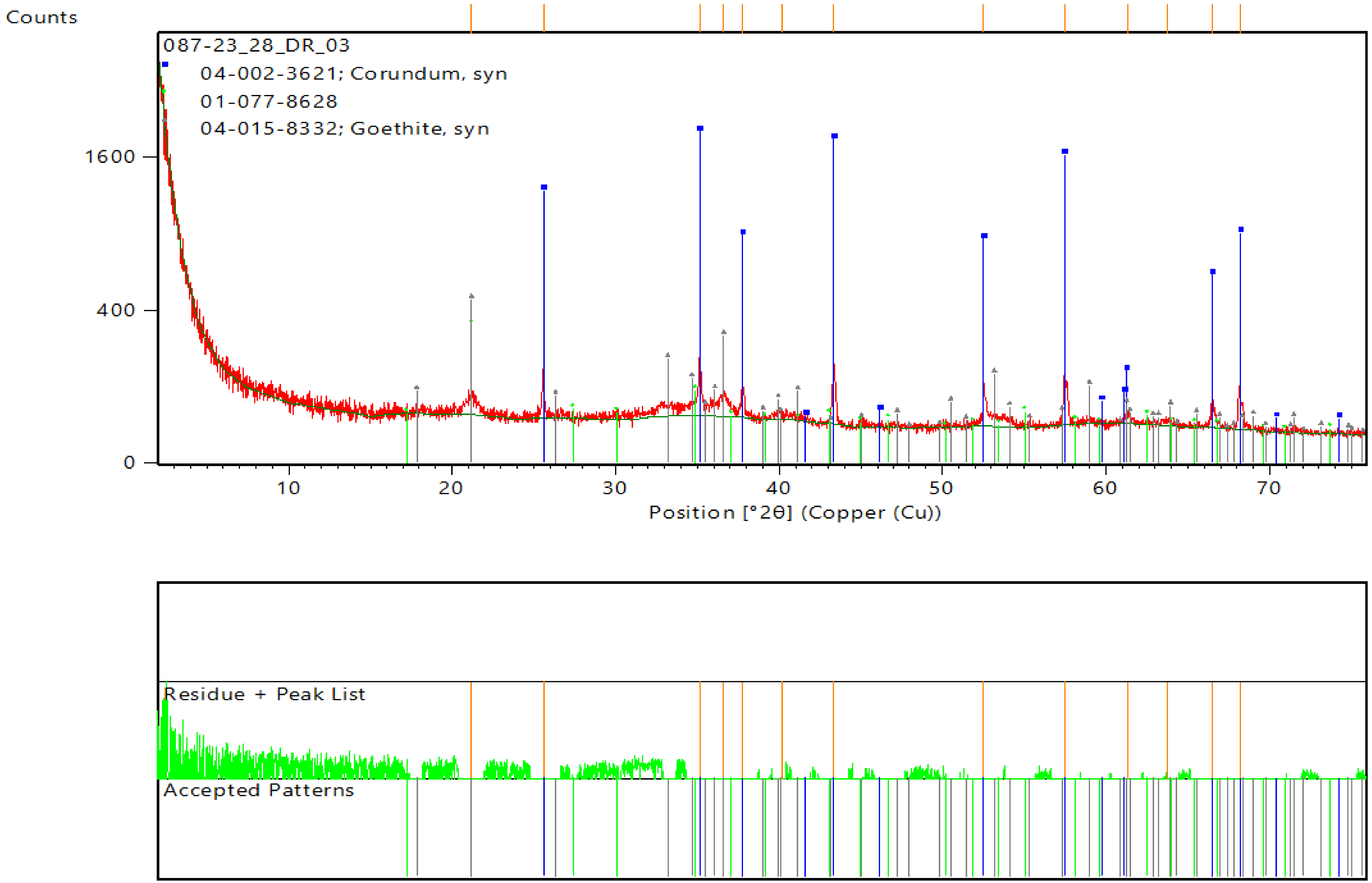
Counts
087-23_28_DR_03
04-002-3621; Corundum, syn
01-077-8628
04-015-8332; Goethite, syn
1600
400
0
10
20
30
40
50
60
70
Position [°2θ] (Copper (Cu))
Residue + Peak List
Accepted Patterns

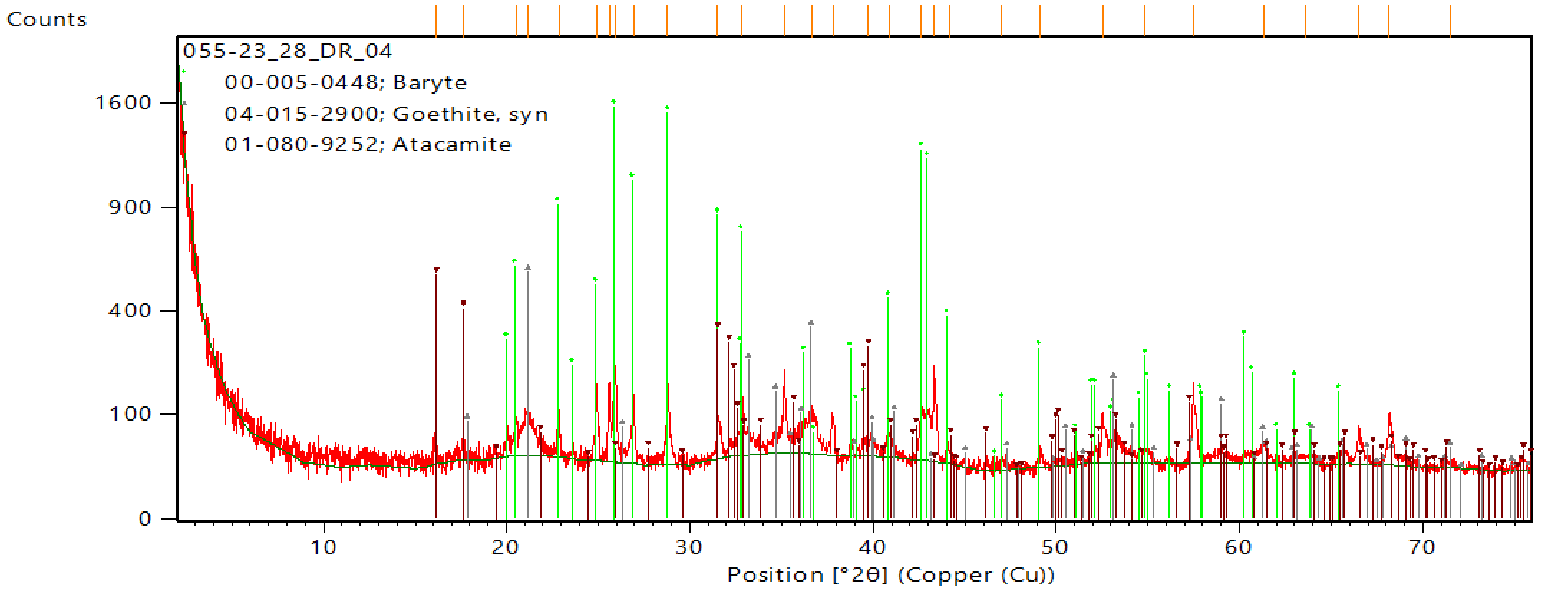
Counts
055-23_28_DR_04
00-005-0448; Baryte
04-015-2900; Goethite, syn
01-080-9252; Atacamite
1600
900
400
100
0
10
20
30
40
50
60
70
Position [°2θ] (Copper (Cu))

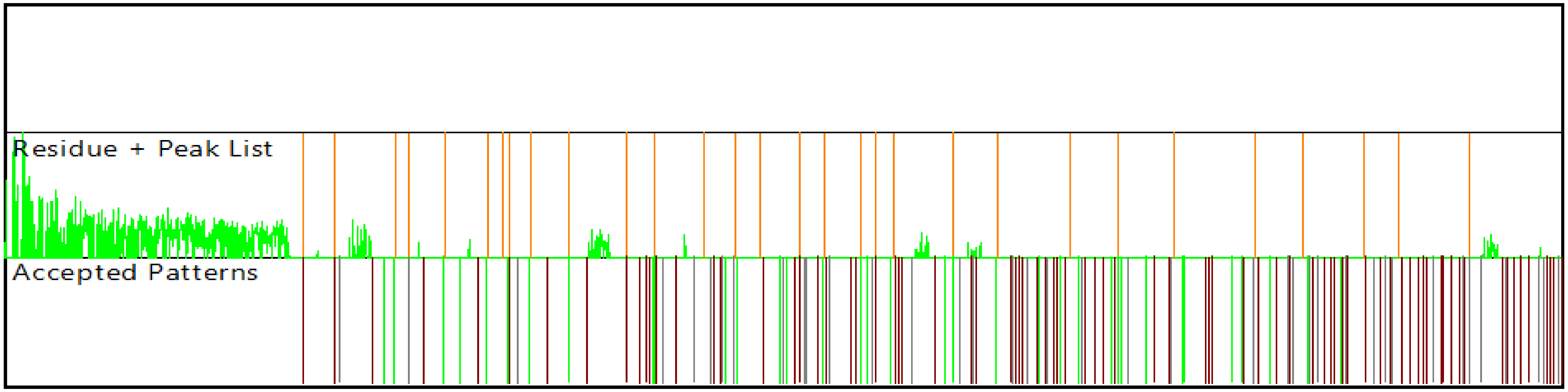
Residue + Peak List
Accepted Patterns

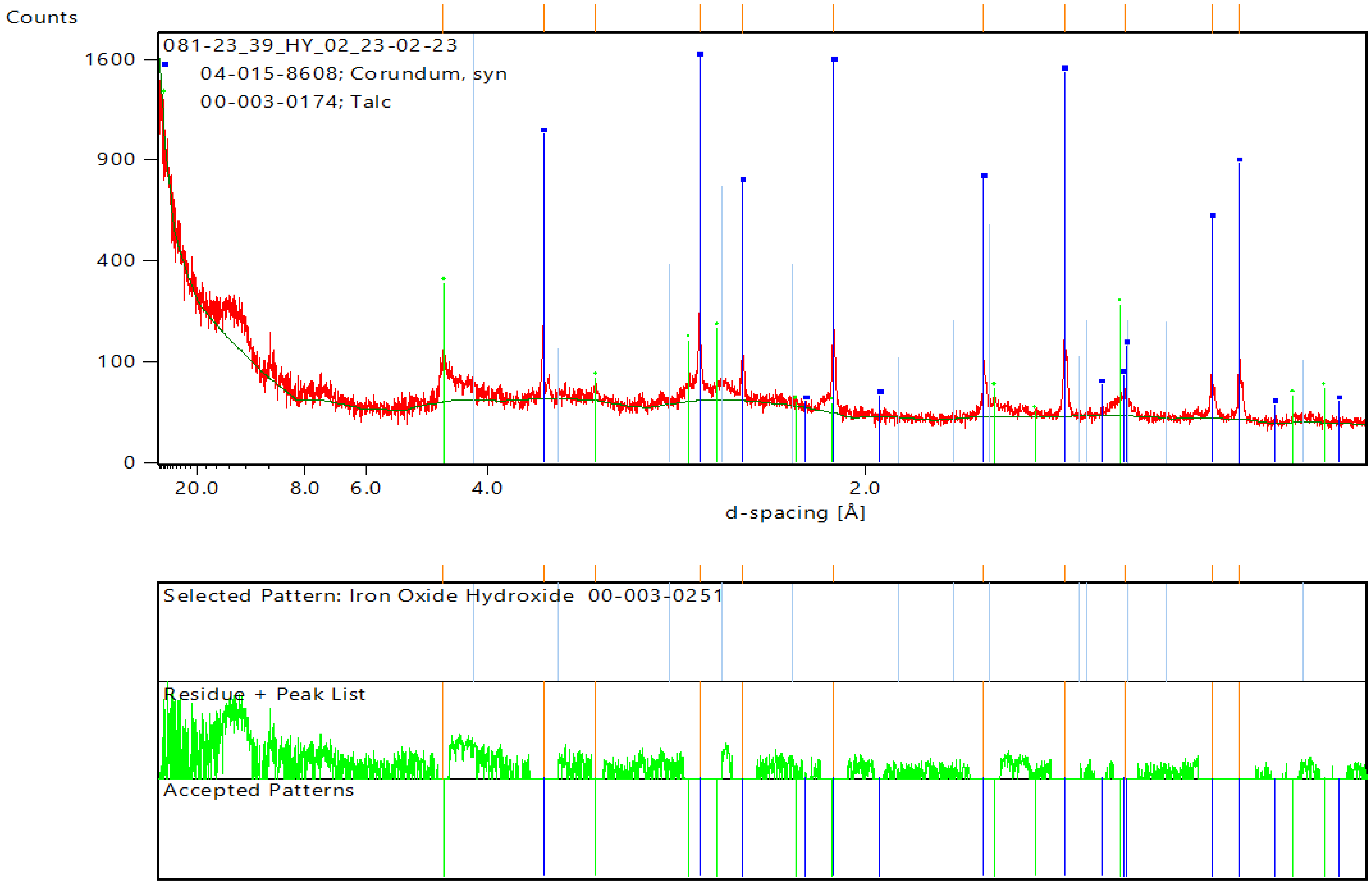
Counts
081-23_39_HY_02_23-02-23
04-015-8608; Corundum, syn
00-003-0174; Talc
1600
900
400
100
0
20.0
8.0
6.0
4.0
2.0
d-spacing [Å]
Selected Pattern: Iron Oxide Hydroxide 00-003-0251
Residue + Peak List
Accepted Patterns

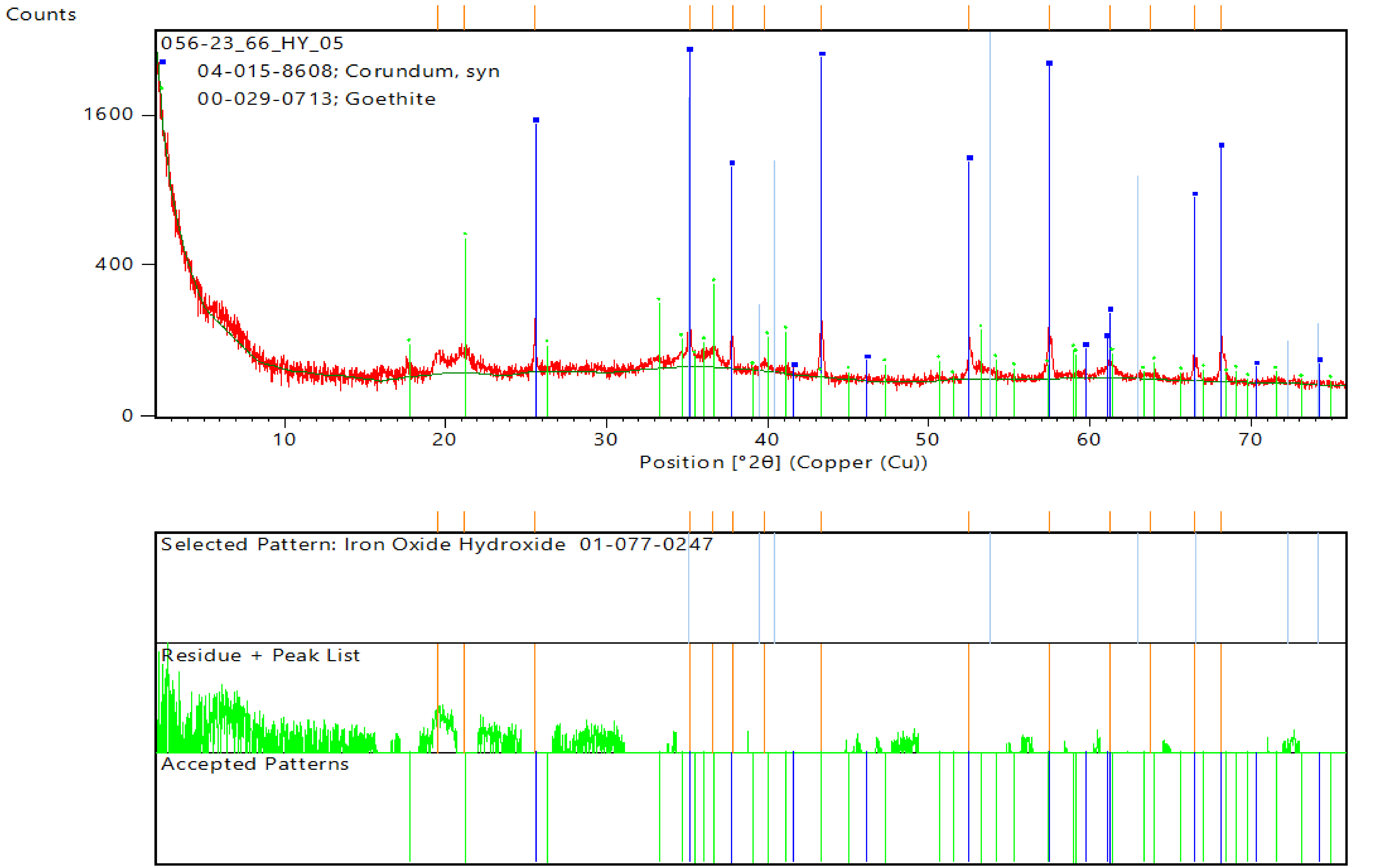

Counts
056-23_66_HY_05
04-015-8608; Corundum, syn
00-029-0713; Goethite
1600
400
0
10
20
30
40
50
60
70
Position [°2θ] (Copper (Cu))
Selected Pattern: Iron Oxide Hydroxide 01-077-0247
Residue + Peak List
Accepted Patterns

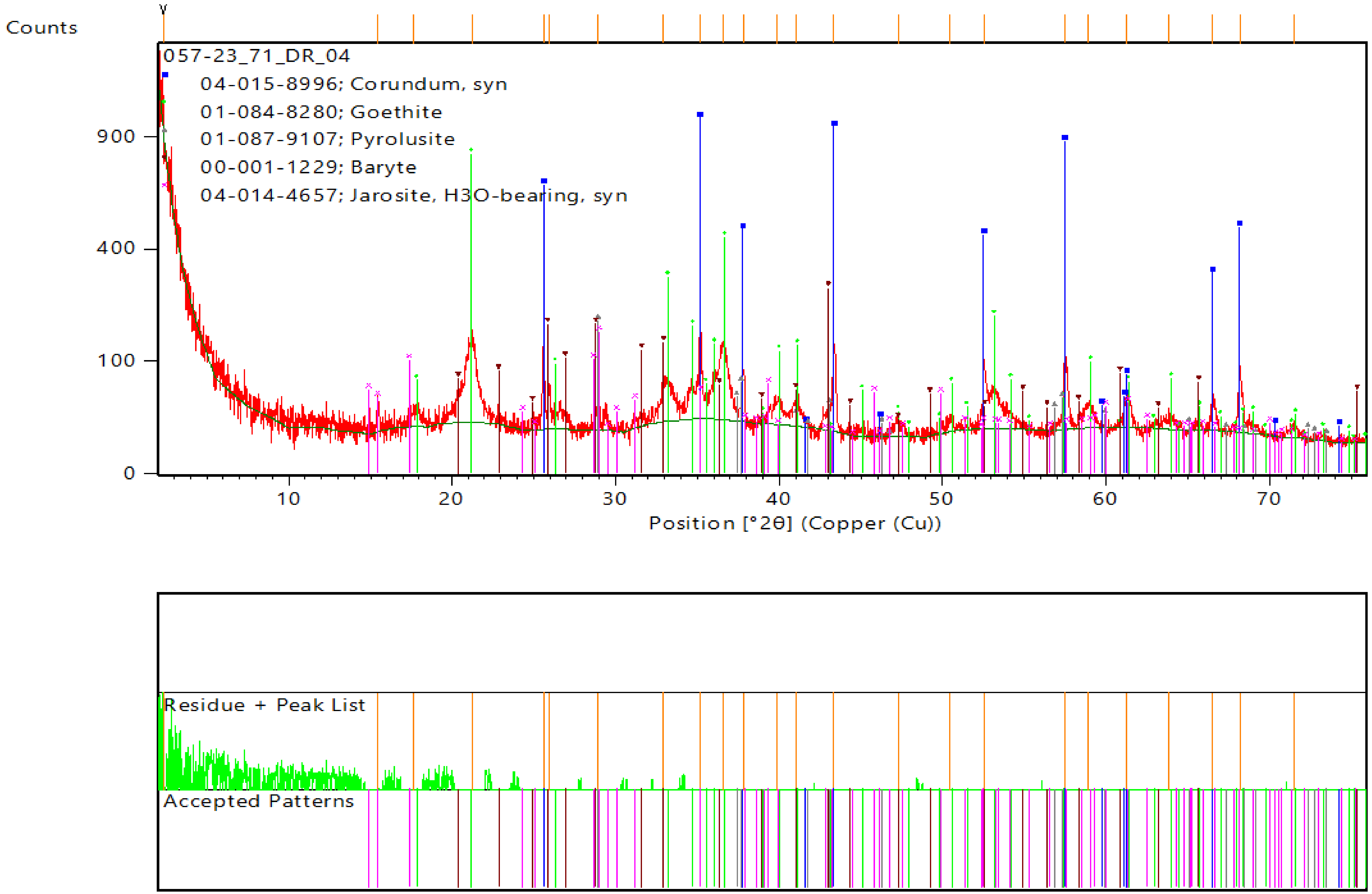
Counts
057-23_71_DR_04
04-015-8996; Corundum, syn
01-084-8280; Goethite
01-087-9107; Pyrolusite
00-001-1229; Baryte
04-014-4657; Jarosite, H3O-bearing, syn
900
400
100
0
10
20
30
40
50
60
70
Position [°2θ] (Copper (Cu))
Residue + Peak List
Accepted Patterns

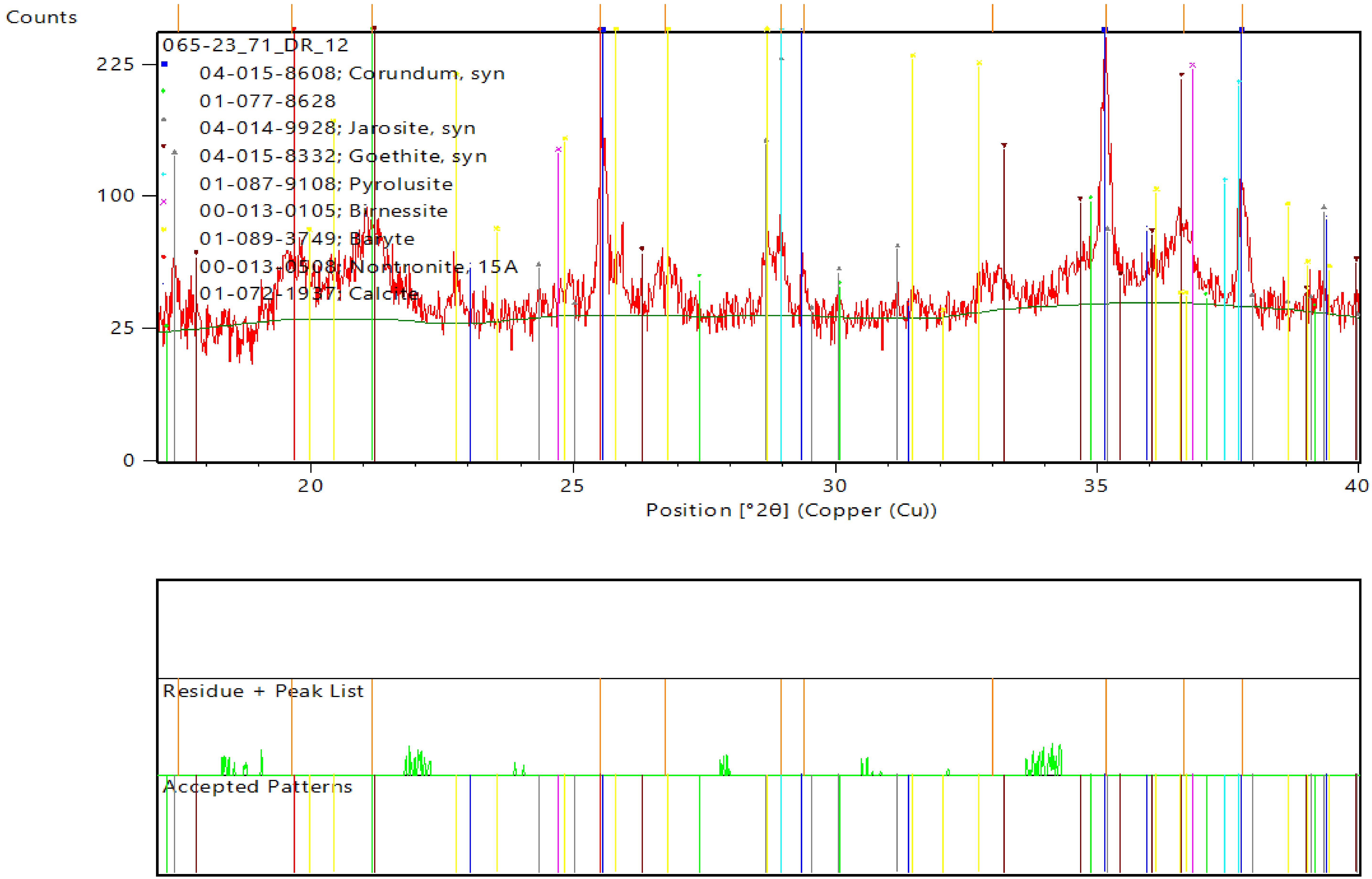
Counts
065-23_71_DR_12
04-015-8608; Corundum, syn
01-077-8628
04-014-9928; Jarosite, syn
04-015-8332; Goethite, syn
01-087-9108; Pyrolusite
00-013-0105; Birnessite
01-089-3749; Baryte
00-013-0508; Nontronite, 15A
01-072-1937; Calcite
225
100
25
0
20
25
30
35
40
Position [°2θ] (Copper (Cu))
Residue + Peak List
Accepted Patterns

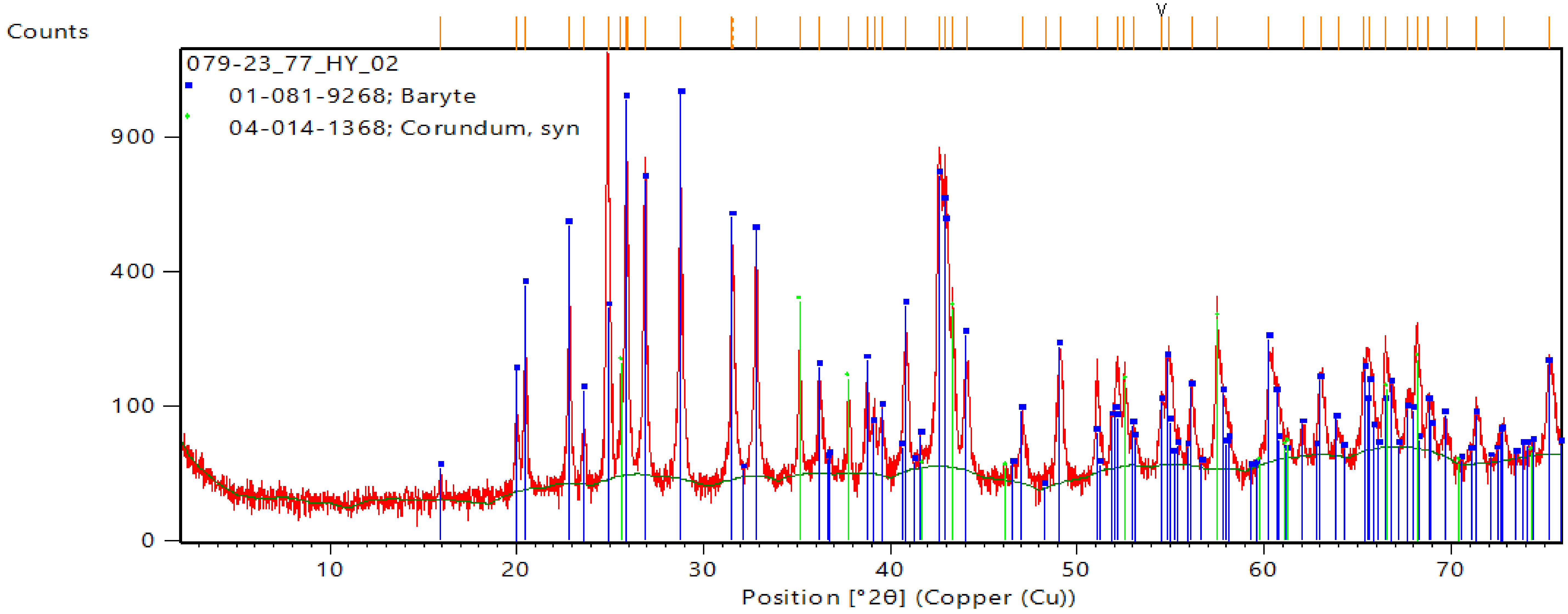

Counts
079-23_77_HY_02
01-081-9268; Baryte
04-014-1368; Corundum, syn
900
400
100
0
10
20
30
40
50
60
70
Position [°2θ] (Copper (Cu))

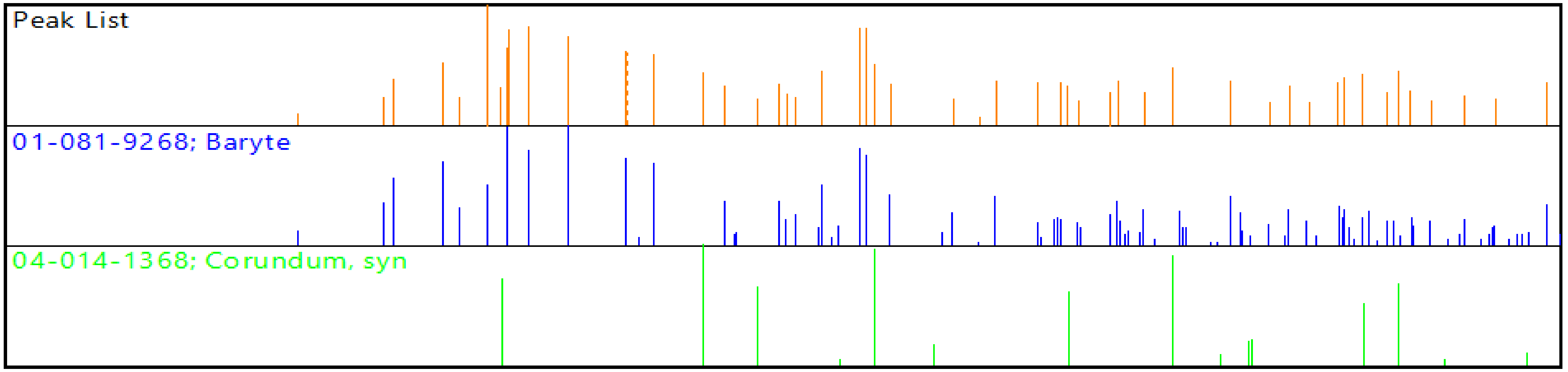

Peak List
01-081-9268; Baryte
04-014-1368; Corundum, syn

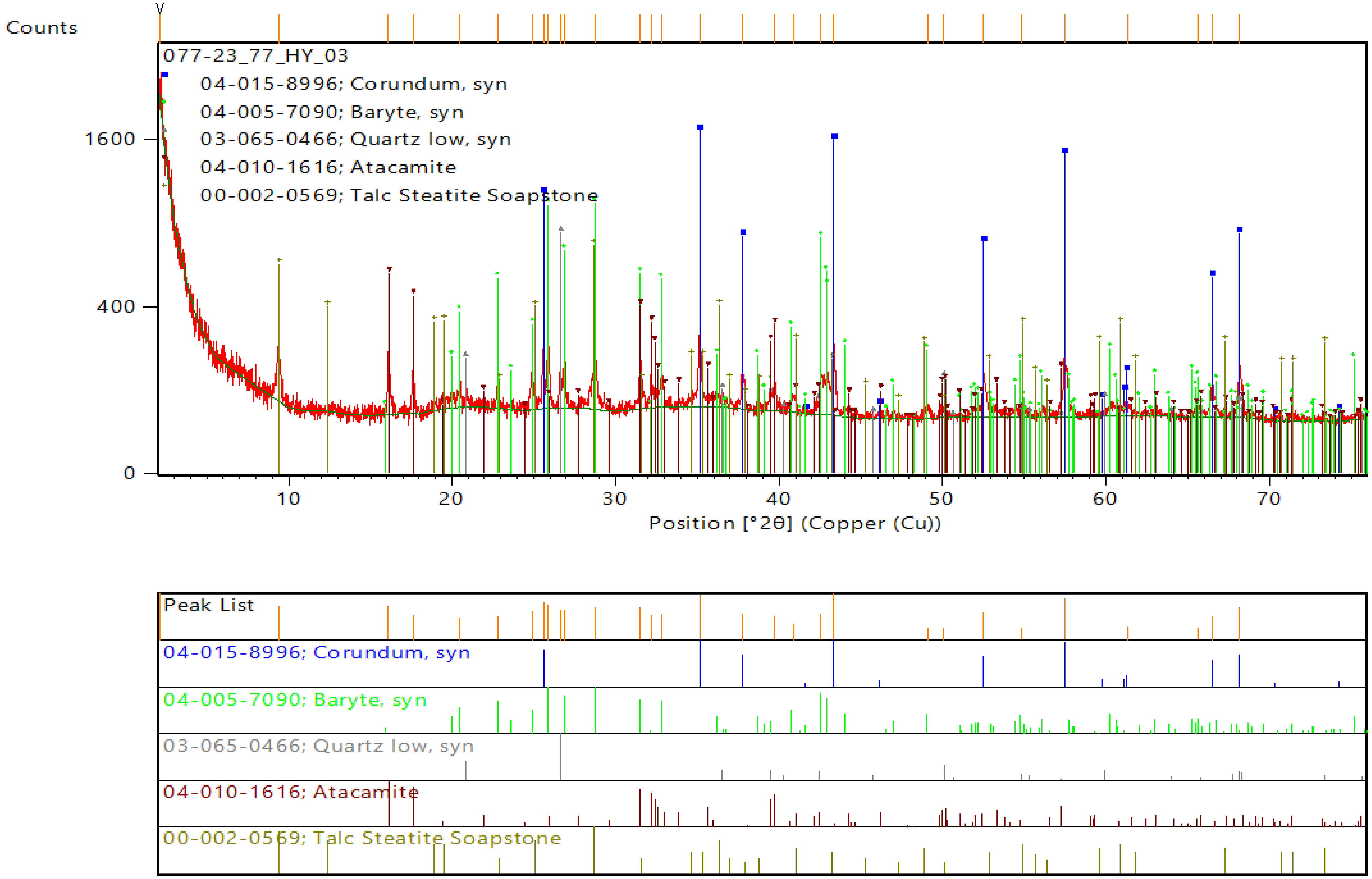
Counts
077-23_77_HY_03
04-015-8996; Corundum, syn
04-005-7090; Baryte, syn
03-065-0466; Quartz low, syn
04-010-1616; Atacamite
00-002-0569; Talc Steatite Soapstone
1600
400
0
10
20
30
40
50
60
70
Position [°2θ] (Copper (Cu))
Peak List
04-015-8996; Corundum, syn
04-005-7090; Baryte, syn
03-065-0466; Quartz low, syn
04-010-1616; Atacamite
00-002-0569; Talc Steatite Soapstone

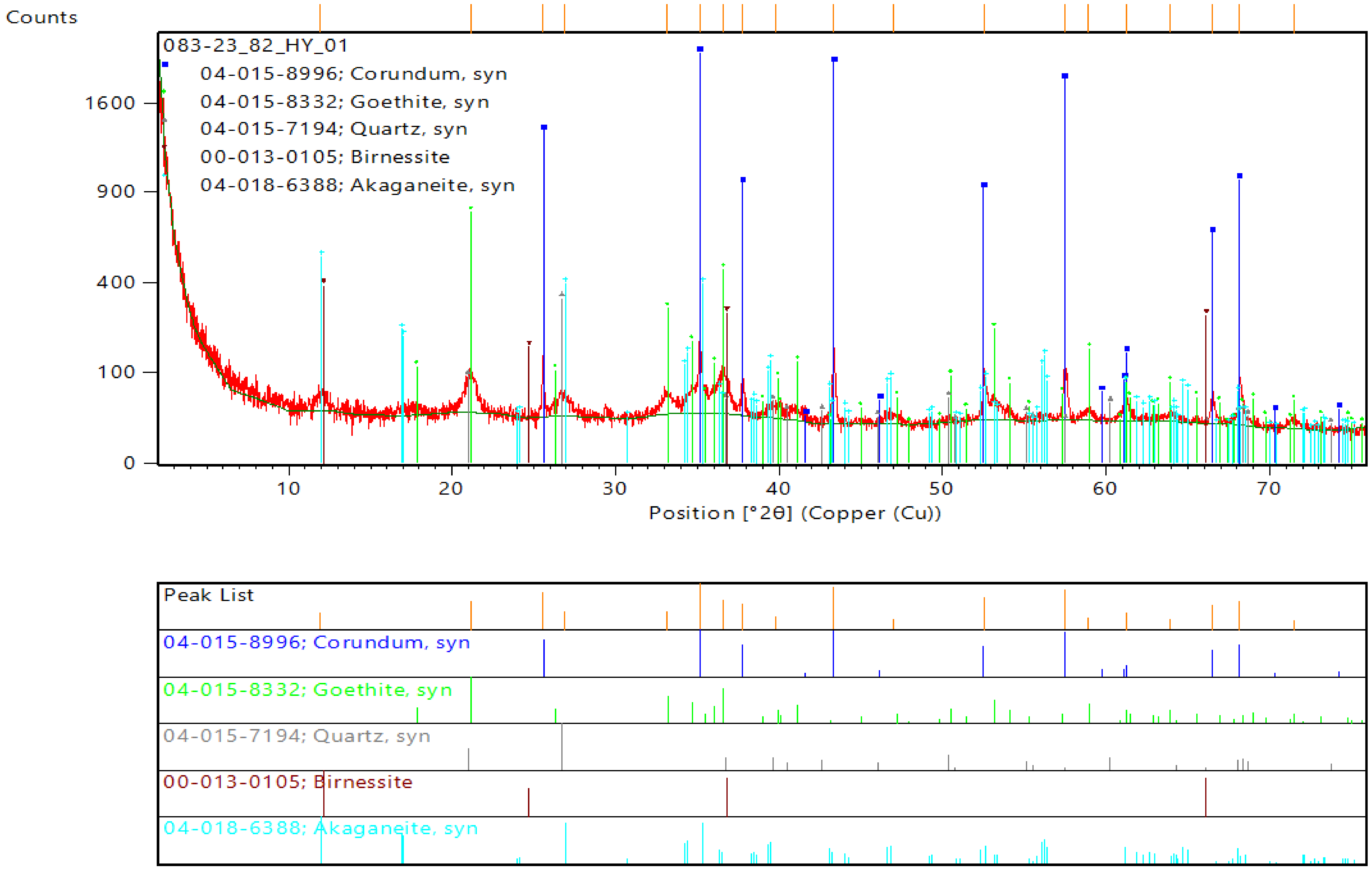
Counts
083-23_82_HY_01
04-015-8996; Corundum, syn
04-015-8332; Goethite, syn
04-015-7194; Quartz, syn
00-013-0105; Birnessite
04-018-6388; Akaganeite, syn
1600
900
400
100
0
10
20
30
40
50
60
70
Position [°2θ] (Copper (Cu))
Peak List
04-015-8996; Corundum, syn
04-015-8332; Goethite, syn
04-015-7194; Quartz, syn
00-013-0105; Birnessite
04-018-6388; Akaganeite, syn

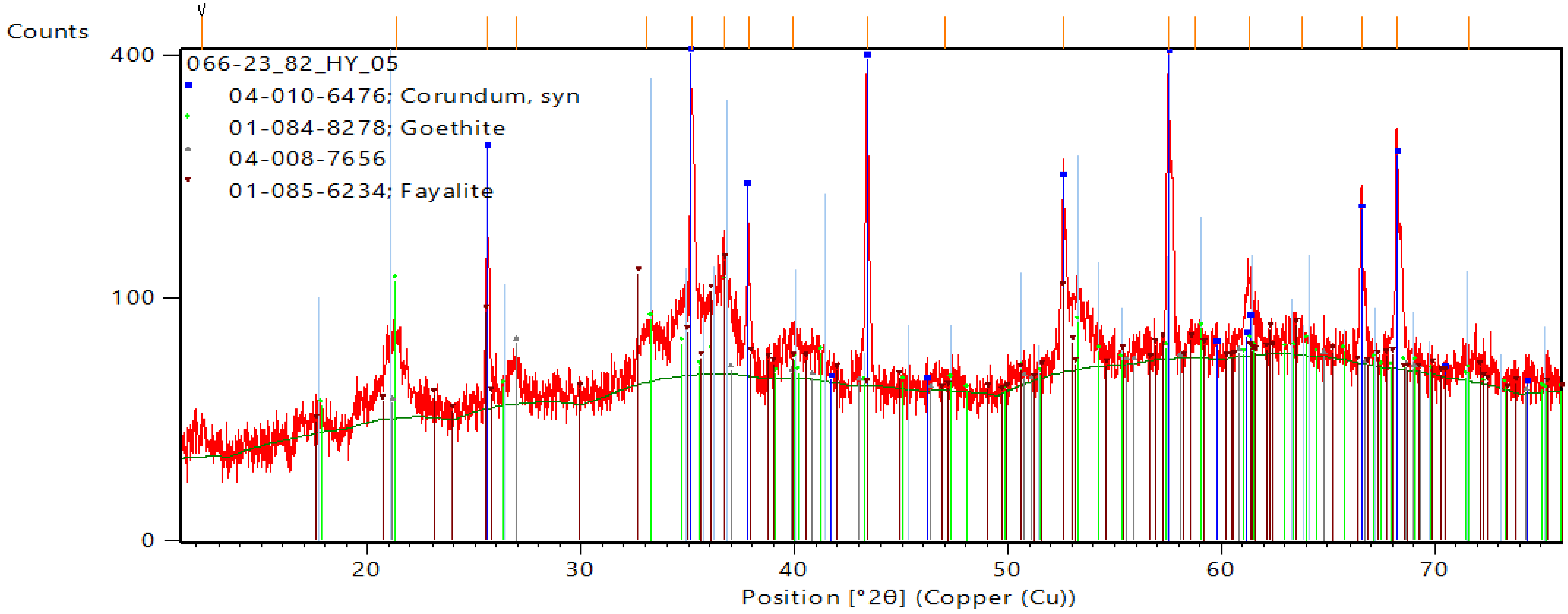
Counts
400
100
0
066-23_82_HY_05
04-010-6476; Corundum, syn
01-084-8278; Goethite
04-008-7656
01-085-6234; Fayalite
20
30
40
50
60
70
Position [°2θ] (Copper (Cu))

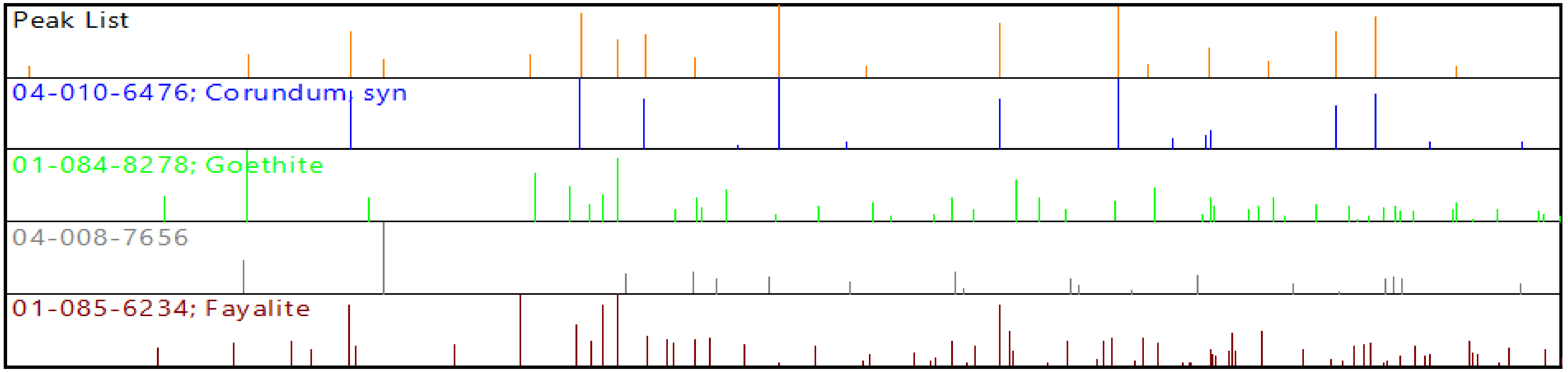
Peak List
04-010-6476; Corundum, syn
01-084-8278; Goethite
04-008-7656
01-085-6234; Fayalite

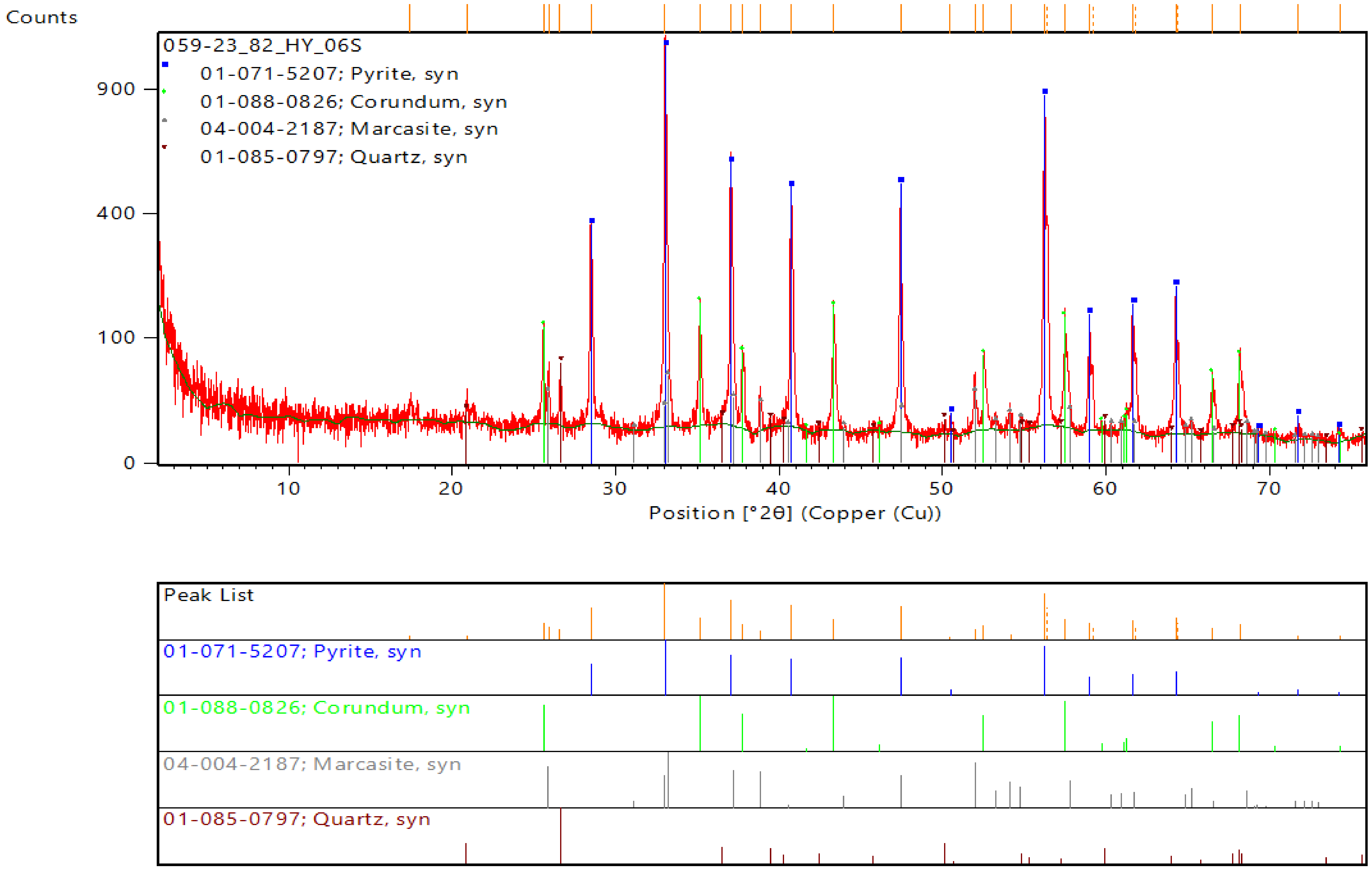
Counts
059-23_82_HY_06S
01-071-5207; Pyrite, syn
01-088-0826; Corundum, syn
04-004-2187; Marcasite, syn
01-085-0797; Quartz, syn
900
400
100
0
10
20
30
40
50
60
70
Position [°2θ] (Copper (Cu))
Peak List
01-071-5207; Pyrite, syn
01-088-0826; Corundum, syn
04-004-2187; Marcasite, syn
01-085-0797; Quartz, syn

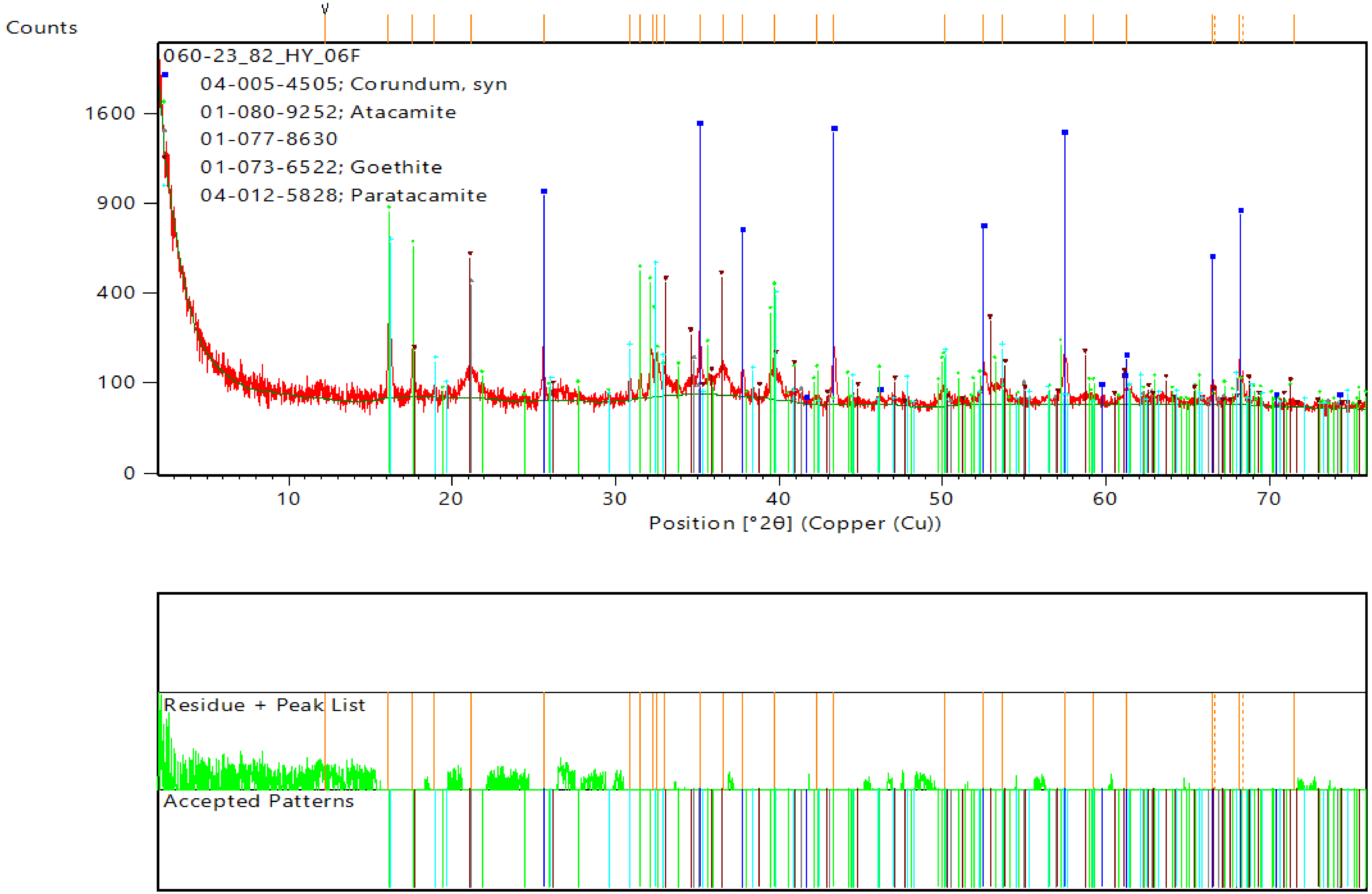
Counts
060-23_82_HY_06F
04-005-4505; Corundum, syn
01-080-9252; Atacamite
01-077-8630
01-073-6522; Goethite
04-012-5828; Paratacamite
1600
900
400
100
0
10
20
30
40
50
60
70
Position [°2θ] (Copper (Cu))
Residue + Peak List
Accepted Patterns

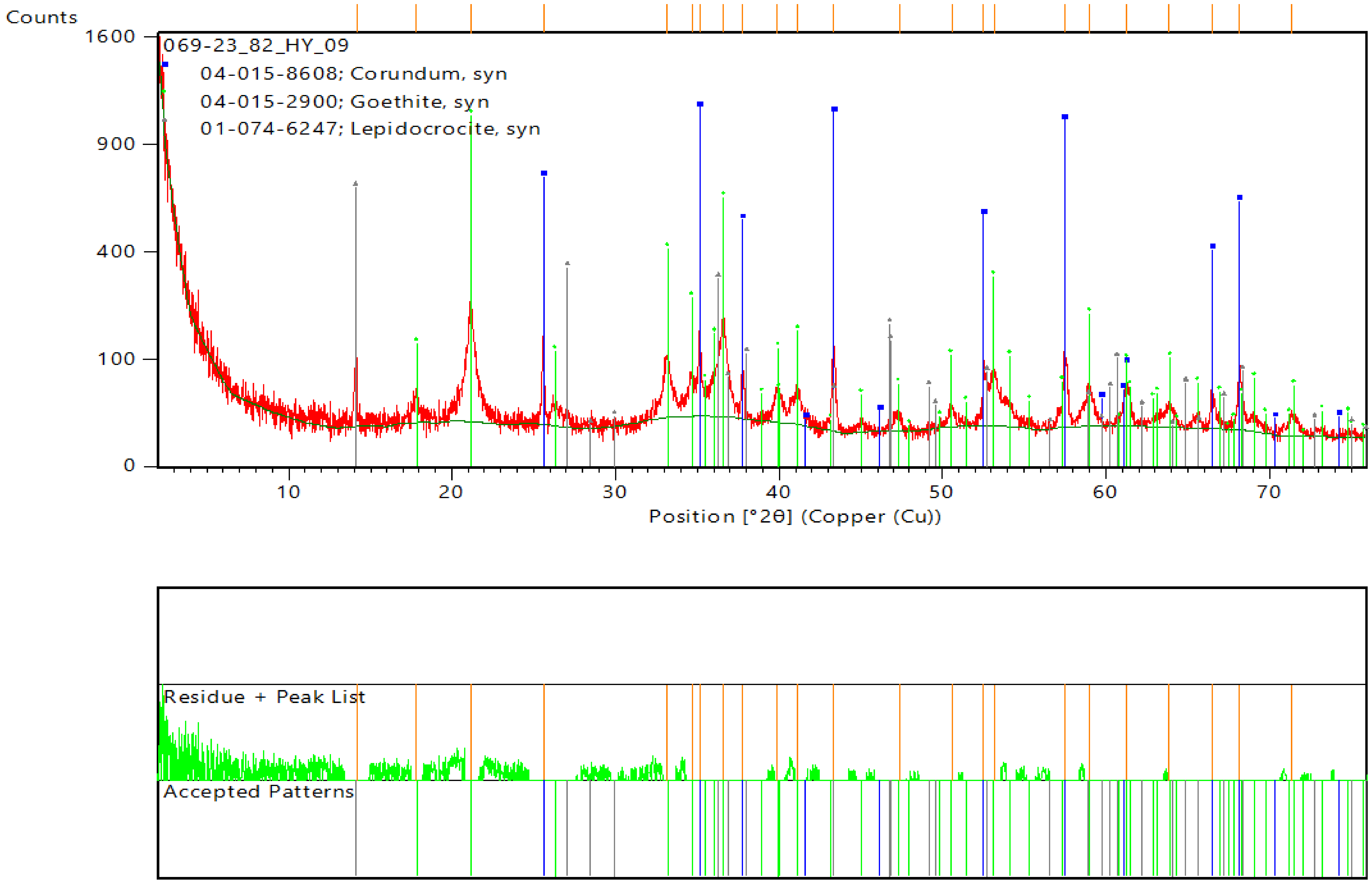
Counts
1600
900
400
100
0
069-23_82_HY_09
04-015-8608; Corundum, syn
04-015-2900; Goethite, syn
01-074-6247; Lepidocrocite, syn
10
20
30
40
50
60
70
Position [°2θ] (Copper (Cu))
Residue + Peak List
Accepted Patterns

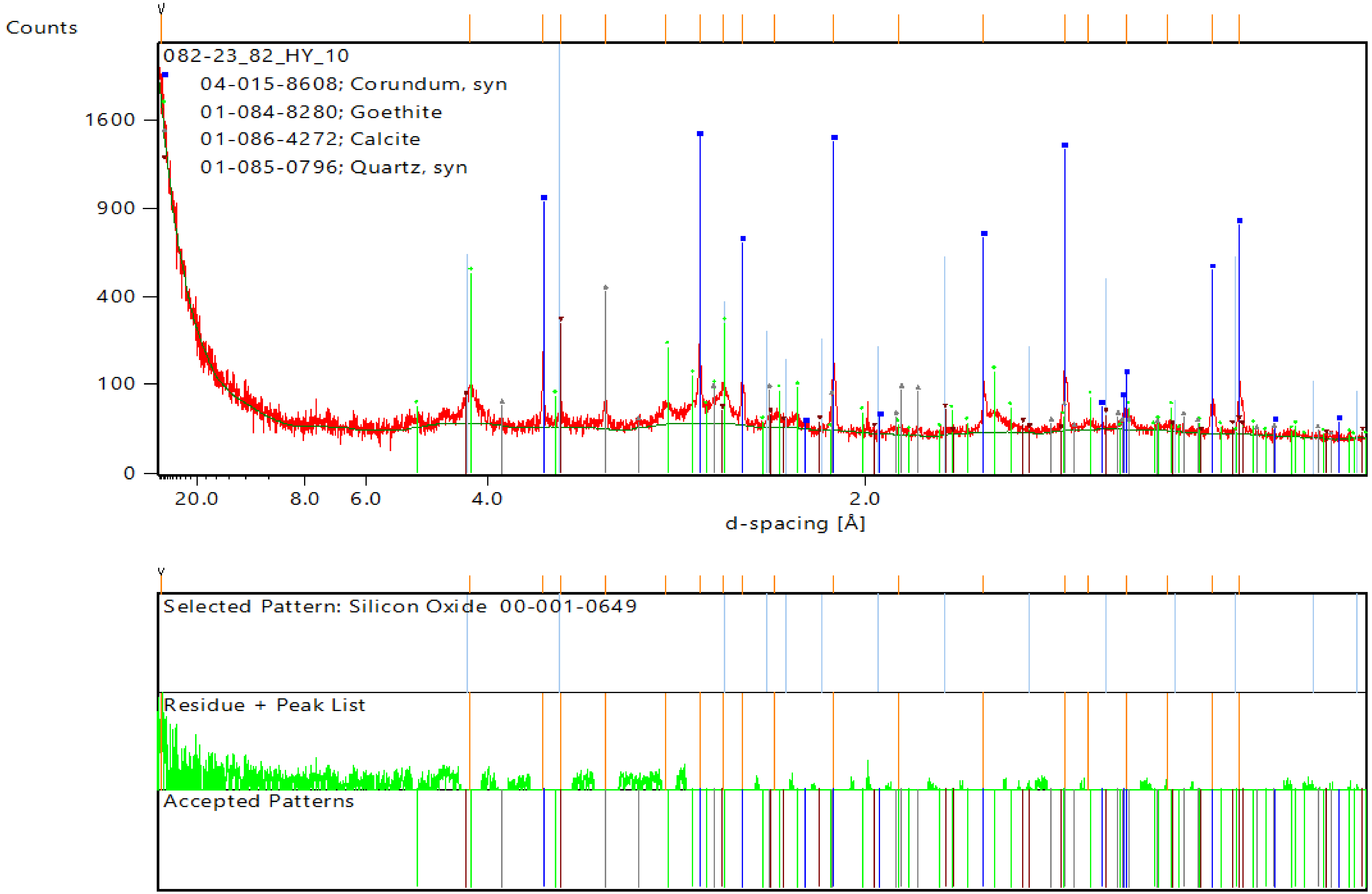
Counts
082-23_82_HY_10
04-015-8608; Corundum, syn
01-084-8280; Goethite
01-086-4272; Calcite
01-085-0796; Quartz, syn
1600
900
400
100
0
20.0
8.0
6.0
4.0
2.0
d-spacing [Å]
Selected Pattern: Silicon Oxide 00-001-0649
Residue + Peak List
Accepted Patterns

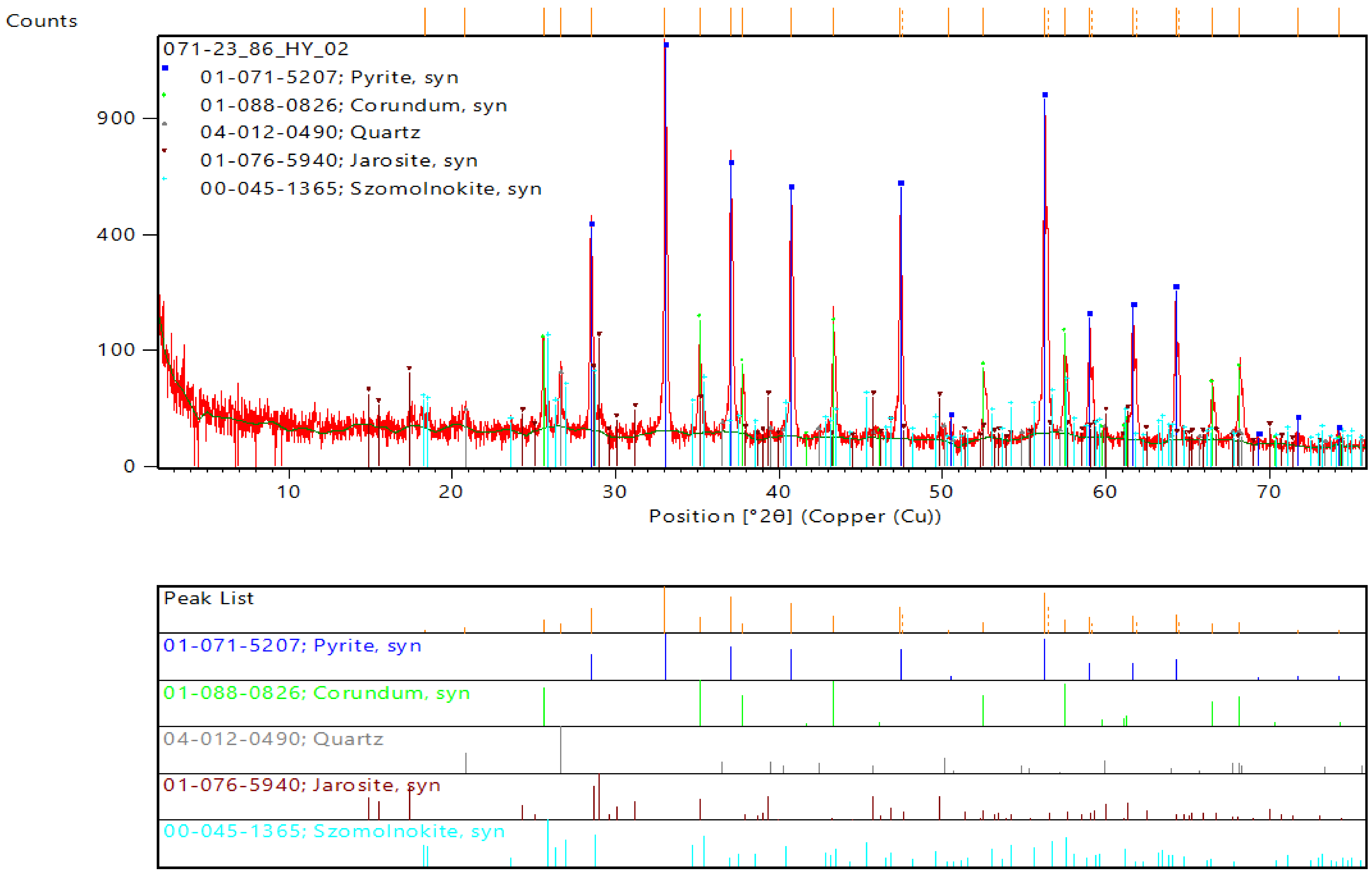
Counts
071-23_86_HY_02
01-071-5207; Pyrite, syn
01-088-0826; Corundum, syn
04-012-0490; Quartz
01-076-5940; Jarosite, syn
00-045-1365; Szomolnokite, syn
900
400
100
0
10
20
30
40
50
60
70
Position [°2θ] (Copper (Cu))
Peak List
01-071-5207; Pyrite, syn
01-088-0826; Corundum, syn
04-012-0490; Quartz
01-076-5940; Jarosite, syn
00-045-1365; Szomolnokite, syn

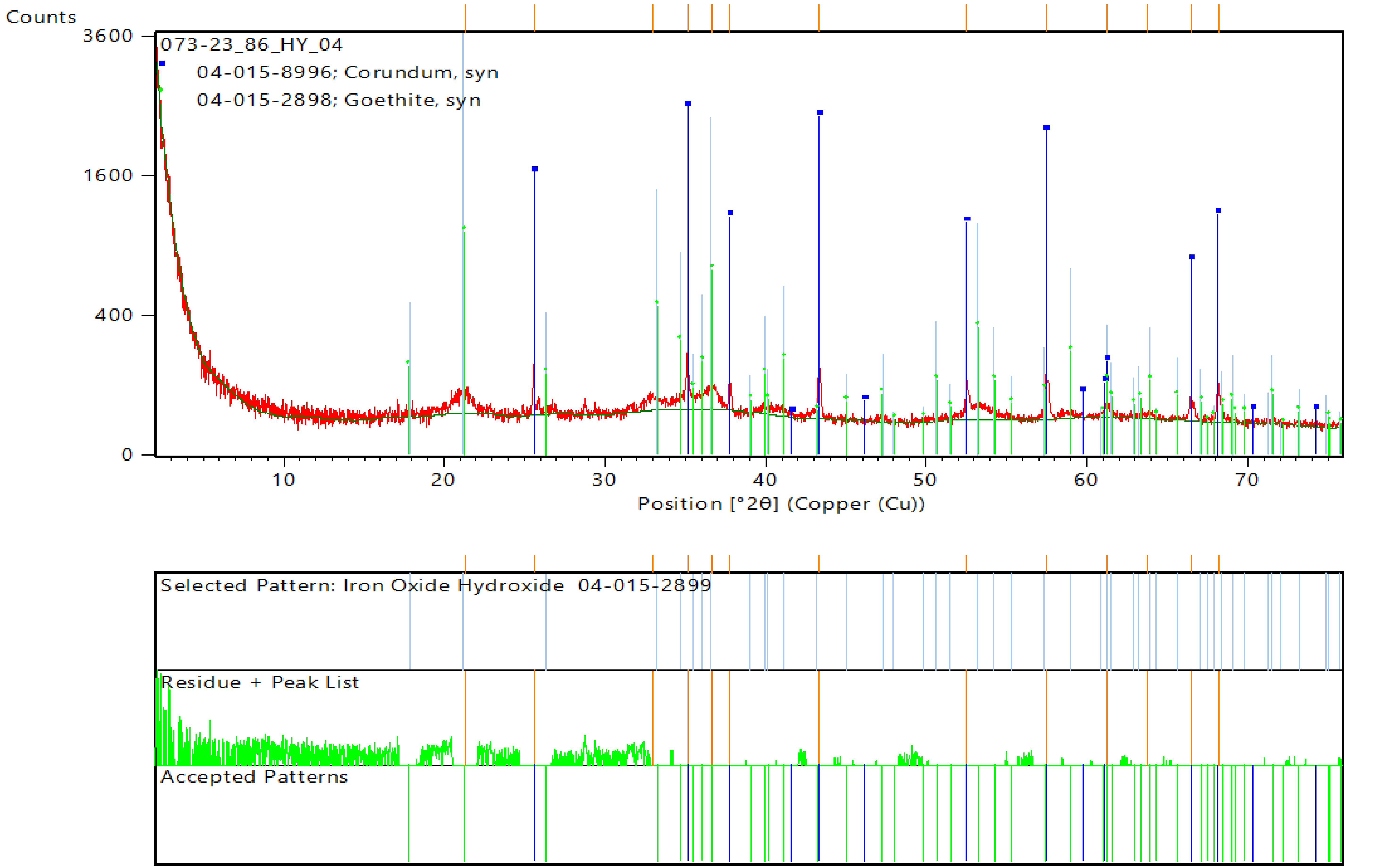
Counts
3600
1600
400
0
073-23_86_HY_04
04-015-8996; Corundum, syn
04-015-2898; Goethite, syn
10
20
30
40
50
60
70
Position [°2θ] (Copper (Cu))
Selected Pattern: Iron Oxide Hydroxide 04-015-2899
Residue + Peak List
Accepted Patterns

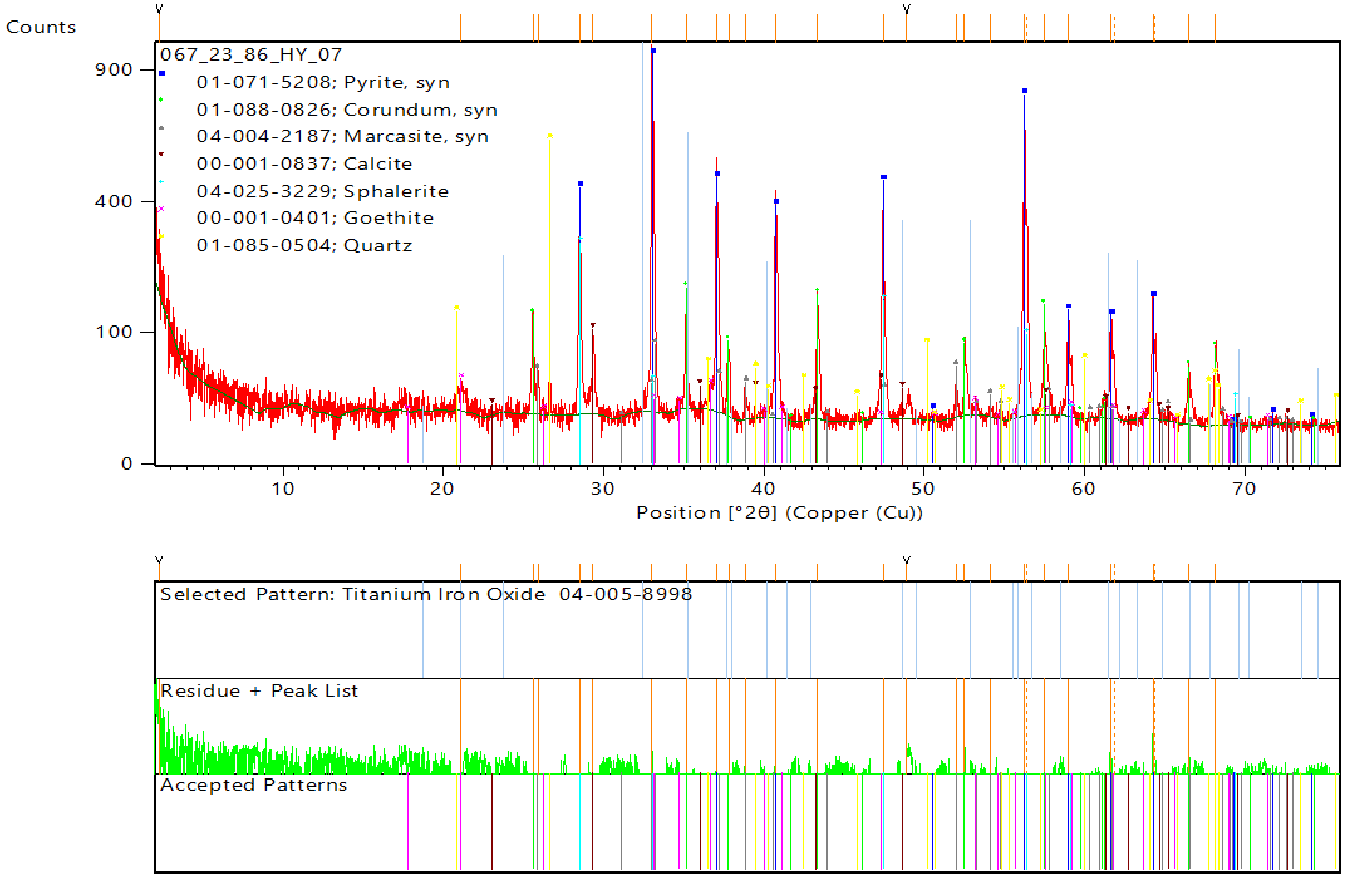
Counts
067_23_86_HY_07
01-071-5208; Pyrite, syn
01-088-0826; Corundum, syn
04-004-2187; Marcasite, syn
00-001-0837; Calcite
04-025-3229; Sphalerite
00-001-0401; Goethite
01-085-0504; Quartz
900
400
100
0
10
20
30
40
50
60
70
Position [°2θ] (Copper (Cu))
Selected Pattern: Titanium Iron Oxide 04-005-8998
Residue + Peak List
Accepted Patterns

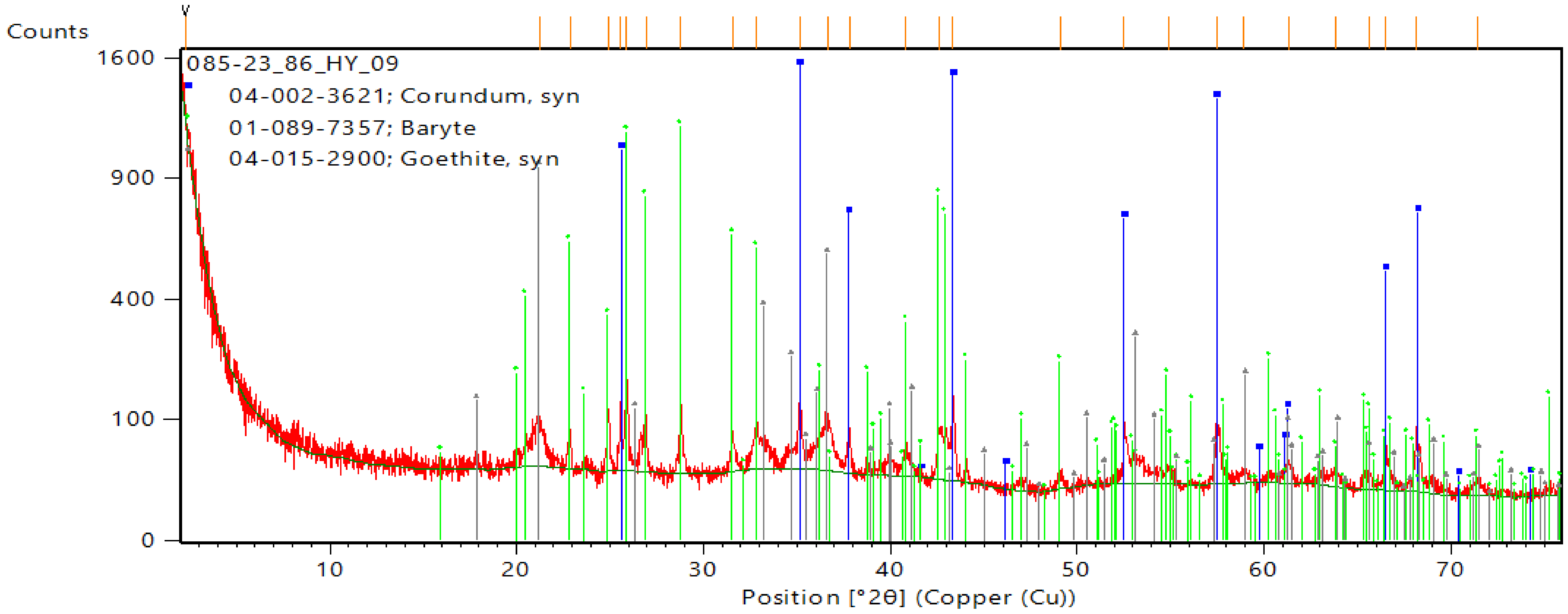
Counts
1600
900
400
100
0
085-23_86_HY_09
04-002-3621; Corundum, syn
01-089-7357; Baryte
04-015-2900; Goethite, syn
10
20
30
40
50
60
70
Position [°2θ] (Copper (Cu))

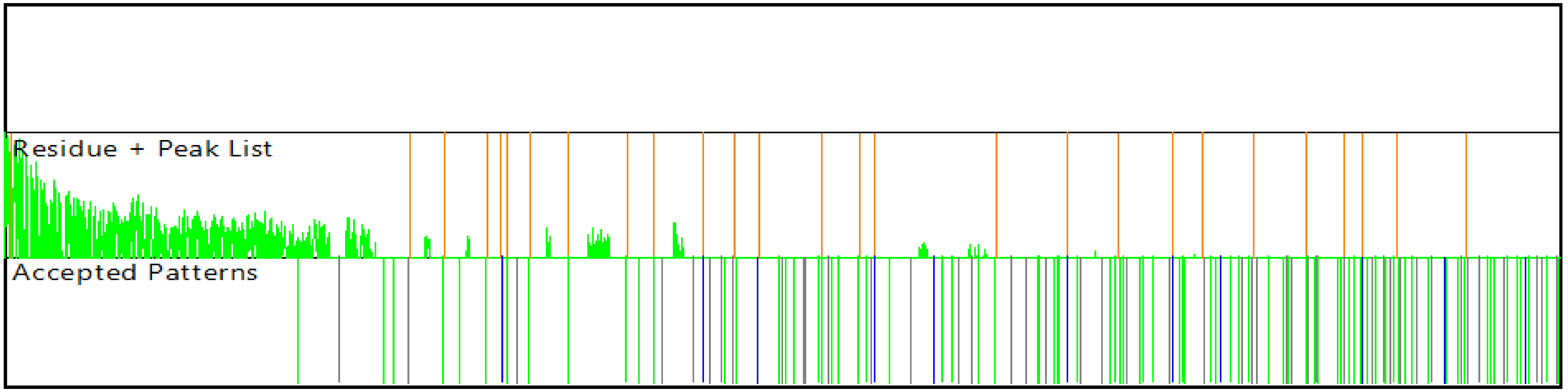
Residue + Peak List
Accepted Patterns

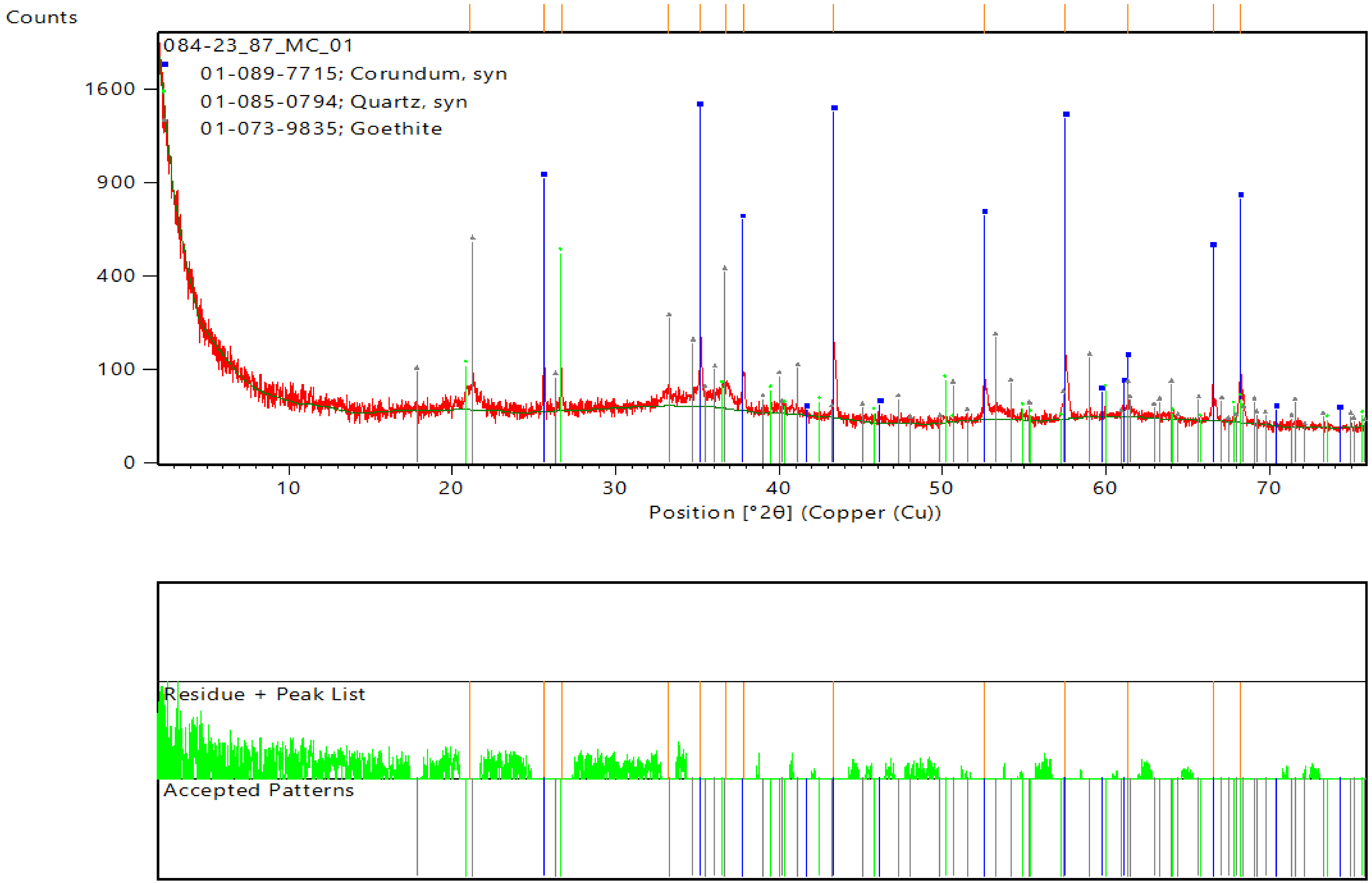
Counts
084-23_87_MC_01
01-089-7715; Corundum, syn
01-085-0794; Quartz, syn
01-073-9835; Goethite
1600
900
400
100
0
10
20
30
40
50
60
70
Position [°2θ] (Copper (Cu))
Residue + Peak List
Accepted Patterns

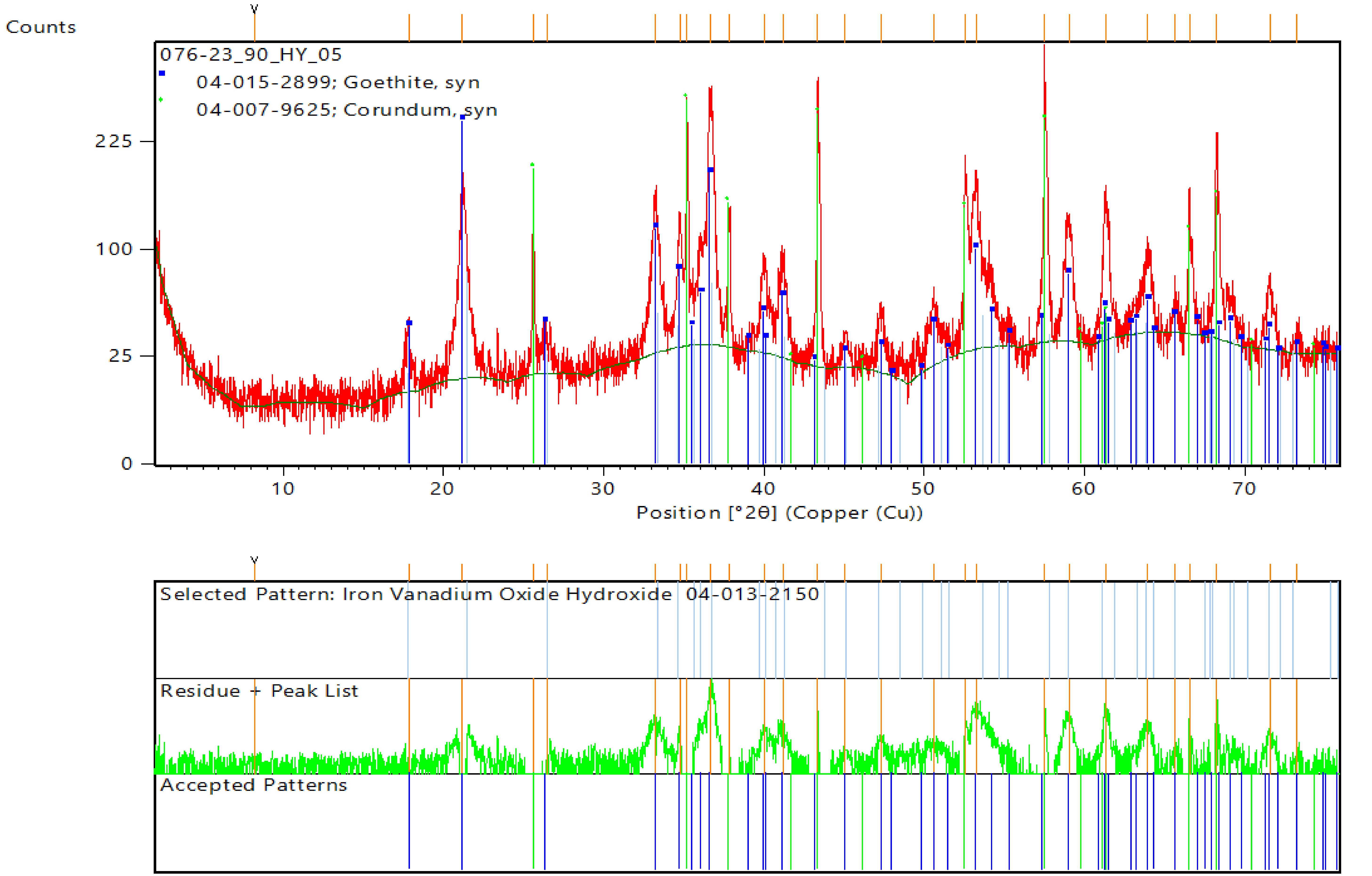
Counts
076-23_90_HY_05
04-015-2899; Goethite, syn
04-007-9625; Corundum, syn
225
100
25
0
10
20
30
40
50
60
70
Position [°2θ] (Copper (Cu))
Selected Pattern: Iron Vanadium Oxide Hydroxide 04-013-2150
Residue + Peak List
Accepted Patterns

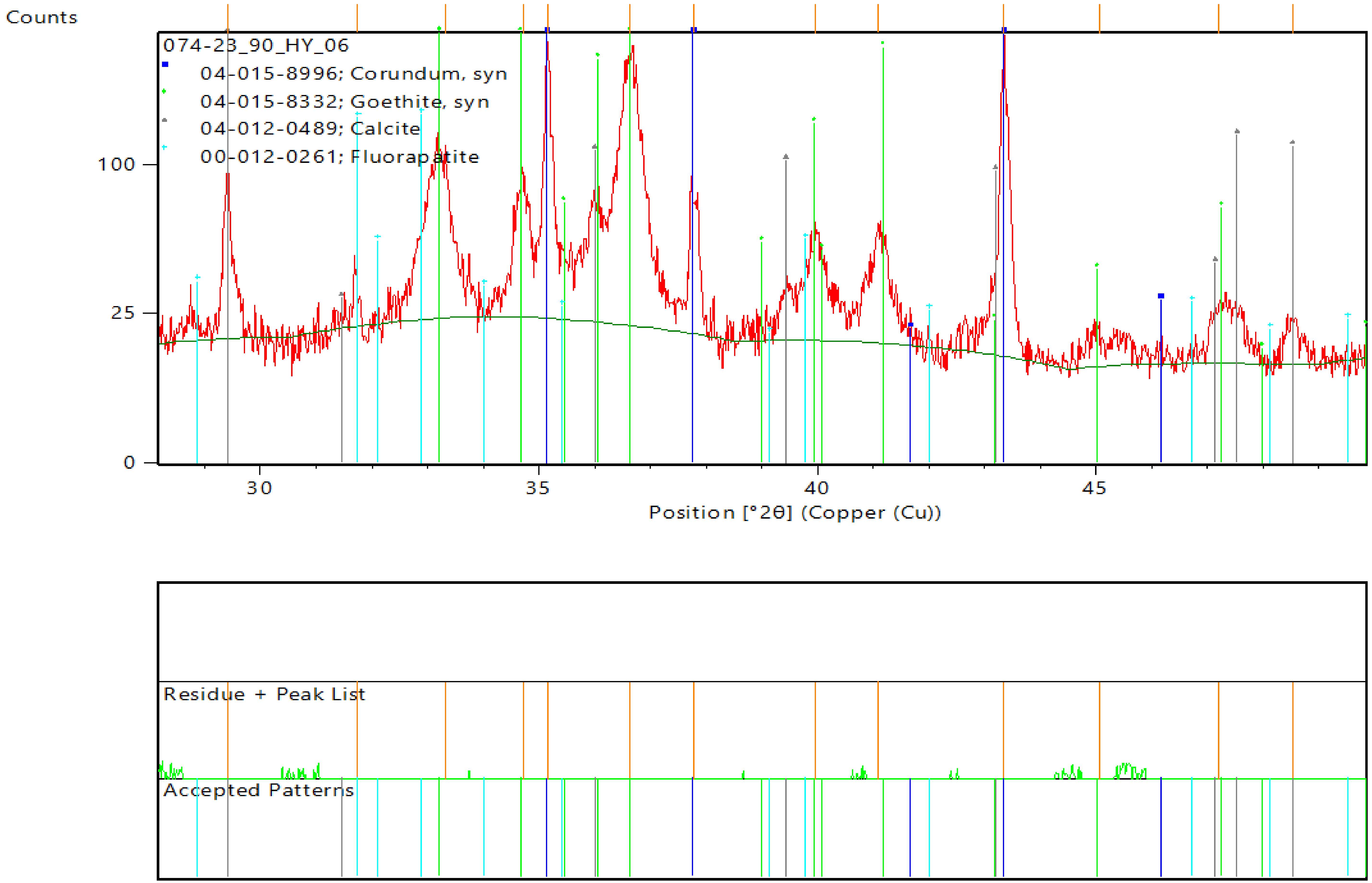
Counts
074-23_90_HY_06
04-015-8996; Corundum, syn
04-015-8332; Goethite, syn
04-012-0489; Calcite
00-012-0261; Fluorapatite
100
25
0
30
35
40
45
Position [°2θ] (Copper (Cu))
Residue + Peak List
Accepted Patterns

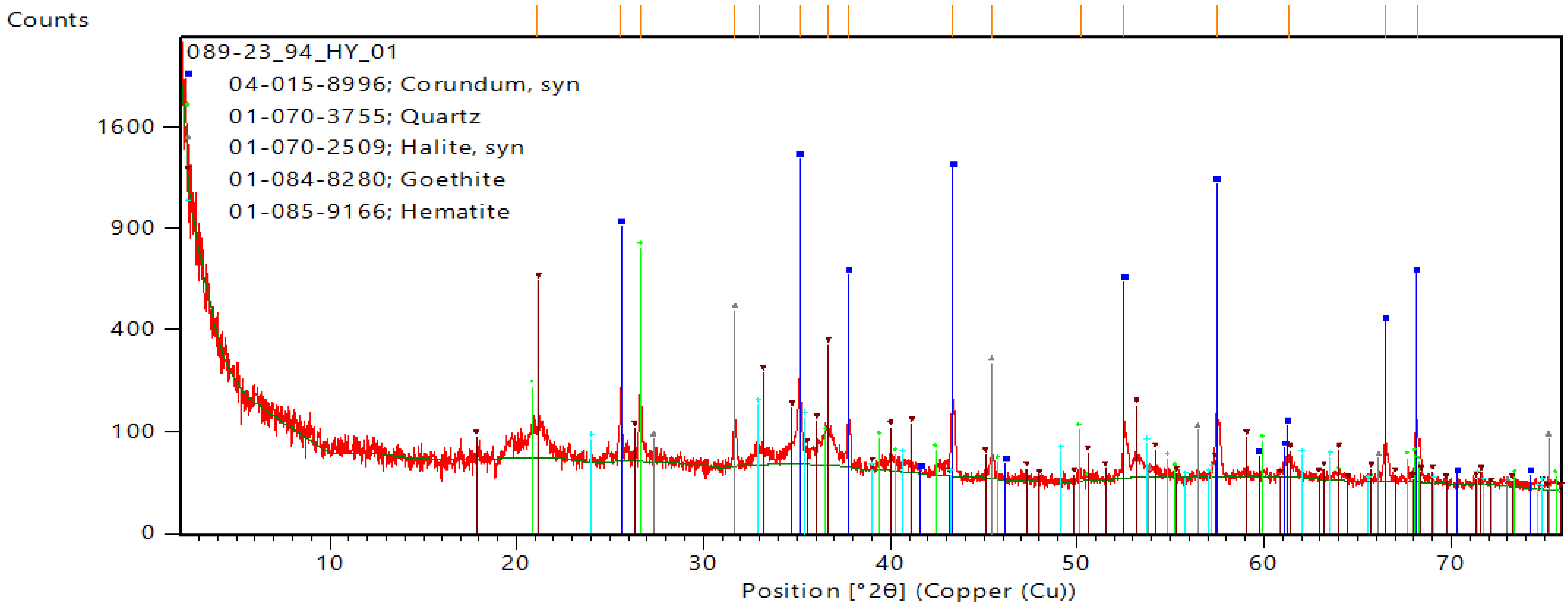
Counts
089-23_94_HY_01
04-015-8996; Corundum, syn
01-070-3755; Quartz
01-070-2509; Halite, syn
01-084-8280; Goethite
01-085-9166; Hematite
1600
900
400
100
0
10
20
30
40
50
60
70
Position [°2θ] (Copper (Cu))

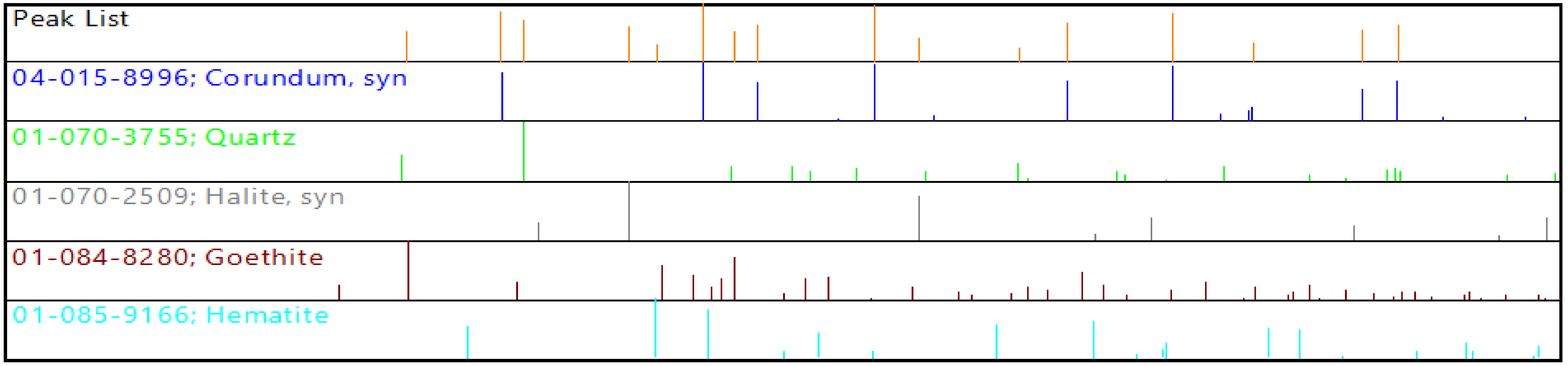
Peak List
04-015-8996; Corundum, syn
01-070-3755; Quartz
01-070-2509; Halite, syn
01-084-8280; Goethite
01-085-9166; Hematite

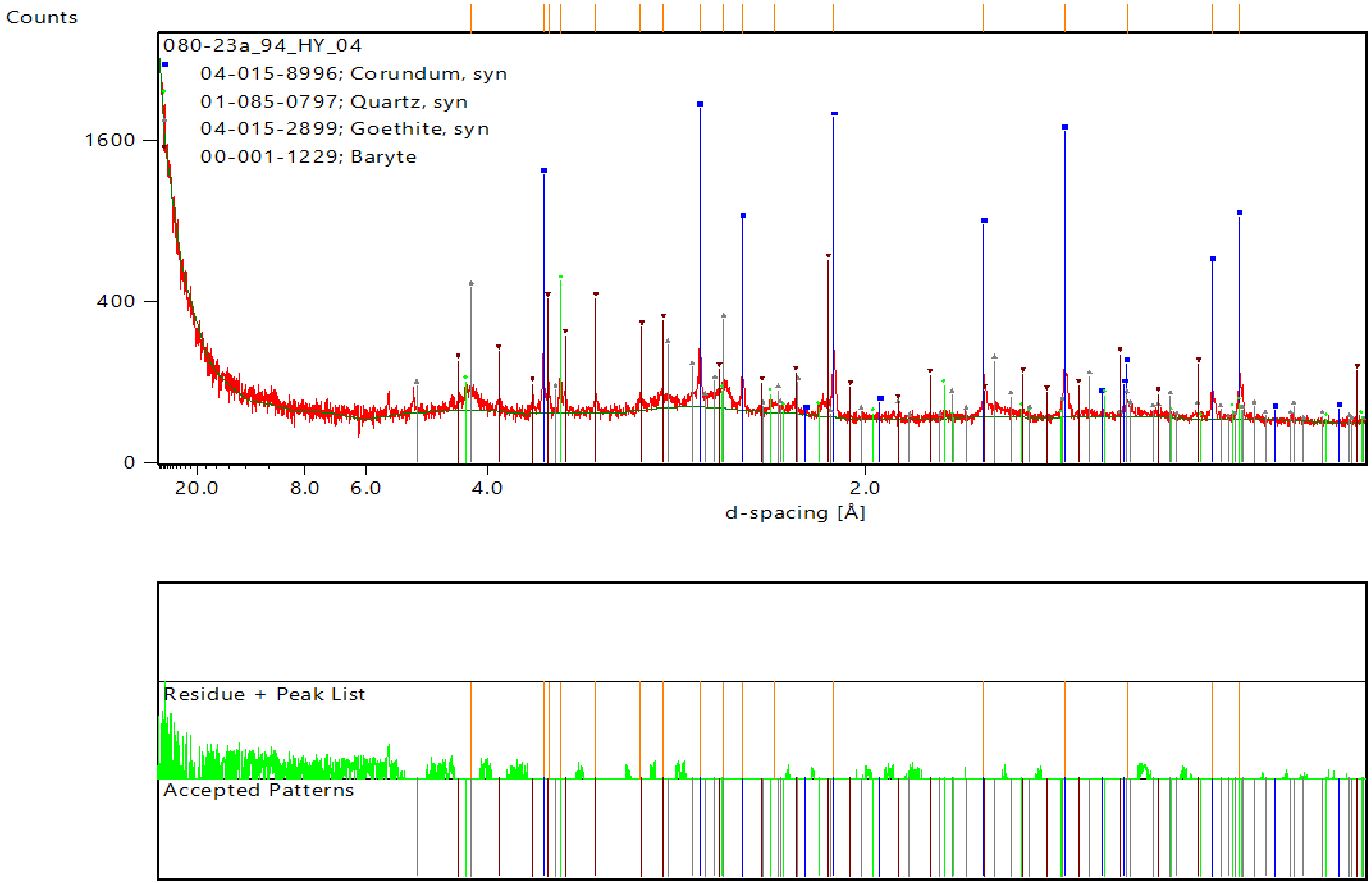
Counts
080-23a_94_HY_04
04-015-8996; Corundum, syn
01-085-0797; Quartz, syn
04-015-2899; Goethite, syn
00-001-1229; Baryte
1600
400
0
20.0
8.0
6.0
4.0
2.0
d-spacing [Å]
Residue + Peak List
Accepted Patterns

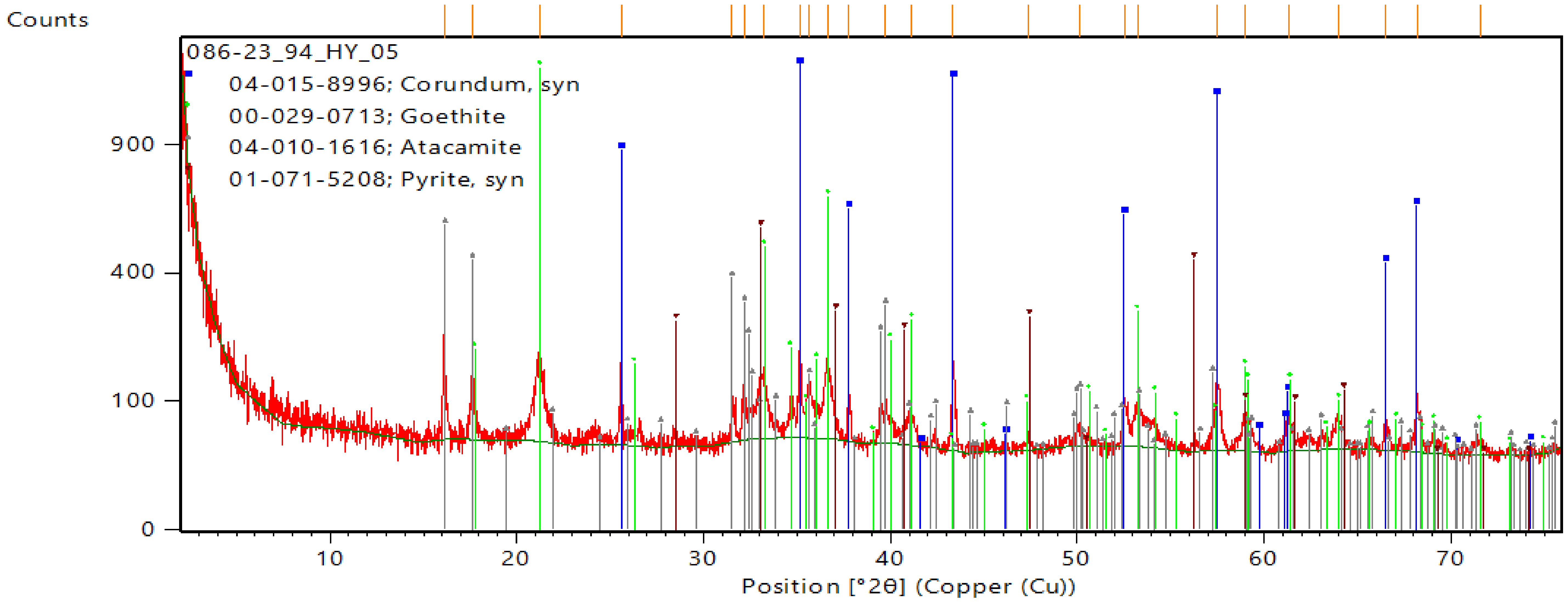
Counts
086-23_94_HY_05
04-015-8996; Corundum, syn
00-029-0713; Goethite
04-010-1616; Atacamite
01-071-5208; Pyrite, syn
900
400
100
0
10
20
30
40
50
60
70
Position [°2θ] (Copper (Cu))

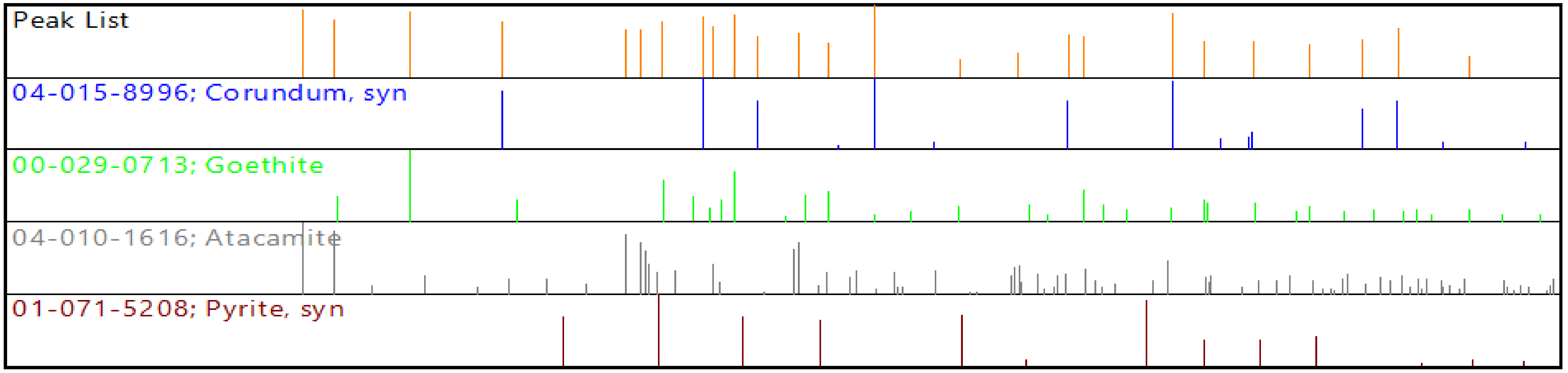
Peak List
04-015-8996; Corundum, syn
00-029-0713; Goethite
04-010-1616; Atacamite
01-071-5208; Pyrite, syn

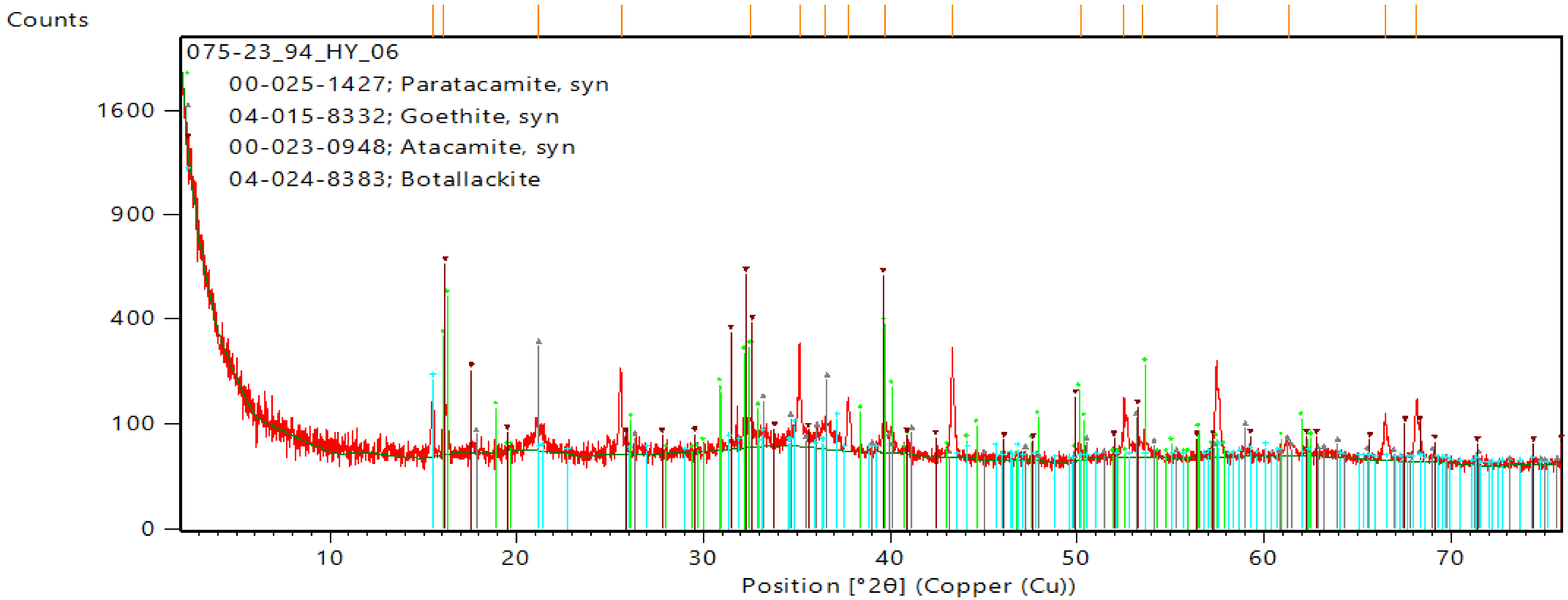
Counts
075-23_94_HY_06
00-025-1427; Paratacamite, syn
04-015-8332; Goethite, syn
00-023-0948; Atacamite, syn
04-024-8383; Botallackite
1600
900
400
100
0
10
20
30
40
50
60
70
Position [°2θ] (Copper (Cu))

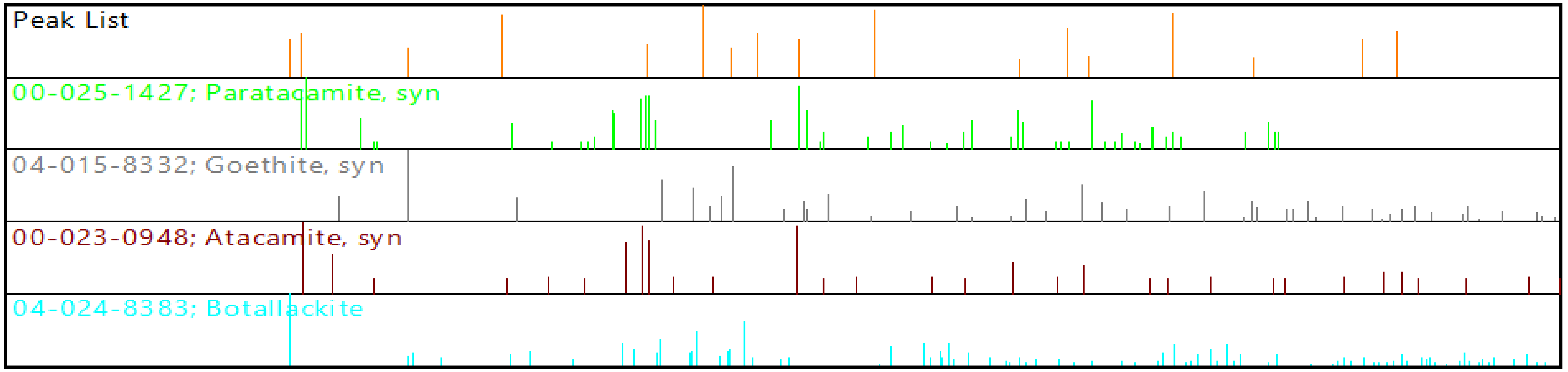
Peak List
00-025-1427; Paratacamite, syn
04-015-8332; Goethite, syn
00-023-0948; Atacamite, syn
04-024-8383; Botallackite

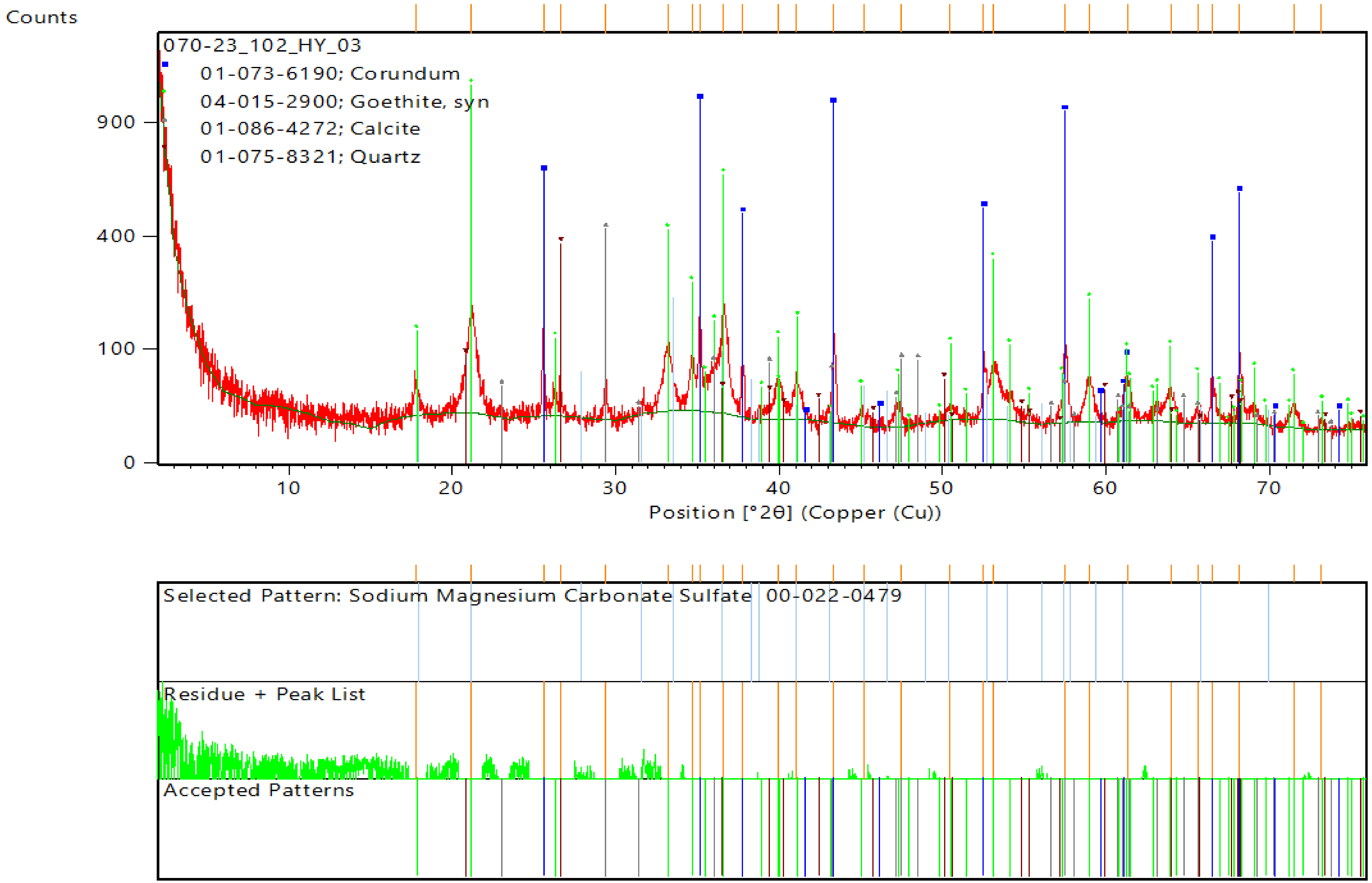
Counts
070-23_102_HY_03
01-073-6190; Corundum
04-015-2900; Goethite, syn
01-086-4272; Calcite
01-075-8321; Quartz
900
400
100
0
10
20
30
40
50
60
70
Position [°2θ] (Copper (Cu))
Selected Pattern: Sodium Magnesium Carbonate Sulfate 00-022-0479
Residue + Peak List
Accepted Patterns

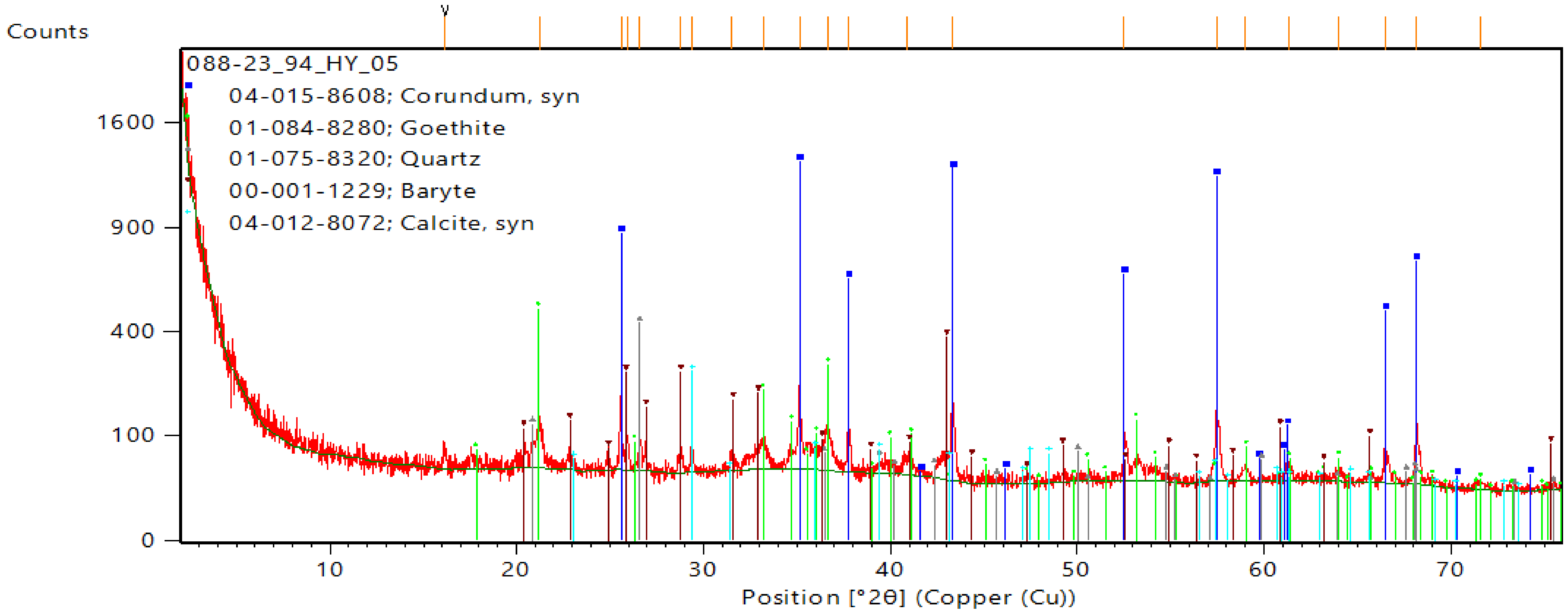
Counts
088-23_94_HY_05
04-015-8608; Corundum, syn
01-084-8280; Goethite
01-075-8320; Quartz
00-001-1229; Baryte
04-012-8072; Calcite, syn
1600
900
400
100
0
10
20
30
40
50
60
70
Position [°2θ] (Copper (Cu))

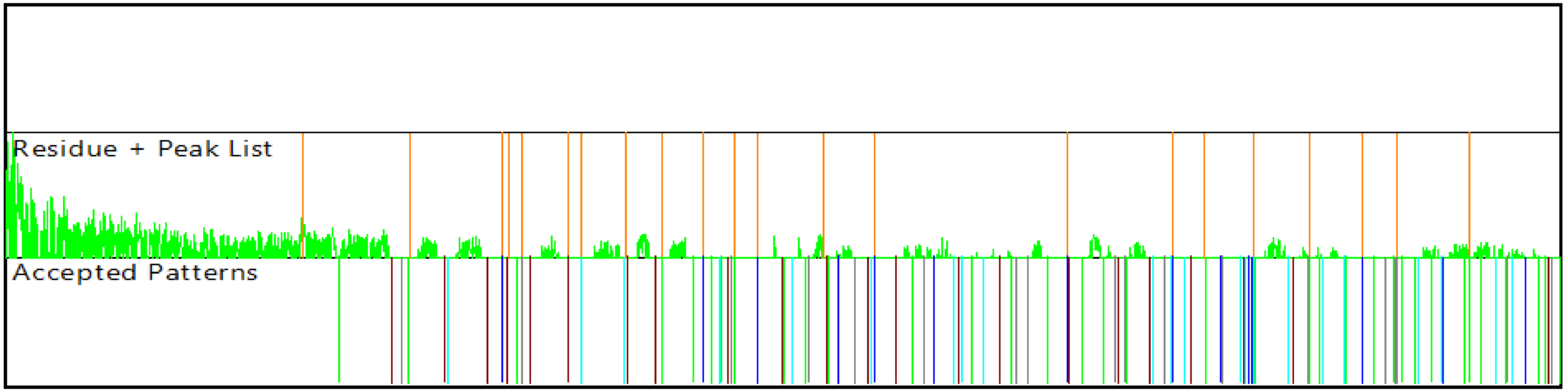
Residue + Peak List
Accepted Patterns

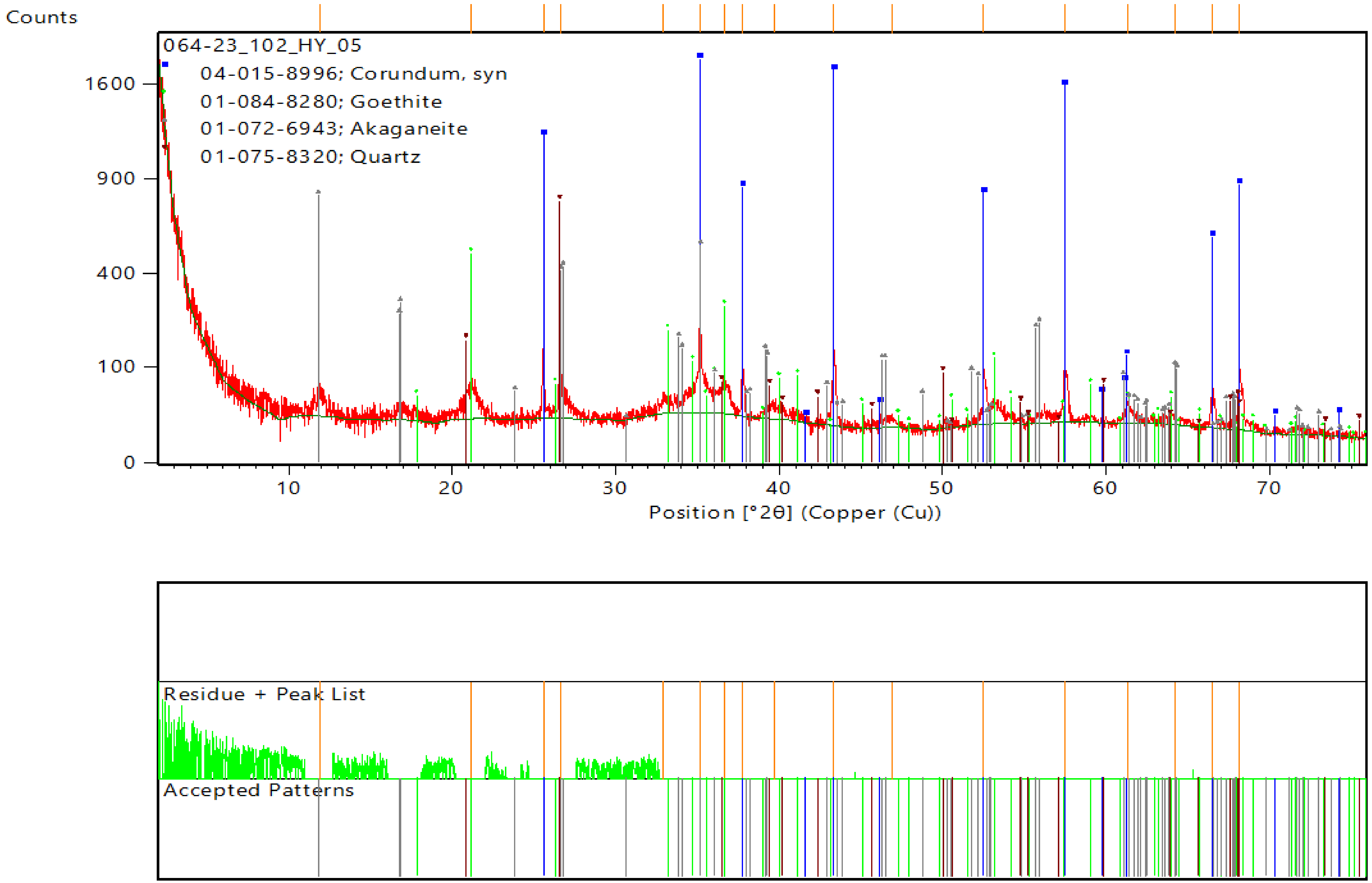
Counts
064-23_102_HY_05
04-015-8996; Corundum, syn
01-084-8280; Goethite
01-072-6943; Akaganeite
01-075-8320; Quartz
1600
900
400
100
0
10
20
30
40
50
60
70
Position [°2θ] (Copper (Cu))
Residue + Peak List
Accepted Patterns

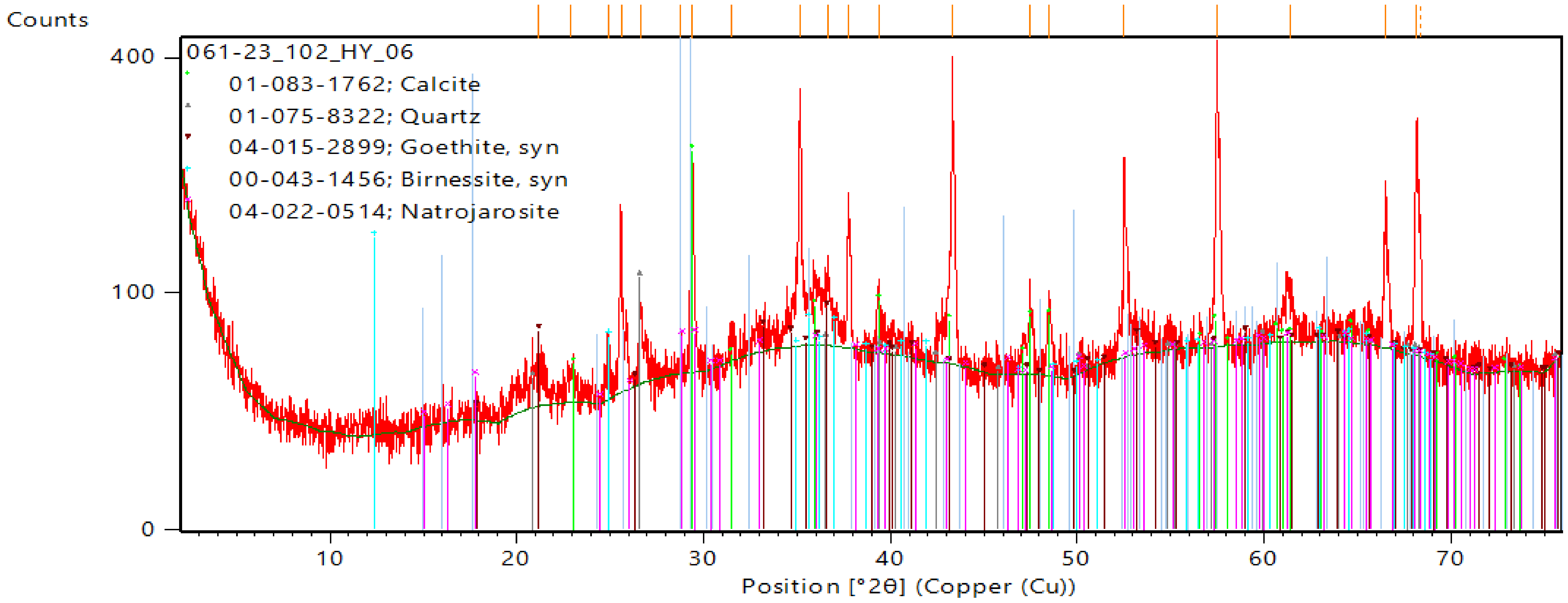
Counts
400
100
0
061-23_102_HY_06
01-083-1762; Calcite
01-075-8322; Quartz
04-015-2899; Goethite, syn
00-043-1456; Birnessite, syn
04-022-0514; Natrojarosite
10
20
30
40
50
60
70
Position [°2θ] (Copper (Cu))

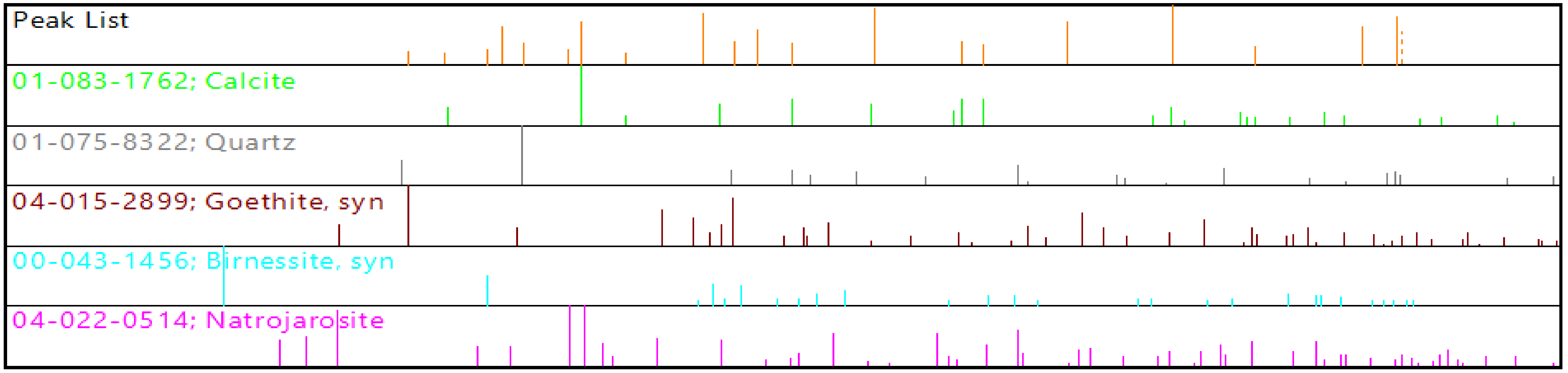
Peak List
01-083-1762; Calcite
01-075-8322; Quartz
04-015-2899; Goethite, syn
00-043-1456; Birnessite, syn
04-022-0514; Natrojarosite

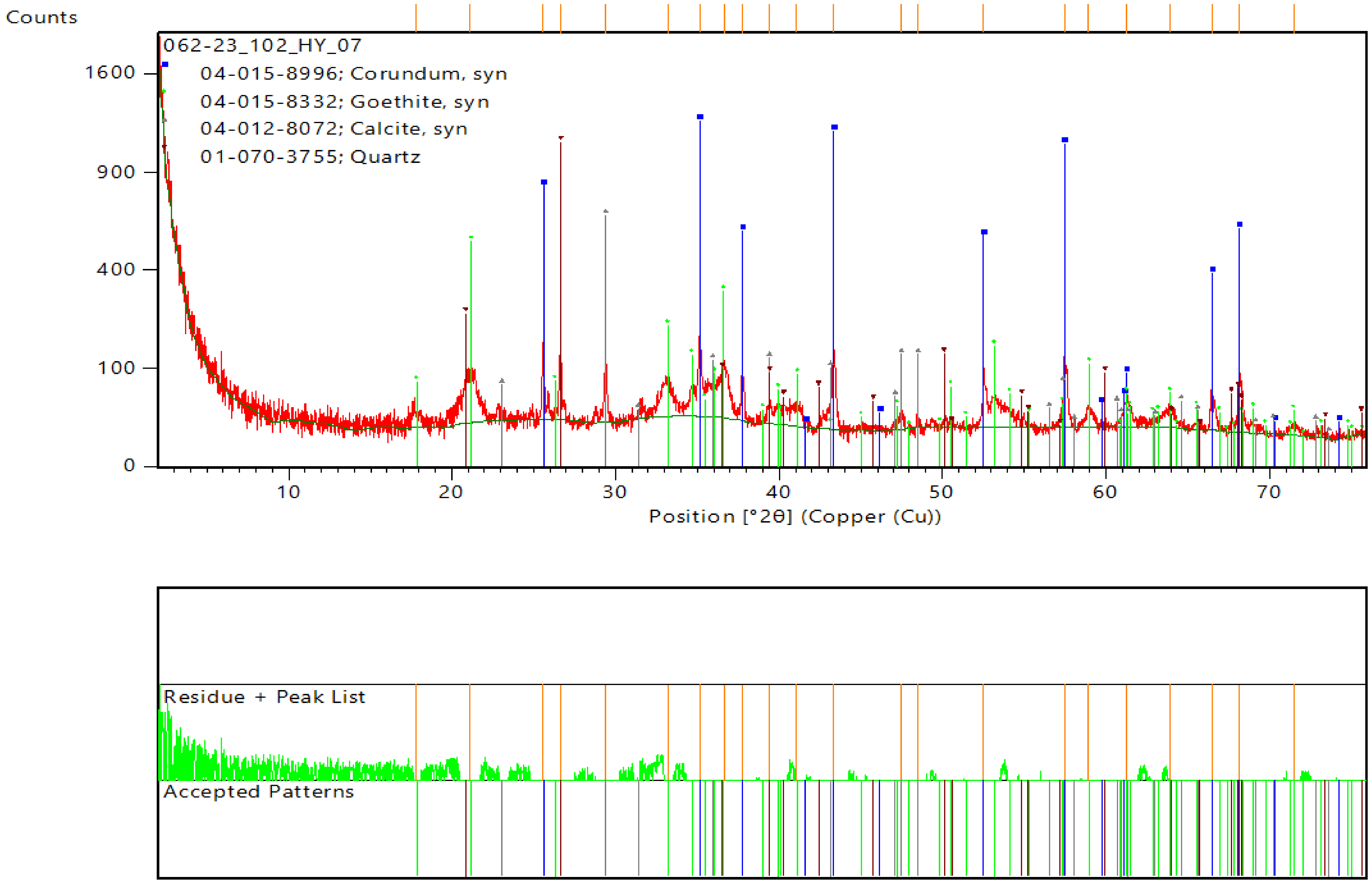
Counts
062-23_102_HY_07
04-015-8996; Corundum, syn
04-015-8332; Goethite, syn
04-012-8072; Calcite, syn
01-070-3755; Quartz
1600
900
400
100
0
10
20
30
40
50
60
70
Position [°2θ] (Copper (Cu))
Residue + Peak List
Accepted Patterns
