# Supplementary material for: Fe-oxyhydroxide deposits at Semenov hydrothermal field (13°30′N), Mid-Atlantic ridge: insights into formation, modification and resource potential
Source: Miner Depos. 2025 Aug 1;61(2):257–79. doi: 10.1007/s00126-025-01376-6 (PMC12858624; doi:10.1007/s00126-025-01376-6)
Supplement: Supplementary file 4 — Supplementary Material 4 (DOCX 268 KB) [file 126_2025_1376_MOESM4_ESM.docx]

# Electronic Supplementary Materials 2

Fe-oxyhydroxide deposits at Semenov Hydrothermal Field (13°30′N), Mid-Atlantic Ridge: insights into formation, modification and resource potentialMineralium Deposita
Authors - Christian Bishop^1^, Anna Lichtschlag^2^, Stephen Roberts^1^, Maxime Lesage^3^ & Bramley J. Murton^2^

^1^School of Ocean and Earth Science, University of Southampton, Southampton, UK, ^2^National Oceanography Centre, Southampton, UK, ^3^Nedre Slottsgate 8 - 0157 Oslo, Norway

[csb1u21@soton.ac.uk](mailto:csb1u21@soton.ac.uk) & [chris.star.bish@gmail.com](mailto:chris.star.bish@gmail.com)

ORCiD - 0000-0003-0652-3008

Electronic supplementary materials 2

## ESM2.1: Full acid digestion methods

For digestion, 50 mg of powdered sample was placed in a Teflon pot with 15 drops of sub boiled acid (6 M nitric acid (HNO3)) to prevent the sample from escaping the pots due to static. This was followed by adding 2 mL of Romil SpA grade hydrofluoric acid (HF), closing the Teflon pot and left 24 hours on a hot plate at 130^o^C. Following this, the Teflon pots were opened and dried out, and were then re-digested with sufficient (ca 5 mL) 6 M hydrochloric acid (HCl) with the Teflon pots closed and left overnight on a hot plate at 130^o^C. Following this, the Teflon pots were opened and left to dry out. This step was repeated to ensure no more HF was in the solution, since this could form insoluble salts. Dissolved samples were transferred to pre-weighed, high-density polyethylene scintillation vials and made up to ~20 mL using an approximate 50:50 mixture of 6 M HCl and Milli-Q® to form an approximate 400-fold diluted mother solution.

Solutions were dried on a hotplate at 130^o^C to incipient dryness using 1 mL of mother solution. The samples were made to 4000-fold dilution with 3% HNO3 containing an internal spike of Ru-Re at 5 ppb and Be at 20 ppb to monitor drift for Inductively Couple Plasma Mass Spectrometry (ICP-MS). To assess drift in the Inductively Coupled Plasma - Optical emission spectrum spectrometry (ICP-OES), after every ten samples a known chemical standard is analysed again – a drift standard. Measurements were calibrated against synthetic standards of known concentrations. In addition, sample blanks (spiked 3% HNO3) and sample duplicates were run and accuracy and reproducibility were determined by using triplicates of certified raw material (CRM; JMn-1, RTS-1, GSPN-2) were analysed at semi-regular intervals throughout the sampling process. The CRM standard triplicates were run for monitoring accuracy and reproducibility throughout the analysis; a further four CRM rock standards were measured once (GSPN-3, CH-4, Nod-A-1 and Nod-P-1) to ascertain the accuracy of the data.

## ESM2.2: Data verification of bulk geochemical composition

**Table S2.1** Average of measure trace element concentrations of rock standards GSPN-2 & JMn-1. %RE – percentage relative error with respect to certified/recommended published values. %RSD – percentage relative standard deviation around the mean of the measurements. ^a^ Elements with only duplicate measurements with respect to GSPN-2.

|  | GSPN-2 | | | | JMn-1 | | | |  |
| --- | --- | --- | --- | --- | --- | --- | --- | --- | --- |
| Wt.% | Average (n=3) | %RSD | Reported | %RE | Average (n=3) | %RSD | Reported | %RE | |
| Na | **1.83** | 1.4 | **1.90** | 3.8 | **2.05** | 3.0 | **2.08** | 1.1 | |
| Mg^a^ | **1.59** | 1.6 | **1.83** | 12.9 | **1.74** | 2.9 | **1.88** | 7.6 | |
| Al | **2.28** | 4.1 | **2.75** | 17.2 | **1.97** | 6.2 | **2.27** | 13.5 | |
| Ca | **1.80** | 0.7 | **1.91** | 5.5 | **2.07** | 1.2 | **2.08** | 0.2 | |
| Mn | **22.65** | 1.6 | **24.70** | 8.3 | **23.60** | 1.3 | **25.63** | 7.9 | |
| Fe^a^ | **9.86** | 0.9 | **10.87** | 9.3 | **9.74** | 3.1 | **10.07** | 3.3 | |
| Ni^a^ | **0.96** | 1.3 | **1.02** | 6.2 | **1.20** | 9.5 | **1.26** | 5.1 | |
| Cu | **0.66** | 5.9 | **0.69** | 4.9 | **0.99** | 7.1 | **1.11** | 11.0 | |
| ppm | Average (n=3) | %RSD | Reported | %RE | Average (n=3) | %RSD | Reported | %RE | |
| Li | **68** | 7.3 | **78** | 12.4 | **58** | 7.6 | **66** | 12.6 | |
| Sc | **15** | 8.4 | **13** | -16.3 | **12.1** | 6.4 | **13.0** | 6.7 | |
| Ti^a^ | **6610** | 7.9 | **8210** | 19.6 | **5440** | 10.5 | **6360** | 14.5 | |
| Co | **2316** | 9.7 | **2900** | 20.1 | **1450** | 7.2 | **1732** | 16.3 | |
| Zn | **820** | 6.5 | **918** | 10.6 | **950** | 3.2 | **1070** | 10.6 | |
| As | **97** | 9.5 | **105** | 7.7 | **69** | 7.4 | **75** | 8.0 | |
| Sr | **847** | 2.7 | **869** | 2.5 | **789** | 2.9 | **792** | 0.4 | |
| Nb | **39** | 5.1 | **48** | 18.3 | **23** | 4.5 | **22.3 - 33** | 0.0 | |
| Cd | **8.3** | 7.5 | **10.0** | 17.4 | **12.8** | 5.2 | **13.5** | 5.3 | |
| Sb | **27.6** | 1.5 | **31.0** | 11.1 | **30.56** | 3.0 | **37.50** | 18.5 | |
| Ba | **1520** | 1.3 | **1800** | 15.6 | **1390** | 3.5 | **1710** | 18.6 | |
| La | **156** | 6.6 | **184** | 15.4 | **102** | 3.8 | **122** | 16.0 | |
| Ce | **549** | 6.7 | **620** | 11.5 | **233** | 2.9 | **277** | 15.9 | |
| Pr | **44** | 5.3 | **49** | 9.9 | **28** | 4.0 | **31** | 10.0 | |
| Nd | **184** | 5.6 | **198** | 7.2 | **115** | 2.2 | **137** | 15.8 | |
| Sm | **43** | 5.1 | **46** | 5.5 | **26.5** | 1.8 | **30.2** | 12.4 | |
| Eu | **10.5** | 7.1 | **11.0** | 4.3 | **6.50** | 3.4 | **7.60** | 14.4 | |
| Gd | **45** | 4.5 | **48** | 6.9 | **28.2** | 0.9 | **29.8** | 5.4 | |
| Tb | **6.6** | 3.7 | **7.6** | 12.6 | **4.19** | 0.5 | **4.81** | 12.9 | |
| Dy | **41** | 4.1 | **42** | 2.8 | **26.6** | 0.9 | **28.3** | 5.9 | |
| Ho | **7.4** | 3.8 | **8.2** | 9.3 | **5.01** | 0.7 | **5.76** | 12.9 | |
| Er | **20** | 4.1 | **20** | 3.8 | **13.9** | 0.5 | **14.6** | 4.9 | |
| Tm | **2.9** | 4.5 | **3.1** | 6.3 | **1.98** | 0.4 | **2.14** | 7.5 | |
| Yb | **19** | 6.4 | **19** | 3.6 | **13.01** | 3.4 | **13.80** | 5.7 | |
| Lu | **2.9** | 3.8 | **2.9** | -1.4 | **1.98** | 0.5 | **2.07** | 4.5 | |
| W | **72** | 6.2 | **67** | -7.1 | **39** | 8.1 | **45** | 13.6 | |
| Pb | **652** | 1.3 | **709** | 8.0 | **413** | 3.6 | **430** | 4.1 | |
| Bi | **15.8** | 1.8 | **15.0** | -5.6 | **4.73** | 2.1 | **4** | -9.0 | |
| Th | **23.8** | 3.2 | **26.0** | 8.6 | **11.5** | 6.2 | **11.7** | 1.4 | |
| U | **6.8** | 3.6 | **6.2** | -10.0 | **5.30** | 7.2 | **5.00** | -4.8 | |

**Table S2.2** Average of measure trace element concentrations of rock standards RTS-1. %RE – percentage relative error with respect to certified/recommended published values. %RSD – percentage relative standard deviation around the mean of the measurements. ^a^ Elements with only duplicate measurements with respect to GSPN-2. Values with * indicate provisional values and values with ** indicate informational values.

|  | RTS-1 | | | |
| --- | --- | --- | --- | --- |
| Wt.% | Average (n=3) | %RSD | Reported | %RE |
| Na | **0.66** | 21.7 | **0.5**** | -31.9 |
| Mg^a^ | **2.69** | 3.1 | **2.67** | -0.7 |
| Al | **3.85** | 2.7 | **4.26*** | 9.7 |
| Ca | **3.20** | 7.5 | **2.67** | -19.7 |
| Mn | **0.26** | 6.5 | **0.19**** | -36.6 |
| Fe^a^ | **18.05** | 2.5 | **19.64*** | 8.1 |
| ppm | Average (n=3) | %RSD | Reported | %RE |
| Cu | 719 | 14.3 | 600* | -20.8 |
| Co | **12** | 22.2 | **17** | 25.2 |
| Ni | 17.0 | 35.2 | 22* | 20.8 |
| Ti^a^ | **4140** | 13.1 | **4000**** | -3.5 |
| Zn | **520** | 14.8 | **553** | 6.1 |
| As | **10** | 26.5 | **8.2*** | -20.3 |
| Sr | **72** | 17.9 | **60**** | -20.2 |
| Cd | **0.4** | 59.2 | **2**** | 78.2 |
| Ba | 119 | 34.1 | 123** | 3.2 |
| Pb | **102** | 16.1 | **105*** | 2.2 |
| Bi | 86 | 1.3 | 81** | -6.4 |

**Table S2.3** Average of measure trace element concentrations of rock standards CH-4 & Nod-P-1. %RE – percentage relative error with respect to certified/recommended published values. %RSD – percentage relative standard deviation around the mean of the measurements. Values with * indicate provisional values and values with ** indicate informational values.

|  | CH-4 | | | | Nod-P-1 | | | |
| --- | --- | --- | --- | --- | --- | --- | --- | --- |
| Wt.% | Measured | %RSD | Reported | %RE | Measured | %RSD | Reported | %RE |
| Na | **3.61** | 0.7 | **0.87*** | -317.4 | **1.78** | 0.5 | **1.71** | -4.0 |
| Mg | N/A | N/A | N/A | N/A | **2.03** | 11.4 | **2.03** | 0.2 |
| Al | **8.38** | 7.1 | **7.73*** | -8.5 | **2.49** | 9.4 | **2.46** | -1.0 |
| Ca | **2.19** | 1.0 | **1.96*** | -12.0 | **2.38** | 0.7 | **2.24** | -6.1 |
| Mn | **0.05** | 5.0 | **0.04*** | -6.8 | **27.68** | 0.1 | **29.60** | 6.5 |
| Fe | N/A | N/A | N/A | N/A | **6.13** | 6.6 | **5.89** | -4.1 |
| Ni | N/A | N/A | N/A | N/A | **1.41** | 11.0 | **1.34** | -5.4 |
| Cu | **0.23** | 0.4 | **0.20** | -17.3 | **1.19** | 0.1 | **1.15** | -3.2 |
| ppm | Measured | %RSD | Reported | %RE | Measured | %RSD | Reported | %RE |
| Li | **12.8** | 0.7 | **12.0** | -6.9 | **130** | 2.7 | **140** | 6.9 |
| Sc | **10.9** | 5.6 | **13.0** | 16.4 | **10** | 6.6 | **10** | -3.5 |
| Ti | N/A | N/A | N/A | N/A | **2720** | 2.6 | **2720** | 0.0 |
| Co | **21** | 1.8 | **26** | 19.0 | **1940** | 1.7 | **2240** | 13.4 |
| Zn | **191** | 2.3 | **200** | 4.7 | **1414** | 2.2 | **1600** | 11.6 |
| As | **8.8** | 0.9 | **8.8*** | 0.1 | **76.7** | 0.8 | **88.5** | 13.4 |
| Sr | **223** | 0.4 | **209**** | -6.7 | **665** | 0.3 | **680** | 2.3 |
| Nb | **3.5** | 4.1 | **4**** | 12.3 | **18.3** | 2.5 | **21.3** | 14.0 |
| Cd | **1.03** | 3.5 | **1** | 9.9 | **20.6** | 1.3 | **22.6** | 8.7 |
| Sb | **0.9** | 10.1 | **0.77*** | -12.5 | **49.4** | 1.7 | **49.4** | 0.1 |
| Ba | **455** | 2.0 | **425**** | -7.0 | **2455** | 2.3 | **3350** | 26.7 |
| La | **14.9** | 0.7 | **16**** | 6.7 | **98** | 1.5 | **104**** | 6.1 |
| Ce | **31.1** | 0.8 | **35**** | 11.2 | **294** | 1.6 | **290**** | -1.5 |
| Pr | **3.72** | 1.0 | **4**** | 6.9 | **30.2** | 1.3 | **31.0** | 2.5 |
| Nd | **14.59** | 0.6 | **16**** | 8.8 | **127** | 1.2 | **120**** | -5.6 |
| Sm | **2.97** | 1.4 | **3**** | 1.1 | **31.2** | 1.4 | **30.0** | -4.0 |
| Eu | **0.65** | 5.4 | **0.7**** | 6.6 | **7.4** | 1.8 | **7.5** | 1.7 |
| Gd | **2.7** | 0.6 | **3**** | 10.0 | **30.4** | 0.6 | **28.0** | -8.7 |
| Tb | **0.37** | 1.5 | **0.4**** | 7.3 | **4.53** | 0.6 | **4.90** | 7.6 |
| Dy | **2.33** | 1.5 | **2**** | -16.4 | **27.3** | 0.8 | **27**** | -1.1 |
| Ho | **0.45** | 2.3 | **0.4**** | -11.3 | **4.95** | 0.7 | **5.00** | 1.0 |
| Er | **1.25** | 0.9 | **1.2**** | -4.4 | **13.4** | 1.5 | **13.6** | 1.3 |
| Tm | **0.19** | 4.2 | **0.2**** | 7.0 | **1.93** | 0.6 | **1.90** | -1.5 |
| Yb | **1.21** | 3.4 | **1**** | -21.4 | **12.6** | 2.2 | **13**** | 3.2 |
| Lu | **0.19** | 1.8 | **0.2**** | 4.8 | **1.93** | 1.4 | **1.8**** | -7.3 |
| W | **5.8** | 1.3 | **3**** | -94.0 | **67** | 2.2 | **58** | -15.4 |
| Pb | **17.5** | 1.3 | **14**** | -25.1 | **460** | 0.9 | **560** | 17.9 |
| Bi | **0.61** | 3.2 | **0.6**** | -1.7 | **5.49** | 0.8 | **5.80** | 5.3 |
| Th | **2.47** | 2.0 | **2**** | -23.3 | **16.27** | 0.9 | **16.70** | 2.5 |
| U | **0.85** | 2.8 | **0.7**** | -21.7 | **4.6** | 1.2 | **4.0** | -14.9 |

**Table S2.4** Average of measure trace element concentrations of rock standards CH-4 & Nod-P-1. %RE – percentage relative error with respect to certified/recommended published values. %RSD – percentage relative standard deviation around the mean of the measurements. Values with ** indicate informational values.

|  | Nod-A-1 | | | | GSPN-3 | | | |
| --- | --- | --- | --- | --- | --- | --- | --- | --- |
| Wt.% | Measured | %RSD | Reported | %RE | Measured | %RSD | Reported | %RE |
| Na | **0.88** | 1.2 | **0.74** | -19.1 | **3.20** | 0.9 | **2.25** | -42.2 |
| Mg | **2.87** | 6.0 | **2.87** | 0.1 | **1.99** | 6.4 | **2.15** | 7.2 |
| Al | **2.03** | 7.2 | **2.05** | 0.8 | **2.21** | 9.4 | **2.49** | 11.1 |
| Ca | **11.66** | 0.2 | **11.01** | -5.9 | **2.23** | 1.4 | **1.61** | -38.5 |
| Mn | **18.99** | 0.3 | **18.30** | -3.8 | **29.21** | 0.2 | **32.20** | 9.3 |
| Fe | **11.44** | 3.8 | **10.91** | -4.8 | **4.49** | 6.1 | **4.70** | 4.4 |
| Ni | **0.68** | 8.3 | **0.64** | -6.2 | **1.57** | 8.4 | **1.55** | -1.3 |
| Cu | **0.12** | 0.3 | **0.11** | -6.0 | **1.37** | 0.7 | **1.36** | -1.0 |
| ppm | Measured | %RSD | Reported | %RE | Measured | %RSD | Reported | %RE |
| Li | **63.6** | 0.8 | **76.1** | 16.4 | **196** | 1.5 | **205** | 4.2 |
| Sc | **10.8** | 4.5 | **12.4** | 13.1 | **9.6** | 5.3 | **9.4** | -1.8 |
| Ti | **2920** | 0.3 | **3177** | 8.0 | **2950** | 0.3 | **3237** | 8.9 |
| Co | **2567** | 1.0 | **3110** | 17.5 | **1375** | 2.6 | **1700** | 19.1 |
| Zn | **509** | 1.1 | **590** | 13.8 | **1400** | 2.6 | **1600** | 12.2 |
| As | **258** | 1.0 | **310** | 16.9 | **52.8** | 0.8 | **53.0** | 0.4 |
| Sr | **1560** | 1.2 | **1750** | 10.9 | **613** | 0.6 | **561** | -9.3 |
| Nb | **38.2** | 1.3 | **43.1** | 11.4 | **17.1** | 2.0 | **21.0** | 18.8 |
| Cd | **6.7** | 1.9 | **6.8** | 1.2 | **19.9** | 1.1 | **23.0** | 13.4 |
| Sb | **33.3** | 2.0 | **33.8** | 1.4 | **46.2** | 2.6 | **46.0** | -0.5 |
| Ba | **1394** | 0.5 | **1670** | 16.5 | **2220** | 1.7 | **2400** | 7.6 |
| La | **100.2** | 0.5 | **112.0** | 10.5 | **84.6** | 1.0 | **96.0** | 11.8 |
| Ce | **676** | 1.5 | **743** | 9.0 | **232** | 1.1 | **249** | 6.9 |
| Pr | **22.6** | 0.6 | **24.3** | 6.8 | **27.7** | 0.7 | **29.0** | 4.3 |
| Nd | **94.1** | 0.9 | **93.0** | -1.2 | **116** | 0.9 | **121** | 4.2 |
| Sm | **21.1** | 0.6 | **21**** | -0.4 | **29.3** | 0.6 | **31.0** | 5.4 |
| Eu | **5.00** | 2.3 | **5**** | -0.1 | **7.0** | 1.4 | **7.6** | 7.5 |
| Gd | **24.9** | 0.6 | **24.9** | 0.0 | **28.6** | 0.8 | **28.0** | -2.2 |
| Tb | **3.62** | 0.5 | **3.90** | 7.1 | **4.4** | 0.7 | **4.6** | 4.3 |
| Dy | **23.7** | 0.4 | **23.5** | -0.7 | **26.7** | 0.6 | **27.0** | 1.0 |
| Ho | **4.82** | 0.4 | **4.90** | 1.6 | **4.8** | 1.3 | **5.1** | 5.9 |
| Er | **14.1** | 0.5 | **14.6** | 3.3 | **13.0** | 0.9 | **13.0** | -0.1 |
| Tm | **2.06** | 1.2 | **2.09** | 1.3 | **1.89** | 1.4 | **1.90** | 0.3 |
| Yb | **13** | 2.3 | **14** | 2.8 | **12.3** | 2.2 | **12.0** | -2.6 |
| Lu | **2.17** | 1.0 | **2.20** | 1.2 | **1.87** | 1.4 | **1.80** | -4.0 |
| W | **93** | 1.3 | **87** | -7.4 | **74** | 2.1 | **61** | -21.3 |
| Pb | **850** | 1.8 | **846** | -0.4 | **307** | 0.3 | **328** | 6.5 |
| Bi | **10.7** | 1.6 | **10.2** | -5.2 | **6.5** | 1.0 | **5.0** | -29.2 |
| Th | **23.9** | 1.7 | **23.4** | -2.1 | **15.6** | 1.1 | **15.0** | -4.0 |
| U | **7.9** | 1.9 | **7.0** | -12.7 | **4.25** | 0.8 | **3.80** | -11.9 |

**Table S2.5** Comparison of measured and published Pb isotope systematics for certified reference material. ± indicates two standard error.

|  | ^206^Pb/^204^Pb | | ^207^Pb/^204^Pb | | ^208^Pb/^204^Pb | |
| --- | --- | --- | --- | --- | --- | --- |
|  | Measured | Published | Measured | Published | Measured | Published |
| Nod-A-1 | 18.9629 ± 0.0002 | 18.957 - 18.964 | 15.681 ± 0.0011 | 15.675 - 15.685 | 38.94 ± 0.0101 | 38.929 - 39.9518 |
| Nod-P-1 | 18.7033 ± 0.0002 | 18.697 - 18.7081 | 15.6356 ± 0.0008 | 15.629 - 15.638 | 38.681 ± 0.0020 | 38.669 - 38.6994 |

**Table S2.6** Comparison of measure and published Nd and Sr isotope systematics for certified reference material. ± indicates two standard error.

|  | 87Sr/86Sr | | ^143^Nd/^144^Nd | |
| --- | --- | --- | --- | --- |
|  | Measured | Published | Measured | Published |
| Nod-A-1 | 0.7092333 ± 0.000005 | N/A | 0.512153 ± 0.000009 | 0.51213 - 0.512148 |
| Nod-P-1 | 0.709279 ±  0.000006 | N/A | 0.512443 ± 0.000008 | 0.51242 - 0.51455 |
| BHVO-1 | 0.703496 ± 0.000006 | 0.70337 - 0.703603 | 0.512993 ± 0.000008 | 0.5129 – 0.513049 |

## ESM2.3: Geochemical composition of FeOOH and sulphide

**Table S2.7** Contents of major and trace elements in FeOOH and massive sulphide samples from Semyenov hydrothermal field. Where Mn is >1000 ppm (0.1 wt.%), value was obtained by ICP-OES, below is measured by ICP-MS. Other data obtained by ICP-MS. b.d.l – below detection limit. Table 1 of 2.

|  | **Na (wt.%)** | | **Mg** | | **Al** | | **Ca** | | **Mn** | | **Fe** | | **Cu** | | **Li (ppm)** | | **Sc** | | **Ti** | | **Co** | | **Ni** | | **Zn** | | **As** | | **Sr** | | **Nb** | | **Cd** | | **Sb** | | **Ba** | | **W** | |
| --- | --- | --- | --- | --- | --- | --- | --- | --- | --- | --- | --- | --- | --- | --- | --- | --- | --- | --- | --- | --- | --- | --- | --- | --- | --- | --- | --- | --- | --- | --- | --- | --- | --- | --- | --- | --- | --- | --- | --- | --- |
| 28_DR_03 | 1.33 | | 0.62 | | 0.12 | | 0.77 | | 3.66 | | 28.99 | | 0.48 | | 15.7 | | 1.1 | | 80 | | 295 | | 40 | | 2500 | | 260 | | 370 | | 0.6 | | 1.1 | | 9.3 | | 980 | | 2.3 | |
| 28_DR_04 | 1.00 | | 0.29 | | 0.12 | | 0.22 | | 0.02 | | 29.30 | | 4.18 | | 1.2 | | 0.5 | | 40 | | 14 | | b.d.l | | 820 | | 260 | | 890 | | 0.1 | | 0.3 | | 11.8 | | 10300 | | 1.35 | |
| 28_DR_05 | 1.04 | | 0.28 | | 0.01 | | 0.20 | | 0.03 | | 37.29 | | 0.08 | | 1.5 | | 0.1 | | <10 | | 5 | | b.d.l | | 2120 | | 160 | | 100 | | <0.1 | | 0.3 | | 37.8 | | 40 | | 0.67 | |
| 28_DR_18 | 1.22 | | 0.26 | | 0.14 | | 0.81 | | 0.01 | | 31.99 | | 3.77 | | 1.4 | | 0.6 | | 40 | | 5 | | b.d.l | | 970 | | 290 | | 680 | | 0.2 | | 0.5 | | 11.5 | | 10040 | | 1.60 | |
| 39_HY_02 | 1.48 | | 1.05 | | 0.32 | | 0.67 | | 7.51 | | 20.48 | | 0.14 | | 102 | | 2.1 | | 240 | | 111 | | 450 | | 840 | | 200 | | 480 | | 2.2 | | 2.7 | | 26.8 | | 9580 | | 3.5 | |
| 66_HY_05 | 0.82 | | 0.75 | | 0.15 | | 0.72 | | 0.19 | | 29.99 | | 2.48 | | 3.9 | | 1.2 | | 120 | | 309 | | 40 | | 1060 | | 240 | | 270 | | 1.0 | | 0.6 | | 8.2 | | 880 | | 1.85 | |
| 71_DR_01 | 1.76 | | 0.70 | | 0.19 | | 0.83 | | 11.58 | | 21.47 | | 0.16 | | 143 | | 0.9 | | 140 | | 59 | | 190 | | 1370 | | 100 | | 400 | | 0.9 | | 0.8 | | 5.8 | | 2930 | | 1.26 | |
| 71_DR_02 | 1.83 | | 0.72 | | 0.12 | | 0.70 | | 7.23 | | 20.53 | | 0.06 | | 128 | | 0.3 | | 60 | | 9 | | 50 | | 630 | | 57 | | 230 | | 0.3 | | 0.6 | | 3.8 | | 290 | | 0.82 | |
| 71_DR_03 | 1.7 | | 0.67 | | 0.14 | | 0.75 | | 9.29 | | 20.24 | | 0.25 | | 104 | | 0.4 | | 80 | | 63 | | 100 | | 940 | | 65 | | 290 | | 0.4 | | 0.6 | | 3.8 | | 1150 | | 0.92 | |
| 71_DR_04 | 1.03 | | 0.88 | | 0.23 | | 1.18 | | 0.06 | | 36.46 | | 0.20 | | 11.3 | | 1.3 | | 110 | | 15 | | 10 | | 1150 | | 300 | | 530 | | 1.2 | | 0.9 | | 13.1 | | 6810 | | 3.8 | |
| 71_DR_07 | 1.62 | | 0.78 | | 0.12 | | 0.61 | | 4.74 | | 22.29 | | 0.06 | | 72 | | 0.4 | | 60 | | 9 | | 40 | | 600 | | 59 | | 190 | | 0.3 | | 0.4 | | 2.7 | | 590 | | 0.61 | |
| 71_DR_09 | 1.42 | | 0.93 | | 0.59 | | 1.50 | | 1.07 | | 31.82 | | 0.19 | | 5.5 | | 7.0 | | 420 | | 119 | | 80 | | 770 | | 970 | | 550 | | 5.4 | | 0.6 | | 22.0 | | 7530 | | 8.4 | |
| 71_DR_12 | 1.41 | | 0.70 | | 0.26 | | 1.01 | | 5.98 | | 24.84 | | 0.16 | | 90 | | 0.9 | | 160 | | 35 | | 50 | | 670 | | 160 | | 420 | | 1.2 | | 0.7 | | 6.7 | | 7720 | | 1.92 | |
| 77_HY_02 | 2.58 | | 0.69 | | 0.46 | | 1.53 | | 2.48 | | 24.64 | | 0.87 | | 3.0 | | 1.2 | | 180 | | 139 | | 10 | | 290 | | 160 | | 490 | | 0.9 | | 0.5 | | 3.8 | | 5370 | | 4.1 | |
| 77_HY_03 | 1.47 | | 2.05 | | 1.00 | | 0.56 | | 1.38 | | 18.65 | | 6.90 | | 7.1 | | 2.5 | | 510 | | 59 | | b.d.l | | 130 | | 170 | | 1180 | | 2.0 | | 0.2 | | 3.5 | | 9760 | | 2.3 | |
| 82_HY_01 | 0.61 | | 0.63 | | 0.24 | | 1.00 | | 0.62 | | 34.68 | | 1.84 | | 3.6 | | 1.4 | | 330 | | 38 | | 120 | | 310 | | 330 | | 210 | | 1.7 | | 0.6 | | 23.7 | | 390 | | 2.2 | |
| 82_HY_05 | 2.17 | | 0.87 | | 0.22 | | 1.41 | | 0.04 | | 31.38 | | 1.96 | | 4.0 | | 1.4 | | 170 | | 10 | | 50 | | 380 | | 360 | | 300 | | 0.6 | | 0.7 | | 17.1 | | 1630 | | 2.6 | |
| 82_HY_06a | 0.55 | | 0.18 | | 0.04 | | 0.22 | | <0.01 | | 30.01 | | 16.79 | | 0.6 | | 0.2 | | <10 | | 4 | | b.d.l | | 910 | | 160 | | 80 | | <0.1 | | 1.3 | | 14.4 | | 50 | | 0.45 | |
| 82_HY_06b | 0.07 | | 0.01 | | 0.01 | | 0.01 | | <0.01 | | 34.65 | | 0.37 | | 0.1 | | 0.1 | | 10 | | 29 | | b.d.l | | 4370 | | 750 | | 0 | | <0.1 | | 11.9 | | 9.9 | | 10 | | 1.21 | |
| 82_HY_09 | 0.60 | | 0.87 | | 0.22 | | 0.44 | | 0.44 | | 37.90 | | 0.30 | | 7.2 | | 3.7 | | 270 | | 263 | | 50 | | 1100 | | 810 | | 120 | | 4.3 | | 1.0 | | 30.7 | | 100 | | 5.0 | |
| 82_HY_10 | 1.12 | | 0.98 | | 0.62 | | 1.92 | | 0.56 | | 28.73 | | 1.73 | | 5.5 | | 2.9 | | 480 | | 63 | | 160 | | 560 | | 460 | | 320 | | 2.5 | | 0.5 | | 19.4 | | 1180 | | 4.5 | |
| 82_HY_11 | 0.61 | | 0.71 | | 0.26 | | 0.55 | | 0.29 | | 34.52 | | 0.29 | | 6.7 | | 2.2 | | 130 | | 53 | | 90 | | 1610 | | 460 | | 240 | | 1.3 | | 1.0 | | 17.4 | | 10010 | | 3.1 | |
| 86_HY_02 | 0.05 | | 0.01 | | 0.09 | | 0.01 | | <0.01 | | 33.74 | | 0.11 | | 0.2 | | 0.1 | | 10 | | 29 | | b.d.l | | 280 | | 81 | | 20 | | 0.1 | | 0.5 | | 1.5 | | 290 | | 0.42 | |
| 86_HY_04 | 1.24 | | 0.57 | | 0.17 | | 0.80 | | 0.30 | | 29.32 | | 2.42 | | 0.9 | | 1.8 | | 50 | | 25 | | b.d.l | | 3820 | | 590 | | 330 | | 0.8 | | 0.6 | | 21.8 | | 7540 | | 4.9 | |
| 86_HY_07 | 0.11 | | 0.02 | | 0.09 | | 0.00 | | <0.01 | | 33.87 | | 2.01 | | 0.5 | | 0.1 | | 20 | | 494 | | b.d.l | | 610 | | 80 | | 10 | | 0.1 | | 0.8 | | 6.6 | | 200 | | 0.44 | |
|  | **Na (wt.%)** | **Mg** | | **Al** | | **Ca** | | **Mn** | | **Fe** | | **Cu** | | **Li (ppm)** | | **Sc** | | **Ti** | | **Co** | | **Ni** | | **Zn** | | **As** | | **Sr** | | **Nb** | | **Cd** | | **Sb** | | **Ba** | | **W** | |  |
| 86_HY_08 | 1.81 | 0.58 | | 0.02 | | 0.85 | | 7.21 | | 27.72 | | 0.04 | | 40 | | 0.1 | | 10 | | 1 | | b.d.l | | 2400 | | 130 | | 330 | | <0.1 | | 1.6 | | 9.6 | | 600 | | 2.4 | |  |
| 86_HY_09 | 1.2 | 0.66 | | 0.08 | | 0.55 | | 0.01 | | 33.15 | | 0.93 | | 1.1 | | 0.1 | | 10 | | 7 | | <10 | | 5520 | | 470 | | 840 | | 0.1 | | 1.0 | | 16.2 | | 12750 | | 3.1 | |  |
| 87_MC_01 | 1.24 | 0.62 | | 0.23 | | 1.04 | | 1.58 | | 31.07 | | 0.26 | | 9.8 | | 1.0 | | 130 | | 85 | | 250 | | 2430 | | 210 | | 370 | | 0.8 | | 0.5 | | 4.9 | | 170 | | 1.02 | |  |
| 90_HY_04a | 1.53 | 1.00 | | 0.11 | | 1.09 | | 9.15 | | 28.96 | | 0.40 | | 63 | | 0.6 | | 20 | | 36 | | 140 | | 1880 | | 460 | | 560 | | 0.2 | | 2.5 | | 27.1 | | 3540 | | 6.3 | |  |
| 90_HY_04b | 0.18 | 0.02 | | 0.01 | | 0.04 | | 0.01 | | 29.25 | | 15.23 | | 0.1 | | <0.1 | | <10 | | 2 | | b.d.l | | 2400 | | 30 | | 510 | | <0.1 | | 8.6 | | 11.4 | | 3250 | | 0.61 | |  |
| 90_HY_04c | 0.20 | 0.09 | | 0.01 | | 0.34 | | 0.01 | | 31.69 | | 6.03 | | 0.1 | | <0.1 | | <10 | | 3 | | b.d.l | | 1850 | | 130 | | 410 | | <0.1 | | 3.2 | | 10.4 | | 170 | | 0.39 | |  |
| 90_HY_05 | 0.71 | 0.46 | | 0.29 | | 0.72 | | 0.87 | | 35.84 | | 0.35 | | 2.2 | | 2.7 | | 440 | | 140 | | 70 | | 1110 | | 200 | | 180 | | 3.2 | | 0.4 | | 16.1 | | 2170 | | 5.2 | |  |
| 90_HY_06 | 1.03 | 0.35 | | 0.48 | | 3.08 | | 1.36 | | 36.51 | | 0.36 | | 3.1 | | 3.0 | | 230 | | 148 | | 40 | | 800 | | 180 | | 380 | | 5.0 | | 0.4 | | 16.0 | | 4560 | | 6.5 | |  |
| 94_HY_01 | 2.11 | 1.11 | | 1.75 | | 0.38 | | 1.31 | | 20.71 | | 1.04 | | 10.2 | | 4.2 | | 1250 | | 188 | | 90 | | 2640 | | 220 | | 250 | | 8.0 | | 0.9 | | 9.1 | | 7460 | | 2.9 | |  |
| 94_HY_04 | 1.03 | 0.58 | | 0.72 | | 0.61 | | 1.38 | | 26.26 | | 4.44 | | 2.2 | | 3.0 | | 790 | | 156 | | 70 | | 690 | | 320 | | 510 | | 1.3 | | 0.3 | | 12.0 | | 7890 | | 3.2 | |  |
| 94_HY_05 | 1.07 | 0.30 | | 0.17 | | 0.29 | | 0.40 | | 33.2 | | 8.87 | | 1.0 | | 0.7 | | 110 | | 104 | | b.d.l | | 460 | | 130 | | 80 | | 0.5 | | 0.8 | | 17.1 | | 370 | | 0.97 | |  |
| 94_HY_06 | 0.77 | 0.35 | | 0.21 | | 0.58 | | 0.64 | | 31.9 | | 8.71 | | 1.0 | | 0.8 | | 130 | | 615 | | b.d.l | | 990 | | 200 | | 310 | | 0.4 | | 0.3 | | 9.0 | | 4150 | | 1.79 | |  |
| 102_HY_03 | 1.12 | 0.42 | | 0.34 | | 1.42 | | 1.24 | | 37.59 | | 0.29 | | 2.7 | | 2.4 | | 390 | | 163 | | 70 | | 2260 | | 190 | | 240 | | 2.9 | | 0.4 | | 41.8 | | 2160 | | 4.4 | |  |
| 102_HY_04 | 0.94 | 0.41 | | 0.16 | | 1.11 | | 0.74 | | 31.99 | | 2.01 | | 1.1 | | 0.9 | | 150 | | 74 | | 40 | | 1620 | | 250 | | 400 | | 0.9 | | 0.7 | | 5.1 | | 920 | | 1.6 | |  |
| 102_HY_05 | 0.69 | 0.30 | | 0.24 | | 0.43 | | 0.07 | | 35.84 | | 0.09 | | 1.1 | | 1.8 | | 50 | | 56 | | <10 | | 3530 | | 220 | | 200 | | 0.4 | | 0.5 | | 19.4 | | 1770 | | 1.2 | |  |
| 102_HY_06 | 1.76 | 0.87 | | 0.97 | | 5.48 | | 3.67 | | 26.14 | | 1.71 | | 5.7 | | 8.8 | | 1170 | | 261 | | 160 | | 1650 | | 470 | | 1010 | | 10.1 | | 0.8 | | 13.0 | | 5900 | | 10.9 | |  |
| 102_HY_07 | 1.37 | 0.49 | | 0.49 | | 3.18 | | 1.84 | | 31.59 | | 0.34 | | 3.6 | | 3.9 | | 530 | | 102 | | 80 | | 1160 | | 250 | | 570 | | 4.2 | | 0.4 | | 10.5 | | 10550 | | 7.9 | |  |

**Table S2.8** Contents of major and trace elements in FeOOH and massive sulphide samples from Semenov hydrothermal field. Where Mn is >1000 ppm (>1 wt.%), value was obtained by ICP-OES, below is measured by ICP-MS. Other data obtained by ICP-MS. <b.d.l – below detection limit. Table 2 of 2.

| **Sample** | **Pb (ppm)** | **Bi** | **Th** | **U** | **Sample** | | **Pb (ppm)** | | **Bi** | | **Th** | | **U** | |
| --- | --- | --- | --- | --- | --- | --- | --- | --- | --- | --- | --- | --- | --- | --- |
| 28_DR_03 | 32 | 0.11 | 0.30 | 6.6 | 86_HY_08 | | 150 | | 0.03 | | 0.01 | | 8.1 | |
| 28_DR_04 | 486 | 1.2 | 0.14 | 15.8 | 86_HY_09 | | 256 | | 0.29 | | 0.03 | | 8.3 | |
| 28_DR_05 | 606 | 0.05 | 0.01 | 15.2 | 87_MC_01 | | 4 | | 0.05 | | 0.57 | | 9.1 | |
| 28_DR_18 | 511 | 1.0 | 0.09 | 16.4 | 90_HY_04a | | 14 | | 0.05 | | 0.08 | | 6.9 | |
| 39_HY_02 | 51 | 0.20 | 1.40 | 2.0 | 90_HY_04b | | 98 | | 0.05 | | 0.01 | | 3.6 | |
| 66_HY_05 | 17 | 0.21 | 0.67 | 5.3 | 90_HY_04c | | 195 | | 0.05 | | <0.01 | | 4.4 | |
| 71_DR_01 | 39 | 0.09 | 0.85 | 1.9 | 90_HY_05 | | 204 | | 0.10 | | 1.63 | | 37.6 | |
| 71_DR_02 | 16 | 0.05 | 0.26 | 1.1 | 90_HY_06 | | 201 | | 0.16 | | 2.54 | | 36.1 | |
| 71_DR_03 | 9 | 0.05 | 0.33 | 1.3 | 94_HY_01 | | 96 | | 1.6 | | 3.32 | | 3.9 | |
| 71_DR_04 | 28 | 1.9 | 0.48 | 12.3 | 94_HY_04 | | 170 | | 3.1 | | 0.87 | | 10.6 | |
| 71_DR_07 | 12 | 0.03 | 0.27 | 0.9 | 94_HY_05 | | 339 | | 0.60 | | 0.46 | | 15.8 | |
| 71_DR_09 | 68 | 0.49 | 3.16 | 7.5 | 94_HY_06 | | 42 | | 5.3 | | 0.20 | | 32.3 | |
| 71_DR_12 | 19 | 0.52 | 0.78 | 4.9 | 102_HY_03 | | 467 | | 0.20 | | 1.75 | | 43.6 | |
| 77_HY_02 | 57 | 1.0 | 0.78 | 7.7 | 102_HY_04 | | 22 | | 0.08 | | 0.33 | | 8.2 | |
| 77_HY_03 | 284 | 2.2 | 1.51 | 7.5 | 102_HY_05 | | 250 | | 0.05 | | 0.12 | | 53.3 | |
| 82_HY_01 | 190 | 15.1 | 2.06 | 16.7 | 102_HY_06 | | 154 | | 0.49 | | 6.26 | | 8.4 | |
| 82_HY_05 | 258 | 6.9 | 0.74 | 8.4 | 102_HY_07 | | 369 | | 0.28 | | 2.78 | | 12.0 | |
| 82_HY_06a | 392 | 8.5 | 0.01 | 31.3 |  |  | |  | |  | |  | |  |
| 82_HY_06b | 300 | 1.7 | 0.04 | 2.3 |  |  | |  | |  | |  | |  |
| 82_HY_09 | 108 | 0.93 | 2.25 | 9.6 |  |  | |  | |  | |  | |  |
| 82_HY_10 | 142 | 7.7 | 2.10 | 8.9 |  |  | |  | |  | |  | |  |
| 82_HY_11 | 373 | 1.1 | 0.73 | 22.5 |  |  | |  | |  | |  | |  |
| 86_HY_02 | 58 | 1.0 | 0.12 | 3.0 |  |  | |  | |  | |  | |  |
| 86_HY_04 | 423 | 0.07 | 0.19 | 10.0 |  |  | |  | |  | |  | |  |
| 86_HY_07 | 131 | 5.1 | 0.17 | 7.1 |  |  | |  | |  | |  | |  |

## ESM2.4: REE content of FeOOH and massive sulphide at Semenov hydrothermal field

**Table S2.9** Contents of REE in FeOOH and massive sulphide samples collected at Semyenov hydrothermal field. Ce/Ce*= (Ce_N_)/((La_N_ x Nd_N_) x 0.5).Eu/Eu* = (Eu_N_)/((Sm_N_ x Gd_N_) x 0.5). X_n_ indicates that the element is normalised by carbonaceous chondrite after Barrat et al. (2012). LREE = La-Sm and HREE = Eu-Lu.

| Sample ID | La | Ce | Pr | Nd | Sm | Eu | Gd | Tb | Dy | Ho | Er | Tm | Yb | Lu | ΣLREE | ΣHREE | ΣREE | LREE/  HREE | Ce/Ce* | | Eu/Eu* | | La_n_/Yb_n_ | |  |
| --- | --- | --- | --- | --- | --- | --- | --- | --- | --- | --- | --- | --- | --- | --- | --- | --- | --- | --- | --- | --- | --- | --- | --- | --- | --- |
| 28_DR_03 | 7.9 | 7.1 | 1.82 | 7.7 | 1.61 | 0.51 | 1.90 | 0.26 | 1.76 | 0.36 | 1.04 | 0.15 | 0.98 | 0.150 | 26.1 | 7.10 | 33.2 | 3.67 | 0.45 | | 0.88 | | 5.79 | |  |
| 28_DR_04 | 2.8 | 2.0 | 0.54 | 2.4 | 0.71 | 1.27 | 0.70 | 0.10 | 0.63 | 0.13 | 0.41 | 0.06 | 0.43 | 0.066 | 8.41 | 3.78 | 12.2 | 2.22 | 0.40 | | 5.45 | | 4.71 | |  |
| 28_DR_05 | 1.2 | 0.6 | 0.15 | 0.6 | 0.12 | 0.08 | 0.16 | 0.02 | 0.13 | 0.03 | 0.09 | 0.01 | 0.07 | 0.013 | 2.78 | 0.60 | 3.38 | 4.68 | 0.36 | | 1.80 | | 12.95 | |  |
| 28_DR_18 | 2.8 | 2.6 | 0.63 | 2.8 | 0.86 | 1.29 | 0.88 | 0.13 | 0.80 | 0.17 | 0.51 | 0.07 | 0.53 | 0.080 | 9.67 | 4.46 | 14.1 | 2.17 | 0.48 | | 4.46 | | 3.73 | |  |
| 39_HY_02 | 15.3 | 26.6 | 3.82 | 15.8 | 3.30 | 1.39 | 3.45 | 0.49 | 3.10 | 0.60 | 1.71 | 0.24 | 1.61 | 0.239 | 64.8 | 12.8 | 77.6 | 5.05 | 0.85 | | 1.25 | | 6.78 | |  |
| 66_HY_05 | 7.7 | 11.3 | 1.75 | 7.2 | 1.53 | 0.53 | 1.76 | 0.25 | 1.70 | 0.35 | 1.03 | 0.15 | 0.99 | 0.154 | 29.4 | 6.93 | 36.4 | 4.25 | 0.75 | | 0.98 | | 5.54 | |  |
| 71_DR_01 | 7.9 | 12.7 | 1.88 | 7.7 | 1.62 | 0.77 | 1.72 | 0.24 | 1.54 | 0.30 | 0.82 | 0.11 | 0.74 | 0.106 | 31.8 | 6.34 | 38.1 | 5.01 | 0.80 | | 1.39 | | 7.58 | |  |
| 71_DR_02 | 2.0 | 2.8 | 0.51 | 2.2 | 0.45 | 0.34 | 0.48 | 0.07 | 0.43 | 0.09 | 0.24 | 0.03 | 0.23 | 0.033 | 7.98 | 1.94 | 9.92 | 4.11 | 0.67 | | 2.21 | | 6.24 | |  |
| 71_DR_03 | 3.8 | 4.5 | 0.81 | 3.3 | 0.69 | 0.38 | 0.77 | 0.11 | 0.70 | 0.14 | 0.41 | 0.05 | 0.37 | 0.053 | 13.1 | 2.99 | 16.1 | 4.38 | 0.62 | | 1.60 | | 7.34 | |  |
| 71_DR_04 | 3.8 | 6.4 | 0.86 | 3.5 | 0.79 | 0.51 | 0.97 | 0.15 | 1.04 | 0.22 | 0.66 | 0.09 | 0.64 | 0.107 | 15.4 | 4.38 | 19.7 | 3.51 | 0.86 | | 1.75 | | 4.21 | |  |
| 71_DR_07 | 2.9 | 3.2 | 0.64 | 2.7 | 0.54 | 0.31 | 0.63 | 0.09 | 0.57 | 0.12 | 0.34 | 0.05 | 0.31 | 0.048 | 9.94 | 2.47 | 12.4 | 4.03 | 0.58 | | 1.60 | | 6.53 | |  |
| 71_DR_09 | 29.7 | 48.1 | 7.23 | 29.6 | 6.44 | 1.93 | 7.39 | 1.11 | 7.54 | 1.56 | 4.72 | 0.69 | 4.94 | 0.783 | 121.1 | 30.7 | 151.7 | 3.95 | 0.80 | | 0.85 | | 4.30 | |  |
| 71_DR_12 | 6.9 | 10.2 | 1.48 | 6.0 | 1.22 | 0.70 | 1.36 | 0.20 | 1.28 | 0.26 | 0.74 | 0.10 | 0.70 | 0.105 | 25.8 | 5.45 | 31.3 | 4.74 | 0.78 | | 1.64 | | 7.02 | |  |
| 77_HY_02 | 5.9 | 17.3 | 1.81 | 7.5 | 1.71 | 1.05 | 1.72 | 0.25 | 1.62 | 0.30 | 0.86 | 0.12 | 0.80 | 0.126 | 34.2 | 6.86 | 41.1 | 4.98 | 1.29 | | 1.86 | | 5.23 | |  |
| 77_HY_03 | 8.6 | 21.1 | 2.92 | 12.1 | 2.63 | 1.64 | 2.63 | 0.39 | 2.47 | 0.47 | 1.31 | 0.19 | 1.22 | 0.180 | 47.4 | 10.5 | 57.9 | 4.51 | 1.02 | | 1.89 | | 5.05 | |  |
| 82_HY_01 | 16.6 | 28.8 | 3.99 | 16.6 | 3.58 | 0.92 | 4.10 | 0.60 | 3.97 | 0.80 | 2.34 | 0.34 | 2.25 | 0.347 | 69.5 | 15.7 | 85.1 | 4.44 | 0.86 | | 0.72 | | 5.27 | |  |
| 82_HY_05 | 13.6 | 11.1 | 3.18 | 13.6 | 2.92 | 0.95 | 3.59 | 0.54 | 3.89 | 0.85 | 2.65 | 0.39 | 2.65 | 0.438 | 44.4 | 15.9 | 60.3 | 2.78 | 0.41 | | 0.88 | | 3.66 | |  |
| 82_HY_06a | 0.8 | 0.7 | 0.14 | 0.6 | 0.11 | 0.04 | 0.17 | 0.03 | 0.18 | 0.04 | 0.12 | 0.02 | 0.10 | 0.017 | 2.34 | 0.70 | 3.04 | 3.32 | 0.55 | | 0.82 | | 5.39 | |  |
| 82_HY_06b | 0.1 | 0.2 | 0.02 | 0.1 | 0.01 | 0.00 | 0.01 | 0.00 | 0.01 | 0.00 | 0.01 | 0.00 | 0.00 | 0.001 | 0.35 | 0.04 | 0.39 | 10.05 | 0.97 | | 0.78 | | 18.59 | |  |
| 82_HY_09 | 11.9 | 30.2 | 2.99 | 12.3 | 2.82 | 0.69 | 3.43 | 0.54 | 3.72 | 0.76 | 2.30 | 0.34 | 2.30 | 0.384 | 60.3 | 14.5 | 74.7 | 4.17 | 1.24 | | 0.67 | | 3.70 | |  |
| 82_HY_10 | 19.3 | 25.3 | 4.48 | 18.9 | 3.99 | 1.05 | 4.75 | 0.70 | 4.93 | 1.06 | 3.24 | 0.47 | 3.05 | 0.522 | 72.0 | 19.8 | 91.7 | 3.64 | 0.66 | | 0.73 | | 4.53 | |  |
| Sample ID | La | Ce | Pr | Nd | Sm | Eu | Gd | Tb | Dy | Ho | Er | Tm | Yb | Lu | ΣLREE | ΣHREE | ΣREE | LREE/  HREE | | Ce/Ce* | | Eu/Eu* | | La_N_/Yb_N_ | |
| 82_HY_11 | 6.5 | 10.3 | 1.44 | 6.0 | 1.41 | 0.80 | 1.84 | 0.29 | 2.14 | 0.49 | 1.57 | 0.25 | 1.74 | 0.298 | 25.6 | 9.4 | 35.1 | 2.72 | | 0.82 | | 1.51 | | 2.66 | |
| 86_HY_02 | 0.2 | 0.5 | 0.05 | 0.2 | 0.03 | 0.02 | 0.02 | 0.00 | 0.02 | 0.00 | 0.01 | 0.00 | 0.01 | 0.001 | 0.94 | 0.08 | 1.02 | 11.60 | | 1.08 | | 1.79 | | 21.88 | |
| 86_HY_04 | 9.3 | 4.5 | 1.87 | 8.0 | 1.66 | 0.82 | 2.17 | 0.32 | 2.33 | 0.53 | 1.65 | 0.24 | 1.58 | 0.275 | 25.4 | 9.92 | 35.3 | 2.56 | | 0.26 | | 1.30 | | 4.22 | |
| 86_HY_07 | 0.9 | 1.4 | 0.14 | 0.5 | 0.07 | 0.03 | 0.05 | 0.01 | 0.05 | 0.01 | 0.03 | 0.00 | 0.02 | 0.004 | 2.89 | 0.19 | 3.08 | 14.93 | | 0.96 | | 1.67 | | 34.01 | |
| 86_HY_08 | 2.5 | 4.9 | 0.73 | 3.7 | 0.99 | 0.60 | 1.18 | 0.13 | 0.68 | 0.11 | 0.25 | 0.03 | 0.13 | 0.017 | 12.8 | 3.13 | 15.9 | 4.09 | | 0.89 | | 1.69 | | 13.16 | |
| 86_HY_09 | 1.2 | 0.9 | 0.14 | 0.5 | 0.10 | 0.55 | 0.13 | 0.02 | 0.14 | 0.04 | 0.11 | 0.02 | 0.12 | 0.020 | 2.84 | 1.16 | 3.99 | 2.46 | | 0.52 | | 14.79 | | 6.96 | |
| 87_MC_01 | 7.1 | 7.2 | 1.55 | 6.5 | 1.34 | 0.31 | 1.54 | 0.22 | 1.48 | 0.31 | 0.92 | 0.13 | 0.77 | 0.134 | 23.7 | 5.82 | 29.5 | 4.07 | | 0.53 | | 0.66 | | 6.57 | |
| 90_HY_04a | 3.6 | 1.8 | 0.66 | 2.8 | 0.60 | 0.44 | 0.79 | 0.12 | 0.85 | 0.20 | 0.66 | 0.10 | 0.67 | 0.112 | 9.49 | 3.93 | 13.4 | 2.42 | | 0.28 | | 1.93 | | 3.88 | |
| 90_HY_04b | 0.5 | 0.6 | 0.03 | 0.1 | 0.01 | 0.15 | 0.01 | <0.01 | 0.01 | <0.01 | 0.01 | <0.01 | <0.01 | 0.001 | 1.25 | 0.20 | 1.45 | 6.28 | | 1.14 | | 34.53 | | 85.94 | |
| 90_HY_04c | 0.2 | 0.2 | 0.01 | 0.0 | 0.01 | 0.02 | 0.01 | <0.01 | 0.01 | <0.01 | 0.01 | <0.01 | <0.01 | 0.002 | 0.46 | 0.06 | 0.52 | 7.85 | | 1.00 | | 5.55 | | 29.09 | |
| 90_HY_05 | 17.4 | 31.2 | 4.44 | 18.2 | 4.08 | 1.63 | 4.45 | 0.67 | 4.35 | 0.84 | 2.36 | 0.34 | 2.22 | 0.350 | 75.3 | 17.2 | 92.5 | 8.73 | | 0.87 | | 1.16 | | 5.58 | |
| 90_HY_06 | 26.5 | 45.8 | 6.53 | 26.7 | 5.78 | 2.15 | 6.35 | 0.93 | 5.99 | 1.15 | 3.20 | 0.45 | 2.77 | 0.448 | 111.2 | 23.4 | 134.7 | 7.53 | | 0.85 | | 1.08 | | 6.83 | |
| 94_HY_01 | 15.7 | 26.7 | 4.12 | 16.4 | 3.56 | 1.16 | 3.71 | 0.56 | 3.76 | 0.75 | 2.21 | 0.33 | 2.18 | 0.355 | 66.5 | 15.0 | 81.6 | 6.85 | | 0.81 | | 0.96 | | 5.15 | |
| 94_HY_04 | 14.6 | 16.2 | 3.21 | 13.5 | 2.97 | 1.54 | 3.45 | 0.49 | 3.33 | 0.72 | 2.18 | 0.31 | 2.02 | 0.342 | 50.5 | 14.4 | 64.9 | 3.51 | | 0.58 | | 1.46 | | 5.17 | |
| 94_HY_05 | 5.7 | 15.3 | 2.19 | 10.0 | 2.70 | 1.51 | 2.79 | 0.41 | 2.49 | 0.46 | 1.36 | 0.21 | 1.40 | 0.219 | 35.9 | 10.9 | 46.7 | 3.31 | | 1.06 | | 1.67 | | 2.90 | |
| 94_HY_06 | 4.7 | 6.1 | 1.06 | 4.6 | 0.96 | 0.46 | 1.30 | 0.19 | 1.31 | 0.29 | 0.87 | 0.12 | 0.75 | 0.124 | 17.4 | 5.41 | 22.8 | 3.22 | | 0.67 | | 1.24 | | 4.49 | |
| 102_HY_03 | 24.3 | 37.3 | 6.15 | 24.9 | 5.56 | 1.75 | 5.95 | 0.88 | 5.62 | 1.08 | 3.01 | 0.42 | 2.65 | 0.404 | 98.2 | 21.8 | 120.0 | 5.25 | | 0.74 | | 0.92 | | 6.55 | |
| 102_HY_04 | 8.9 | 5.7 | 1.58 | 6.4 | 1.33 | 0.36 | 1.63 | 0.23 | 1.64 | 0.35 | 1.06 | 0.15 | 0.96 | 0.151 | 24.0 | 6.53 | 30.5 | 3.67 | | 0.37 | | 0.75 | | 6.64 | |
| 102_HY_05 | 3.1 | 2.9 | 0.78 | 3.4 | 0.77 | 0.48 | 0.94 | 0.14 | 0.92 | 0.20 | 0.58 | 0.08 | 0.57 | 0.092 | 10.9 | 4.00 | 14.9 | 2.74 | | 0.45 | | 1.70 | | 3.88 | |
| 102_HY_06 | 90.8 | 114.1 | 22.29 | 91.0 | 19.01 | 4.69 | 20.33 | 2.92 | 18.47 | 3.48 | 9.57 | 1.32 | 8.56 | 1.276 | 337.2 | 70.6 | 407.9 | 4.78 | | 0.62 | | 0.72 | | 7.59 | |
| 102_HY_07 | 38.4 | 49.6 | 9.20 | 37.2 | 7.85 | 2.88 | 8.37 | 1.19 | 7.41 | 1.38 | 3.70 | 0.51 | 3.14 | 0.476 | 142.3 | 29.1 | 171.3 | 8.62 | | 0.64 | | 1.08 | | 8.73 | |

## ESM2.5: Pb-Nd-Sr isotope systematics

**Table S2.10** Sr-Nd-Pb isotopic composition of the Fe-oxyhydroxide and sulphide (82_HY_06_b_ & 90_HY_04_b_) samples at Semenov hydrothermal field. 2SE – two-standard error.

| **Sample ID** | ^206^Pb/^204^Pb | 2SE | ^207^Pb/^204^Pb | 2SE | ^208^Pb/^204^Pb | 2SE | ^87^Sr/^86^Sr | 2SE | ^143^Nd/^144^Nd | 2SE | εNd |
| --- | --- | --- | --- | --- | --- | --- | --- | --- | --- | --- | --- |
| 28_DR_03 | 18.8546 | 0.0002 | 15.5490 | 0.0010 | 38.3690 | 0.0032 | 0.709036 | 0.000005 | 0.512156 | 0.000006 | -9.40 |
| 39_HY_02 |  |  |  |  |  |  | 0.707028 | 0.000007 | 0.512106 | 0.000007 | -10.39 |
| 66_HY_05 | 18.8858 | 0.0002 | 15.5775 | 0.0008 | 38.5510 | 0.0020 | 0.708939 | 0.000006 | 0.512067 | 0.000007 | -11.13 |
| 71_DR_01 | 19.0127 | 0.0002 | 15.5708 | 0.0008 | 38.6250 | 0.0022 | 0.708498 | 0.000007 | 0.512074 | 0.000006 | -11.00 |
| 82_HY_01 | 18.9306 | 0.0002 | 15.5639 | 0.0009 | 38.4990 | 0.0025 | 0.709185 | 0.000006 | 0.512022 | 0.000005 | -12.02 |
| 82_HY_05 | 18.9278 | 0.0002 | 15.5565 | 0.0008 | 38.4620 | 0.0023 | 0.708463 | 0.000006 | 0.512066 | 0.000006 | -11.16 |
| 82_HY_06_a_ | 18.9345 | 0.0002 | 15.5570 | 0.0009 | 38.4680 | 0.0022 |  |  |  |  |  |
| 82_HY_06_b_ | 18.9324 | 0.0002 | 15.5541 | 0.0008 | 38.4600 | 0.0023 |  |  |  |  |  |
| 82_HY_09 | 18.9431 | 0.0002 | 15.5791 | 0.0008 | 38.5620 | 0.0023 | 0.709220 | 0.000008 | 0.512003 | 0.000007 | -12.39 |
| 82_HY_10 | 18.9306 | 0.0002 | 15.5637 | 0.0009 | 38.4980 | 0.0021 | 0.709222 | 0.000006 | 0.512038 | 0.000008 | -11.70 |
| 86_HY_04 | 18.8471 | 0.0002 | 15.5440 | 0.0009 | 38.3440 | 0.0022 | 0.707575 | 0.000005 | 0.512097 | 0.000006 | -10.56 |
| 86_HY_09 | 18.8627 | 0.0002 | 15.5456 | 0.0009 | 38.3660 | 0.0020 |  |  |  |  |  |
| 90_HY_04_b_ | 18.8661 | 0.0003 | 15.545 | 0.0011 | 38.3720 | 0.0023 |  |  |  |  |  |
| 94_HY_01 | 18.8661 | 0.0002 | 15.5562 | 0.0009 | 38.4310 | 0.0056 | 0.707953 | 0.000006 | 0.512082 | 0.000006 | -10.84 |
| 94_HY_04 | 18.8610 | 0.0002 | 15.5487 | 0.0008 | 38.3690 | 0.0020 | 0.707482 | 0.000006 | 0.512222 | 0.000007 | -8.11 |
| 94_HY_05 | 18.8580 | 0.0002 | 15.5464 | 0.0008 | 38.3600 | 0.0025 |  |  | 0.512381 | 0.000007 | -5.02 |
| 94_HY_06 | 18.8470 | 0.0001 | 15.5501 | 0.0008 | 38.3660 | 0.0020 | 0.708237 | 0.000005 | 0.512234 | 0.000007 | -7.87 |
| 102_HY_03 | 18.8938 | 0.0002 | 15.5534 | 0.0007 | 38.4000 | 0.0021 | 0.708715 | 0.000006 | 0.512148 | 0.000006 | -9.56 |
| 102_HY_04 | 18.8548 | 0.0002 | 15.5647 | 0.0007 | 38.4400 | 0.0019 | 0.709061 | 0.000006 | 0.512043 | 0.000008 | -11.60 |
| 102_HY_05 | 18.8618 | 0.0002 | 15.5462 | 0.0008 | 38.3610 | 0.0019 | 0.708593 | 0.000006 | 0.512213 | 0.000008 | -8.29 |
| 102_HY_06 | 18.8771 | 0.0002 | 15.5676 | 0.0008 | 38.4710 | 0.0019 | 0.708481 | 0.000006 | 0.512087 | 0.000009 | -10.75 |

## ESM2.6: Estimated sediment component of bulk FeOOH samples


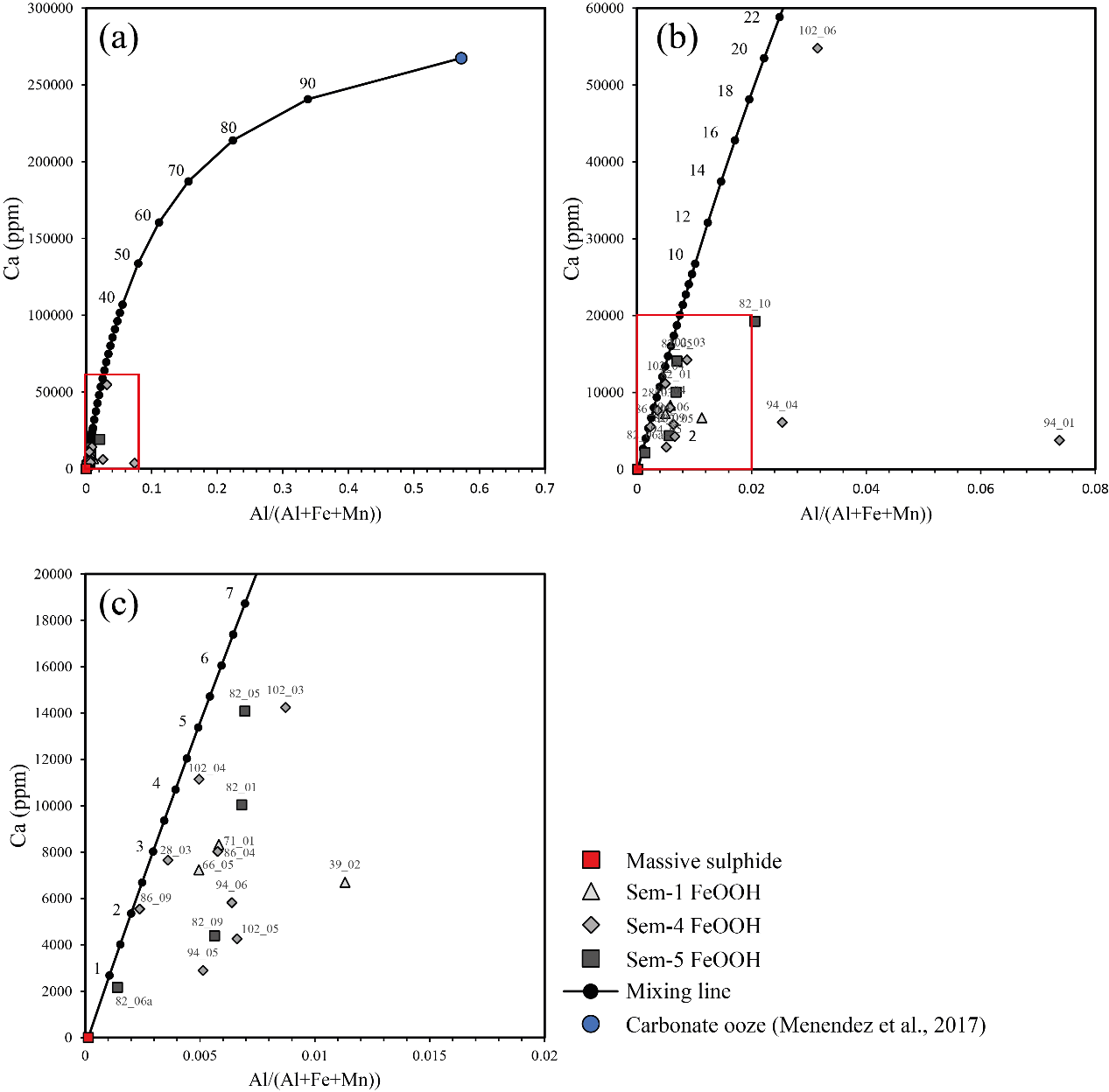


**Figure S2.1** Sulphide-sediment theoretical mixing lines produced from Eq. 1 and Eq. 2. Values of % sediment acquired from Al/(Al+Fe+Mn) vs Ca (ppm) mixing lines N/A values are either where no isotopic analysis was measured or FeOOH Pb isotopic ratio lies below that of the massive sulphide or sulphide-sediment mix and therefore, unable to obtain a W/R value. Note values are approximate.

## ESM2.7: Calculated F/R ratio

**Table S2.11.** Derived F/R ratio of FeOOH samples at Semenov with estimated sediment content derived from Figure S2.1. N/A values where FeOOH Pb isotopic ratio lies below that of the massive sulphide or sulphide-sediment mix or deviate considerably from the mixing line and therefore, unable to obtain a F/R value. Note values are approximate.

| Sample | ^206^Pb/^204^Pb | ^207^Pb/^204^Pb | ^208^Pb/^204^Pb | Estimated sediment content (%) | Calculated  F/R ratio |
| --- | --- | --- | --- | --- | --- |
| 28_03 | 18.855 | 15.549 | 38.368 | 3.0 | 8.5x10^5^ |
| 66_05 | 18.886 | 15.577 | 38.551 | 2.5 | 1.0x10^7^ |
| 82_01 | 18.931 | 15.564 | 38.498 | 4.0 | 1.0x10^6^ |
| 82_05 | 18.928 | 15.557 | 38.462 | 5.5 | 1.0x10^5^ |
| 82_06a | 18.935 | 15.557 | 38.468 | 0.8 | 3.0x10^5^ |
| 82_09 | 18.943 | 15.579 | 38.562 | 1.5 | 3.0x10^6^ |
| 82_10 | 18.931 | 15.564 | 38.498 | 7.5 | 8.5x10^5^ |
| 86_04 | 18.847 | 15.544 | 38.344 | 3.0 | N/A |
| 86_09 | 18.863 | 15.546 | 38.367 | 2.0 | 1.5x10^4^ |
| 94_01 | 18.866 | 15.556 | 38.431 | 1.5 | 3.0x10^6^ |
| 94_04 | 18.861 | 15.549 | 38.369 | 2.5 | 8.0x10^5^ |
| 94_05 | 18.858 | 15.546 | 38.360 | 1.0 | 2.0x10^5^ |
| 94_06 | 18.847 | 15.550 | 38.366 | 2.0 | N/A |
| 102_03 | 18.894 | 15.553 | 38.400 | 5.5 | 2.0x10^6^ |
| 102_04 | 18.855 | 15.565 | 38.440 | 4.0 | N/A |
| 102_05 | 18.862 | 15.546 | 38.361 | 1.5 | 1.5x10^5^ |
| 102_06 | 18.877 | 15.568 | 38.471 | 21.0 | 6.0x10^6^ |
